# The Identification of Circulating MiRNA in Bovine Serum and Their Potential as Novel Biomarkers of Early *Mycobacterium avium* subsp *paratuberculosis* Infection

**DOI:** 10.1371/journal.pone.0134310

**Published:** 2015-07-28

**Authors:** Damien Farrell, Ronan G. Shaughnessy, Louise Britton, David E. MacHugh, Bryan Markey, Stephen V. Gordon

**Affiliations:** 1 UCD School of Veterinary Medicine, University College Dublin, Dublin, Ireland; 2 UCD School of Agriculture and Food Science, University College Dublin, Dublin, Ireland; 3 UCD School of Medicine, University College Dublin, Dublin, Ireland; 4 UCD School of Biomolecular and Biomedical Science, University College Dublin, Dublin, Ireland; 5 UCD Conway Institute, University College Dublin, Dublin, Ireland; Fundació Institut d’Investigació en Ciències de la Salut Germans Trias i Pujol. Universitat Autònoma de Barcelona. CIBERES, SPAIN

## Abstract

*Mycobacterium avium* subspecies *paratuberculosis* (MAP) is the aetiological agent of Johne’s disease (JD), a chronic enteritis in ruminants that causes substantial economic loses to agriculture worldwide. Current diagnostic assays are hampered by low sensitivity and specificity that seriously complicate disease control; a new generation of diagnostic and prognostic assays are therefore urgently needed. Circulating microRNAs (miRNAs) have been shown to have significant potential as novel biomarkers for a range of human diseases, but their potential application in the veterinary sphere has been less well characterised. The aim of this study was therefore to apply RNA-sequencing approaches to serum from an experimental JD infection model as a route to identify novel diagnostic and prognostic miRNA biomarkers. Sera from experimental MAP-challenged calves (n = 6) and age-matched controls (n = 6) were used. We identified a subset of known miRNAs from bovine serum across all samples, with approximately 90 being at potentially functional abundance levels. The majority of known bovine miRNAs displayed multiple isomiRs that differed from the canonical sequences. Thirty novel miRNAs were identified after filtering and were found within sera from all animals tested. No significant differential miRNA expression was detected when comparing sera from MAP-challenged animals to their age-matched controls at six-month’s post-infection. However, comparing sera from pre-infection bleeds to six-month’s post-infection across all 12 animals did identify increased miR-205 (2-fold) and decreased miR-432 (2-fold) within both challenged and control groups, which suggests changes in circulating miRNA profiles due to ageing or development (P<0.00001). In conclusion our study has identified a range of novel miRNA in bovine serum, and shown the utility of small RNA sequencing approaches to explore the potential of miRNA as novel biomarkers for infectious disease in cattle.

## Introduction

Johne’s disease is a chronic enteritis of ruminants that is caused by *Mycobacterium avium* subspecies *paratuberculosis* (MAP) infection [1]. Clinical features such as prolonged diarrhoea, progressive wasting and reduced milk yields have obvious implications for animal health and welfare and translate into substantial economic losses, with the dairy industry being particularly vulnerable [2]. In herds where MAP infection is a problem significant resources are therefore expended in reducing the prevalence and transmission of MAP [3].

MAP infection stages can be sequentially classed as silent, subclinical, clinical and advanced [4]. T_H_1 immune responses are characteristic of silent infections, with IFN-**γ** being the hallmark cytokine, and which can effectively contain mycobacterial infection [5]. This cell-mediated response prevails within 85–90% of infected animals and prevents the onset of clinical signs and bacterial transmission. The other 10–15% of animals eventually enter the subclinical phase, where T_H_1 responses gradually decline and T_H_2 responses become more dominant [4,5]. The T_H_2 humoral response offers little protection against disease progression and faecal shedding of MAP commences [6]. At this point, transmission within herds through the faecal-oral route is enabled and becomes more established as shedding increases in line with the onset and progression of the clinical disease. Isolation of infected animals in the early subclinical phase before shedding is established would serve to halt MAP dissemination within herds. However, the ability to accurately identify animals at an early stage of infection that are progressing towards shedding is critical to the success of such a disease control approach and requires a robust prognostic biomarker.

Faecal culture is regarded as the “gold standard” for the diagnosis of MAP infection but requires lengthy incubation periods, and sensitivity can be low in early subclinical infections [7]. Faecal PCR assays are less reliable in the early stages of disease, with one study reporting a detection rate of 4% for low to moderate MAP shedders [8]. IFN-**γ** based diagnostics have problems of specificity, as the crude MAP antigen preparations used in the whole-blood stimulations show cross-reactivity to antigens from other, environmental mycobacterial species. Similar specificity issues also arise with the available serological assays [9]. In addition, serological assays are of low sensitivity and MAP-specific antibodies are only detected in about 15% of subclinical cases [10]. Overall, it is clear that novel and reliable MAP-specific diagnostic and prognostic assays are required.

Recently, microRNAs (miRNAs) have attracted attention as potential prognostic and diagnostic biomarkers for numerous human pathologies [11]. These short (~22 nt) non-coding RNAs regulate mRNA expression and are predicted to target at least one-third of known mammalian genes [12,13]. Some of the key miRNAs that are known to regulate immune responses to intracellular pathogens have been reported to be upregulated in several cell types in response to mycobacterial infections [14,15]. Thus, specific miRNA expression signatures in tissue samples appear to reflect the underlying host-defence processes in different tissues/cells, and it is possible that different stages of infection have distinct miRNA signatures. Identifying miRNA signatures in the circulation is a more attractive option, as sampling is less invasive and the extracellular miRNAs present are resistant to degradation due to their containment within vesicles or association with proteins [11,16]. Several studies have shown that miRNA profiles in human sera samples of active tuberculosis (TB) patients differ from those of healthy controls [17–21]. It is therefore possible that specific circulating miRNA profiles exist for other mycobacterial infections, including Johne’s disease in cattle.

miRNA-sequencing can simultaneously identify and quantify the full repertoire of miRNAs in the circulation at any specific physiological state. A major hindrance with sequencing a cellular body fluids, however, is that their RNA concentrations are below the requirements of most commercial (small RNA) library preparation kits. Nevertheless, there has been some recent notable successes with small biofluid volumes [22,23], suggesting that this approach may be applicable under similar circumstances.

Our hypothesis in this study was that MAP infection would cause significant changes in the miRNA profile of bovine serum and thus offer a novel diagnostic modality where MAP infection could be disclosed via miRNA profiles. To explore this, we applied RNA sequencing approaches to samples from an experimental MAP infection model. Small RNA-sequencing for miRNA detection on bovine serum has yet to be reported and thus a key aim of this study was to determine whether miRNAs could be sequenced from low serum volumes (≤ 1ml). In our infection model, 3–6 week old calves were infected with a high MAP-challenge dose, and the course of infection followed over the first 6 months of infection. This early period would typically represent the silent phase of infection, when faecal shedding and any serological response would be negative. Parallel whole blood IFN-**γ** responses to MAP antigens (PPD-J) provided an immune reference in which to place the miRNA response. A data analysis pipeline was developed for bovine miRNA discovery, and a number of novel miRNAs were identified. Comparison of the miRNA repertoire between infected and control animals allowed an initial assessment of the potential of miRNAs as biomarkers of early silent stage MAP infection.

## Materials and Methods

### Bacterial cultivation and experimental challenge

*Mycobacterium avium* subspecies *paratuberculosis* (MAP) strain CIT003 (a gift from Dr Jim O’Mahony, Cork Institute of Technology, Ireland) was cultivated to mid-exponential phase in a modified Middlebrook 7H9 broth consisting of 0.47% Middlebrook 7H9 powder (Becton Dickinson, Dublin, Ireland), 0.05% Casitone (Becton Dickinson, Dublin, Ireland), 0.25% Glycerol (Sigma-Aldrich, Wicklow, Ireland), 10% Middlebrook OADC (Becton Dickinson, Dublin, Ireland) and 2 **μ**g/ml Mycobactin J (Synbiotics, Lyon, France) at 37°C with rolling at 4 rpm. Mycobacterial CFU numbers were estimated using both the “pelleted wet weight method” [24] and optical densitometry [25]. Based on these quantification approaches, an inoculum dose was prepared to represent approximately 2 × 10^9^ CFU; this was used to infect thirty-five male Holstein-Friesian calves between three to six weeks of age, with the inoculum administrated orally on two consecutive days. Retrospective plate counts using Middlebrook 7H11 agar (Becton Dickinson, Dublin, Ireland) supplemented with Mycobactin J revealed the viable challenge dose to be 3.8 × 10^9^ CFU. In parallel to the challenged animals, a control group of twenty calves received a placebo consisting of sterile 7H9 broth. Blood samples were routinely collected from all animals in lithium heparin vacutainers (Becton Dickinson, Dublin, Ireland). At intervals over the first six months of the experiment, cell mediated immunity was investigated using the BOVIGAM IFN-**γ** release assay (Prionics, Life Technologies) according to the manufacturer’s specification, with IFN-**γ** levels in whole blood samples being quantified following stimulations with MAP-derived PPD-J (22 **μ**g/ml; a gift from Dr Douwe Bakker, CVI, Netherlands). Sera was prepared by centrifuging clotted blood samples from silicon-coated (red top) vacutainers (Becton Dickinson, Dublin, Ireland) at 1200 × *g* for 20 mins. The collected sera were immediately stored at -80°C. Sera were routinely screened for screened for MAP-specific antibodies using ELISA (IDEXX). Faecal samples were collected from all animals prior to MAP-challenge and at intervals post-infection. These were cultured for 42 days using the TREK ESP para-JEM system (Thermo Scientific) according to the manufacturer’s recommendations. The animal work was approved by the Animal Research Ethics Committee of University College Dublin (AREC-P-12-61-Markey) and licensed by the Irish Government Department of Health and Children (B100-2828).

### RNA extraction

1 ml serum samples were incubated with 5 ml of QIAzol Lysis Reagent (Qiagen, Manchester, United Kingdom) for 10 mins at room temperature. RNA was subsequently extracted from the lysate using the miRNeasy Mini Kit (Qiagen, Manchester, United Kingdom) according to the manufacturer’s specifications. To precipitate the RNA samples, the 60 **μ**l eluates were mixed with 1/10 volumes of 3M sodium acetate pH 5.2 (Sigma-Aldrich, Wicklow, Ireland), 1 **μ**l of glycogen (Invitrogen, Ireland) and 3 volumes of 100% ethanol (Sigma-Aldrich, Wicklow, Ireland) and stored overnight at -80°C. The samples were subsequently centrifuged at 16,000 × *g* for 25 mins at 4°C. The resulting pellets were washed with 70% ethanol, re-suspended in 6 **μ**l of nuclease-free water and stored at -80°C.

### Small RNA sequencing

Libraries were constructed from 5 **μ**l of the prepared RNA using the TruSeq Small RNA Sample Preparation Kit (Illumina, Eindhoven, Netherlands) with slight deviations from the manufacturer’s protocol. Fifteen cycles of PCR were performed, and the resulting amplicons were purified using an Agencourt AMPure XP kit (Beckman Coulter, Ireland). Briefly, 1.8× concentrations of AMPure beads were incubated with the library preparations for 15 mins. The bound PCR products were then washed twice with 80% ethanol, and subsequently re-suspended in 20 **μ**l of nuclease-free water. The quality and quantity of each library preparation was determined using Agilent DNA 1000 chips (Agilent Technologies, Cork, Ireland) with an Agilent 2100 Bioanalyzer. Each construct was diluted to approximately 20 nM, and randomly assigned to one of two different pools. Electrophoresis using Novex 8% TBE Gels (Invitrogen, Ireland) was used to purify the pools and minimise adapter dimer contamination. The pools were each clustered in two lanes of an Illumina HiSeq 2500 Rapid Run Flow Cell (v1) and sequenced in a SE50bp format using Rapid SBS reagents (sequencing performed at Michigan State University RTSF Genomics Core, Michigan, USA).

Sequencing data is available in the ArrayExpress database (www.ebi.ac.uk/arrayexpress) under accession number E-MTAB-3445.

### Identification of known and novel miRNAs

miRDeep2 was used to quantify the reads that aligned to known mature miRNAs and for the prediction of potential novel miRNAs [26]. miRDeep2 uses modules to separate the steps of miRNA quantification and novel prediction. The quantifier module maps to a fasta file of known precursors provided by the user (e.g. miRBase for known mature sequences). The output is a table of read counts and read counts normalised to library size that may be used for expression studies. The core miRDeep.pl module performs prediction and scoring of novel microRNAs using the canonical model of micro RNA biogenesis and by examining the patterns of reads aligned to the reference genome (using the mapper.pl module). Putative miRNAs are scored based on the read stack pattern, stability of the pre-miRNA hairpin and homology to previously identified miRNAs. The expression profile of the novel miRNAs is then also quantified. miRBase version 21 was used for known miRNA mapping.

Before submission to miRDeep2, the adapters were trimmed with cutadapt discarding all reads <18nt. Known tRNA mappable reads were then identified using Bowtie (only reads with zero mismatches were mapped) and the remaining reads used for analysis. An important aspect of the miRDeep2 results are the performance statistics which give an indication of appropriate score cut-offs by estimating false positives at each score level. Our results showed poor signal-to-noise even at high score levels likely owing to the relatively small percentage of mappable miRNAs in each sample. Therefore we chose a stringent score cut-off of 4 (signal-to-noise = 5.5) for the novel miRNAs and a cut-off of 0 for the known set. An additional reads-based filter was used to remove the remaining low abundance hits remaining from the miRDeep2 analysis. This filter removed all results with reads present in less than 50% of samples and mean normalised read count ≤150. Another algorithm, sRNAbench [27], was also applied to the data mainly to serve as a comparison and for isomiR profiling.

### Benchmarking of miRDeep2 and comparison with sRNAbench

To get an indication of read depth required for adequate miRNA discovery we took a single file from our dataset and ran both mirDeep2 and sRNAbench iteratively for increasing random subsets of the reads, ranging from 0.5 to 7 million reads, increasing by 1 million at each run.

### Identification of small RNA fractions

To ascertain the degree of contamination in each sample caused by degraded nucleic acid and other small RNA species, we applied a custom Python script to each sample. FastQC was used initially to determine the quality but no reads needed to be removed due to low quality. Corresponding samples from two sequencing lanes were combined together by concatenating the files.

We then applied the following steps for each sample:

1. The 3' adapter was trimmed and quality trimming was performed with q>20 using cutadapt.
2. The cleaned reads were collapsed with the copy number of each unique read retained.
3. Collapsed reads were successively mapped with Bowtie [28] to multiple small RNA bovine annotations according to the category given below. All mapped reads were discarded at each iteration and the unmapped remainder passed on to the next mapping. miRbase (release 21). The Bowtie parameters were set to allow 1 mismatch: **bowtie-f-S-v 1—best–un <remaining><input collapsed fasta>><outfile>**
4. The total percentage of reads mapping to each class was calculated using the original copy numbers.

The following RNA categories were used for mapping: known miRNA found from miRDeep2, tRNAs from the Genomic tRNA Database [29], rRNAs from RFAM [30], other non-coding RNAs from NONCODE [31], snRNA (downloaded from Ensembl BioMart) and the *Bos taurus* reference genome (UMD 3.1).

### Conservation analysis for novel miRNAs

A computational method to find conservation across mammal species was used to aid verification of novel miRNAs. This approach is somewhat similar to the one used by the authors of miRNAminer [32]. A custom Python script using the pyCogent [33] library was written that utilises the Ensembl Compara programming interface. For each novel miRNA, syntenic alignments from Ensembl (39-way mammal low coverage EPO ensembl 79 [34]) were obtained for a set of 12 species with the most complete assemblies. The following metrics were computed for any aligned syntenic regions in available mammal species:

- sequence identity
- if the seed region was conserved
- if the miRNA is found inside a known gene, the gene type (e.g. protein coding) and whether located inside an exon or intron
- whether there was base pair complementary of the seed in the 3' UTR of any enclosing gene

The results were filtered to produce a list of likely conserved miRNAs (those with ≥90% identity in at least one other species) and the remainder considered to be non-conserved.

### Differential expression analysis

EdgeR [35] was used to perform pairwise differential expression (DE) analyses for both conditions (experimentally infected and control) at each time point. Only the core miRNAs were included in the DE analysis since the remainder would be missing in too many samples (or too low abundance) to be statistically significant. Only samples with FDR <0.05 based on Benjamini and Hochberg multiple testing correction [36] and log fold change >1.5 were considered significant.

### Determination of a threshold read count for relevant miRNA selection

The read count threshold used to filter the miRDeep results was arrived at by using the method of Koh et al. [37] to estimate the minimum threshold of biological significance of read counts across replicates. Briefly, this method utilises the observation that the read count distribution of each sample can be interpreted as a mixture of two essentially distinct distributions. The low-abundant count provides noise-rich data and is followed by a fairly even distribution across the log scale of counts, tailing off with sporadic high counts representing the high abundance genes. This latter distribution should be similar between replicates since it represents the more consistently expressed transcripts. By iteratively removing the lowest counts from two replicates and comparing the count distributions using the Komologorov-Smirnov (KS) test it is possible to estimate the point at which replicates gain a high level of similarity. The read count threshold at which the KS value reaches a minimum is selected since it represents the point at which distributions become similar. This is illustrated for our data in S4 Fig.

The Python scripts used to perform all computational work described here are available at https://github.com/dmnfarrell/mirnaseq

## Results

### Interferon-gamma release assay (IGRA) and selection of animals for sequencing

**Fig 1:**
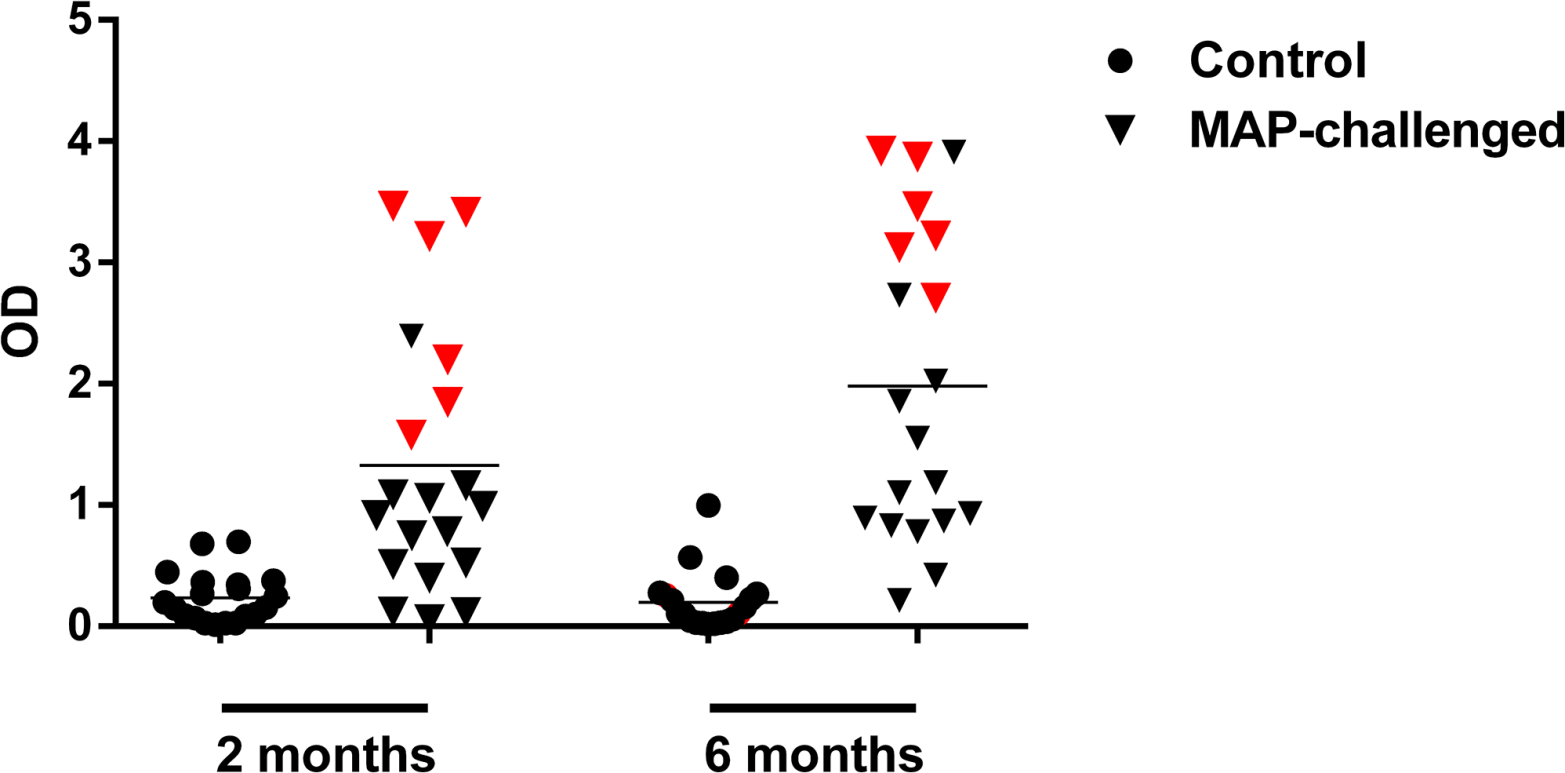
Responses to PPD-J in both sets of animals. IFN-**γ** production in control (n = 20) and MAP-challenged (n = 20) cattle at 2 months and 6 months post-infection in whole blood in response to PPD-J. Each point represents an individual animal while the red points indicate the six MAP-challenged cattle selected for miRNA-sequencing. The horizontal lines represent mean OD_450_ values for each plot.

At the three month and six month intervals, all cattle lacked MAP-specific antibodies in their sera and were negative for faecal shedding of MAP (data not shown). This suggested that the animals were still in the silent phase of infection and consequently, assessing cell mediated immunity was deemed appropriate for measuring infection status. The BOVIGAM IFN-**γ** release assay was used to measure IFN-**γ** production in whole blood after stimulation with MAP-purified protein derivative (PPD-J). From the experimental animals, 20 MAP-challenged cattle and 20 control cattle were selected for IFN-**γ** analysis at two-months and six-months post-infection. At both intervals, control animals showed minimal responsiveness to PPD-J, while IFN-**γ** responses were variable across the challenged animals (Fig 1). Six challenged cattle that had consistently high IFN-**γ** production at the 2 and 6 month time points post-infection were selected for miRNA-sequencing (highlighted in red, Fig 1). Six animals were selected at random from the control group to serve as age-matched comparisons. The 0 and 6 month time-points were chosen for sequencing analysis to represent two extremes as IFN-**γ** responses showed maximum differences between groups at these time points.

### Identification of small RNAs within bovine serum via sequencing

**Fig 2:**
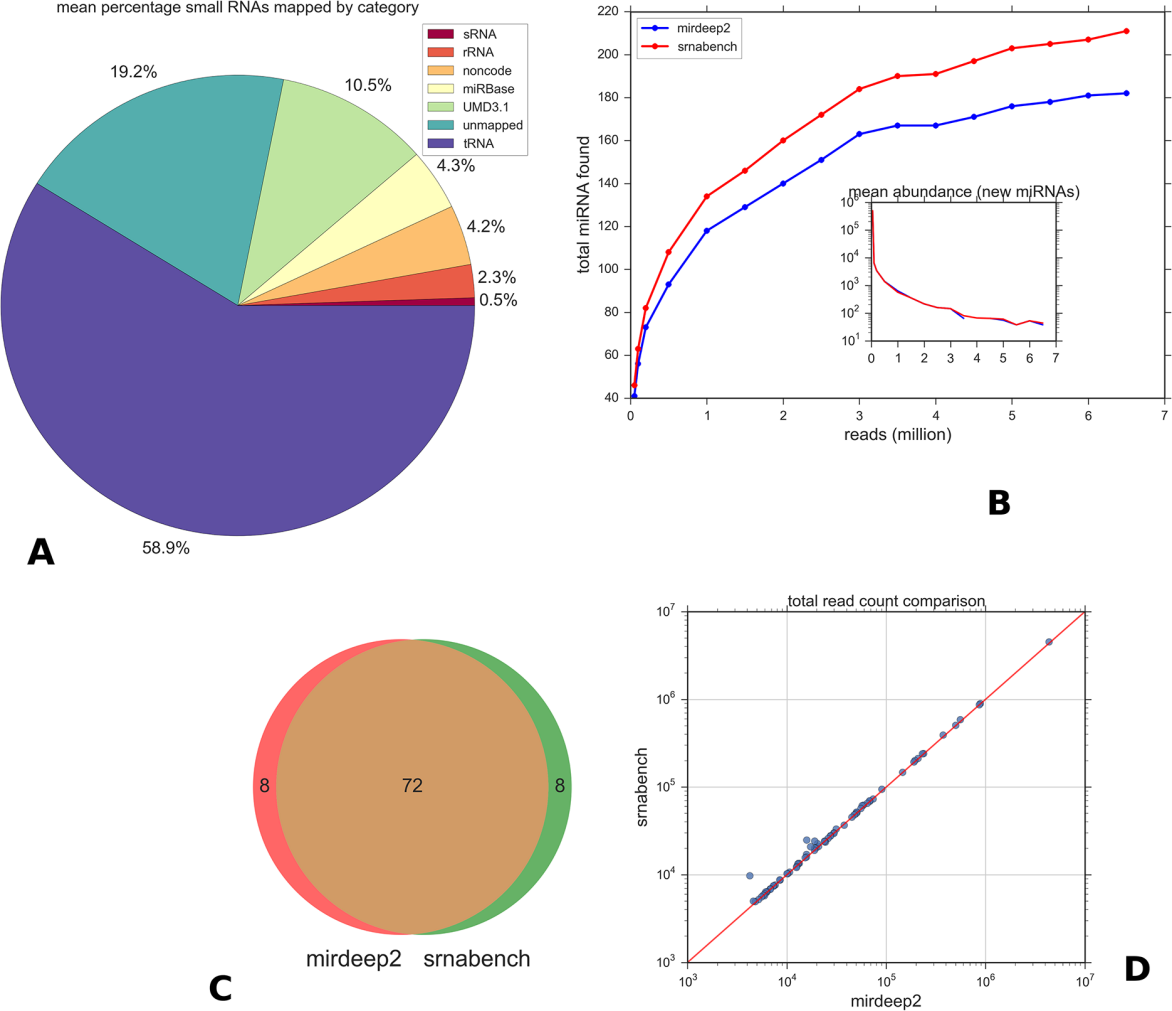
miRNA abundances in miRDeep2 and sRNAbench. A: Proportion of reads mapped to different small RNAs. Values are the average percentage over all samples. Reads matching tRNA dominate. The unmapped percentage is likely due to mismatches in the reads or unannotated ncRNAs. B: Variation of known miRNA discovered with increasing random reads for both methods used in this study. sRNAbench is more sensitive to low abundance genes and produces more hits at a given file size. miRDeep2 was run with no score cut-off. Discovery tails off at 4 million reads in both methods. The inset plot shows the mean abundance of each newly discovered set, illustrating that only low abundance miRNAs are being added after ~3 million mark. C: Overlap between the top 80 miRDeep and sRNAbench results for known miRNAs shows almost identical results. D: Correlation between total read counts determined by both methods for the overlapping miRNAs.

To the best of our knowledge, this is the first report of successful microRNA sequencing from bovine serum. Thoroughly characterising the serum-specific miRNA repertoire, to identify both known and novel miRNA, will provide valuable insight into circulating miRNAs in cattle. We conducted miRNA-seq on 24 sample libraries resulting in a total of 352,249,513 raw reads. Mapping with Bowtie to *Bos taurus* RNA annotations revealed that only a small percentage of reads in each sample (mean 4.3%) mapped to miRNAs. On average, 58% mapped to tRNA and ~7% to other non-coding RNA databases, while 19% of reads could not be reliably mapped at all (Fig 2A). No alignments were found to the MAP genome. There were significant variations in small RNA composition between individual samples (S1 Fig) but the general trend was consistent. A plot of the typical read length distribution in a sample (S3 Fig) highlights the large amount of degraded small RNA product present in relation to the smaller lengths in the 18–22 range represented by miRNAs.

### Read depth analysis and method comparison

To estimate whether the total number of reads were sufficient for miRNA discovery, read depth was compared against the number of miRNAs detected. With the aid of both prediction methods, miRDeep2 and sRNAbench, gradually increasing the read depth correlated with a gradual increase in specific miRNAs. However, the numbers of newly identified miRNAs reached a saturation point at approximately 4 million reads (Fig 2B). Beyond this point, only low abundance genes are newly found by both methods and the majority of these are filtered out in actual discovery due to their low levels and low scores. This indicates that the sequencing depth used in this study is more than sufficient for miRNA discovery.

There was a high degree of correlation between sRNAbench and miRDeep2 with regard to the filtered known miRNAs detected (Fig 2C) and their respective total abundance (Fig 2D). In general sRNAbench assigned a slightly higher number of total reads. This is likely due to slight differences in how the canonical miRNA is counted and how length variants and non-templated additions (addition of a nucleotide to the 3' end) are dealt with. Significant differences were found in total read count for only 3 miRNAs (notably bta-miR-99a-5p and bta-miR-99b) as shown by the outlying point in Fig 2D. IsomiR analysis showed that multiple short sub-dominant 3' length variants are seen for these miRNAs by sRNAbench but those reads were not counted by miRDeep2 due to their short length.

For the analyses that follow we used miRDeep2 for expression analyses and novel miRNA discovery, as it provides a good estimate of false positive miRNAs. sRNAbench was used for isomiR profiling because of ease in handling its output.

### Known miRNA profiles

**Fig 3:**
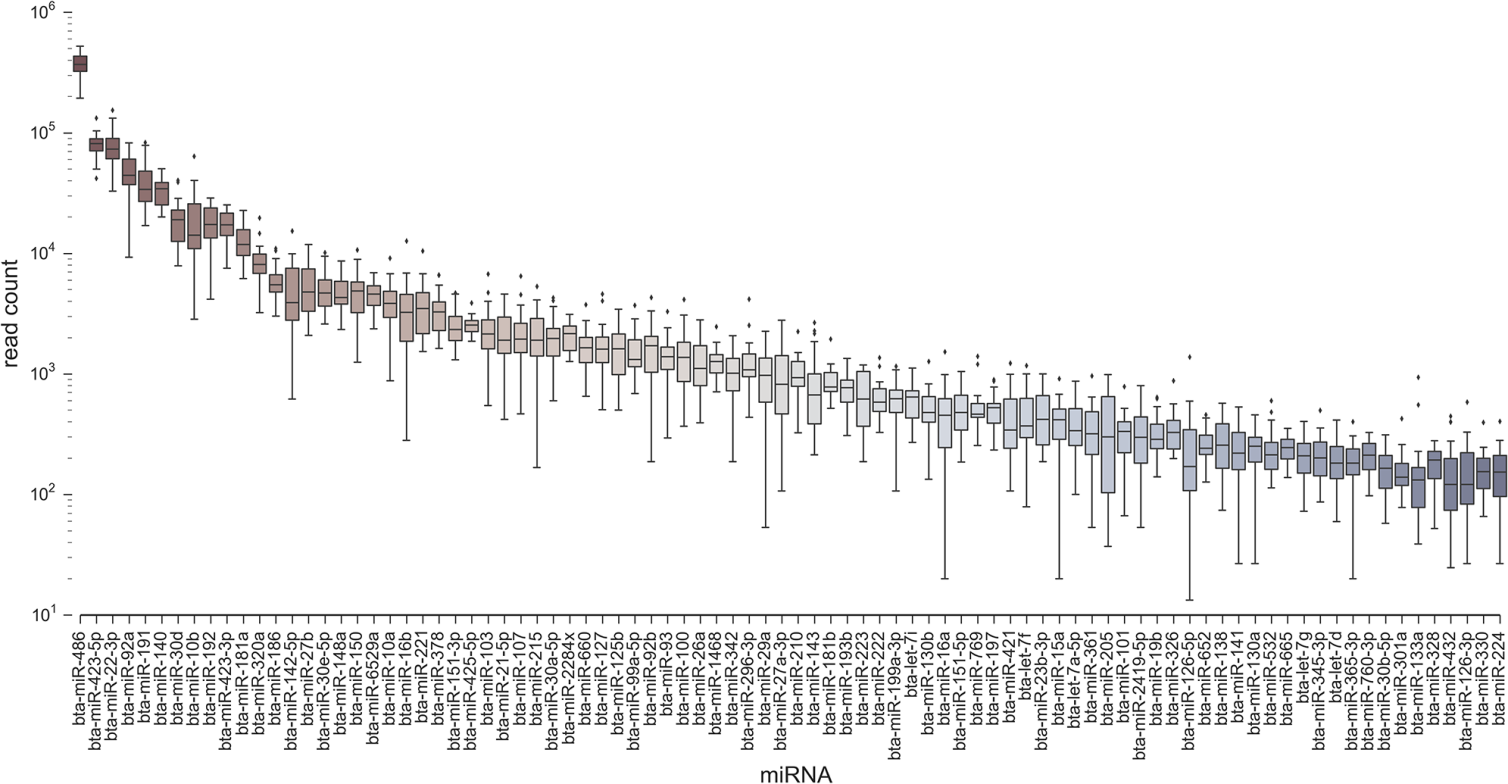
Read count distributions in all samples for top known miRNAs found by miRDeep2.

**Fig 4:**
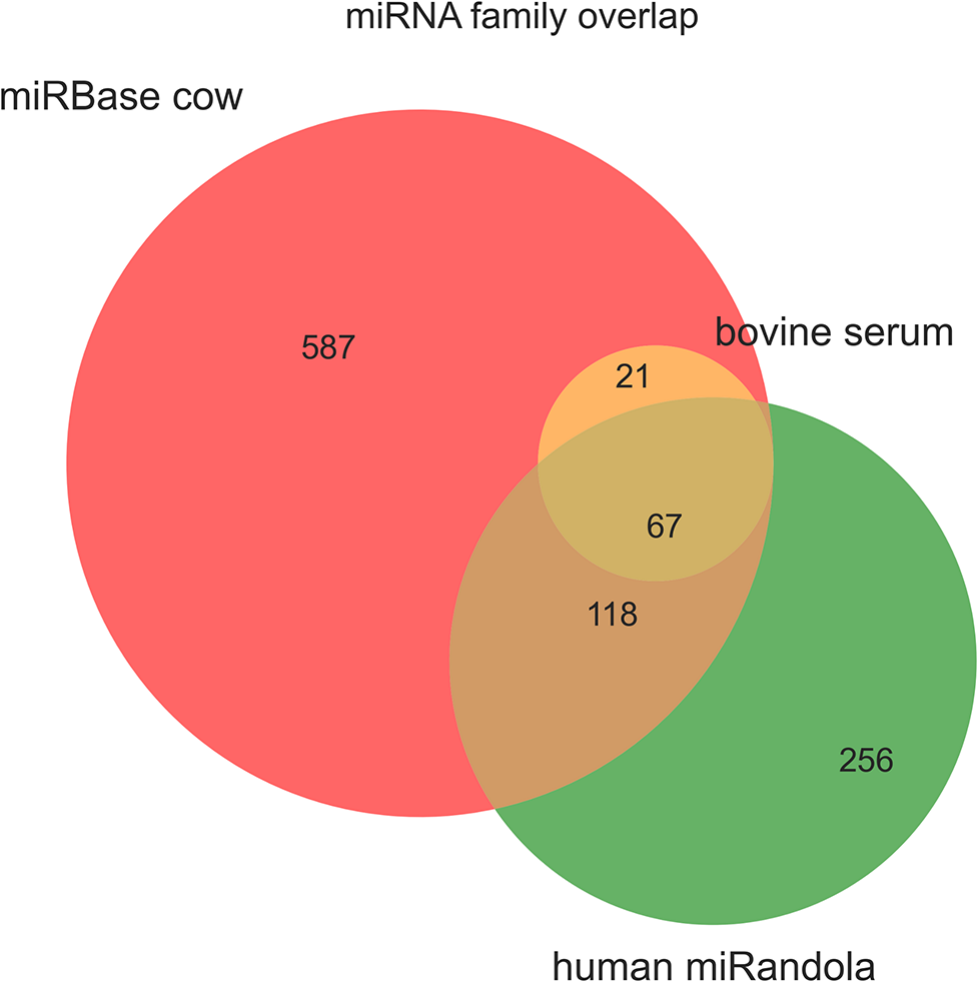
Overlap between core miRNAs in this study and known databases. The known bovine miRnome from miRBase 1.2 is in red. The green set are currently annotated extracellular circulating miRNA human families from the miRandola database. The yellow set represents our core bovine miRNAs.

Applying the miRDeep2 algorithm to all 24 of our samples and applying the score thresholds and other filters (see Methods section) produced a set of core miRNAs. The core list consisted of 88 known miRNAs (Fig 3) of which the top ten most abundant accounted for 85% of the total. In order of decreasing abundance, the top five were miR-486, miR-423-5p, miR-92a, miR-22-3p and miR-191. Our core set thus represents only a fraction of the currently known total miRNA bovine repertoire in miRBase. The majority of our core set have homologs in other mammalian species. Only 13% were from the 3p strand, and there was a roughly linear relationship between abundance (log mean read count across all samples) and frequency (the total number of samples in which the miRNA is found), as shown in S2 Fig. That is, miRNAs at low abundance are observed less frequently, probably due to insufficient depth in a subset of samples. We also compared our core miRNAs to the known human plasma and serum co-fractionate microRNAs contained in the miRandola database [38]. As shown in Fig 4 there is a substantial overlap between the miRNA families with over 70% of our circulating miRNAs being present in the database though they are only a small subset of the total bovine repertoire. There were no significant patterns in global miRNA populations across the two time points.

### IsomiR analysis

Sequence variant (isomiR) profiles across all samples were obtained from sRNAbench. The results were filtered to remove all low abundance isomiRs (total reads<10 across all samples and present in less than 50% of samples) that could be attributable to sequencing or alignment artefacts. As shown in previous studies [39] for many miRNAs the dominant isomiR was different to the reference miRBase mature sequence. The dominant and reference sequences differed in 51% of cases. Table 1 shows the 20 most significant isomiRs. In some of these cases the reference sequence was not present at all. 99% of miRNAs presented isomiRs with the number of variants seen being highly dependent on the abundance (S5 Fig). Typically isomer length distribution was normal, most obviously when low abundance variants are included.

**Table 1:** Top 20 dominant isomiRs across all samples. Those not marked as 'exact' variants are different from the canonical miRBase sequence. Variants are named using the sRNAbench nomenclature.

name	top isomeric sequence	counts	total	percentage	isomirs	variant
bta-miR-486	TCCTGTACTGAGCTGCCCCGA	1339764	4497982	0.30	733	lv3p
bta-miR-22-3p	AAGCTGCCAGTTGAAGAACTGT	665789	866536	0.77	152	lv3p
bta-miR-423-5p	TGAGGGGCAGAGAGCGAGACTTT	409857	890481	0.46	300	exact
bta-miR-92a	TATTGCACTTGTCCCGGCCTGT	325252	582925	0.56	288	exact
bta-miR-191	CAACGGAATCCCAAAAGCAGCTG	324529	502395	0.65	186	exact
bta-miR-30d	TGTAAACATCCCCGACTGGAAGCT	131657	238102	0.55	138	exact
bta-miR-25	CATTGCACTTGTCTCGGTCTGA	117960	198545	0.59	104	exact
bta-miR-10b	TACCCTGTAGAACCGAATTTGT	113970	236010	0.48	146	lv3p
bta-miR-140	ACCACAGGGTAGAACCACGGAC	106449	383774	0.28	354	mv
bta-miR-192	CTGACCTATGAATTGACAGCC	64586	207690	0.31	191	lv3p
bta-miR-423-3p	AGCTCGGTCTGAGGCCCCTCAGT	55006	189431	0.29	184	lv5p
bta-miR-181a	AACATTCAACGCTGTCGGTGA	45981	144760	0.32	104	lv3p
bta-miR-148a	TCAGTGCACTACAGAACTTTGT	42423	60311	0.70	56	exact
bta-miR-6529a	GAGAGATCAGAGGCGCAGAGT	39309	50349	0.78	45	exact
bta-miR-30e-5p	TGTAAACATCCTTGACTGGAAGCT	38605	63314	0.61	65	exact
bta-miR-16b	TAGCAGCACGTAAATATTGGCG	38479	47914	0.80	26	mlv3p
bta-miR-186	CAAAGAATTCTCCTTTTGGGCT	31118	71685	0.43	60	exact
bta-miR-27b	TTCACAGTGGCTAAGTTCTG	29769	68708	0.43	46	lv3p
bta-miR-142-5p	CCCATAAAGTAGAAAGCACT	27060	67172	0.40	65	mv
bta-miR-375	TTTGTTCGTTCGGCTCGCGTGA	25503	52553	0.49	61	lv5p

**Fig 5:**
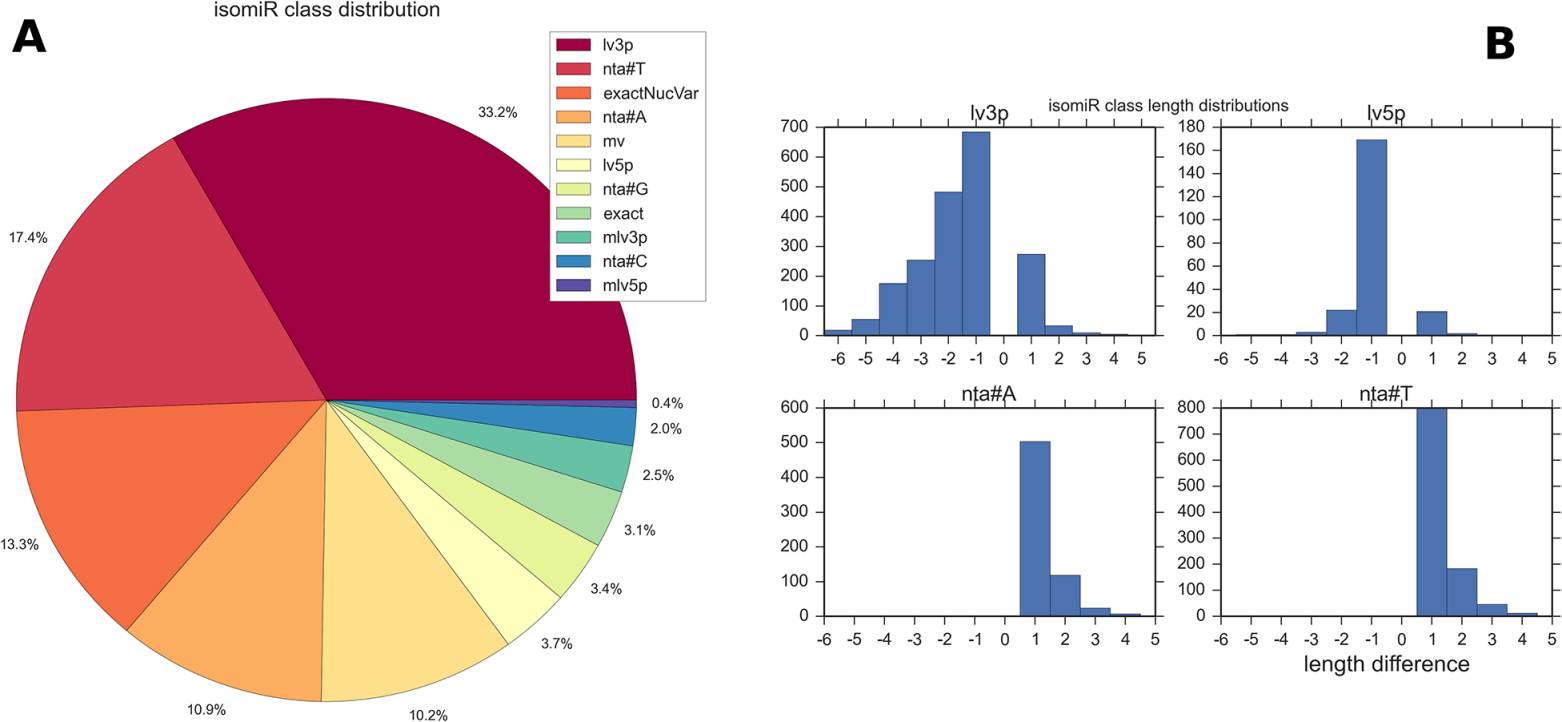
Isomir results. A: The relative frequencies of all isomiR classes including exact matches to the reference. nta#* denote non-templated additions, *lv3p* and *lv5p* the 5' and 3' length variants and *mv* denotes variants different at both ends. Classification is according to the sRNAbench hierarchical scheme. B: Length distributions for 5' and 3' variants showing the large variation in the latter. (x-axis shows length difference from canonical form). A number of the extreme 3' trimmed sequences are possibly due to erroneous mapping of short reads <18nt.

On average the dominant form accounted for 54% of all reads for a miRNA, but this varied greatly. Variant expression followed two main patterns as seen in other studies [40] those miRNAs with a strong predominant isomiR, such as bta-miR-191 and bta-miR-103; and those miRNAs where there is no predominant isomiR, such as bta-miR-486 and bta-miR-320a. The most common isomeric forms are 3' prime length variants, with the frequencies of all variant classes shown in Fig 5A using the sRNAbench nomenclature. Some 3' trimmed variants as much as 5 or 6 nt shorter than the reference were seen, as shown in Fig 5B, although most of these reflect low abundance forms of abundant miRNAs and may represent mismatches of short degradation products. However in one of these cases, bta-miR-23b-3p, an 18nt 3' length variant constitutes the dominant form with 58% of all reads. This highlights the importance of strict parameters in counting of isomeric variants and how such heterogeneity can affect the consistency and accuracy of abundance in certain cases.

### Novel miRNA discovery

After filtering-out low scoring and low frequency results, 30 putative novel miRNAs predicted by miRDeep2 were retained (Table 2). The majority of these are at low abundance levels but present consistently in all samples. Since the most likely candidates are those conserved across related species, we attempted to computationally screen our list by finding evidence of conservation across syntenic regions of other mammal genomes. The approach, (Methods section) uses the Ensembl Compara [34] genome wide comparisons to identify regions of synteny for each of our novel miRNA precursor coordinates and retrieved the aligned sequences for all available mammal species. The results could be grouped into three categories: 1) those conserved in at least five species, 2) those conserved only in ruminants (sheep and cattle) and 3) the remainder with no alignments due to lack of synteny with other species. Twelve were found to be contained inside a host protein coding gene, all of which were in introns with the exception of two found in exons. These proportions are consistent with the literature [41] and with our core subset of known miRNAs in which 64% are intergenic, 33% in host gene introns and just two in exons. None of the candidates were found to have targets in the 3' UTR regions of nearby or containing genes.

**Table 2:** Novel micro RNAs predicted in the data by mirDeep2 (after filtering steps). The 'gene' column denotes a host gene where found with transcription unit (intron or exon). The table is sorted by conservation in mammal species, that is, the number of species with aligned orthologs (aligned column). tr. unit = transcription unit. *max identity is the highest sequence identity to another species.

miRNA	max identity*	aligned	energy	tr. unit	biotype	gene	read count	miRDeep2 score	mirbase seed match	precursor coordinate
20_11724	1	11	-34.6	intron	protein coding	DROSHA	223	133.9	hsa-miR-635	20:42077865..42077925:+
22_13473	1	11	-22.2		miRNA	bta-mir-191	257	133.1	-	22:51543482..51543545:-
15_6321	0.952	10	-49.2				74	79.7	-	15:36372561..36372621:-
3_18437	0.992	10	-105.0		protein coding	SLC35D2	219	136.1	-	3:78601739..78601806:+
3_18843	0.863	9	-42.3				70	71.7	hsa-miR-7977	3:20198831..20198891:-
28_17041	1	9	-24.5				256	5.3	hsa-miR-1469	28:8597865..8597927:-
13_4632	0.99	8	-91.0	intron	protein coding	SNX5	1498	5.7	hsa-miR-4532	13:38543580..38543652:-
10_1684	0.941	7	-22.2				59	4.9	hsa-miR-423-5p	10:13002726..13002777:-
11_2549	0.984	7	-28.1	exon	protein coding	PTGS1	59	33.7	-	11:93244929..93244990:+
13_4213	0.763	5	-36.3	exon	protein coding	SRMS	253	160	-	13:54591461..54591519:+
28_17046	0.873	5	-30.1				3763	5.3	hsa-miR-4766-5p	28:9347067..9347134:-
18_8057	0.673	3	-19.6				177	4.2	hsa-miR-22-3p	18:1864501..1864552:+
9_24948	0.91	2	-18.3				192	5.1	hsa-miR-142-5p	9:86842891..86842958:+
6_21896	0.966	2	-10.3	intron	protein coding	PDCL2	235	120.6	-	6:72695823..72695882:+
5_20820	0.968	2	-28.1	intron	protein coding	ABCC9	71	5.2	hsa-miR-6747-5p	5:88730950..88731013:+
4_20321	1	2	-16.4				186	4.8	hsa-miR-6827-5p	4:115125346..115125387:-
3_18517	0.966	2	-21.6	intron	protein coding	ORC1	1771	1374.1	-	3:94548590..94548649:+
10_1480	0.948	2	-25.1	intron	protein coding	DAAM1	193	38.7	-	10:71809966..71810024:+
3_18032	0.965	2	-31.8				54	5.6	hsa-miR-561-3p	3:2852303..2852360:+
26_16197	0.763	2	-25.9	intron	protein coding	DMBT1	1572	887.3	-	26:42790257..42790316:+
26_16101	0.915	2	-19.6				143	99	hsa-miR-548at-3p	26:26545665..26545724:+
9_25151	0.957	2	-22.9				7363	4.2	hsa-miR-6873-5p	9:27844229..27844299:-
29_18010				intron	protein coding	BRSK2	152	5.9	hsa-let-7i-3p	29:51095161..51095211:-
4_20156				intron	protein coding	FAM3C	197	102.5	-	4:86669291..86669351:-
24_14535			-19.8				227	198.4	-	24:6436128..6436188:+
9_24602				exon	misc_RNA	Metazoa_SRP	491	5.5	hsa-miR-6829-5p	9:2105903..2105987:+
1_987			-23.0				51	4.6	hsa-miR-6890-3p	1:135841882..135841950:-
4_19879							2175	5.1	hsa-miR-515-5p	4:15729..15810:-
25_15966							173	6.2	hsa-miR-132-5p	25:42673013..42673075:-
3_18831							141	95.2	hsa-miR-637	3:19127469..19127551:-

Of the putative novel miRNAs, several had interesting features and are detailed here. 20_11724 is contained inside the intronic region of the Drosha gene in all species and is >95% conserved in all species. This sequence is annotated as a Gencode predicted human novel miRNA (transcript ID ENSCSAT00000020128) but has not been observed experimentally before. 22_13473 was 99% conserved in all species and was found to be reverse complementary to the bta-miR-191 gene. 26_16197 is present in the *DMBT1* gene along with three other novel miRNAs predicted by miRDeep2 but which did not meet the filtering criteria; these form a possible cluster of up to 4 miRNAs inside this gene. None of these miRNAs have so far been annotated in miRBase for any species. After filtering we only saw one probable false positive in the final list. This was 9_24602, found inside an existing predicted ncRNA Metazoan signal (Ensembl ID ENSBTAG00000048185). Further inspection of the read mappings indicated this may not be a real miRNA because of the inconsistent alignment of reads across the hairpin sequence.

**Fig 6:**
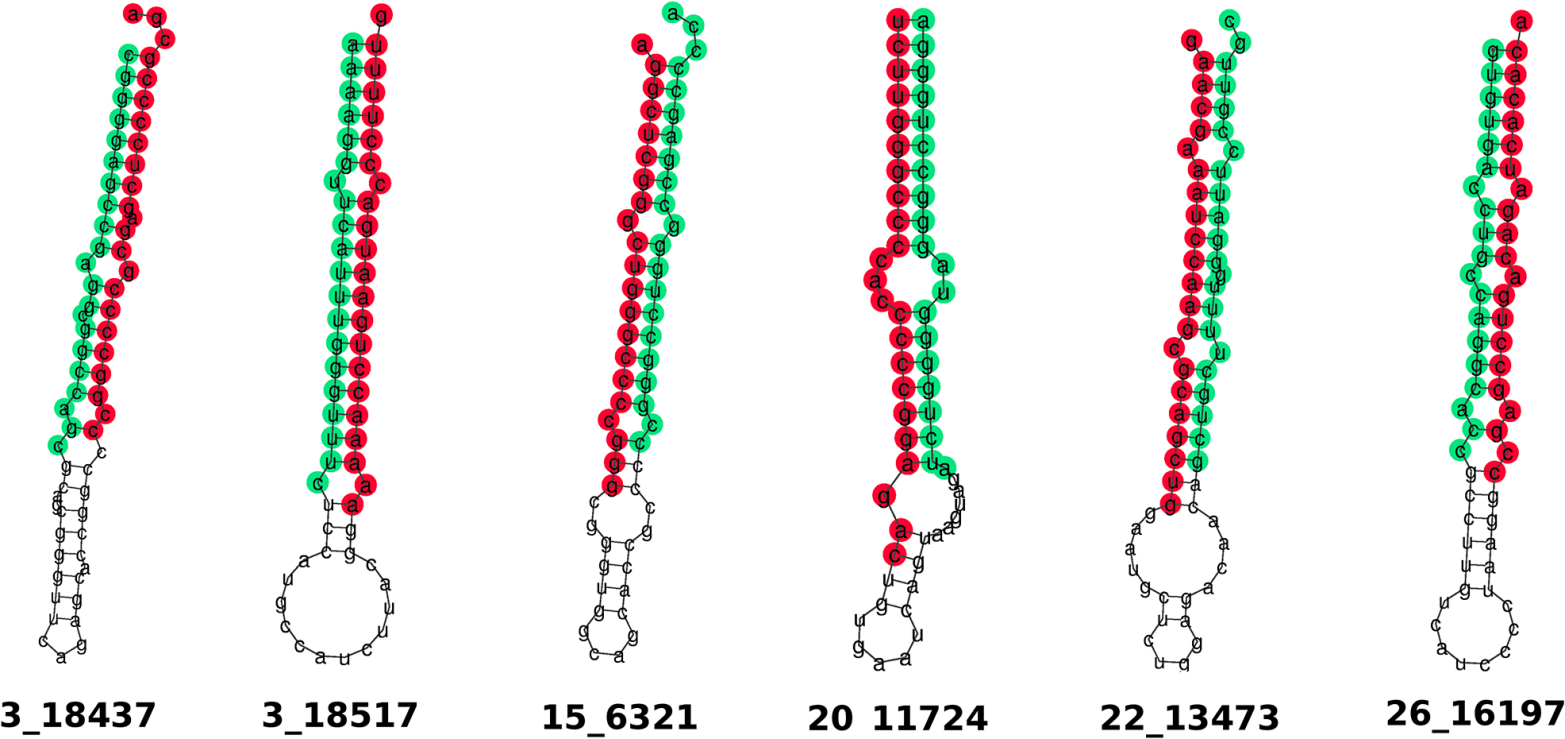
Selected novel miRNAs. Predicted RNA secondary structure of the hairpin for novel micro RNAs. Sequence is coloured according to mature (red) and star (green). Sequences are consensus derived by miRDeep2.

The majority of candidates contained a seed that already exists in a known human miRNA but with the remainder of each sequence not well conserved. Some of the non-conserved hits are likely to be bovine specific. The predicted structures for the six top hits are shown in Fig 6. A csv file containing the aligned sequence, genomic coordinate, sequence identity and folding energy for all miRNAs in each species that could be aligned is given in S3 Table. Read alignments in all samples and scores breakdown from miRDeep2 are given in for all novel miRNAs are given in S1 File.

### Differential miRNA expression

Both within group and between group comparisons were used to investigate differential miRNA expression. Stringency was ensured by using a combined FDR of <0.05 and a fold-change of 1.5 as cut-offs for significance. Examining differences between the 0 and 6 month time-point post-MAP infection identified two significantly differentially expressed miRNAs. Specifically at the latter interval, miR-205 was increased (2-fold) while miR-432 was decreased (2-fold). Control animals, however, also displayed similar miR-205 increases (2-fold) and miR-432 decreases (2-fold) at the 6 month time-point but were also accompanied by subtle increases (~1.5 fold) in expression for miR-27a, miR-92b, miR-10b, miR-143 and miR-126-5p (Table 3). Overall, these within group differences appear to be an ageing effect that is independent of infection status as direct comparisons between the infected and control cattle at the six month interval revealed no significantly differentially expressed miRNA.

**Table 3:** Differentially expressed miRNA identified by comparing time-point 0 to time-point 6 months within each group (MAP-infected versus controls).

Gene	Log_2_ Fold Change	P value	FDR
**Infected–TP0 vs 6 months**			
bta-miR-205	2.14	1.61×10^−13^	1.39×10^−11^
bta-miR-432	-2.23	1.50×10^−12^	6.45×10^−11^
**Control–TP0 vs 6 months**			
bta-miR-205	2.23	1.54×10^−6^	9.18×10^−5^
bta-miR-126-5p	1.67	0.000202	0.002174
bta-miR-143	1.59	1.45×10^−5^	0.000389
bta-miR-27a-3p	1.55	1.81×10^−5^	0.000389
bta-miR-92b	1.54	8.55×10^−5^	0.001050
bta-miR-10b	1.51	3.49×10^−5^	0.000500
bta-miR-127	-1.78	2.45×10^−5^	0.000422
bta-miR-432	-2.07	2.14×10^−6^	9.18×10^−5^

**Fig 7:**
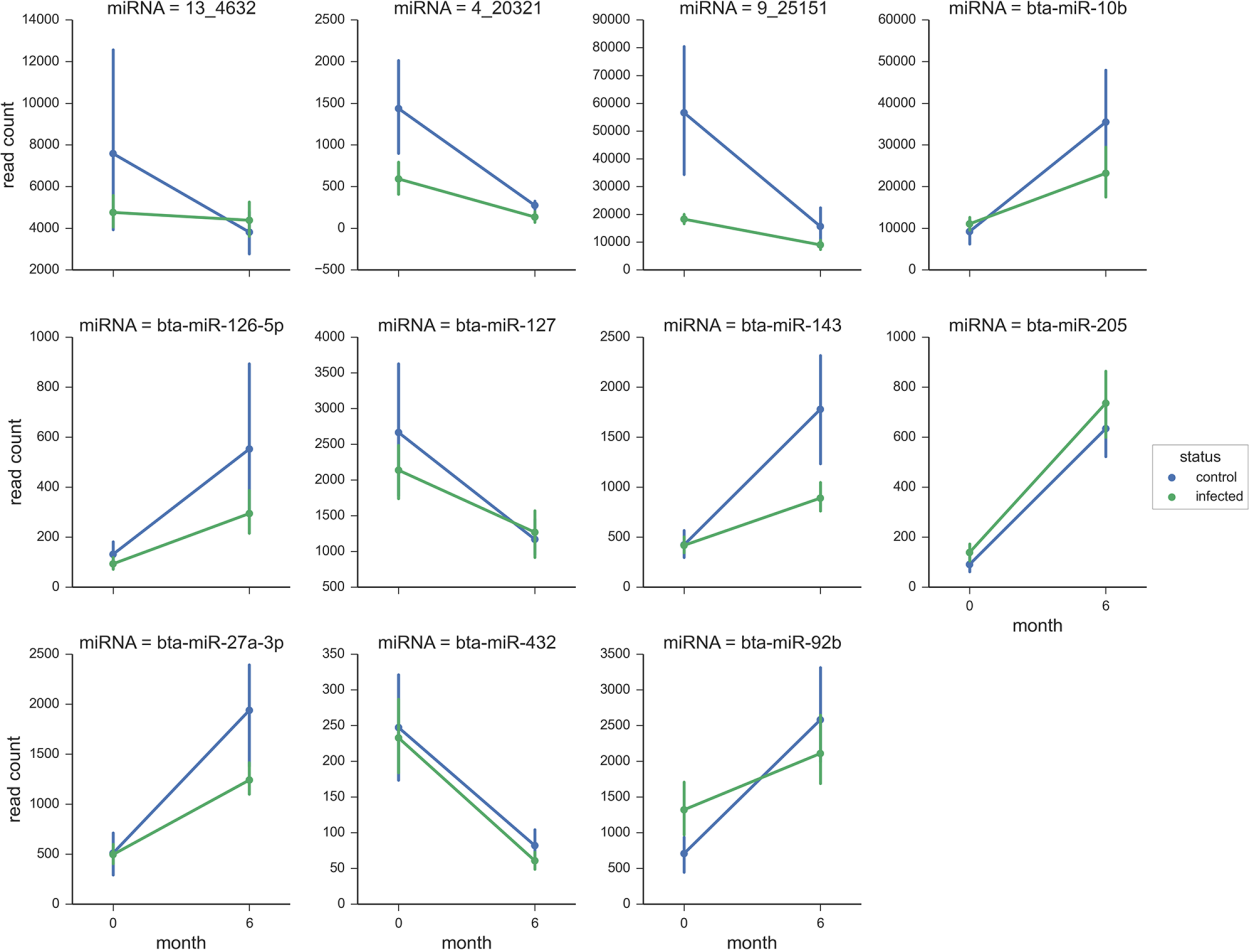
Differential expression results. Normalised read variation for differentially abundant genes from 0–6 months in both control (blue) and infected (green) groups. The bars on each point represent the within group variation for 6 animals (95% confidence interval).

Subsequently for each group, the reads counts of the eight differentially expressed miRNAs identified during the above pairwise comparisons (miR-10b, miR-126-5p, miR-127, miR-143, miR-205, miR-27a-3p, miR-432 and miR-92b) were plotted to gain further insight into their expression profiles (Fig 7). For all the miRNAs, there were similar expression patterns for the infected and control cattle, but the read counts were somewhat variable at each time-point (Fig 7).

## Discussion

Small RNA sequencing is currently the method of choice for identification of the entire repertoire of known and novel miRNAs in a given tissue type or specific disease state [42]. There are however relatively few papers on the application of this technology to circulating miRNA in sera, largely due to very low total RNA concentrations in biofluids. Nevertheless, it has recently been shown that investigation of differential miRNA expression can be achieved with as little as 1 ml of serum [43]. We show here that it is possible to describe circulating miRNA repertoires in bovine serum from low serum volumes, opening up the application of this approach to bovine sera samples and existing biobanks.

It has been reported that large percentages of small RNA reads derived from mammalian sera typically either align to non-miRNA sequences or else cannot be reliably mapped [44–46]. In agreement with these observations, only approximately 5% of our reads mapped to known miRNAs. Notably, a high abundance of reads in each sample corresponded to fragmented tRNAs (25–35 nt), likely arising due to degradation processes that occur within the circulation or as a result of the clotting process during sera preparation. This is, however, not exclusively a serum problem, as Metpally *et al*. also reported a high percentage of fresh human plasma reads mapping to non-miRNA categories [47]. An obvious concern with low numbers of miRNA-specific reads is whether adequate sequencing depth has been used, and we therefore explored this in our data. By gradually increasing the reads analysed, it was obvious that the numbers of miRNAs identified proportionally increased. However, miRNA discovery appeared to reach a saturation point at approximately 3.5 million reads, a depth above which all our samples had been sequenced at (ranging from 12–29 million reads). While the sequencing depth used may lack the sensitivity to detect rare lowly expressed miRNAs, our depth of coverage was sufficient for the robust identification of potential biomarkers.

We identified approximately 80–100 functionally relevant known miRNAs per sample, with the majority being orthologs of known human circulating miRNAs [48,49]. The top 10 miRNAs accounted for 86% of the total, and miR-486 was the most abundant miRNA across all samples. Interestingly, it has been reported that the IlluminaTruSeq Small RNA Sample Preparation Kit enriches hsa-miR-486 in human plasma libraries yields 50-fold more RNA than other library preparation methods [50], suggesting that some quantitative biases are likely to be apparent within our data. This, however, should not have major implications for differential expression analyses.

Extensive isoform diversity for the majority of miRNAs was shown. Undoubtedly some of this variation is due to technical artefacts such as sequence mismatches of very short reads. However after low abundance filtering, multiple variants are still seen consistently across all samples. These consistent patterns of variant expression provide confidence that such variants are not simply due to degradation products [51]. Distribution of isomiRs from specific tissues is now thought to be non-random and of biological relevance. In serum samples, the picture may be even more complex as multiple subgroups of dominant isomiRs reflecting different tissues may be present. Thus the ubiquity of isomiRs is a potential confounding factor that must be considered in biomarker assays, and even more so for miRNA from biofluids such as blood. The presence of isomiRs could have profound effects on qPCR detection of a miRNA in which several sub-dominant isoforms are actually present that differ sufficiently from the canonical form to cause misdetections.

Various technical factors affect the outcome of miRNA-seq data analyses and it is not always obvious which algorithm is best suited to handling the data; for this reason we chose two approaches, namely sRNAbench and miRDeep2. For instance, sRNAbench was found to be more convenient for isomiR reporting. For several of the lower abundance known miRNAs we found large differences in total read count between sRNAbench and miRDeep2. However, both methods are however highly configurable (for example, the flanking regions within which reads are assigned to a precursor may be varied) and the configuration of multiple profiling parameters could account for significant differences between the two programs.

Tool selection will also have a significant impact on novel miRNA discovery. miRDeep2 was preferred for novel prediction because 1) its scoring model allows transparent screening of false positives and 2) it is designed to be applied to all samples in one run, thus independent samples are easily integrated for the whole study. We found sRNAbench somewhat more cumbersome in this regard. We note that miRDeep2 erroneously missed counting of bta-mir-1246 in the known list and assigned this as a novel miRNA; the reason for this is unclear. Therefore, care must be taken in parsing and filtering the novel candidates provided. However in general miRDeep2 proved to be a powerful ‘off the shelf’ tool for novel discovery once additional filtering was applied. Regardless of the data, it is strongly advised to use at least two tools for initial analysis both for consistency checking and since particular features of one tool may be more appropriate to the data in question [52].

While the cancer and cardiovascular fields already have a significant body of literature dedicated to the potential of miRNAs as biomarkers, to date there have been relatively few published investigations of circulating miRNA biomarkers applied to infectious bacterial diseases. A few notable exceptions have explored the potential of miRNAs in tuberculosis cases [17–21,53] and as biomarkers of pathology in bovine TB [54]. qPCR-based assays have been the preferred technological approach to identify potential candidates in serum [18,19], but recently miRNA-seq has increased in popularity [20,21] due to the ability to conduct differential expression analysis across the entire “miRNome” of the biofluid.

The lack of robust diagnostic and prognostic assays for MAP infection and resulting disease severely hampers the control and eradication of this infection in cattle. Circulating miRNAs hold promise as a novel diagnostic modality for the disclosure of MAP infection status. The presence of miRNA in peripheral blood is thought to arise due to a number of distinct mechanisms; for example, activated lymphocytes are known to release exosomes containing specific miRNAs [55], while a range of tissue pathologies have distinct circulating miRNA profiles, from fatty liver disease [56] and intracranial aneurysms [57] to prostate cancer [58] and Crohn’s Disease [59]. In this study, we therefore examined MAP-challenged calves displaying early signs of cell-mediated immune responses to MAP, as indicated by IFN-**γ** responses in infected animals. While calves infected with MAP for only six months would not be expected to show pathology in the gastrointestinal tract, we hypothesised that the cellular responses to infection detected in peripheral blood may give rise to a miRNA profile that would be detectible in serum. A comparison of the serum miRNome of MAP-challenged IFN-**γ** responders to their age-matched unchallenged controls six months after infection did not identify a significant difference in miRNA expression. This lack of differential miRNA signature in infected animals is likely due to the insidious nature of the infection; at the time-points examined the animals would still be within the silent stage. Future analyses will focus on characterising serum miRNA profiles from cattle that are at a more advanced stage of infection, having developed a serological response to MAP indicative of a T_H_1 to T_H_2 switch and that have commenced faecal shedding.

Longitudinal differences in miRNA expression were, however, evident and similar within both the challenged and control groups. Comparing the 0 to the six month time-points within each group revealed similar read count profiles for miR-205, miR-10b, miR-92b, miR-432, miR-27a, miR-127, miR-126 and miR-143. All these miRNAs were significantly differentially expressed within the control group but, only miR-205 and miR-432 were identified as significant in the MAP-infected group. These changes in the circulating miRNA profile are likely the result of developmental processes in the calves as all of these miRNAs have been shown to have cellular proliferation and development roles in various mammals [60–67]. Furthermore, in humans, it has been reported that the serum miRNA expression signature (profile) changes with age [68]. Thus, our findings also highlight the critical importance of using age-matched controls in such studies.

In summary we have used deep sequencing of small RNA fractions isolated from bovine serum to explore the utility of circulating miRNAs as potential biomarkers of infection and disease progression in MAP-infection. We have revealed a number of novel circulating miRNA in bovine serum, and identified miRNA whose abundance is linked to bovine development. While no robust miRNA signature of infection could be defined, this may be due to the silent nature of MAP infection at the early time point selected; future studies focussed on animals in the subclinical and clinical phases of infection may prove more revealing.

## Supporting Information

S1 Fig Small RNAs per sample. Proportional mapping by Bowtie to each category of small RNA for each sample in the study. Bars are grouped by pool but not otherwise ordered.
(TIF)

S2 Fig Read count vs. sample frequency. Relationship between log normalised mean normalized read count and sample frequency (number of samples in which each miRNA is found). The majority of high abundance hits are found in over 80% of samples.
(TIF)

S3 Fig Read length distribution for a representative sample after adapter trimming. The large numbers of reads>30 represent tRNA degradation product and the small peaks around 20 reads represent the miRNA content. Reads <18nt were removed for later miRNA analysis.
(TIF)

S4 Fig Read count threshold estimation. Thresholds where the read count distribution of a pair biological replicates become similar can be estimated using the Komologorov-Smirnov statistic as a distance measure. This point can be defined as the initial minimum as increasing read count values are removed from each distribution. For our data these values ranged from 50–150 depending on samples. Data shown for here is for three pairs of replicates.
(TIF)

S5 Fig IsomiR copy number per miRNA versus log total abundance. Unique log IsomiR copy number per miRNA versus log total abundance across all samples shows a linear relationship.
(TIF)

S6 Fig miRDeep2 dependency of signal to noise and false discovery rate on score. (TIF)

S7 Fig Read count distributions in all samples for novel miRNAs found by miRDeep2. (TIF)

S1 File PDF files generated by miRDeep2 for all miRNAs discovered in this study. (ZIP)

S1 Table Known miRNAs specified by miRDeep2 that meet the filtering criteria described in the paper. (CSV)

S2 Table Novel miRNA data (miRDeep2). (CSV)

S3 Table Orthologs and sequences of all conserved miRNA in each species found using the Ensembl conservation analysis. (CSV)
