# Supplementary figures and images for: The Identification of Circulating MiRNA in Bovine Serum and Their Potential as Novel Biomarkers of Early Mycobacterium avium subsp paratuberculosis Infection
Source: PLoS One. 2015 Jul 28;10(7):e0134310. doi: 10.1371/journal.pone.0134310 (PMC4517789; doi:10.1371/journal.pone.0134310)

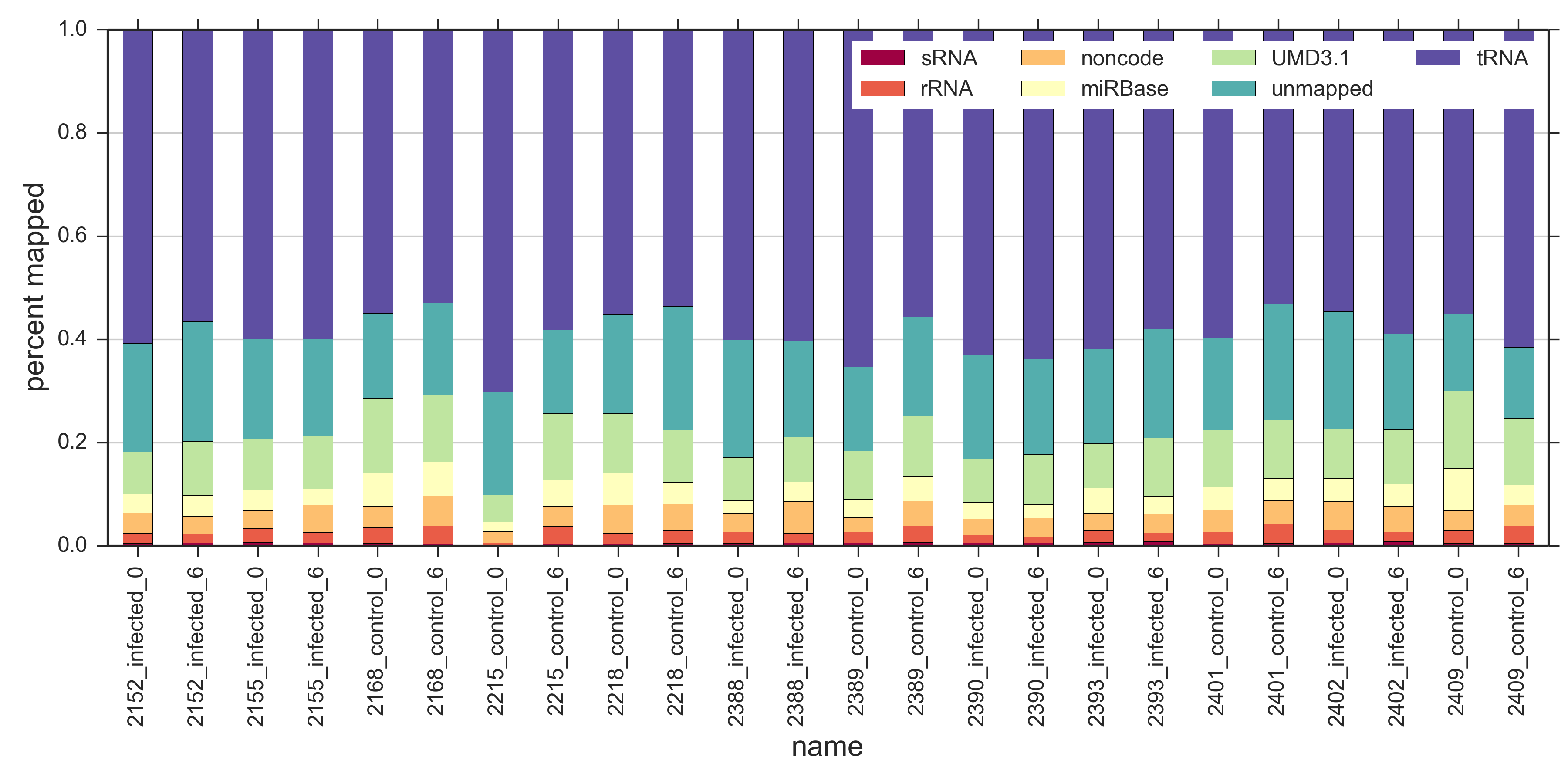

Supplement: S1 Fig — Proportional mapping by Bowtie to each category of small RNA for each sample in the study. Bars are grouped by pool but not otherwise ordered. (TIF) [file pone.0134310.s001.tif]

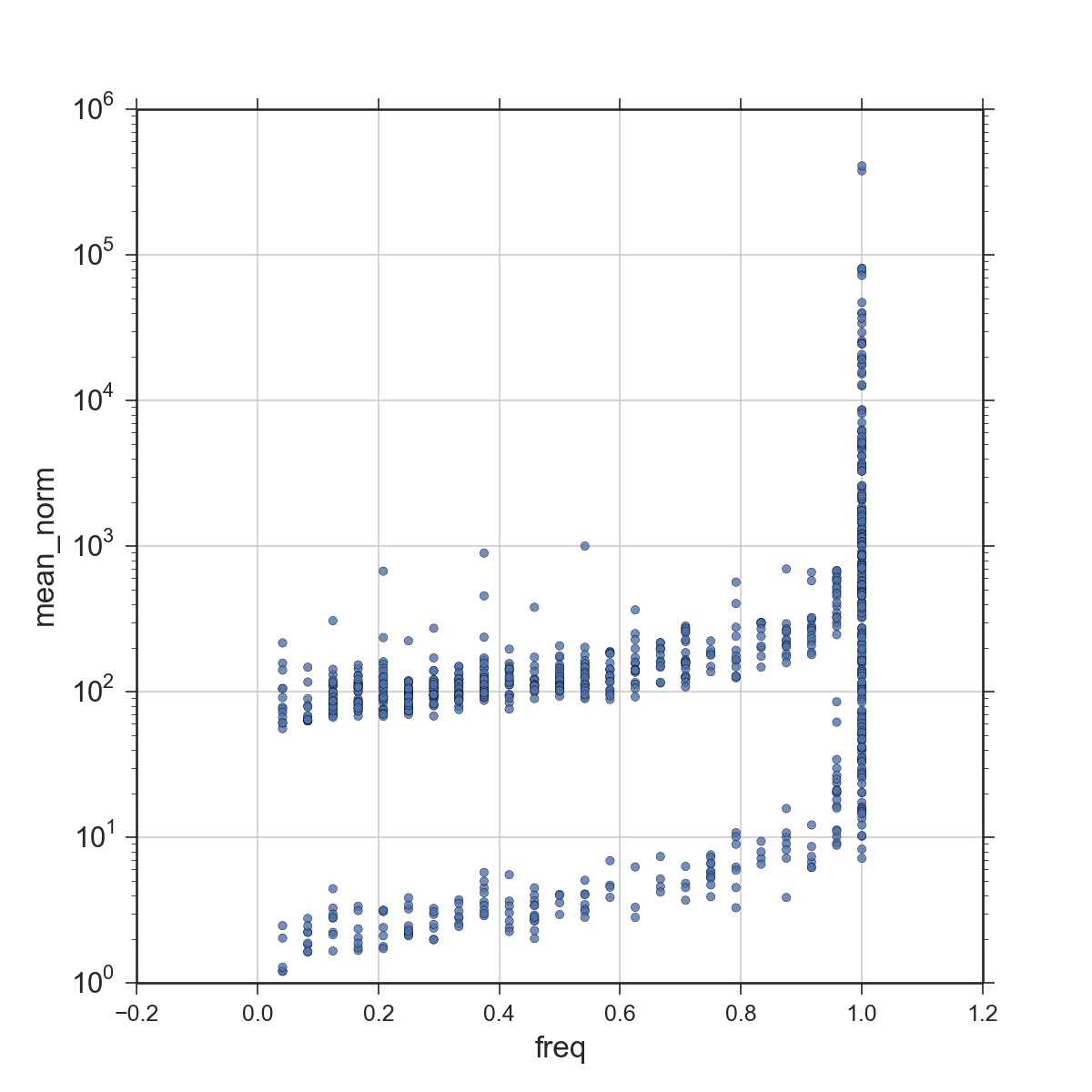

Supplement: S2 Fig — Relationship between log normalised mean normalized read count and sample frequency (number of samples in which each miRNA is found). The majority of high abundance hits are found in over 80% of samples. (TIF) [file pone.0134310.s002.tif]

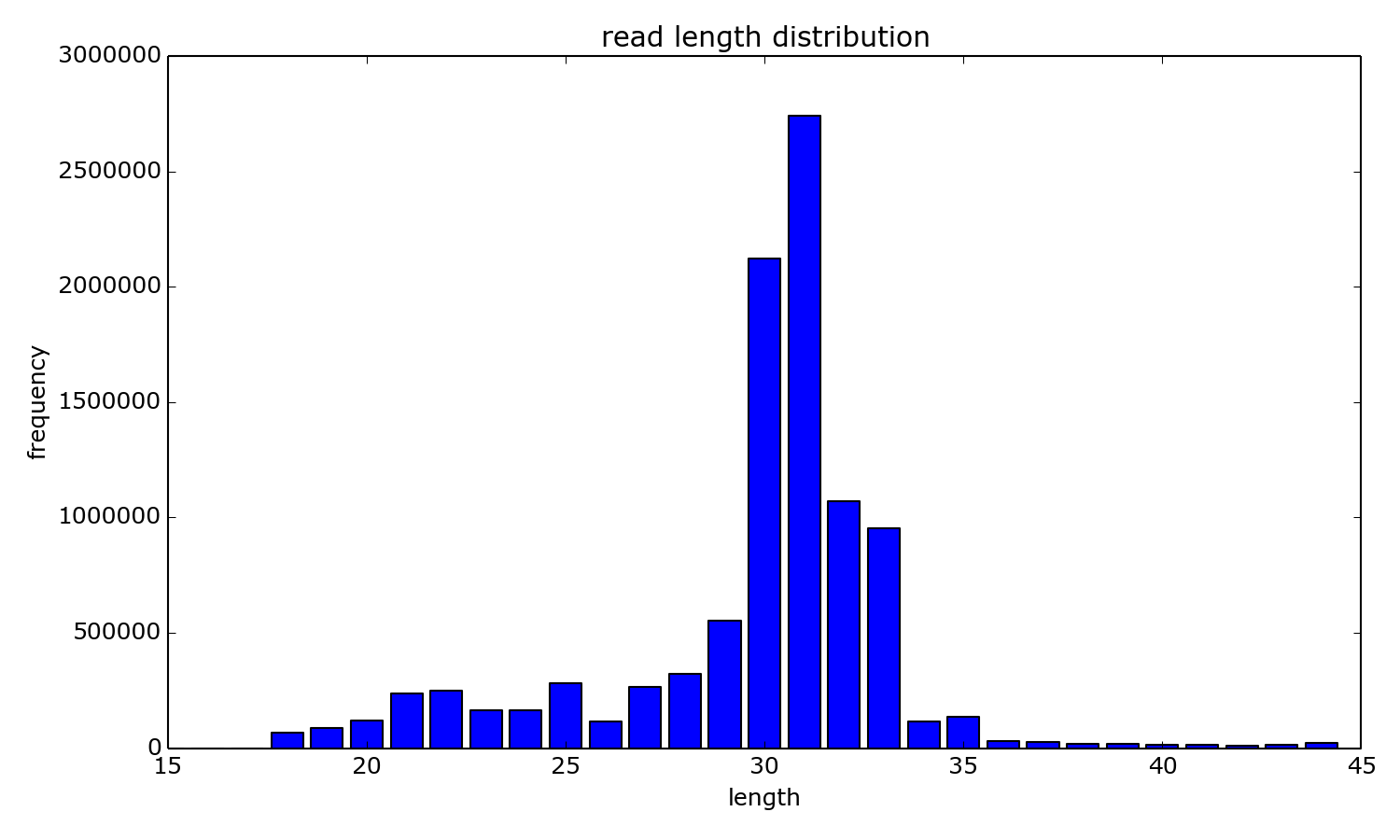

Supplement: S3 Fig — The large numbers of reads>30 represent tRNA degradation product and the small peaks around 20 reads represent the miRNA content. Reads <18nt were removed for later miRNA analysis. (TIF) [file pone.0134310.s003.tif]

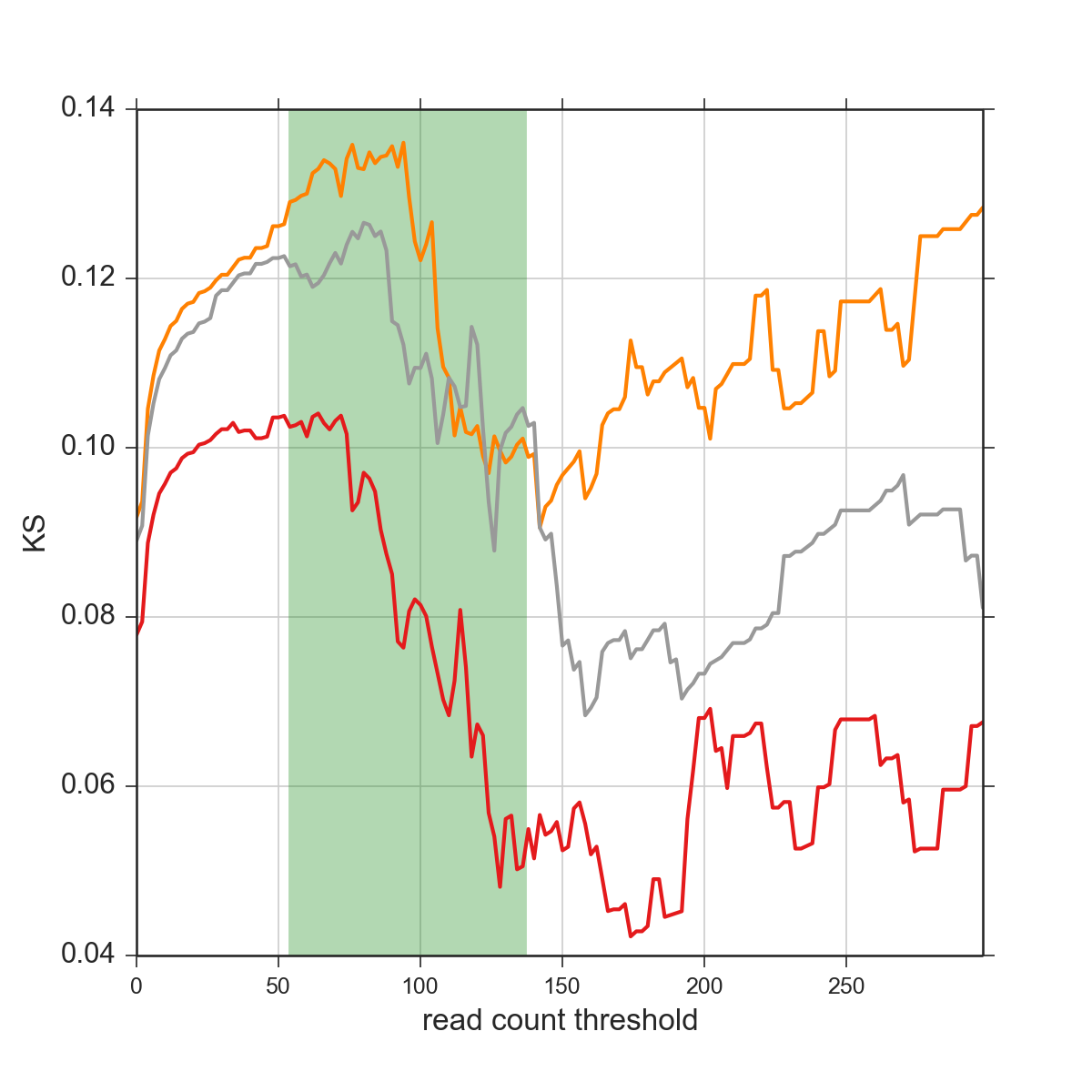

Supplement: S4 Fig — Thresholds where the read count distribution of a pair biological replicates become similar can be estimated using the Komologorov-Smirnov statistic as a distance measure. This point can be defined as the initial minimum as increasing read count values are removed from each distribution. For our data these values ranged from 50–150 depending on samples. Data shown for here is for three pairs of replicates. (TIF) [file pone.0134310.s004.tif]

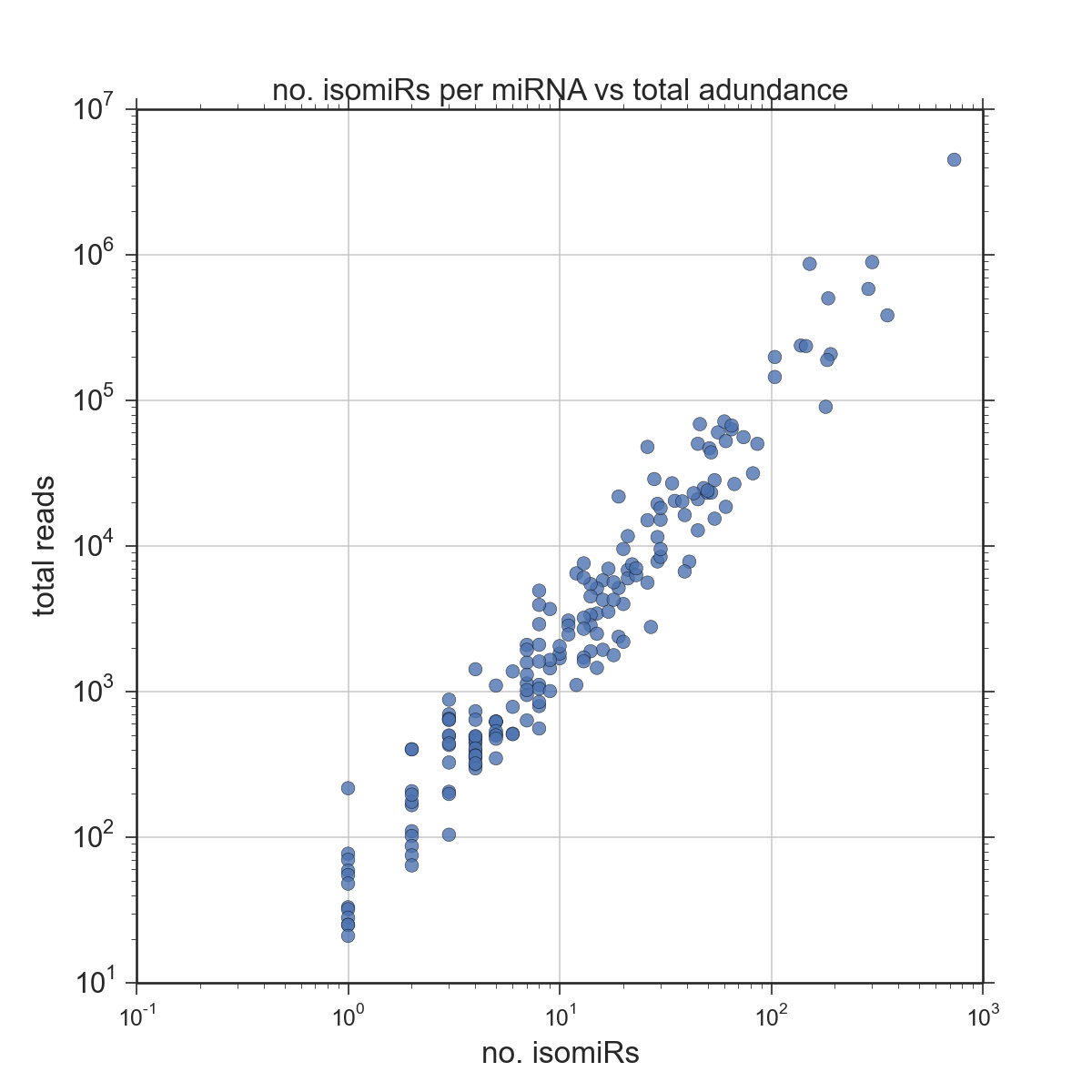

Supplement: S5 Fig — Unique log IsomiR copy number per miRNA versus log total abundance across all samples shows a linear relationship. (TIF) [file pone.0134310.s005.tif]

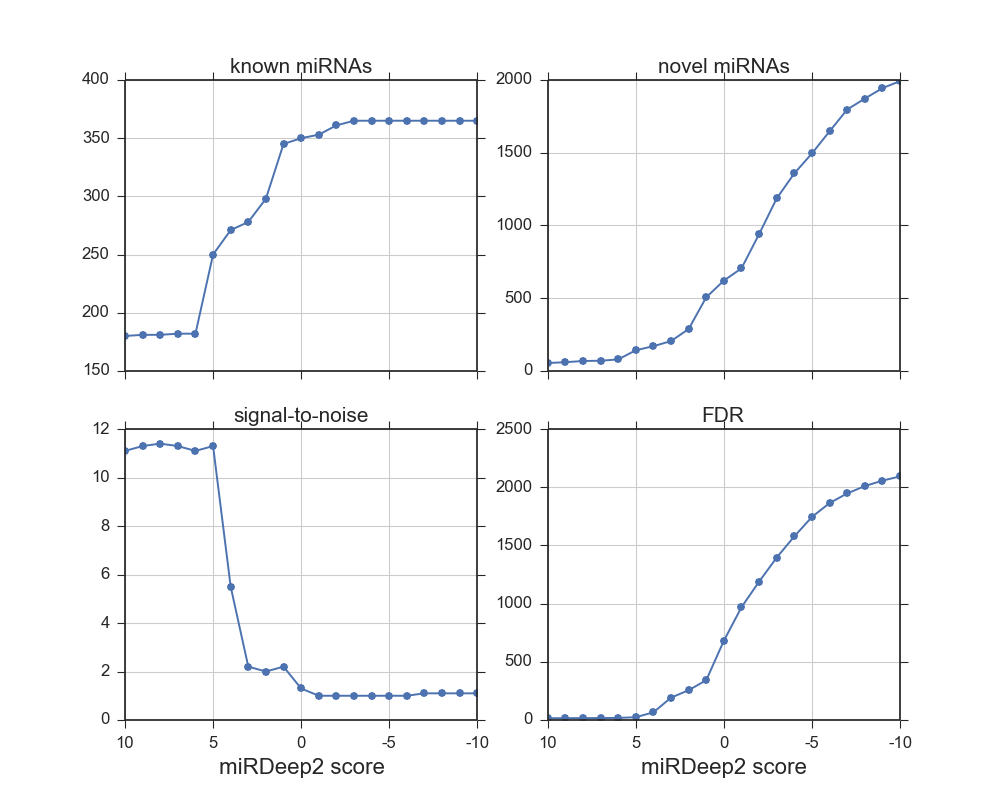

Supplement: S6 Fig — (TIF) [file pone.0134310.s006.tif]

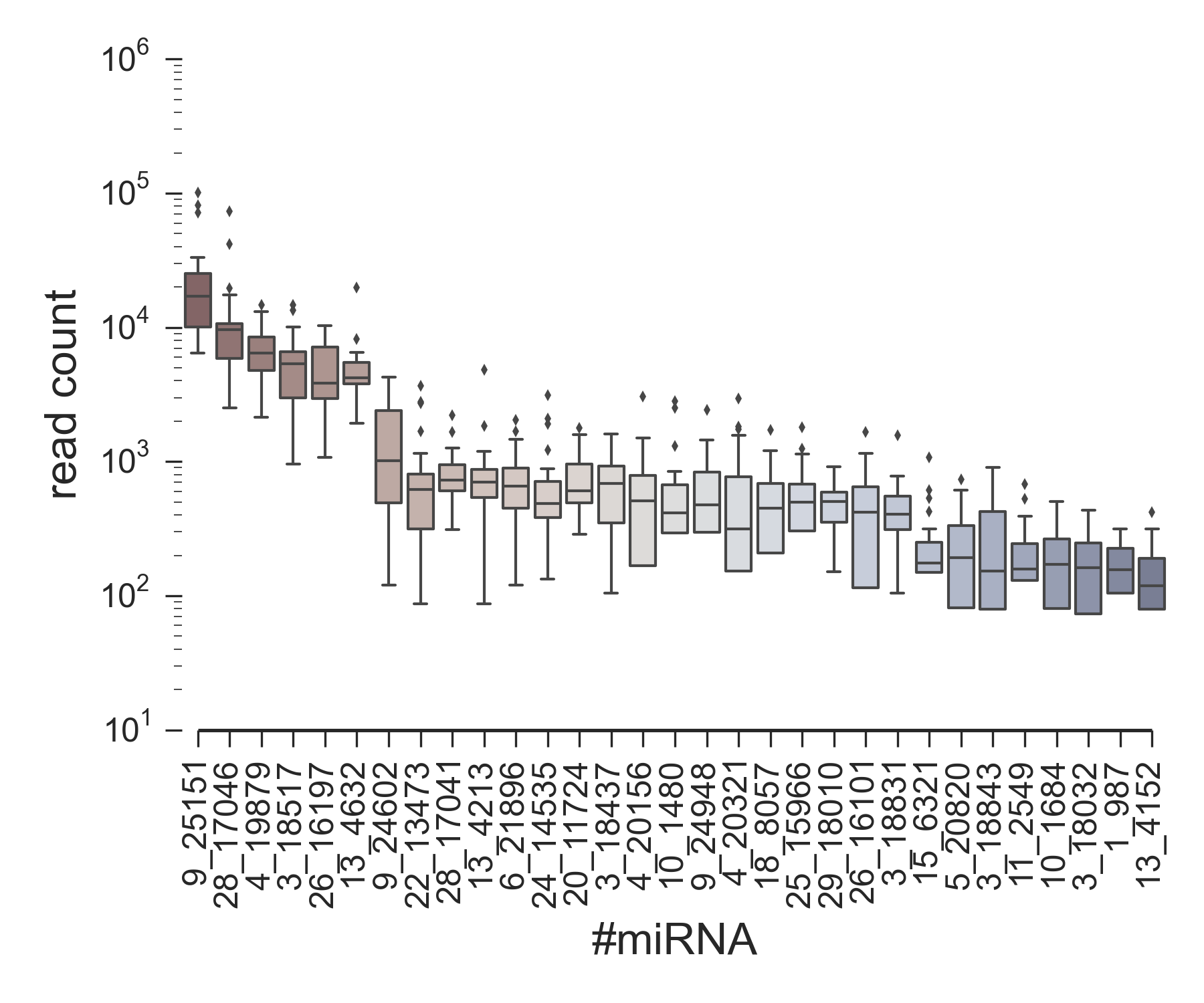

Supplement: S7 Fig — (TIF) [file pone.0134310.s007.tif]

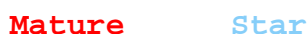[illegible]

Supplement: S1 File — (ZIP) [file pone.0134310.s008.zip › novel_pdfs/4_20321.pdf]
