# Supplementary material for: The Identification of Circulating MiRNA in Bovine Serum and Their Potential as Novel Biomarkers of Early Mycobacterium avium subsp paratuberculosis Infection
Source: PLoS One. 2015 Jul 28;10(7):e0134310. doi: 10.1371/journal.pone.0134310 (PMC4517789; doi:10.1371/journal.pone.0134310)
Supplement: S1 File — (ZIP) [file pone.0134310.s008.zip › novel_pdfs/13_4152.pdf]

Provisional ID	:	13_4152
Score total	:	4.8
Score for star read(s)	:	-1.3
Score for read counts	:	-0.3
Score for mfe	:	1.8
Score for randfold	:	1.6
Score for cons. seed	:	3
Total read count	:	11
Mature read count	:	11
Loop read count	:	0
Star read count	:	0

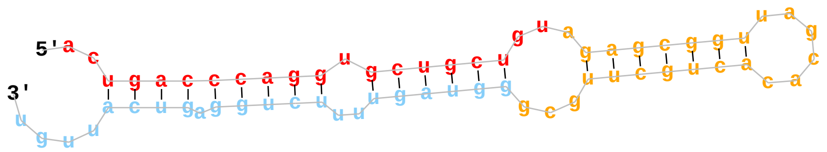

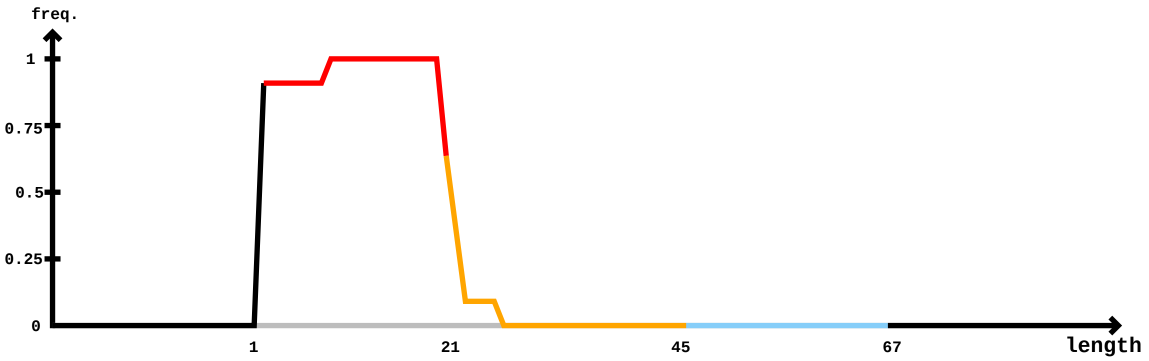

Mature Star

```
5'- ugcagcgcuugagccauggcacugacccaggugcugcuguagagcgguuagcacacugcuugcggguaguuuucuggagucauuguucccuccuugugcaguagcugcu   -3'    exp
    .((((((((...((((.((((.(((((((((.((((((...(((((((......)))))))...))))))..))))).)))).))......)).)).))))).))))).    reads    mm    sample
    ....................acugacccaggugcugcugG.....................................................................    1        1     s07

    ...........................caggugcugcugCagagc................................................................    1        1     s14

    ....................acugacccaggugcugcugG.....................................................................    1        1     s10

    ....................acAgacccaggugcugcug......................................................................    1        1     s18

    ....................acugacccaggugcugcugG.....................................................................    1        1     s17

    ....................acugacccaggugcugcug......................................................................    1        0     s06

    ....................acugacccaggugcugcugG.....................................................................    2        1     s05

    ....................acugacccaggugcugcugG.....................................................................    1        1     s16

    ....................acCgacccaggugcugcug......................................................................    1        1     s04

    ....................acugacccaggugcugcug......................................................................    1        0     s11
```
