# Supplementary material for: The Identification of Circulating MiRNA in Bovine Serum and Their Potential as Novel Biomarkers of Early Mycobacterium avium subsp paratuberculosis Infection
Source: PLoS One. 2015 Jul 28;10(7):e0134310. doi: 10.1371/journal.pone.0134310 (PMC4517789; doi:10.1371/journal.pone.0134310)
Supplement: S1 File — (ZIP) [file pone.0134310.s008.zip › novel_pdfs/1_987.pdf]

Provisional ID	:	1_987
Score total	:	4.6
Score for star read(s)	:	-1.3
Score for read counts	:	0
Score for mfe	:	1.3
Score for randfold	:	1.6
Score for cons. seed	:	3
Total read count	:	40
Mature read count	:	40
Loop read count	:	0
Star read count	:	0

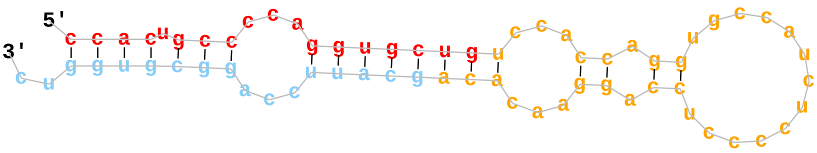

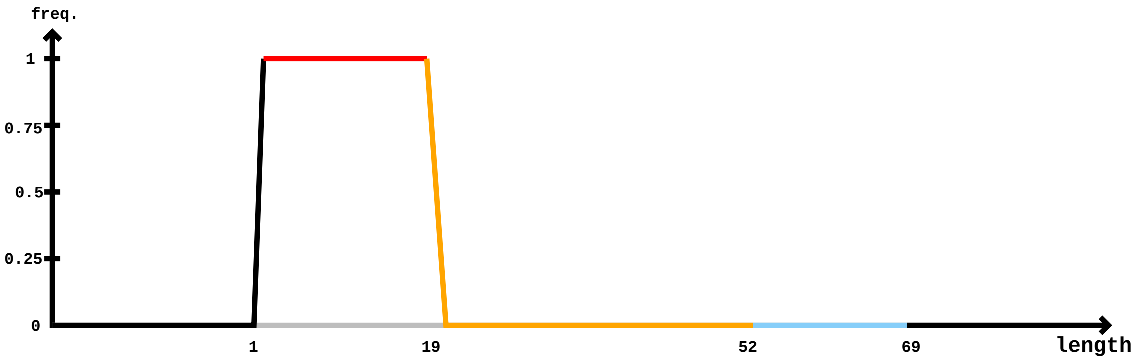

Mature **Star**

```
5'- acguggaggaauccaccaggccacugccccaggugcuguccaccaggugccaucuccccuccaggaacacagcauuccaggcgugguc aaacuccccagagaagcucu   -3'    exp
    ..(((((....)))))..((((((.(((...((((((((...((.((.............)).))...))))))))...)))))))))...(((....))).......   reads      mm         sample
    ....................ccacugccccaggugcug......................................................................   1          0          s12
    ....................ccacugccccaggugcug......................................................................   1          0          s05
    ....................ccacugccccaggugcug......................................................................   2          0          s22
    ....................ccacugccccaggugcug......................................................................   2          0          s06
    ....................ccUcugccccaggugcug......................................................................   1          1          s16
    ....................ccacugccccCggugcug......................................................................   1          1          s17
    ....................ccacugccccaggugcug......................................................................   2          0          s04
    ....................ccacugccccaggugcug......................................................................   2          0          s15
    ....................ccacugccccaggugcug......................................................................   1          0          s01
    ....................ccacugccccaggugcug......................................................................   2          0          s09
    ....................ccacugccccaggugcug......................................................................   3          0          s07
    ....................ccacugccccaggugcug......................................................................   2          0          s14
    ....................ccacugccccaggugcug......................................................................   2          0          s11
    ....................ccacugccccaggugcug......................................................................   1          0          s24
    ....................ccacugccccaggugcug......................................................................   2          0          s23
    ....................ccacugccccaggugcug......................................................................   2          0          s21
    ....................ccacugccccaggugcug......................................................................   1          0          s20
    ....................ccacugccccaggugcug......................................................................   1          0          s08
```

Mature Star

```
acguggaggaauccaccaggccacugccccaggugcuguccaccaggugccaucuccccuccaggaacacagcauuccaggcgugguc aaacuccccagagaagcucu
```

....................ccacugccccaggugcug..	3	0	s10
....................ccacugccccaggugcug..	4	0	s18
....................ccacugccccaggugcug..	4	0	s03
