# Supplementary material for: The Identification of Circulating MiRNA in Bovine Serum and Their Potential as Novel Biomarkers of Early Mycobacterium avium subsp paratuberculosis Infection
Source: PLoS One. 2015 Jul 28;10(7):e0134310. doi: 10.1371/journal.pone.0134310 (PMC4517789; doi:10.1371/journal.pone.0134310)
Supplement: S1 File — (ZIP) [file pone.0134310.s008.zip › novel_pdfs/3_18032.pdf]

Provisional ID	:	3_18032
Score total	:	5.6
Score for star read(s)	:	-1.3
Score for read counts	:	0
Score for mfe	:	2.3
Score for randfold	:	1.6
Score for cons. seed	:	3
Total read count	:	59
Mature read count	:	57
Loop read count	:	1
Star read count	:	1

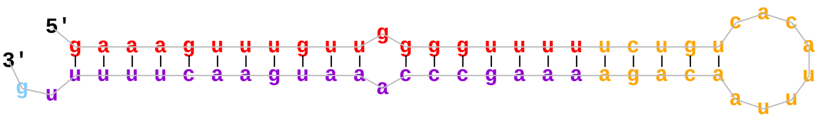

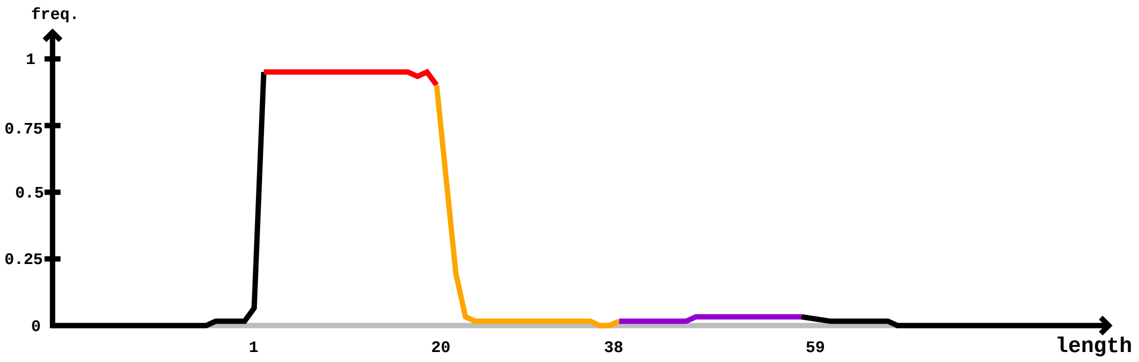

Mature **Star**

```
5'- caauaauauuggguuggccagaaaguuuguugggguuuuucugucacauuuaacagaaaagcccaaaugaacuuuuuggucagcccaacagaaagcaccugguaguga  -3'  obs
    caauaauauuggguuggccagaaaguuuguugggguuuuucugucacauuuaacagaaaagcccaaaugaacuuuuugucagcccaacagaaagcaccugguaguga        exp
    ........(((((((((((((((((((((((.((((((((((((........)))))))))))).))))))))))))))))))))))).......(((......))).  reads  mm  sample
    ....................gaaaguuuguugggguuu......................................................................  1  0  s09
    ....................gaaaguuuguugggguuuu.....................................................................  2  0  s09
    ....................gaaaguuuguugggguuuuuc...................................................................  1  0  s09
    .........................................................aaagcccGaaugaacuuuuu...............................  1  1  s09

    ....................gaaaguuuguugggguuuu.....................................................................  1  0  s19

    ...................agaaaguuuguugggguuuu.....................................................................  1  0  s14
    ....................gaaaguuuguugggguuuu.....................................................................  1  0  s14
    ....................gaaaguuuguugggguuuuucA..................................................................  1  1  s14

    ....................gaaaguuuguugggguuuu.....................................................................  2  0  s07
    ....................gaaaguuuguugggguuuuuc...................................................................  1  0  s07

    ....................gaaaguuuguugggguuuu.....................................................................  1  0  s12
    .................................................................aaugaacuuuuuggucaCccc......................  1  1  s12

    ....................gaaaguuuguugggguuuu.....................................................................  2  0  s15
    ....................gaaaguuuguuggUguuuu.....................................................................  1  1  s15

    ....................gaUaguuuguugggguuuu.....................................................................  1  1  s13

    ....................gaaaguuuguugggguuuu.....................................................................  2  0  s04

    ....................gaaaguuuguugggguuuu.....................................................................  3  0  s01
    ....................gaaaguuuguugggguuuuuc...................................................................  1  0  s01

    ....................gaaaguuuguugggguuuu.....................................................................  1  0  s06
    ....................gaaaguuuguugggguuuuuc...................................................................  1  0  s06

    ...................agaaaguuuguugggguuuu.....................................................................  1  0  s22
    ....................gaaaguuuguugggguuuu.....................................................................  1  0  s22

    ....................gaaaguuuguugggguuuu.....................................................................  1  0  s16
```

Mature Star

caauaauauuggguuggccagaaaguuuguugggguuuuucugucacauuuaacagaaaagcccaaaugaacuuuuuggucagcccaacagaaagcaccugguaguga

```
....................gaaaguuuguugggguuuu...................................................................	2	0	s17

....................gUaaguuuguugggguuuu...................................................................	1	1	s18
....................gaaaguuuguugggguuuu...................................................................	1	0	s18
....................gaaaguuuguugggguuuuu..................................................................	1	0	s18

....................gaaaguuuguugggguuu....................................................................	1	0	s08
....................gaaaguuuguugggguuuu...................................................................	1	0	s08
....................gaaaguuuguugggguuuuuc.................................................................	2	0	s08

....................gaaaguuuguugggguuu....................................................................	1	0	s10
....................gaaaguuuguugggguuuu...................................................................	2	0	s10
....................gaaaguuuguugggguuuuuc.................................................................	1	0	s10
.....................................uuCcugucacauuuaaca...................................................	1	1	s10

...................agaaCguuuguugggguuuu...................................................................	1	1	s03
....................gaaaguuuguugggguuuu...................................................................	4	0	s03
....................gaaaAuuuguugggguuuu...................................................................	1	1	s03
....................gaaaguuuguugggguuuuuc.................................................................	1	0	s03

....................gaaaguuuguugggguuuu...................................................................	1	0	s21
....................gaaaguuuguugggguuuuuc.................................................................	2	0	s21

....................gaaaguuuguugggguuuu...................................................................	1	0	s23

...............ggccaAaaaguuuguuggggu......................................................................	1	1	s11
....................gaaaguuuguugggguuuu...................................................................	6	0	s11
```
