# Supplementary material for: The Identification of Circulating MiRNA in Bovine Serum and Their Potential as Novel Biomarkers of Early Mycobacterium avium subsp paratuberculosis Infection
Source: PLoS One. 2015 Jul 28;10(7):e0134310. doi: 10.1371/journal.pone.0134310 (PMC4517789; doi:10.1371/journal.pone.0134310)
Supplement: S1 File — (ZIP) [file pone.0134310.s008.zip › novel_pdfs/10_1684.pdf]

Provisional ID	:	10_1684
Score total	:	4.9
Score for star read(s)	:	-1.3
Score for read counts	:	0
Score for mfe	:	1.6
Score for randfold	:	1.6
Score for cons. seed	:	3
Total read count	:	1115
Mature read count	:	1115
Loop read count	:	0
Star read count	:	0

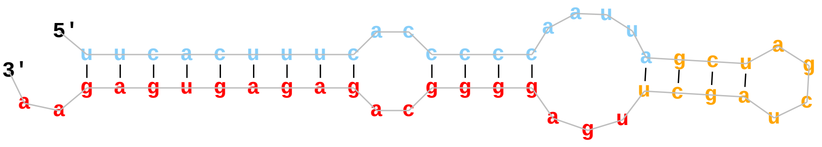

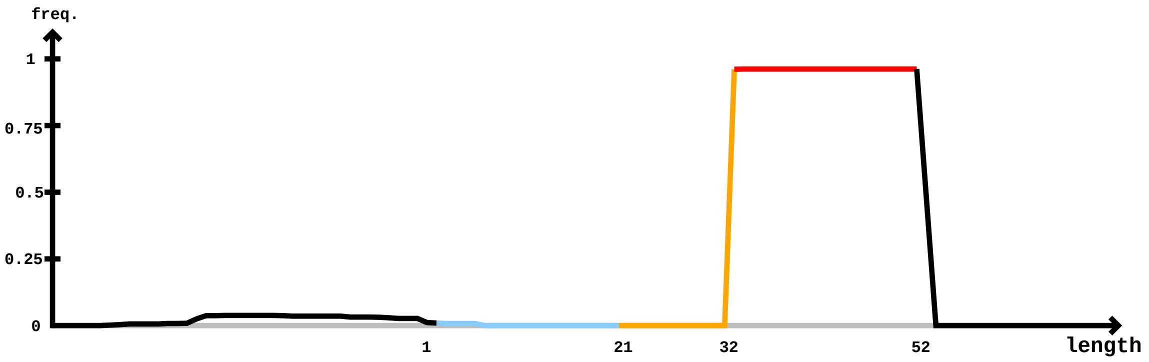

Star Mature

```
5'- ccauggggucacaaagagucggauaugacugagugacuuucacuuucacccccaauuagcuagcuagcuugaggggcagagagugagaaggcaggcucaucccccuugaa  -3'   exp
    .((.((((.........((((....))))(((((..(((((((((((..((((....((((....))))...))))..))))))))).))....))))).)))).))..   reads    mm    sample
    .............aagagucggaCaugacugagugacuuucac..................................................................   1        1     s03
    ..............agagucggaAaugacuga.............................................................................   1        1     s03
    .....................................................................ugaggggcagagagCgagaa....................   57       1     s03
    .....................................................................ugaggggcagagagugagaC....................   1        1     s03
    .....................................................................ugaggggcagagagCgagaag...................   3        1     s03

    .............aagagucggaCaugacugagugacuuucac..................................................................   1        1     s18
    .....................................................................ugaggggcagagagugagaC....................   1        1     s18
    .....................................................................ugaggggcagagagCgagaa....................   23       1     s18
    .....................................................................ugaggggcagagagCgagaag...................   1        1     s18

    .....................................................................ugaggggcagagagugagaC....................   1        1     s08
    .....................................................................ugaggggcagagagCgagaa....................   52       1     s08
    .....................................................................ugaggggcagagagCgagaag...................   6        1     s08

    .....gggucacaaagagucggauaCgac................................................................................   1        1     s10
    ..........acaGagagucggauaugac................................................................................   1        1     s10
    .....................................................................ugaggggcagagagCgagaa....................   34       1     s10
    .....................................................................ugaggggcagagagugagaC....................   2        1     s10
    .....................................................................ugaggggcagagagCgagaag...................   3        1     s10

    ..............agagucggaCaugacugagugacuu......................................................................   1        1     s21
    .....................................................................ugaggggcagagagCgagaa....................   37       1     s21
    .....................................................................ugaggggcagagagCgagaag...................   3        1     s21

    .....gggucacaGagagucgga......................................................................................   1        1     s24
    .............aagagucggaCaugacugagugacuuucac..................................................................   2        1     s24
    .....................................................................ugaggggcagagagugagaC....................   5        1     s24
    .....................................................................ugaggggcagagagCgagaa....................   53       1     s24
    .....................................................................ugaggggcagagagCgagaag...................   1        1     s24

    ..............agagucggauaugacugagCgac........................................................................   1        1     s23
    .....................................................................ugaggggcagagagCgagaa....................   29       1     s23
    .....................................................................ugaggggcagagagugagaC....................   1        1     s23
```

```
                                      Star                          Mature
ccauggggucacaaagagucggauaugacugagugacuuucacuuucacccccaauuagcuagcuagcuugaggggcagagagugagaaggcaggcucaucccuugaa
.....................................................................ugaggggcagagagCgagaag..................    1    1    s23

......ggucGcaaagagucggauaugac...............................................................................    1    1    s20
................agucggauaCgacugagugac.......................................................................    1    1    s20
.....................................................................ugaggggcagagagugagaC...................    1    1    s20
.....................................................................ugaggggcagagagCgagaa...................    24   1    s20
.....................................................................ugaggggcagagagCgagaag..................    3    1    s20

.............aagagucggauaCgacugagugac.......................................................................    1    1    s11
..............agagucggauaugacugagCgac.......................................................................    1    1    s11
.....................................................................ugaggggcagagagugagaC...................    3    1    s11
.....................................................................ugaggggcagagagCgagaa...................    84   1    s11
.....................................................................ugaggggcagagagCgagaag..................    7    1    s11

.....................................................................ugaggggcagagagCgagaa...................    45   1    s14
.....................................................................ugaggggcagagagCgagaag..................    3    1    s14

......ggucacaaagagucggauaCgacugag...........................................................................    1    1    s07
.............aagagucAgauaugacugagugac.......................................................................    1    1    s07
..............agagucggauaugacugagC..........................................................................    1    1    s07
.....................................................................ugaggggcagagagugagaC...................    2    1    s07
.....................................................................ugaggggcagagagCgagaa...................    17   1    s07

......ggucacaaagaguUggauaugacugag...........................................................................    1    1    s09
.............aagagucggauaCgacugagugac.......................................................................    2    1    s09
.....................................................................ugaggggcagagagCgagaa...................    51   1    s09
.....................................................................ugaggggcagagagugagaC...................    3    1    s09
.....................................................................ugaggggcagagagCgagaag..................    4    1    s09

..........acaaagagucggauaCgac...............................................................................    1    1    s19
..............agagucggauaugacugagC..........................................................................    1    1    s19
.....................................................................ugaggggcagagagugagaC...................    3    1    s19
.....................................................................ugaggggcagagagCgagaa...................    84   1    s19
.....................................................................ugaggggcagagagCgagaag..................    2    1    s19

.....................................................................ugaggggcagagagCgagaa...................    48   1    s15
.....................................................................ugaggggcagagagAgagaa...................    1    1    s15
.....................................................................ugaggggcagagagugagaC...................    3    1    s15
.....................................................................ugaggggcagagagCgagaag..................    4    1    s15
......................................................................gaggggcagagagCgagaa...................    1    1    s15

.............aagagucggauaugacugagCgac.......................................................................    1    1    s04
..............agagucggauaugacugagCgac.......................................................................    1    1    s04
..............agagucggauaCgacugagugac.......................................................................    1    1    s04
..............agagucggauaCgacugagugacu......................................................................    1    1    s04
..............agagucggaCaugacugagugacuu.....................................................................    1    1    s04
.....................................................................ugaggggcagagagCgagaa...................    64   1    s04
.....................................................................ugaggggcagagagugagaC...................    1    1    s04
.....................................................................ugaggggcagagagCgagaag..................    3    1    s04

....gggUucacaaagagucgga.....................................................................................    1    1    s13
..............agagucggauaugacugagugac.......................................................................    1    0    s13
.....................................................................ugaggggcagagagCgagaa...................    9    1    s13

.............aagagucggauaugacugagugac.......................................................................    1    0    s01
.....................................................................ugaggggcagagagCgagaa...................    51   1    s01
.....................................................................ugaggggcagagagugagaC...................    1    1    s01
.....................................................................ugaggggcagagagCgagaag..................    3    1    s01

....ggggucacaaagagucgg......................................................................................    1    0    s05
.....................................................................ugaggggcagagagCgagaa...................    31   1    s05
.....................................................................ugaggggcagagagugagaC...................    5    1    s05

.............aagagucggaCaugacugagugacuuucac.................................................................    1    1    s22
.....................................................................ugaggggcagagagCgagaa...................    31   1    s22
.....................................................................ugaggggcagagagCgagaag..................    1    1    s22

............aaagagucggauaugacugagugac.......................................................................    1    0    s16
.....................................................................ugaggggcagagagCgagaa...................    26   1    s16
.....................................................................ugaggggcagagagCgagaag..................    1    1    s16
```

Star **Mature**

```
ccauggggucacaaagagucggauaugacugagugacuuucacuuucacccccaauuagcuagcuagcuugaggggcagagagugagaaggcaggcucauccccuugaa

.............aagagucggauaCgacugagugac........................................................................   2    1    s06
.....................................................................ugaggggcagagagCgagaa....................   15   1    s06
.....................................................................ugaggggcagagagugagaC....................   1    1    s06
.....................................................................ugaggggcagagagCgagaag...................   2    1    s06

.............aagagucggauaugacugagCgac........................................................................   1    1    s17
..............agaguUggauaugacugagugac........................................................................   1    1    s17
.....................................................................ugaggggcagagagugagaC....................   3    1    s17
.....................................................................ugaggggcagagagCgagaa....................   42   1    s17
.....................................................................ugaggggcagagagCgagaag...................   2    1    s17

.............aagagucggaCaugacugagugacuuucac..................................................................   1    1    s02
.....................................................................ugaggggcagagagCgagaa....................   26   1    s02
.....................................................................ugaggggcagagagugagaC....................   1    1    s02
.....................................................................ugaggggcagagagCgagaag...................   1    1    s02

.............aagaguUggauaugacugagugac........................................................................   1    1    s12
.............aagagucggaCaugacugagugacuuucac..................................................................   3    1    s12
..............agagucggauaugacugagC...........................................................................   1    1    s12
..............agagucggauaugacugGgugacu.......................................................................   1    1    s12
.....................................................................ugaggggcagagagCgagaa....................   83   1    s12
.....................................................................ugaggggcagagagCgagaag...................   5    1    s12
```
