# Supplementary material for: The Identification of Circulating MiRNA in Bovine Serum and Their Potential as Novel Biomarkers of Early Mycobacterium avium subsp paratuberculosis Infection
Source: PLoS One. 2015 Jul 28;10(7):e0134310. doi: 10.1371/journal.pone.0134310 (PMC4517789; doi:10.1371/journal.pone.0134310)
Supplement: S1 File — (ZIP) [file pone.0134310.s008.zip › novel_pdfs/11_2549.pdf]

Provisional ID	:	11_2549
Score total	:	33.7
Score for star read(s)	:	3.9
Score for read counts	:	26.6
Score for mfe	:	2.2
Score for randfold	:	1.6
Score for cons. seed	:	-0.6
Total read count	:	64
Mature read count	:	60
Loop read count	:	0
Star read count	:	4

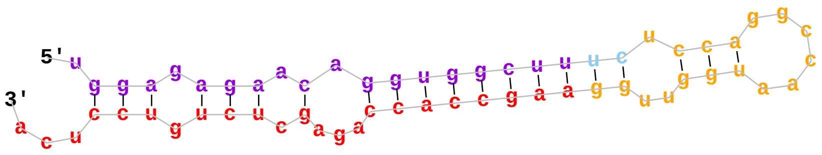

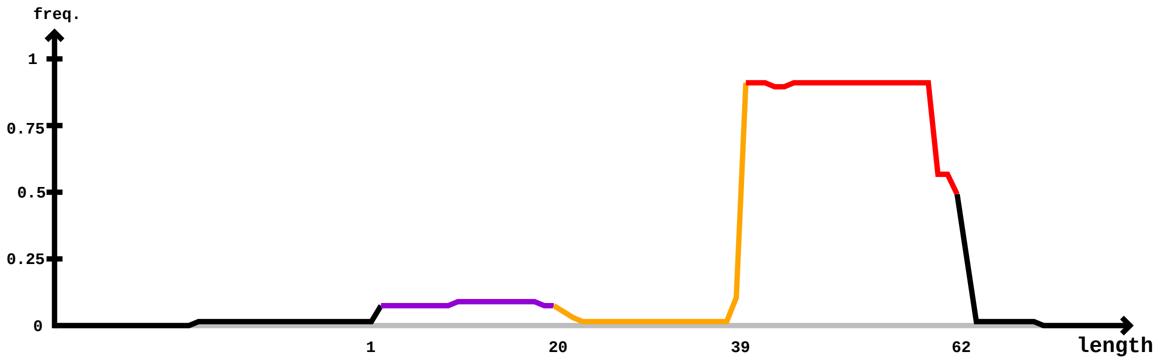


```
                                     Star                                         Mature
5'- ucucagugggaccccaaaugagacccuggauauggagagaacagguggcuuucuccaggccaaugguuggaagccaccagagcucuguccucauccaggucucaacucac  -3'   obs
    ucucagugggaccccaaaugagacccuggauauggagagaacagguggcuuucuccaggccaaugguuggaagccaccagagcucuguccucauccaggucucaacucac        exp
    ......(((....)))..(((((((.(((((..(((.(((.(.((((((((((.(((......)))..))))))))))...).))).)))..))))))))))))......   reads   mm   sample
    ......................................................................aagccaccagagcucuguccuca.................   2       0    s21

    ......................................................................aagccaccagagcucugucc....................   1       0    s23
    ......................................................................aagccaccagagcucuguccuca.................   1       0    s23

    .............ccaaaugagacccuggauauggagagaacagguggc.............................................................   1       0    s24
    ................................uggagagaacagguggcuu...........................................................   1       0    s24
    ......................................................................aagccaccagagcucuguccuca.................   1       0    s24
    ......................................................................aagcAaccagagcucuguccuca.................   1       1    s24

    .....................................................................gaagccaccagagcucugucc....................   1       0    s11
    ......................................................................aagccaccagagcucugucc....................   1       0    s11

    ......................................................................aagccaccagagcucugucc....................   1       0    s03
    ...........................................................................accagagcucuguccucauccagguc.........   1       0    s03

    ................................uggagagaacagguggcuu...........................................................   1       0    s18
    ......................................................................aagccaccagCgcucugucc....................   1       1    s18
    ......................................................................aagccaccagagcucuguccuca.................   3       0    s18
    ......................................................................aagcGaccagagcucuguccuca.................   1       1    s18

    .....................................................................gaUgccaccagagcucugucc....................   1       1    s10
    ......................................................................aagccaccagagcucugucc....................   1       0    s10
    ......................................................................aagccaccagagcucuguccuc..................   1       0    s10
    ......................................................................aagccaccagagcucuguccuca.................   1       0    s10

    ......................................................................aagcAaccagagcucuguccuc..................   1       1    s01
    ......................................................................aagccaccagagcucuguccuca.................   1       0    s01

    .....................................................................gaagccaccagagcucuguccuc..................   1       0    s13
    ......................................................................aagGcaccagagcucugucc....................   1       1    s13

    .....................................................................gaagccaccagagcucugucc....................   1       0    s15
```

```
                                Star                                    Mature
ucucagugggaccccaaaugagacccuggauauggagagaacagguggcuuucuccaggccaaugguuggaagccaccagagcucuguccucauccaggucucaacucac
......................................................................aagccaccagagcucugucc....................   1   0   s15

......................................................................aagcAaccagagcucugucc....................   1   1   s04
......................................................................aagccaccagagcucugucc....................   1   0   s04

................................uggagagaacagguggcuu...........................................................   1   0   s17
......................................................................aagccaccagagcucuguccuca.................   2   0   s17

......................................................................aagccaccagagcucugucc....................   1   0   s02
......................................................................aagccaccagagcucuguccuca.................   2   0   s02

......................................................................aagccaccagagcucuguccucaG................   1   1   s22

......................................................................aagcAaccagagcucuguccuca.................   1   1   s16

......................................................................aagcAaccagagcucugucc....................   1   1   s05
......................................................................aagccaccagagcucugucc....................   3   0   s05
......................................................................aagccaccagagcucuguccuca.................   1   0   s05

......................................................................aagccaccagagcucugucc....................   1   0   s06
......................................................................aagccaccagagcucuguccuca.................   1   0   s06

........................................acagguggcuuucuccaggccaaugguuggaag.....................................   1   0   s12
.....................................................................gaagccaccagagcucuguccuc..................   1   0   s12
......................................................................aagccaccagagcucugucc....................   2   0   s12
......................................................................aagccaccagagcucuguccuca.................   2   0   s12

......................................................................aagccaccagagcucuguccuca.................   2   0   s14

......................................................................aagccaccagagcucugucc....................   1   0   s07
......................................................................aaUccaccagagcucuguccuca.................   1   1   s07
......................................................................aagcGaccagagcucuguccuca.................   1   1   s07
......................................................................aagcAaccagagcucuguccuca.................   1   1   s07

................................uggagagaacagguggcuuuc.........................................................   1   0   s19
.....................................................................gaagccaccagagcucuguccuA..................   1   1   s19
......................................................................aagccaccagagcucugucc....................   2   0   s19
......................................................................aagccaccagagcucuguccuca.................   6   0   s19
```
