# Supplementary material for: The Identification of Circulating MiRNA in Bovine Serum and Their Potential as Novel Biomarkers of Early Mycobacterium avium subsp paratuberculosis Infection
Source: PLoS One. 2015 Jul 28;10(7):e0134310. doi: 10.1371/journal.pone.0134310 (PMC4517789; doi:10.1371/journal.pone.0134310)
Supplement: S1 File — (ZIP) [file pone.0134310.s008.zip › novel_pdfs/3_18843.pdf]

Provisional ID	:	3_18843
Score total	:	71.7
Score for star read(s)	:	3.9
Score for read counts	:	60.8
Score for mfe	:	2.5
Score for randfold	:	1.6
Score for cons. seed	:	3
Total read count	:	131
Mature read count	:	72
Loop read count	:	0
Star read count	:	59

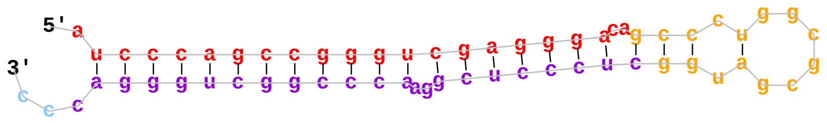

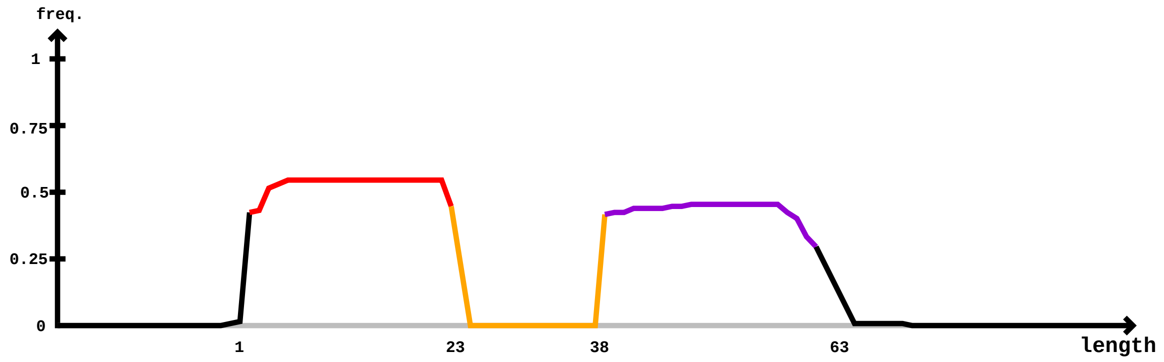

Mature Star

```
5'- uugggucccggagaccgaaucccagccgggucgagggacagcccuggcgcgauggcucccucggaacccggcugggacccccucggcuucgcccccuggagcgguccgc   -3'    obs
    uugggucccggagaccgaaucccagccgggucgagggacagcccuggcgcgauggcucccucggaacccggcugggacccccucggcuucgcccccuggagcgguccgc          exp
    ..(((...(((((.((((.((((((((((((((((((..(((.(......).))))))))))..)))))))))))).....))))))))).)))..((((....)))).    reads     mm     sample
    ..................aucccagccgggucgagggaca.....................................................................    2         0      s03
    .......................................................cucccucggaacccggcuggg.................................    1         0      s03
    .......................................................cuccGucggaacccggcuggg.................................    1         1      s03
    .......................................................cucccucggaacccggcugggac...............................    1         0      s03

    .......................................................cuUccucggaacccggcugggac...............................    1         1      s18
    .......................................................cucccucggaacccggcugggacc..............................    1         0      s18

    ..................aucccagccggguGgagggaca.....................................................................    1         1      s10
    ..................Uucccagccgggucgagggaca.....................................................................    1         1      s10
    .......................................................cucccucggaacccggcuggg.................................    1         0      s10
    .......................................................cucccucggaacccggcugggac...............................    2         0      s10

    ..................auccAagccgggucgagggac......................................................................    1         1      s08
    ..................aucccagccgggucgagUgaca.....................................................................    1         1      s08
    ..................aucccagccgggucgagggaca.....................................................................    5         0      s08
    ....................cccagccgggucgagggac......................................................................    1         0      s08
    ....................cccagccgggucgagggaca.....................................................................    1         0      s08
    .......................................................cucccucggaacccggcuggg.................................    1         0      s08
    .......................................................cucccucggaacccggcuggg.................................    1         0      s08

    ..................aucccagccgggucgagggac......................................................................    1         0      s20
    ..................aucccagccgggucgagggaca.....................................................................    2         0      s20
    ....................cccagccgggucgagggaca.....................................................................    2         0      s20
    .......................................................cucccucggaacccggcuggga................................    2         0      s20
    .......................................................cucccucggaacccggcugggac...............................    1         0      s20

    .......................................................cucccucggaacccggcugg..................................    1         0      s21
    .......................................................cucccucggaacccggcugggac...............................    2         0      s21

    ..................aucccagccgggucgagggaca.....................................................................    1         0      s23
    .......................................................cucccucggaacccggcuggga................................    1         0      s23
    .......................................................cuccAucggaacccggcugggac...............................    1         1      s23
```

```
                  Mature                              Star
uugggucccggagaccgaaucccagccgggucgagggacagcccuggcgcgaugggcucccucggaacccggcugggacccccucggcuucgccccccuggagcgguccgc
.......................................................cucccucggaacccggcugggac................................    1    0    s23
.......................................................cucccucggaacccggcugggaccc..............................    1    0    s23

.......................................................cucccucggaacccggcug....................................    1    0    s11
.......................................................cucccucggaacccggcuggg..................................    1    0    s11
.......................................................cuUccucggaacccggcugggac................................    1    1    s11

..................aucccagccgggucgagggac.......................................................................    2    0    s14
..................aucccagccgggucgagggaca......................................................................    3    0    s14
...................Ccccagccgggucgagggac.......................................................................    1    1    s14
......................cagccgggucgagggaca......................................................................    1    0    s14
.......................................................cucccucggaacccggcug....................................    1    0    s14
.......................................................cucccucggaacccggcugggac................................    1    0    s14
..........................................................ccucggaacccggcugggaccc..............................    1    0    s14

..................aucccagccgggucgagggaca......................................................................    2    0    s07
....................cccagccgggucgagggac.......................................................................    1    0    s07
.......................................................cucccucggaacccggcuggg..................................    1    0    s07
.......................................................cucccucggaacccggcugggac................................    1    0    s07
................................................................aacccggcugggaccccucggc........................    1    0    s07

.................aaCcccagccgggucgagggaca......................................................................    1    1    s09
..................aucccagccgggucgagggaca......................................................................    2    0    s09
.....................ccagccgggucgagggaca......................................................................    1    0    s09
.......................................................cucccucggaacccggcuggg..................................    1    0    s09
.......................................................cucccucggaacccggcugggac................................    2    0    s09

..................aucccagccgggucgagggac.......................................................................    1    0    s19
..................aucccagccgggucgagggaca......................................................................    1    0    s19

................gaaucccagccgggucgagggaca......................................................................    1    0    s01
..................aucccagccgggucgagggaca......................................................................    4    0    s01
..................aCcccagccgggucgagggaca......................................................................    1    1    s01
..................Uucccagccgggucgagggaca......................................................................    1    1    s01
.......................................................cucccucggaacccggcugggac................................    1    0    s01
.......................................................cucccucggaacccggcugggacc...............................    1    0    s01
..........................................................ccCcggaacccggcugggac................................    1    1    s01

..................aucccagccgggucgagggaca......................................................................    1    0    s15
.......................................................cucccucggaacccggcugg...................................    1    0    s15
.......................................................cuUccucggaacccggcuggg..................................    1    1    s15
.......................................................cucccucggaacccggcuggg..................................    1    0    s15
.......................................................cucccucggaacccggcugggac................................    1    0    s15
..............................................................ggaaGccggcugggaccc..............................    1    1    s15

..................aucccagccgggucgagggac.......................................................................    1    0    s13
..................auUccagccgggucgagggaca......................................................................    1    1    s13
..................aucccagccgggucgagggaca......................................................................    2    0    s13
.......................................................cucccucggaacccggcugggac................................    2    0    s13
.......................................................cucccucggaCcccggcugggacc...............................    1    1    s13

..................aucccagccgggucgagggac.......................................................................    1    0    s04
..................aucccagccgggucgagggaca......................................................................    1    0    s04

..................aucccagccgggucgagggaca......................................................................    1    0    s02
..................aucccagccgggucgCgggaca......................................................................    1    1    s02
......................cagccgggucgagggaca......................................................................    1    0    s02

..................aucccagccgggucgagggaca......................................................................    2    0    s17
....................cccagccgggucgagggac.......................................................................    1    0    s17
.......................................................cucccucggaacccggcug....................................    1    0    s17
.......................................................cucccucggaacccggcugggac................................    1    0    s17
........................................................ucccucggaacccggcugggacc..............................    1    0    s17

..................aucccagccgggucgagggaca......................................................................    2    0    s22
.......................................................cucccucggaacccggcug....................................    1    0    s22
.......................................................cucccucggaacccggcugggac................................    3    0    s22

..................aucccagccgggucgagggac.......................................................................    1    0    s05
.......................................................cucccucggaacccggcugggac................................    1    0    s05
```

```
                  Mature                               Star
uugggucccggagaccgaaucccagccgggucgagggacagcccuggcgcgaugggcucccucggaacccggcugggacccccucggcuucgcccccuggagcgguccgc
.......................................................cucccucggaacccggcugggacc..............................    1    0    s05

..................aucccagccgggucgagggac......................................................................    1    0    s16
..................aucccagccgggucgCgggaca.....................................................................    2    1    s16
..................aucccagccgggucgagggaca.....................................................................    3    0    s16
....................cccagccgggucgagggaca.....................................................................    2    0    s16
.....................ccagccgggucgagggaca.....................................................................    1    0    s16
.......................................................cucccucggaacccggcuggga................................    2    0    s16
.......................................................cGcccucggaacccggcugggac...............................    1    1    s16
.......................................................cucccucggaacccggcugggac...............................    1    0    s16
.......................................................cuccAucggaacccggcugggaccc.............................    1    1    s16

..................aucccagccgggucgagggaca.....................................................................    2    0    s06
....................cccagccgggucgagggaca.....................................................................    2    0    s06
.......................................................cucccucggaacccggcugggacc..............................    1    0    s06

....................cccagccgggucgagggaca.....................................................................    1    0    s12
.......................................................cucccucggaacccggcugggac...............................    2    0    s12
```
