# Supplementary material for: The Identification of Circulating MiRNA in Bovine Serum and Their Potential as Novel Biomarkers of Early Mycobacterium avium subsp paratuberculosis Infection
Source: PLoS One. 2015 Jul 28;10(7):e0134310. doi: 10.1371/journal.pone.0134310 (PMC4517789; doi:10.1371/journal.pone.0134310)
Supplement: S1 File — (ZIP) [file pone.0134310.s008.zip › novel_pdfs/5_20820.pdf]

Provisional ID	:	5_20820
Score total	:	5.2
Score for star read(s)	:	-1.3
Score for read counts	:	0
Score for mfe	:	1.9
Score for randfold	:	1.6
Score for cons. seed	:	3
Total read count	:	78
Mature read count	:	78
Loop read count	:	0
Star read count	:	0

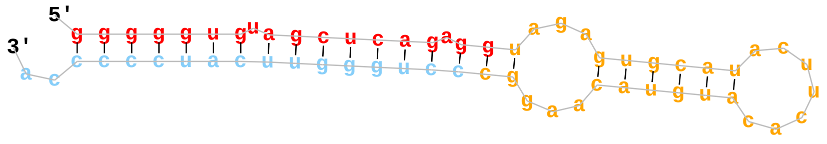

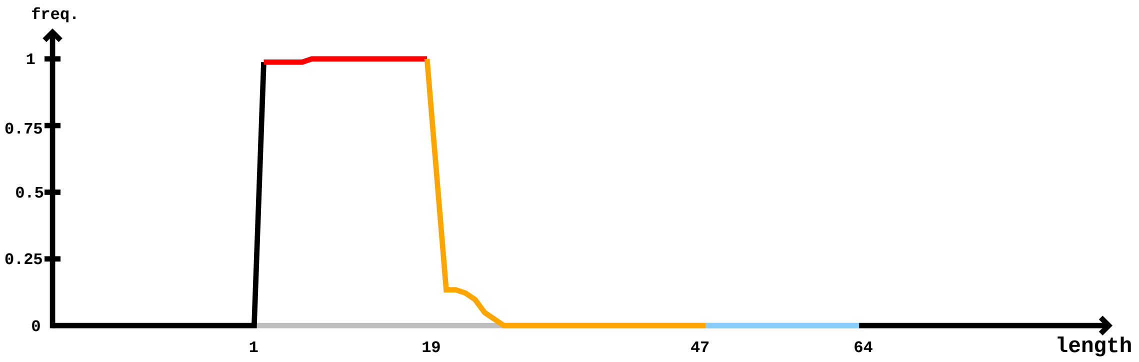

Mature **Star**

```
5'- gaaucugccugccagagcaaggggguguagcucagagguagagugcauacuucacauguacaaggcccuggguucaucccccagcuccuccaucugugucuuucgggc   -3'   exp
    ......(((((...((((..(((((((.(((((((.(((...((((((.......))))))...)))))))))))))))))..))))................)))))   reads   mm   sample
    ....................ggggguguagcucagagg......................................................................   2   0   s11
    ....................ggggAuguagcucagagg......................................................................   2   1   s11
    ....................ggggguguagcucagGgg......................................................................   1   1   s11

    ....................ggggguguagcucagagg......................................................................   2   0   s23
    ....................ggggguguagcucagGgg......................................................................   1   1   s23
    ....................ggggguguagcucagaggGagag.................................................................   1   1   s23

    ....................ggggguguagcucagGgg......................................................................   1   1   s21

    ....................ggggguguagcucagagg......................................................................   3   0   s24
    ....................gggUguguagcucagagg......................................................................   1   1   s24
    ....................ggggguguagcucagCgg......................................................................   2   1   s24
    ....................ggggguguagcucagGgg......................................................................   2   1   s24
    ....................ggggAuguagcucagagg......................................................................   1   1   s24

    ....................ggggguguagcucagGgg......................................................................   1   1   s20

    ....................ggggguguagcucagCgg......................................................................   1   1   s08
    ....................ggggguguagcucagGgg......................................................................   1   1   s08
    ....................ggggguguagcucagagguagagC................................................................   1   1   s08

    ....................ggggAuguagcucagagg......................................................................   1   1   s18
    ....................ggggguguagcucagGgguagag.................................................................   1   1   s18

    ....................ggggguguagcucagagg......................................................................   2   0   s03
    ....................ggggguguagcucagGgg......................................................................   1   1   s03
    ....................ggggAuguagcucagagg......................................................................   1   1   s03

    ....................ggggguguagcucagaUg......................................................................   1   1   s12
    ....................ggggAuguagcucagagg......................................................................   2   1   s12
    ....................ggggguguagcucagagg......................................................................   3   0   s12
    ....................ggggCuguagcucagagg......................................................................   1   1   s12

    ....................ggggguguagcucagGgg......................................................................   3   1   s05
```

```
                    Mature                                      Star
gaaucugccugccagagcaaggggguguagcucagagguagagugcauacuucacauguacaaggcccuggguucaucccccca gcuccuccaucugugucuuucgggc
....................ggggguguagcucagCgg.......................................................................   1   1   s05
....................ggggAuguagcucagagg.......................................................................   1   1   s05
....................ggggguguagcucagagg.......................................................................   1   0   s05
....................ggggguguagcucagGgguaga...................................................................   1   1   s05

....................ggggAuguagcucagagg.......................................................................   3   1   s22
....................gCggguguagcucagagguag....................................................................   1   1   s22

....................ggggguguaAcucagagg.......................................................................   1   1   s16
....................ggggCuguagcucagagg.......................................................................   1   1   s16
....................ggggAuguagcucagagg.......................................................................   1   1   s16
....................ggggguguagcucagGgg.......................................................................   1   1   s16
....................ggggguguagcucagagg.......................................................................   1   0   s16

....................ggggguguagcucagGgg.......................................................................   1   1   s06
....................ggggguguagcucagagg.......................................................................   1   0   s06
....................ggggguguagcucagCgg.......................................................................   2   1   s06
....................ggggguguagcucagagguagagCg................................................................   1   1   s06

....................ggggAuguagcucagagg.......................................................................   1   1   s17

....................ggggguguagcucagGgg.......................................................................   1   1   s02
....................ggggguguagcucagagg.......................................................................   1   0   s02
....................ggggguguaAcucagagg.......................................................................   1   1   s02
....................ggggAuguagcucagagg.......................................................................   1   1   s02
....................ggggguguagcucagCgguaga...................................................................   1   1   s02

....................ggggguguagcucagagguagagCg................................................................   1   1   s04

....................ggggguguagcucagagg.......................................................................   1   0   s15
....................gCggguguagcucagagguagag..................................................................   1   1   s15

....................ggggguguagcucagagg.......................................................................   2   0   s01
....................ggggguguagcucagGgg.......................................................................   2   1   s01
....................gggUguguagcucagagg.......................................................................   1   1   s01
.........................uguagcucagGgguagag..................................................................   1   1   s01

....................ggggCuguagcucagagg.......................................................................   1   1   s09
....................ggggguguagcucagGgg.......................................................................   1   1   s09
....................ggggguguagcucagCgg.......................................................................   2   1   s09
....................gggUguguagcucagagg.......................................................................   1   1   s09

....................ggggguguagcucagagg.......................................................................   1   0   s07
....................ggggguguagcucagGgg.......................................................................   1   1   s07

....................ggggguguaAcucagagg.......................................................................   1   1   s14
....................ggggguguagcucagaUg.......................................................................   1   1   s14
....................ggggguguagcucagagguagagC.................................................................   1   1   s14
```
