# Supplementary material for: The Identification of Circulating MiRNA in Bovine Serum and Their Potential as Novel Biomarkers of Early Mycobacterium avium subsp paratuberculosis Infection
Source: PLoS One. 2015 Jul 28;10(7):e0134310. doi: 10.1371/journal.pone.0134310 (PMC4517789; doi:10.1371/journal.pone.0134310)
Supplement: S1 File — (ZIP) [file pone.0134310.s008.zip › novel_pdfs/15_6321.pdf]

Provisional ID	:	15_6321
Score total	:	79.7
Score for star read(s)	:	3.9
Score for read counts	:	72
Score for mfe	:	2.8
Score for randfold	:	1.6
Score for cons. seed	:	-0.6
Total read count	:	153
Mature read count	:	76
Loop read count	:	0
Star read count	:	77

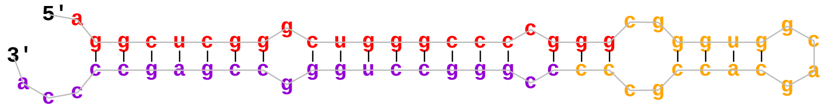

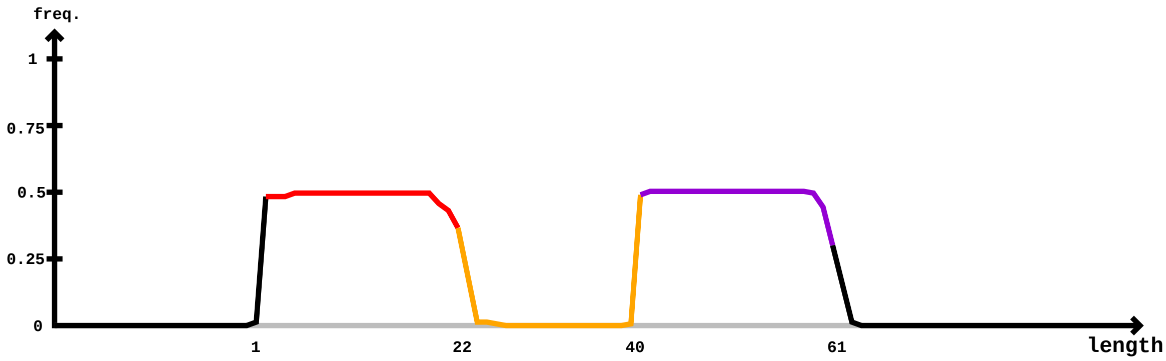

Mature **Star**

```
5'- agcucccucugggagcggucaggcucgggcugggccccgggcggguggcagcaccgcccccgggccugggccgagccccagagcggcggaucucugcggaucugcggagug   -3'    obs
    agcucccucugggagcggucaggcucgggcugggccccgggcggguggcagcaccgcccccgggccugggccgagccccagagcggcggaucucugcggaucugcggagug          exp
    .((((((...))))))....(((((((.((((((((.(((..((((....))))..))).)))))))).)))))))......(.((((((((....)))))))).)....    reads     mm     sample
    ....................aggcucgggcugggccccggg......................................................................    2         0      s07

    ....................aggcucgggcugggccccgg.......................................................................    1         0      s14
    ....................aggGucgggcugggccccggg......................................................................    1         1      s14
    ....................aggcucgggcugggccccggg......................................................................    2         0      s14
    ....................aggcucgggcugggccccgggU.....................................................................    1         1      s14
    ...........................................................ccgggccugggccgagccccag..............................    2         0      s14

    ....................aggcucgggcugggcccc.........................................................................    1         0      s19
    ....................aggcucgggcugggccccgggU.....................................................................    1         1      s19
    ...........................................................ccgggccugggccgagccccag..............................    1         0      s19

    ....................aggcucgggcugggccccggg......................................................................    2         0      s09
    ....................aggcucgggcugggccccgggU.....................................................................    1         1      s09
    ...........................................................ccgggccugggccgagcccc................................    3         0      s09
    ...........................................................ccggCccugggccgagcccca...............................    1         1      s09
    ...........................................................ccgggccugggccgagcccca...............................    1         0      s09

    ...................caggcucgggcugggccccggg......................................................................    1         0      s22
    ....................aggcucgggcugggcccc.........................................................................    1         0      s22
    ....................agUcucgggcugggcccc.........................................................................    1         1      s22
    ....................aggcucgggcugggccccg........................................................................    1         0      s22
    ....................aggcucgggcugggccccgg.......................................................................    1         0      s22
    ....................aggcucgggcugggccccggg......................................................................    4         0      s22
    ...........................................................ccgggccugggccgagcccc................................    3         0      s22

    ...................caggcucgggcugggccccggg......................................................................    1         0      s06
    ....................aggcucgggcugggccccgg.......................................................................    1         0      s06
    ...........................................................cUgggccugggccgagcccca...............................    1         1      s06
    ...........................................................ccgUgccugggccgagcccca...............................    1         1      s06

    ....................aggcucgggcugggccccgg.......................................................................    1         0      s05
    ....................aggcucgggcugggccccggg......................................................................    2         0      s05
```

```
                    Mature                                  Star
agcucccucugggagcggucaggcucgggcugggccccgggcggguggcagcaccgccccgggccugggccgagccccagagcggcggaucucugcggaucugcggagug
...........................................................ccgggccugggccgagcccc................................    2    0    s05
...........................................................ccgggccugggccgagcccca...............................    2    0    s05

....................aggcucgggcugggccccggg......................................................................    2    0    s17
....................aggcucgggcugggccccgggU.....................................................................    1    1    s17
...........................................................ccgggccugggccgagcccc................................    1    0    s17
...........................................................ccgggccugggccgagcccca...............................    2    0    s17
...........................................................ccgggccugggccgagccccag..............................    2    0    s17

....................aggcucgggcugggccccggg......................................................................    1    0    s02
....................agUcucgggcugggccccggg......................................................................    1    1    s02
...........................................................ccgggccugggccgagcccc................................    1    0    s02
...........................................................ccgggccugggccgagcccca...............................    1    0    s02

....................aggcucgggcugggccccggg......................................................................    2    0    s15
...........................................................ccgggccugggccgagcccca...............................    2    0    s15

....................aggcucgggcugggccccg........................................................................    1    0    s04
....................aggcucgggcugggccccgg.......................................................................    1    0    s04
....................aggcucgggcugggccccggg......................................................................    1    0    s04
...........................................................ccgggccugggccgagccccaU..............................    1    1    s04
...........................................................ccgggccugggccgagccccag..............................    1    0    s04

....................aggcucgggcugggcccc.........................................................................    1    0    s13
....................aggcucgggcugggccccggg......................................................................    6    0    s13
....................aggcucgggcugggccccgggU.....................................................................    2    1    s13
...........................................................ccgggccugggccgagccc.................................    1    0    s13
...........................................................ccgggccugggccgagcccc................................    2    0    s13
...........................................................ccgggccugggccgagccccag..............................    1    0    s13

....................aggcucgggcugggcccc.........................................................................    1    0    s01
...........................................................ccgggccugggccgagccc.................................    2    0    s01
...........................................................ccgggccugggccgagcccc................................    1    0    s01
...........................................................ccgggccugggccgagcccca...............................    1    0    s01
...........................................................ccgggccugggccgagccccaU..............................    1    1    s01
............................................................cgggccugggccgagcccca...............................    1    0    s01

....................aggcucgggcugggccccgg.......................................................................    1    0    s12
....................aggcucgggcugggccccggg......................................................................    2    0    s12
...........................................................ccgggccugggccgagcccc................................    1    0    s12
...........................................................ccUggccugggccgagcccc................................    1    1    s12
...........................................................ccgggccugggccgagccccag..............................    2    0    s12
...........................................................ccgggccugggccgagccccaU..............................    1    1    s12

....................aggcucgggcugggccccgg.......................................................................    1    0    s03
....................aggcCcgggcugggccccggg......................................................................    1    1    s03
....................aggcucgggcugggccccggg......................................................................    1    0    s03
...........................................................ccgggccugggccgagccc.................................    1    0    s03
...........................................................ccgggccugggccgagccccU...............................    1    1    s03
...........................................................ccgggccugggccgagccccag..............................    2    0    s03
...........................................................cUgggccugggccgagccccaga.............................    1    1    s03

....................aggcucgggcugggccccg........................................................................    1    0    s08
....................aggcucgggcugggccccgg.......................................................................    2    0    s08
....................aggcucgggcugggccccggg......................................................................    5    0    s08
....................aggcucgggcugggccccgggU.....................................................................    1    1    s08
.......................cucgggcugggccccgggcggg..................................................................    1    0    s08
..........................................................cccgggccugggccgagcccc................................    1    0    s08
...........................................................ccgggccugggccgagcccc................................    3    0    s08
...........................................................ccgggccugggccgagccccU...............................    1    1    s08

....................aggcCcgggcugggccccggg......................................................................    1    1    s10
....................aggcucgggcugggccccggg......................................................................    1    0    s10
....................aggcucgggcugggccccgggU.....................................................................    1    1    s10
...........................................................ccgggccugggccgagcc..................................    1    0    s10
...........................................................ccgggccugggccgagcccc................................    1    0    s10
...........................................................ccgggccugggccgagcccca...............................    1    0    s10
...........................................................ccgggccugggccgagccccag..............................    1    0    s10

....................aggcucgggcugggccccggg......................................................................    2    0    s18
```

```
                    Mature                                      Star
agcucccucugggagcggucaggcucgggcugggccccgggcggguggcagcaccgcccccgggccugggccgagccccagagcggcggaucucugcggaucugcggagug
...........................................................ccgggccugggccgagccc.................................   1   0   s18
...........................................................ccgggccugggccgagcccc................................   1   0   s18
...........................................................ccgggccugggccgagcccca...............................   1   0   s18
...........................................................ccgggccugggccgagccccag..............................   1   0   s18

.......................cucgggcugggccccgggcgg...................................................................   1   0   s11
...........................................................ccgggccugggccgagcccca...............................   2   0   s11
...........................................................ccgggccugggccgagccccag..............................   1   0   s11

....................aggcucgggcugggccccgg.......................................................................   1   0   s21
....................aggcucgggcugggccccggg......................................................................   1   0   s21
....................aggcucgggcugggccccgggU.....................................................................   1   1   s21
...........................................................ccgggccugggccgagccc.................................   1   0   s21
...........................................................ccgggccugggccgagcccca...............................   2   0   s21
...........................................................ccgggccugggccgagccccag..............................   1   0   s21

....................aggcucgggcugggccccg........................................................................   1   0   s24
....................aggcucgggcugggccccggg......................................................................   1   0   s24

....................aggcucgggcugggcccc.........................................................................   1   0   s23
....................aggcucgggcugggccccggg......................................................................   1   0   s23
....................aggcucgggcugggccccgggU.....................................................................   1   1   s23
...........................................................ccgggccugggccgagcccc................................   1   0   s23
...........................................................ccgUgccugggccgagccccag..............................   1   1   s23
...........................................................ccgggccugggccgagccccag..............................   1   0   s23
...........................................................ccgggccugggccgagccccaga.............................   1   0   s23
............................................................cgggccugggccgagccc.................................   1   0   s23

...........................................................ccgggccugggccgagccc.................................   1   0   s20
...........................................................ccgggccugggccgagcccca...............................   3   0   s20
```
