# Supplementary material for: The Identification of Circulating MiRNA in Bovine Serum and Their Potential as Novel Biomarkers of Early Mycobacterium avium subsp paratuberculosis Infection
Source: PLoS One. 2015 Jul 28;10(7):e0134310. doi: 10.1371/journal.pone.0134310 (PMC4517789; doi:10.1371/journal.pone.0134310)
Supplement: S1 File — (ZIP) [file pone.0134310.s008.zip › novel_pdfs/3_18831.pdf]

Provisional ID	: 3_18831
Score total	: 95.2
Score for star read(s)	: 3.9
Score for read counts	: 84.2
Score for mfe	: 2.4
Score for randfold	: 1.6
Score for cons. seed	: 3
Total read count	: 177
Mature read count	: 143
Loop read count	: 0
Star read count	: 34

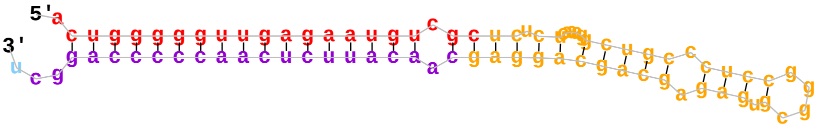

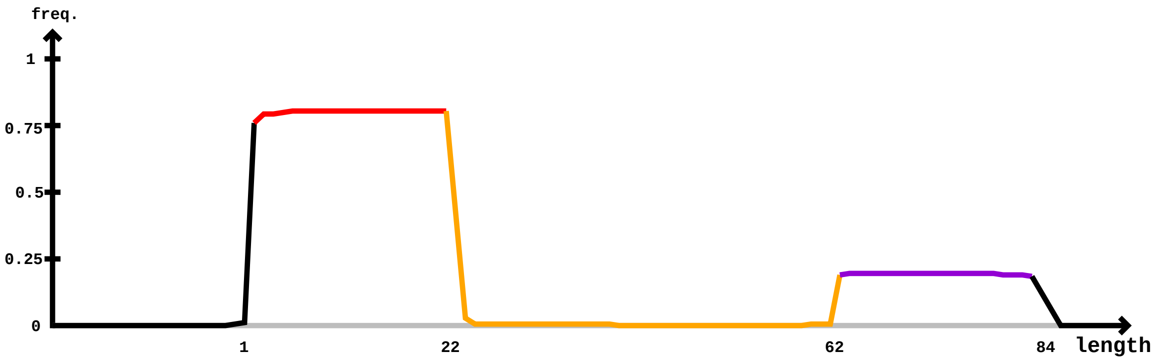


```
                          Mature                                                       Star
5'- gggucuggggccagggcugacuggggguugagaaugucgcucucucaaaggcugcccuccgggcgugagagcagcaggagcaacauucucaacccccaggcuccaaagag   -3'    obs
    gggucuggggccagggcugacuggggguugagaaugucgcucucucaaaggcugcccuccgggcgugagagcagcaggagcaacauucucaacccccaggcuccaaagag          exp
    .((((...)))).((((...(((((((((((((((((.((((.((.....(((((.((((....).))).))))))))))).)))))))))))))))))))))).......    reads    mm    sample
    ...................acuggggguugagaaugucgc......................................................................    6        0     s03
    ...................acuggggguugagaaugucgcuc....................................................................    1        0     s03
    ................................................................................caacauucucaacccccaggc.........    5        0     s03
    ................................................................................cGacauucucaacccccaggcu........    1        1     s03

    ...................acGggggguugagaaugucgc......................................................................    1        1     s18
    ...................acuUgggguugagaaugucgc......................................................................    1        1     s18
    ...................acuggggguugagaaugucgc......................................................................    6        0     s18
    ...................acuggggguugagaaugucgcA.....................................................................    1        1     s18
    ...................acuggggguugagaaugucgcu.....................................................................    1        0     s18
    ................................................................................caacauucucaacccccaggc.........    2        0     s18
    ................................................................................cGacauucucaacccccaggc.........    1        1     s18

    .................ugacuggggguugagaaugucgc......................................................................    1        0     s10
    ...................acuggggguugagaaugucgc......................................................................    6        0     s10
    ...................acugCggguugagaaugucgc......................................................................    1        1     s10
    ...................acCggggguugagaaugucgc......................................................................    1        1     s10
    ...................acAggggguugagaaugucgc......................................................................    1        1     s10
    ...................acuggggguugagaaugucgcu.....................................................................    1        0     s10
    ...................acuggggguugagaaugucgcuA....................................................................    1        1     s10
    ................................................................................caacauucucaacccccaggc.........    1        0     s10
    ................................................................................caacCuucucaacccccaggc.........    1        1     s10

    ...................acAggggguugagaaugucgc......................................................................    2        1     s08
    ...................acuUgggguugagaaugucgc......................................................................    1        1     s08
    ...................acGggggguugagaaugucgc......................................................................    1        1     s08
    ...................acCggggguugagaaugucgc......................................................................    1        1     s08
    ...................acuggggguugagaaugucgc......................................................................    1        0     s08
    ....................cuggggguugagaaugucgc......................................................................    3        0     s08
    ................................................................................caacauucucaacccccaggc.........    1        0     s08

    ...................acuggggguugagaaugucgc......................................................................    3        0     s20

    ...................acuCgggguugagaaugucgc......................................................................    1        1     s21
```

Mature   Star

```
gggucuggggccagggcugacuggggguugagaaugucgcucucucaaaggcugcccuccgggcgugagagcagcaggagcaacauucucaacccccaggcuccaaagag
....................cuggggguugCgaaugucgc......................................................................  1  1  s21

...................acuggggguugagaaugucgc......................................................................  7  0  s23
...................acuUgggguugagaaugucgc......................................................................  1  1  s23
................................................................................caacauucucaacccccaggcuG.......  1  1  s23

...................acugAggguugagaaugucgc......................................................................  1  1  s24
...................acCggggguugagaaugucgc......................................................................  1  1  s24
...................acuggggguugagaaugucgc......................................................................  5  0  s24
.......................gggguugagaaugucgc......................................................................  1  0  s24
................................................................................caacauucucaacccccaggcuG.......  1  1  s24

..................gacuggggguugagaaugucgc......................................................................  1  0  s11
...................acuggggguugagaaugucgc......................................................................  5  0  s11
...................acuggggguugagaaugucgcu.....................................................................  1  0  s11
................................................................................caacauucucaacccccagg..........  1  0  s11

...................acAggggguugagaaugucgc......................................................................  1  1  s14
...................acGggggguugagaaugucgc......................................................................  1  1  s14
...................acuggggguugagaaugucgc......................................................................  3  0  s14
...................acuggggguugagaaugucgcuU....................................................................  1  1  s14
................................................................................caacauucucaacccccaggc.........  1  0  s14

...................acuggggguugagaaugucgc......................................................................  2  0  s07

...................acuggggguugagaaugucgc......................................................................  3  0  s09
...................acuUgggguugagaaugucgc......................................................................  1  1  s09
................................................................................caacauucucaacccccaggc.........  1  0  s09
................................................................................caacauucucaacccccaggA.........  1  1  s09

...................acuggggguugagaaugucgc......................................................................  4  0  s19
...................acCggggguugagaaugucgc......................................................................  2  1  s19
................................................................................caacauucucaacccccaggc.........  4  0  s19
................................................................................caacGuucucaacccccaggc.........  1  1  s19

...................acuUgggguugagaaugucgc......................................................................  1  1  s01
...................acuggggguugagaaugucgc......................................................................  4  0  s01
................................................................................caacauucucaacccccaggc.........  3  0  s01
................................................................................cGacauucucaacccccaggc.........  1  1  s01

...................acuggggguugagaaugucgc......................................................................  2  0  s13

...................acuggggguugagaaugucgc......................................................................  2  0  s04
...................acGggggguugagaaugucgc......................................................................  1  1  s04
...................acuggggguugagaaugucgcu.....................................................................  1  0  s04
................................................................................caacauucucaacccccaggc.........  1  0  s04

...................acuggggguugagaaugucgc......................................................................  3  0  s15
...................acAggggguugagaaugucgc......................................................................  1  1  s15
...................acCggggguugagaaugucgc......................................................................  1  1  s15

...................acuggggguugagaaugucgc......................................................................  5  0  s17
...................acuUgggguugagaaugucgc......................................................................  1  1  s17
...................acGggggguugagaaugucgc......................................................................  1  1  s17
....................cuggCgguugagaaugucgc......................................................................  1  1  s17

...................acuggggguugagaaugucgc......................................................................  4  0  s02
................................................................................caacauucucaacccccaggc.........  1  0  s02

...................acCggggguugagaaugucgc......................................................................  1  1  s05
...................acuggggguugagaaugucgc......................................................................  12  0  s05
...................acuUgggguugagaaugucgc......................................................................  1  1  s05
...................acGggggguugagaaugucgc......................................................................  1  1  s05
................................................................................caacCuucucaacccccaggc.........  1  1  s05
................................................................................caacauucucaacccccaggc.........  2  0  s05

...................acGggggguugagaaugucgc......................................................................  1  1  s16
...................acuggggguugagaaugucgc......................................................................  2  0  s16
...................acuggggguugagaaugucgcuc....................................................................  1  0  s16

...................acCggggguugagaaugucgc......................................................................  1  1  s22
```

```
                   Mature                                                     Star
gggucuggggccagggcugacuggggguugagaaugucgcucucucaaaggcugcccuccgggcgugagagcagcaggagcaacauucucaacccccaggcuccaaagag
...................acuggggguugagaaugucgc......................................................................    3    0    s22
....................cuggggguugagaaugucgc......................................................................    1    0    s22
.......................gggguugagaaugucgcucucucaaaggcugccc.....................................................    1    0    s22

...................acuggggguugagaaugucgc......................................................................    1    0    s06
.............................................................................gagcaacauucucaaccccc.............    1    0    s06

...................acGggggguugagaaugucgc......................................................................    1    1    s12
...................acuggggguugagaaugucgc......................................................................    4    0    s12
...................acuUgggguugagaaugucgc......................................................................    1    1    s12
................................................................................caacauucucaacccccaggc.........    1    0    s12
.................................................................................aacauucucaacccccaggcu........    1    0    s12
```
