# Supplementary material for: The Identification of Circulating MiRNA in Bovine Serum and Their Potential as Novel Biomarkers of Early Mycobacterium avium subsp paratuberculosis Infection
Source: PLoS One. 2015 Jul 28;10(7):e0134310. doi: 10.1371/journal.pone.0134310 (PMC4517789; doi:10.1371/journal.pone.0134310)
Supplement: S1 File — (ZIP) [file pone.0134310.s008.zip › novel_pdfs/26_16101.pdf]

Provisional ID	:	26_16101
Score total	:	99
Score for star read(s)	:	3.9
Score for read counts	:	88.8
Score for mfe	:	1.7
Score for randfold	:	1.6
Score for cons. seed	:	3
Total read count	:	186
Mature read count	:	180
Loop read count	:	0
Star read count	:	6

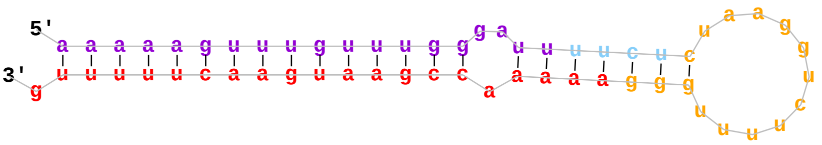

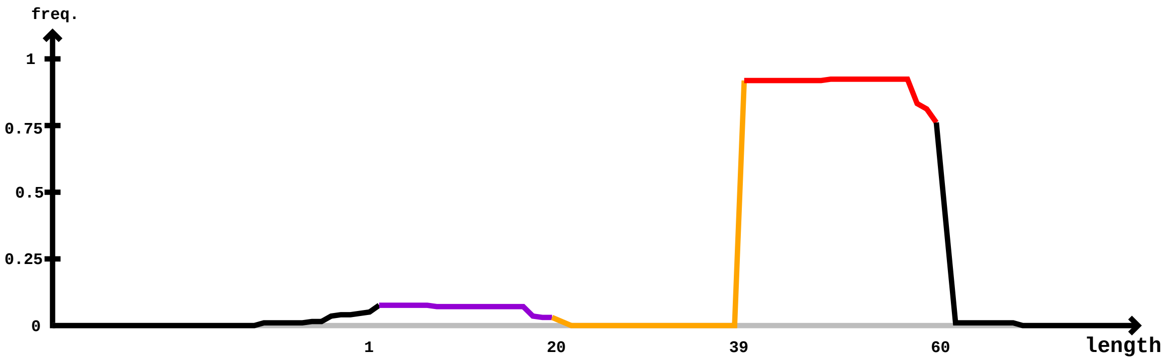


```
                                                Star                                 Mature
5'- ggaagauaucaggcaaagauauuggauuggccaaaaaguuuguuugggauuuucucuaaggucuuuugggaaaaaccgaaugaacuuuuugaccaauccuacacagcagca  -3'   obs
    ggaagauaucaggcaaagauauuggauuggccaaaaaguuuguuugggauuuucucuaaggucuuuugggaaaaaccgaaugaacuuuuugaccaauccuacacagcagca        exp
    ....((((((.......))))))(((((((.((((((((((((((((..(((((((...........))))))).)))))))))))))))).)))))))...........   reads    mm    sample
    ................................aaaaaguuuguuugggUuu............................................................   1        1     s12
    ......................................................................aaaaacGgaaugaacuuu.......................   5        1     s12
    ......................................................................aaaaacGgaaugaacuuuuu.....................   2        1     s12
    ......................................................................aaaaacGgaaugaacuuuuug....................   9        1     s12
    ......................................................................aaaaaccgaaugaacuuuuug....................   1        0     s12
    ......................................................................aaaaaccgaaugaacuuuuuga...................   1        0     s12
    ......................................................................aaaaacGgaaugaacuuuuuga...................   2        1     s12
    ......................................................................aaaaacUgaaugaacuuuuuga...................   1        1     s12

    ......................................................................aaaaacGgaaugaacuuu.......................   1        1     s02
    ......................................................................aaaaacUgaaugaacuuu.......................   1        1     s02
    ......................................................................aaaaacGgaaugaacuuuuug....................   5        1     s02
    ......................................................................aaaaacUgaaugaacuuuuug....................   1        1     s02
    ......................................................................aaaaacGgaaugaacuuuuuga...................   1        1     s02

    ...........................uggccaaaaaguuuguuCggg...............................................................   1        1     s17
    ......................................................................aaaaacGgaaugaacuuuuug....................   4        1     s17

    ......................................................................aaaaacGgaaugaacuuuuu.....................   1        1     s16

    ......................................................................aaaaaccgaaugaacuuuuug....................   2        0     s05
    ......................................................................aaaaacGgaaugaacuuuuug....................   9        1     s05
    ......................................................................aaaaacGgaaugaacuuuuuga...................   2        1     s05
    ......................................................................aaaaCccgaaugaacuuuuuga...................   3        1     s05

    ...............................caaaaaguuuguuugggUuu............................................................   1        1     s06
    ......................................................................aaaaacGgaaugaacuuu.......................   1        1     s06
    ......................................................................aaaaacGgaaugaacuuuuu.....................   1        1     s06
    ......................................................................aaaaacGgaaugaacuuuuug....................   4        1     s06

    ............................ggccaaaaaguuCguuuggg...............................................................   1        1     s22
    ......................................................................aaaaacGgaaugaacuuuuug....................   1        1     s22

    ................................aaaaaguuuguuugggUuu............................................................   2        1     s01
```

```
                                Star                  Mature
ggaagauaucaggcaaagauauuggauuggccaaaaaguuuguuugggauuuucucuaaggucuuuugggaaaaaccgaaugaacuuuuugaccaauccuacacagcagca
......................................................................aaaaacGgaaugaacuuuuu.....................   1    1   s01
......................................................................aaaaacUgaaugaacuuuuug....................   2    1   s01
......................................................................aaaaacGgaaugaacuuuuug....................   3    1   s01
......................................................................aaaaacGgaaugaacuuuuuga...................   2    1   s01

......................................................................aaaaacGgaaugaacuuuuug....................   6    1   s04

................................aaaaaguuuguuugggUuu............................................................   1    1   s13
......................................................................aaaaacGgaaugaacuuuuug....................   1    1   s13

...........................uggccaaaaaguuuguuCggg...............................................................   1    1   s19
................................aaaaaguuuguuugggauu............................................................   1    0   s19
......................................................................aaaaacGgaaugaacuuuuu.....................   1    1   s19
......................................................................aaaaacGgaaugaacuuuuug....................   20   1   s19
......................................................................aaaaaccgaaugaacuuuuug....................   1    0   s19
......................................................................aaaaacGgaaugaacuuuuuga...................   3    1   s19
......................................................................aaaaaccgaaugaacuuuuuga...................   1    0   s19
......................................................................aaaaacUgaaugaacuuuuuga...................   1    1   s19
...............................................................................augaacuuuuugaccaaucc............   1    0   s19

......................................................................aaaaacGgaaugaacuu........................   1    1   s09
......................................................................aaaaacGgaaugaacuuuuug....................   1    1   s09

......................................................................aaaaacGgaaugaacuu........................   1    1   s07
......................................................................aaaaacGgaaugaacuuuuug....................   4    1   s07
......................................................................aaaaacGgaaugaacuuuuuga...................   2    1   s07

......................................................................aaaaacGgaaugaacuuuuug....................   1    1   s14

..............................ccaaaaaguuuguuugggC..............................................................   1    1   s11
......................................................................aaaaacUgaaugaacuu........................   1    1   s11
......................................................................aaaaacGgaaugaacuu........................   1    1   s11
......................................................................aaaaacGgaaugaacuuu.......................   1    1   s11
......................................................................aaaaacGgaaugaacuuuuug....................   7    1   s11
......................................................................aaaaacUgaaugaacuuuuuga...................   1    1   s11
......................................................................aaaaacGgaaugaacuuuuuga...................   3    1   s11
......................................................................aaaaCccgaaugaacuuuuuga...................   1    1   s11

......................................................................aaaaacGgaaugaacuuuuu.....................   1    1   s20
......................................................................aaaaacGgaaugaacuuuuug....................   4    1   s20

......................................................................aaaaacGgaaugaacuuuuug....................   1    1   s23

....................auAggauuggccaaaaag.........................................................................   1    1   s21
......................................................................aaaaacGgaaugaacuu........................   1    1   s21
......................................................................aaaaacUgaaugaacuuuuu.....................   1    1   s21
......................................................................aaaaacGgaaugaacuuuuug....................   2    1   s21
......................................................................aaaaacGgaaugaacuuuuuga...................   1    1   s21
......................................................................aaaaCccgaaugaacuuuuuga...................   1    1   s21

.........................Guuggccaaaaaguuuguuuggg...............................................................   1    1   s24
......................................................................aaaaacGgaaugaacuuu.......................   1    1   s24
......................................................................aaaaacGgaaugaacuuuuu.....................   1    1   s24
......................................................................aaaaacUgaaugaacuuuuug....................   1    1   s24
......................................................................aaaaacGgaaugaacuuuuug....................   8    1   s24
......................................................................aaaaacGgaaugaacuuuuuga...................   3    1   s24
......................................................................aaaaCccgaaugaacuuuuuga...................   1    1   s24

...........................uggccaaaaaguuuguuCggg...............................................................   1    1   s10
......................................................................aaaaacGgaaugaacuu........................   1    1   s10
......................................................................aaaaacGgaaugaacuuu.......................   1    1   s10
......................................................................aaaaacGgaaugaacuuuuu.....................   1    1   s10
......................................................................aaaaaccgaaCgaacuuuuug....................   1    1   s10
......................................................................aaaaacGgaaugaacuuuuug....................   2    1   s10
......................................................................aaaaacGgaaugaacuuuuuga...................   1    1   s10

...........................uggccaaaaaguuuguuCggg...............................................................   1    1   s08
......................................................................aaaaacGgaaugaacuu........................   3    1   s08
......................................................................aaaaacGgaaugaacuuu.......................   1    1   s08
......................................................................aaaaacGgaaugaacuuuuug....................   3    1   s08
```

```
                               Star                                Mature
ggaagauaucaggcaaagauauuggauuggccaaaaaguuuguuugggauuuucucuaaggucuuuugggaaaaaccgaaugaacuuuuugaccaauccuacacagcagca
......................................................................aaaaacGgaaugaacuuuuuga...................    1    1    s08

......................................................................aaaaacUgaaugaacuuuuug....................    1    1    s18
......................................................................aaaaacUgaaugaacuuuuuga...................    1    1    s18
......................................................................aaaaacGgaaugaacuuuuuga...................    1    1    s18
......................................................................aaaaCccgaaugaacuuuuuga...................    1    1    s18
......................................................................aaaaaccAaaugaacuuuuugaccaaucc............    1    1    s18

....................auuggauuggccaaaaaguuuguuuggg...............................................................    1    0    s03
......................................................................aaaaacGgaaugaacuuu.......................    1    1    s03
......................................................................aaaaacGgaaugaacuuuuug....................    5    1    s03
......................................................................aaaaacUgaaugaacuuuuug....................    1    1    s03
......................................................................aaaaacGgaaugaacuuuuuga...................    2    1    s03
```
