# Supplementary material for: The Identification of Circulating MiRNA in Bovine Serum and Their Potential as Novel Biomarkers of Early Mycobacterium avium subsp paratuberculosis Infection
Source: PLoS One. 2015 Jul 28;10(7):e0134310. doi: 10.1371/journal.pone.0134310 (PMC4517789; doi:10.1371/journal.pone.0134310)
Supplement: S1 File — (ZIP) [file pone.0134310.s008.zip › novel_pdfs/29_18010.pdf]

Provisional ID	:	29_18010
Score total	:	5.9
Score for star read(s)	:	-1.3
Score for read counts	:	0
Score for mfe	:	2.6
Score for randfold	:	1.6
Score for cons. seed	:	3
Total read count	:	218
Mature read count	:	217
Loop read count	:	0
Star read count	:	1

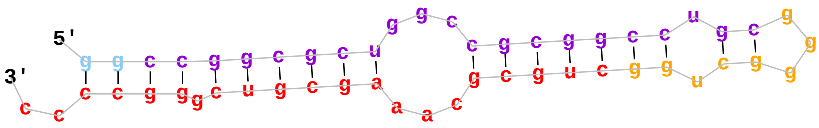

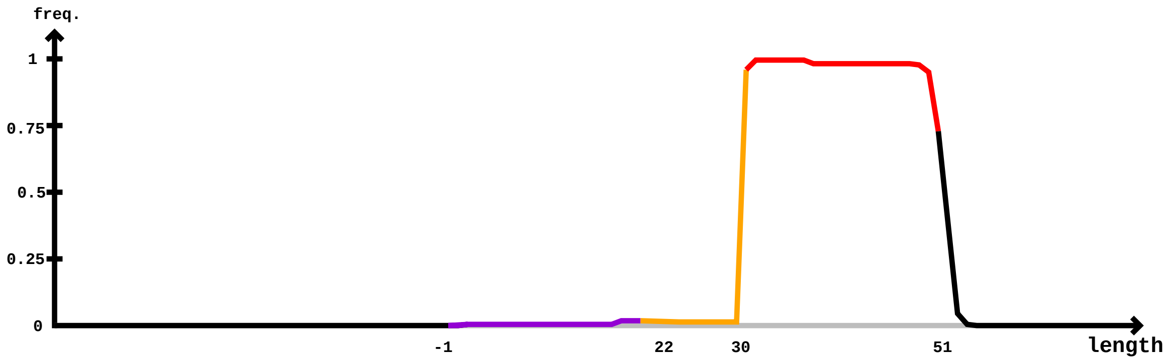


```
                                            Star                       Mature
5'- cuuccgggaacauaaaaagagggcggugcccgccccggcggccggcgcuggccgcggccugcggggcuggcugcgcaaagcgucgggccccggggggcggcguccacuagg   -3'    obs
    cuuccgggaacauaaaaagagggcggugcccgccccggcggccggcgcuggccgcggccugcggggcuggcugcgcaaagcgucgggccccggggggcggcguccacuagg          exp
    .................((.(((((.(((((..(((((.((((((((((...(((((((.((...)).)))))))...)))))).)))))))))))))).))))).))...   reads   mm   sample
    ......................................................................cugcgcaaagcgucgggcc......................   1       0    s22
    ......................................................................cuUcgcaaagcgucgggcc......................   1       1    s22
    ......................................................................cugcgcaaagcgucgggccc.....................   1       0    s22
    ......................................................................cGgcgcaaagcgucgggcccc....................   1       1    s22
    ......................................................................cugcgcaaagcgucgggcccc....................   1       0    s22
    ......................................................................cugcgcaaagcgucgggccccg...................   4       0    s22
    ......................................................................cugcgcaaagcgucgggccccU...................   1       1    s22
    .......................................................................ugcgcaaagcgucgggccccgg..................   1       0    s22

    ......................................................................cugcgcaaagcgucgggccc.....................   1       0    s06
    ......................................................................cugcgcaaagcgucgggcccc....................   2       0    s06
    ......................................................................cGgcgcaaagcgucgggccccg...................   1       1    s06
    ......................................................................cugcgcaaagcgucgggccccg...................   1       0    s06

    .........................................................ccugcggggcuggcugcgca..................................   1       0    s05
    ......................................................................cugcgcaaagcgucgggccc.....................   2       0    s05
    ......................................................................cugcgcaaagcgucgggcccc....................   1       0    s05
    ......................................................................cGgcgcaaagcgucgggcccc....................   1       1    s05
    ......................................................................cugcgcaaagcgucgggccccU...................   1       1    s05
    ......................................................................cugcgcaaagcgucgggccccg...................   1       0    s05

    .........................................................ccugcggggcuggcugcgca..................................   1       0    s16
    ......................................................................cugcgcaaagcgucgggccc.....................   2       0    s16
    ......................................................................cugcgcaaagcgucgggcccc....................   2       0    s16
    ......................................................................cugcgcaaagcgucgggccccg...................   4       0    s16
    .......................................................................ugcgGaaagcgucgggcccc....................   1       1    s16

    ......................................................................cugcgcaaagcgucgggcc......................   1       0    s02
    ......................................................................cugcgcaaagcgucgggcccc....................   2       0    s02
    ......................................................................cugcgcaaagcgucgggcccU....................   2       1    s02
    ......................................................................cugGgcaaagcgucgggccccg...................   1       1    s02
    ......................................................................cugcgcaaagcgucgggccccg...................   2       0    s02

    ......................................................................cugcgcaaagcgucgggc.......................   1       0    s17
```

```
                                         Star                 Mature
cuuccgggaacauaaaaagagggcggugcccgccccggcggccggcgcuggccgcggccugcggggcuggcugcgcaaagcgucgggccccggggggcggcguccacuagg
......................................................................cugcgcaaagcgucgggccc.....................	1	0	s17
......................................................................cugcgcaaagcgucgggccA.....................	1	1	s17
......................................................................cCgcgcaaagcgucgggccc.....................	1	1	s17
......................................................................cugcgcaaagcgucgggcccc....................	2	0	s17
......................................................................cugGgcaaagcgucgggcccc....................	1	1	s17
......................................................................cugcgcaCagcgucgggcccc....................	1	1	s17

......................................................................cugcgcaaagcgucgggccc.....................	2	0	s15
......................................................................cugcgcaaagcgucgggcccc....................	3	0	s15
......................................................................cCgcgcaaagcgucgggccccg...................	1	1	s15
......................................................................cugcgcaaagcgucgggccccU...................	1	1	s15

......................................................................cugcgcaaagcgucgggcc......................	1	0	s04
......................................................................cugcgcaaagcgucgggccc.....................	1	0	s04
......................................................................cugcgcaaagcgucgUgcccc....................	1	1	s04
......................................................................cugcgcaaagcgucgggcccc....................	3	0	s04
......................................................................cugcgcaaagcgucgggccccg...................	1	0	s04
......................................................................cugcCcaaagcgucgggccccg...................	1	1	s04
.......................................................................ugcgcaaagcgucgggccccU...................	2	1	s04

.........................................ccggAgcuggccgcggccugc.................................................	1	1	s13
......................................................................cugcgcaaagcgucgggccU.....................	1	1	s13
......................................................................cugcgcaaagcgucgggcccc....................	1	0	s13

......................................................................cugcgcaaagcgucgggccc.....................	2	0	s01
......................................................................cugcgcaaagcgucgggcccc....................	6	0	s01
......................................................................cugcgcaaagcgucgggccccg...................	4	0	s01
......................................................................cugcgcaaagcgucgggccccA...................	1	1	s01
......................................................................cugcgcaaagcgucgggccccU...................	3	1	s01

.........................................................ccCgcggggcuggcugcgca..................................	1	1	s12
......................................................................cugcgcaaagcgucgggccc.....................	4	0	s12
......................................................................cugcgcaaagcgucgggcccc....................	3	0	s12
......................................................................cugcgcaaagcgucgggccccg...................	2	0	s12
......................................................................cugcgcaaagcgucgggccccgU..................	1	1	s12
......................................................................cugcgcaaagcgucgggccccgA..................	1	1	s12
......................................................................cugcgcaaagcgucgggccccgg..................	1	0	s12

......................................................................cugcgcaaagcgucgggcc......................	1	0	s07
......................................................................cugcgcaaagcgucgggcccc....................	4	0	s07

......................................................................cugcgcaaagcgucgggcccc....................	6	0	s14
......................................................................cugcgcaaagcgucgggGccc....................	1	1	s14
......................................................................cugcgcaaagcgucgggccccC...................	1	1	s14
......................................................................cugcgcaaagcgucgggccccg...................	1	0	s14
......................................................................cugcgcaaagcgucgggccccgC..................	1	1	s14

......................................................................cugcgcaaagcgucgggccc.....................	1	0	s19
......................................................................cugcgcaaagcgucgggcccc....................	1	0	s19
......................................................................cugcgcaaagcgucgggccccg...................	1	0	s19
......................................................................cugcgcaaagcgucgggccccgg..................	1	0	s19

......................................................................cugcgcaaagcgucgggccc.....................	2	0	s09
......................................................................cugcgcaaagcgucgggcccc....................	5	0	s09
......................................................................cugcgcaaagcgucgggccccg...................	1	0	s09

......................................................................cugcgcaaagcgucgggccc.....................	2	0	s11
......................................................................cugcCcaaagcgucgggcccc....................	1	1	s11
......................................................................cCgcgcaaagcgucgggcccc....................	1	1	s11
......................................................................cugcgcaaagcgucgggcccc....................	3	0	s11
......................................................................cugcgcaaagcgucgggccccg...................	2	0	s11

......................................................................cugcgcaaagcgucgggccc.....................	3	0	s24
......................................................................cugcgcaaagcgucgggcccc....................	2	0	s24
.......................................................................ugcgGaaagcgucgggccc.....................	1	1	s24
.......................................................................ugcgcaaagcgucgggccc.....................	1	0	s24
.......................................................................ugcgcaaagcgucgggccccg...................	1	0	s24

......................................................................cugcCcaaagcgucgggccc.....................	1	1	s23
......................................................................cugcgcaaagcgucgggcccc....................	3	0	s23
```

```
                                         Star                        Mature
cuuccgggaacauaaaaagagggcggugcccgccccggcggccggcgcuggccgcggccugcggggcuggcugcgcaaagcgucgggccccggggggcggcguccacuagg
......................................................................cugcgcaaagcgucgggccccg...................   1   0   s23

......................................................................cugcgcaaagcgucgggcc......................   1   0   s21
......................................................................cGgcgcaaagcgucgggccc.....................   1   1   s21
......................................................................cugcgcaaagcgucgggccc.....................   2   0   s21
......................................................................cugcgcaaagcgucgggcccA....................   1   1   s21
......................................................................cugcgcaaagcgucgggcccc....................   5   0   s21
......................................................................cugcgcaaagcgucgggccccg...................   1   0   s21
......................................................................cugcgcaaagcgucgggccccU...................   1   1   s21
......................................................................cugcgcaaagcgucgggccccgg..................   1   0   s21

......................................................................cugcgcaaagcgucgggcccc....................   2   0   s20
......................................................................cGgcgcaaagcgucgggcccc....................   1   1   s20
......................................................................cugcgcaaagcgucgggccccU...................   1   1   s20
......................................................................cugcCcaaagcgucgggccccg...................   1   1   s20
......................................................................cugcgcaaagcgucgggccccgg..................   1   0   s20

......................................................................cugcgcaaagcgucgggccc.....................   3   0   s03
......................................................................cCgcgcaaagcgucgggccc.....................   1   1   s03
......................................................................cugcgcaaagcgucgggcccc....................   6   0   s03
......................................................................cugcCcaaagcgucgggcccc....................   1   1   s03
......................................................................cCgcgcaaagcgucgggcccc....................   1   1   s03
......................................................................cugcgcaaagcgucgggccccg...................   5   0   s03

......................................................................cugcgcaaagcgucgggccc.....................   6   0   s08
......................................................................cugcgcaaagcgucgggcccc....................   6   0   s08
......................................................................cugcgcaaagcgucgggccccg...................   2   0   s08
......................................................................cugcgcaaagcgucgggccccgg..................   1   0   s08

......................................................................cugcgcaaagcgucgggccc.....................   3   0   s10
......................................................................cugcgcaaagcgucgggccA.....................   1   1   s10
......................................................................cugcgcaaagcgucgggcccc....................   7   0   s10
......................................................................cugcgcaaagcgucgggccccg...................   2   0   s10
......................................................................cugcgcaaagcgucgggccccggU.................   1   1   s10

......................................................................cugcgcaaagcgucgggccc.....................   2   0   s18
......................................................................cCgcgcaaagcgucgggcccc....................   1   1   s18
......................................................................cugcgcaaagcgucgggcccc....................   4   0   s18
......................................................................cugcgcaaagcgucgggccccU...................   1   1   s18
.......................................................................ugcgcaaagcgucgggccccg...................   1   0   s18
```
