# Supplementary material for: The Identification of Circulating MiRNA in Bovine Serum and Their Potential as Novel Biomarkers of Early Mycobacterium avium subsp paratuberculosis Infection
Source: PLoS One. 2015 Jul 28;10(7):e0134310. doi: 10.1371/journal.pone.0134310 (PMC4517789; doi:10.1371/journal.pone.0134310)
Supplement: S1 File — (ZIP) [file pone.0134310.s008.zip › novel_pdfs/25_15966.pdf]

| | | |
|---|---|---|
| Provisional ID | : | 25_15966 |
| Score total | : | 6.2 |
| Score for star read(s) | : | -1.3 |
| Score for read counts | : | 0 |
| Score for mfe | : | 2.9 |
| Score for randfold | : | 1.6 |
| Score for cons. seed | : | 3 |
| Total read count | : | 170 |
| Mature read count | : | 170 |
| Loop read count | : | 0 |
| Star read count | : | 0 |

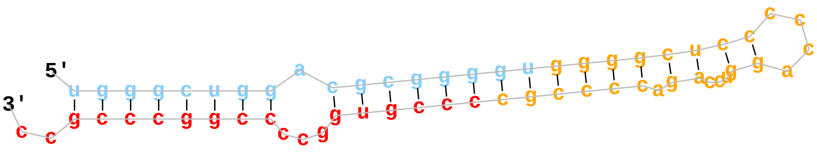


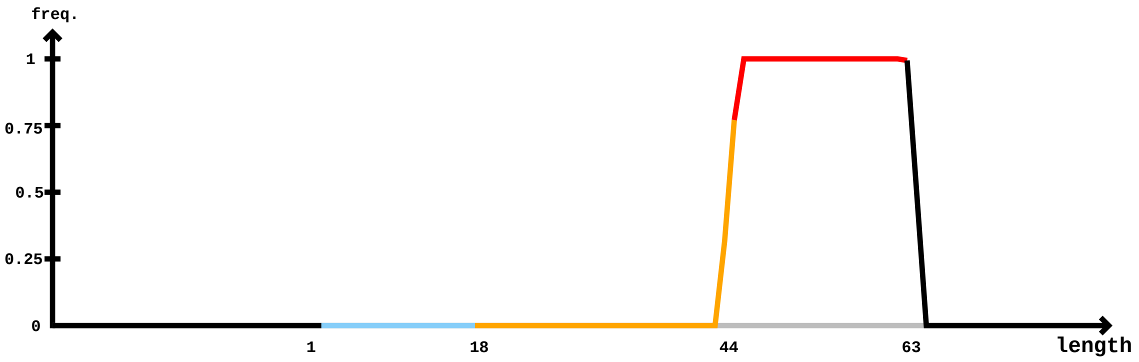


Star Mature

```
5'- cggcccguggagccggggggcgggucugggcuggacgcgggguggggcucccccagguccagaccccgccccguggccccggcccgccccgcccuccccgcuggaagg   -3'   exp
    (..((.((((....((((((((((..((((((((.((((((((((((((((....))...)).))))))))))))...)))))))).))))))))))))))).))..).    reads      mm         sample
    .....................................................................cccguggccccggcccgcc....................    2          0          s19

    ....................................................................Ucccguggccccggcccgcc....................    6          1          s09
    .....................................................................cccguggccccggcccgcc....................    2          0          s09

    ....................................................................Ucccguggccccggcccgcc....................    3          1          s07
    .....................................................................cccguggccccggcccgcc....................    3          0          s07
    ......................................................................ccguggccccggcccgcc....................    2          0          s07

    ....................................................................Ucccguggccccggcccgcc....................    1          1          s14
    .....................................................................cccguggccccggcccgcc....................    1          0          s14
    ......................................................................ccguggccccggcccgcc....................    1          0          s14

    ....................................................................Ucccguggccccggcccgcc....................    5          1          s12
    .....................................................................cccguggccccggcccgcc....................    4          0          s12
    ......................................................................ccCuggccccggcccgcc....................    1          1          s12

    .....................................................................cccguggccccggcccgcc....................    1          0          s02

    ....................................................................Ucccguggccccggcccgcc....................    2          1          s17
    .....................................................................cccguggccccggcccgcc....................    4          0          s17
    ......................................................................ccguggccccggcccgcc....................    3          0          s17

    ....................................................................Ucccguggccccggcccgcc....................    3          1          s06
    .....................................................................cccguggccccggcccgcc....................    5          0          s06
    ......................................................................ccUuggccccggcccgcc....................    2          1          s06
    ......................................................................ccguggccccggcccgcc....................    2          0          s06

    ....................................................................Ucccguggccccggcccgcc....................    4          1          s05
    ......................................................................ccguggccccggcccgcc....................    1          0          s05

    ....................................................................Ucccguggccccggcccgcc....................    1          1          s22
    .....................................................................cccguggccccggcccgcc....................    4          0          s22
    ......................................................................ccguggccccggcccgcc....................    2          0          s22
```

Star Mature

cggcccguggagccggggggcggguc**ugggcuggacgcggggu**ggggcucccccagguccagaccccgc**cccguggccccggcccgcc**ccgcccuccccgcuggaagg

| | | | |
|---|---|---|---|
| ....................................................................Ucccguggccccggcccgcc.................... | 1 | 1 | s16 |
| .....................................................................cccguggccccggcccgcc.................... | 2 | 0 | s16 |
| ......................................................................ccguggccccggcccgcc.................... | 3 | 0 | s16 |
| ....................................................................Ucccguggccccggcccgcc.................... | 1 | 1 | s01 |
| .....................................................................cccguggccccggcccgcc.................... | 1 | 0 | s01 |
| ......................................................................ccguggccccggcccgcc.................... | 1 | 0 | s01 |
| ....................................................................Ucccguggccccggcccgcc.................... | 1 | 1 | s15 |
| .....................................................................cccguggccccggcccgcc.................... | 4 | 0 | s15 |
| ....................................................................Ucccguggccccggcccgcc.................... | 1 | 1 | s04 |
| .....................................................................cccguggccccggcccgcc.................... | 1 | 0 | s04 |
| .....................................................................cccUggccccggcccgcc.................... | 1 | 1 | s04 |
| ......................................................................ccUggccccggcccgcc.................... | 1 | 1 | s04 |
| ......................................................................ccguggccccggcccgcc.................... | 2 | 0 | s04 |
| ....................................................................Ucccguggccccggcccgcc.................... | 6 | 1 | s13 |
| .....................................................................cccguggccccggcccgcc.................... | 6 | 0 | s13 |
| ......................................................................ccguggccccggcccgcc.................... | 3 | 0 | s13 |
| ....................................................................Ucccguggccccggcccgcc.................... | 3 | 1 | s10 |
| .....................................................................cccguggccccggcccgcc.................... | 4 | 0 | s10 |
| ......................................................................ccguggccccggcccgcc.................... | 1 | 0 | s10 |
| ....................................................................Ucccguggccccggcccgcc.................... | 1 | 1 | s08 |
| .....................................................................cccguggccccggcccgc..................... | 1 | 0 | s08 |
| .....................................................................cccguggccccggcccgcc.................... | 4 | 0 | s08 |
| ......................................................................ccUggccccggcccgcc.................... | 2 | 1 | s08 |
| ......................................................................ccguggccccggcccgcc.................... | 3 | 0 | s08 |
| ....................................................................Ucccguggccccggcccgcc.................... | 6 | 1 | s18 |
| .....................................................................Uccguggccccggcccgcc.................... | 1 | 1 | s18 |
| .....................................................................cccguggccccggcccgcc.................... | 5 | 0 | s18 |
| ....................................................................Ucccguggccccggcccgcc.................... | 3 | 1 | s03 |
| .....................................................................cccguggccccggcccgcc.................... | 3 | 0 | s03 |
| .....................................................................cUcguggccccggcccgcc.................... | 1 | 1 | s03 |
| ......................................................................ccguggccccggcccgcc.................... | 1 | 0 | s03 |
| ....................................................................Ucccguggccccggcccgcc.................... | 2 | 1 | s11 |
| .....................................................................cccguggccccggcccgcc.................... | 1 | 0 | s11 |
| ......................................................................ccguggccccggcccgcc.................... | 1 | 0 | s11 |
| .....................................................................cccguggccccggcccgcc.................... | 1 | 0 | s20 |
| ......................................................................ccguCgccccggcccgcc.................... | 1 | 1 | s20 |
| ....................................................................Ucccguggccccggcccgcc.................... | 1 | 1 | s23 |
| .....................................................................cccguggccccggcccgcc.................... | 9 | 0 | s23 |
| ......................................................................ccguggccccggcccgcc.................... | 4 | 0 | s23 |
| ....................................................................Ucccguggccccggcccgcc.................... | 3 | 1 | s21 |
| .....................................................................cccguggccccggcccgcc.................... | 6 | 0 | s21 |
| ......................................................................ccguggccccggcccgcc.................... | 2 | 0 | s21 |
