# Supplementary material for: The Identification of Circulating MiRNA in Bovine Serum and Their Potential as Novel Biomarkers of Early Mycobacterium avium subsp paratuberculosis Infection
Source: PLoS One. 2015 Jul 28;10(7):e0134310. doi: 10.1371/journal.pone.0134310 (PMC4517789; doi:10.1371/journal.pone.0134310)
Supplement: S1 File — (ZIP) [file pone.0134310.s008.zip › novel_pdfs/18_8057.pdf]

Provisional ID	:	18_8057
Score total	:	4.2
Score for star read(s)	:	-1.3
Score for read counts	:	0
Score for mfe	:	0.9
Score for randfold	:	1.6
Score for cons. seed	:	3
Total read count	:	18
Mature read count	:	18
Loop read count	:	0
Star read count	:	0

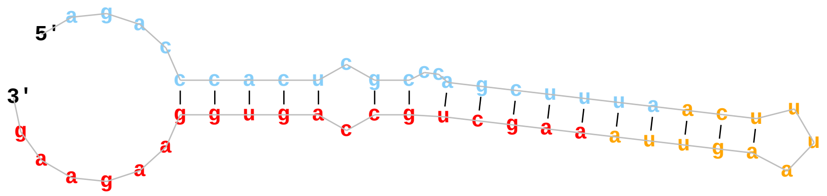

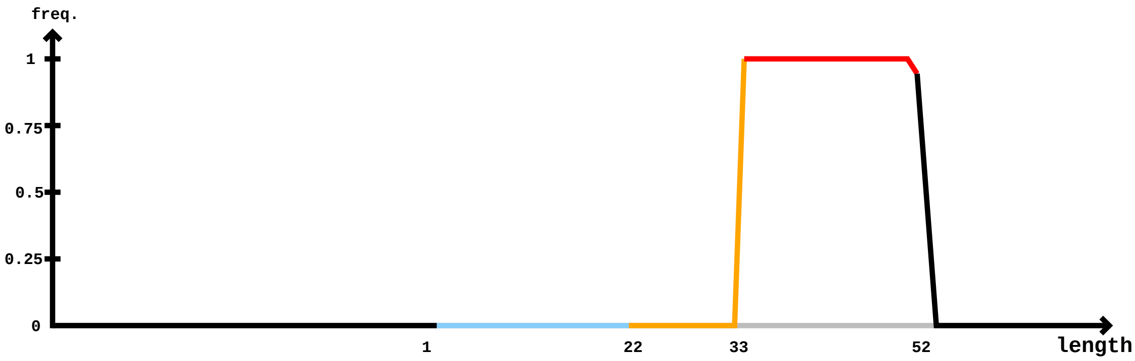

Star Mature

```
5'- uagcaagggccuggacacaugacccggcuccucugaucagacccacucgcccagcuuuaacuuuaaguuaaagcugccaguggaagaagcccaucuuagcaauugaug   -3'   exp
    ...((((((((.((........)).))).))).)).......(((((.((..((((((((((...))))))))))))).))))).........((((.........))))   reads   mm   sample
    ......................................................................aagcAgccaguggaagaa....................   1   1   s03

    ......................................................................aagcugccaguggaagaaC...................   1   1   s10

    ......................................................................aagcugccaguUgaagaag...................   1   1   s08
    ......................................................................aagcugccaguggaagaaC...................   1   1   s08

    ......................................................................aagcugccaguggaagaaC...................   1   1   s19

    ......................................................................aagcugccaguUgaagaag...................   1   1   s18

    ......................................................................aagcugccaguggaagaaC...................   1   1   s09

    ......................................................................aagcugccaguggaagaaC...................   1   1   s17

    ......................................................................aagcugccaguggaagaaC...................   1   1   s16

    ......................................................................aagcugccaguggaagaaC...................   1   1   s22

    ......................................................................aagcugccaguggaagaaC...................   1   1   s15

    ......................................................................aagcugccaguUgaagaag...................   1   1   s04

    ......................................................................aagcugccaguggaagaaC...................   1   1   s11
    ......................................................................aagcugccaguUgaagaag...................   2   1   s11

    ......................................................................aagcugccaguUgaagaag...................   1   1   s12

    ......................................................................aagcugccaguUgaagaag...................   1   1   s23

    ......................................................................aagcugccaguUgaagaag...................   1   1   s21
```
