# Supplementary material for: The Identification of Circulating MiRNA in Bovine Serum and Their Potential as Novel Biomarkers of Early Mycobacterium avium subsp paratuberculosis Infection
Source: PLoS One. 2015 Jul 28;10(7):e0134310. doi: 10.1371/journal.pone.0134310 (PMC4517789; doi:10.1371/journal.pone.0134310)
Supplement: S1 File — (ZIP) [file pone.0134310.s008.zip › novel_pdfs/9_24948.pdf]

Provisional ID	:	9_24948
Score total	:	5.1
Score for star read(s)	:	-1.3
Score for read counts	:	0
Score for mfe	:	1.8
Score for randfold	:	1.6
Score for cons. seed	:	3
Total read count	:	1369
Mature read count	:	1368
Loop read count	:	0
Star read count	:	1

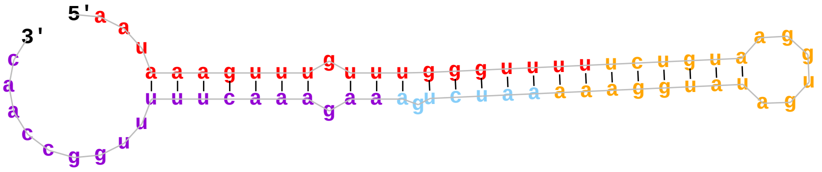

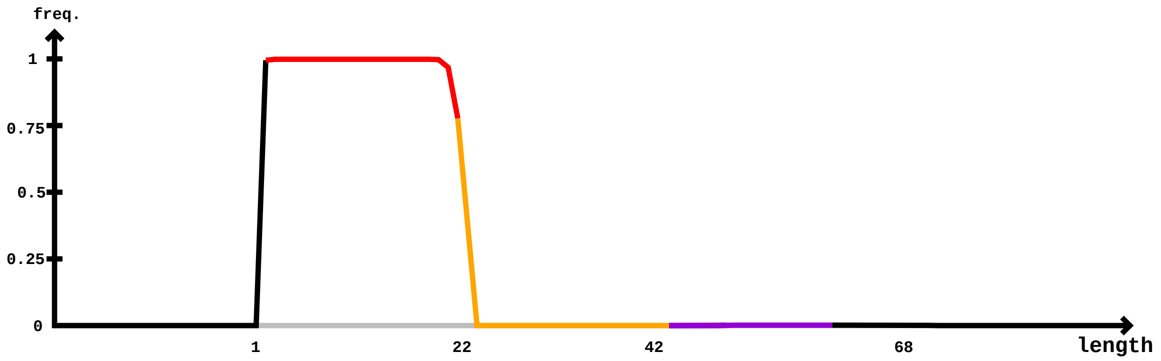

Mature Star

```
5'- aaauguuguauuggguugguaauaaaguuuguuuggguuuuucuguaaggugauauggaaaaaucugaaagaaacuuuuuggccaacccaauccaaaguuuaauuagcau  -3'   obs
    aaauguuguauuggguugguaauaaaguuuguuuggguuuuucuguaaggugauauggaaaaaucugaaagaaacuuuuuggccaacccaauccaaaguuuaauuagcau         exp
    ((((.(((.(((((((((((...(((((((.((((((((((((((((......))))))))))))).))).)))))))...))))))))))).))).)))).........    reads   mm   sample
    ....................aaAaaaguuuguuuggguu.......................................................................    6       1    s03
    ....................aaAaaaguuuguuuggguuu......................................................................    11      1    s03
    ....................aaAaaaguuuguuuggguuuu.....................................................................    25      1    s03
    ....................aaAaaaguuuguuuggguuuuu....................................................................    3       1    s03

    ....................aaAaaaguuuguuuggguu.......................................................................    1       1    s10
    ....................aaAaaaguuuguuuggguuu......................................................................    14      1    s10
    ....................aauaaaguuuguuuggguuuu.....................................................................    4       0    s10
    ....................aauaCaguuuguuuggguuuu.....................................................................    1       1    s10
    ....................aaAaaaguuuguuuggguuuu.....................................................................    103     1    s10
    ....................aauaaaguuuguuuggguuuuu....................................................................    1       0    s10
    ....................aaAaaaguuuguuuggguuuuu....................................................................    11      1    s10

    ....................aaAaaaguuuguuuggguu.......................................................................    1       1    s08
    ....................aaAaaaguuuguuuggguuu......................................................................    7       1    s08
    ....................aaAaaaguuuguuuggguuuu.....................................................................    7       1    s08
    ....................aaAaaaguuuguuuggguuuuu....................................................................    2       1    s08
    ....................aauaaaguuCguuuggguuuuu....................................................................    1       1    s08

    ....................aaAaaaguuuguuuggguu.......................................................................    1       1    s18
    ....................aaAaaaguuuguuuggguuu......................................................................    6       1    s18
    ....................aauaaaguuuguuuggguuuu.....................................................................    1       0    s18
    ....................aaAaaaguuuguuuggguuuu.....................................................................    17      1    s18
    ....................aauaCaguuuguuuggguuuu.....................................................................    1       1    s18
    ....................aaAaaaguuuguuuggguuuuu....................................................................    2       1    s18

    ....................aaAaaaguuuguuuggguuu......................................................................    11      1    s11
    ....................aUuaaaguuuguuuggguuu......................................................................    1       1    s11
    ....................aaAaaaguuuguuuggguuuu.....................................................................    39      1    s11
    ....................aaAaaaguuuguuuggguuuuu....................................................................    5       1    s11

    ....................aaAaaaguuuguuuggguuu......................................................................    9       1    s20
    ....................aaAaaaguuuguuuggguuuu.....................................................................    25      1    s20
```

```
                    Mature                                              Star
aaauguuguauuggguugguaauaaaguuuguuuggguuuuucuguaaggugauauggaaaaaucugaaagaaacuuuuuggccaacccaauccaaaguuuaauuagcau
....................aaAaaaguuuguuuggguuuuu....................................................................   4    1    s20

....................aaAaaaguuuguuuggguuu......................................................................   1    1    s21
....................aaAaaaguuuguuuggguuuu.....................................................................   11   1    s21

....................aaAaaaguuuguuuggguu.......................................................................   3    1    s24
....................aaGaaaguuuguuuggguuu......................................................................   1    1    s24
....................aaAaaaguuuguuuggguuu......................................................................   18   1    s24
....................aauaaaguuuguuuggguuu......................................................................   1    0    s24
....................aauaaaguuuguuuggguuuu.....................................................................   1    0    s24
....................aaAaaaguuuguuuggguuuu.....................................................................   58   1    s24
....................aauaCaguuuguuuggguuuu.....................................................................   1    1    s24
....................aaAaaaguuuguuuggguuuuu....................................................................   14   1    s24
....................aaGaaaguuuguuuggguuuuu....................................................................   1    1    s24
....................................................................aagaaacuuuuuggccaac.......................   1    0    s24

....................aaAaaaguuuguuuggguu.......................................................................   1    1    s23
....................aaAaaaguuuguuuggguuu......................................................................   8    1    s23
....................aaAaaaguuuguuuggguuuu.....................................................................   23   1    s23
....................aauaaaguuuguuuggguuuu.....................................................................   1    0    s23
....................aaAaaaguuuguuuggguuuuu....................................................................   5    1    s23

....................aaAaaaguuuguuuggguuu......................................................................   14   1    s07
....................aauaaaguuuguuuggguuuu.....................................................................   1    0    s07
....................aaAaaaguuuguuuggguuuu.....................................................................   24   1    s07
....................aaAaaaguuuguuuggguuuuu....................................................................   1    1    s07

....................aaAaaaguuuguuuggguu.......................................................................   2    1    s14
....................aaAaaaguuuguuuggguuu......................................................................   2    1    s14
....................aauaCaguuuguuuggguuu......................................................................   1    1    s14
....................aaAaaaguuuguuuggguuuu.....................................................................   13   1    s14
....................aauaaaguuuguuuggguuuu.....................................................................   2    0    s14
....................aaAaaaguuuguuuggguuuuu....................................................................   2    1    s14

....................aaAaaaguuuguuuggguu.......................................................................   3    1    s19
....................aauaaaguuuguuuggguu.......................................................................   1    0    s19
....................aaAaaaguuuguuuggguuu......................................................................   28   1    s19
....................aauaaaguuuguuuggguuu......................................................................   1    0    s19
....................aaAaaaguuuguuuggguuuu.....................................................................   77   1    s19
....................aauaaaguuuguuuggguuuu.....................................................................   2    0    s19
....................aauaaaguuuguuuggguuuuu....................................................................   1    0    s19
....................aaAaaaguuuguuuggguuuuu....................................................................   12   1    s19

....................aaAaaaguuuguuuggguuu......................................................................   6    1    s09
....................aaAaaaguuuguuuggguuuu.....................................................................   30   1    s09
....................aauaaaguuuguuuggguuuu.....................................................................   3    0    s09
....................aaAaaaguuuguuuggguuuuu....................................................................   2    1    s09

....................aaAaaaguuuguuuggguuu......................................................................   17   1    s02
....................aauaaaguuuguuuggguuu......................................................................   1    0    s02
....................aauaCaguuuguuuggguuuu.....................................................................   1    1    s02
....................aaAaaaguuuguuuggguuuu.....................................................................   59   1    s02
....................aauaaaguuuguuuggguuuu.....................................................................   3    0    s02
....................aaAaaaguuuguuuggguuuuu....................................................................   5    1    s02
....................aauaaaguuuguuuggguuuuu....................................................................   1    0    s02

....................aaAaaaguuuguuugggu........................................................................   1    1    s17
....................aaAaaaguuuguuuggguu.......................................................................   3    1    s17
....................aaAaaaguuuguuuggguuu......................................................................   13   1    s17
....................aaAaaaguuuguuuggguuuu.....................................................................   46   1    s17
....................aauaaaguuuguuuggguuuu.....................................................................   1    0    s17
....................aauaaaguuuguuuggguuuuu....................................................................   1    0    s17
....................aaAaaaguuuguuuggguuuuu....................................................................   3    1    s17

....................aaAaaaguuuguuuggguu.......................................................................   4    1    s05
....................aaAaaaguuuguuuggguuu......................................................................   19   1    s05
....................aaAaaaguuuguuuggguuuu.....................................................................   90   1    s05
....................aauaaaguuuguuuggguuuu.....................................................................   1    0    s05
....................aGuaaaguuuguuuggguuuu.....................................................................   1    1    s05
....................aauaCaguuuguuuggguuuuu....................................................................   1    1    s05
```

Mature   Star

```
aaauguuguauuggguugguaauaaaguuuguuuggguuuuucuguaaggugauauggaaaaaucugaaagaaacuuuuuggccaacccaauccaaaguuuaauuagcau
....................aaAaaaguuuguuuggguuuuu....................................................................   18   1   s05

....................aaAaaaguuuguuuggguuu......................................................................   5    1   s06
....................aauaaaguuuguuuggguuuu.....................................................................   1    0   s06
....................aaAaaaguuuguuuggguuuu.....................................................................   31   1   s06
....................aaCaaaguuuguuuggguuuu.....................................................................   1    1   s06
....................aaAaaaguuuguuuggguuuuu....................................................................   6    1   s06

....................aaAaaaguuuguuuggguu.......................................................................   1    1   s16
....................aaAaaaguuuguuuggguuu......................................................................   2    1   s16
....................aaAaaaguuuguuuggguuuu.....................................................................   10   1   s16
....................aaAaaaguuuguuuggguuuuu....................................................................   2    1   s16

....................aaAaaaguuuguuugggu........................................................................   1    1   s22
....................aaAaaaguuuguuuggguu.......................................................................   5    1   s22
....................aaAaaaguuuguuuggguuu......................................................................   13   1   s22
....................aauaaaguuuguuuggguuuu.....................................................................   3    0   s22
....................aaAaaaguuuguuuggguuuu.....................................................................   45   1   s22
....................aauaaaguuuguuuggguuuuu....................................................................   1    0   s22
....................aaAaaaguuuguuuggguuuuu....................................................................   3    1   s22

....................aaAaaaguuuguuuggguu.......................................................................   2    1   s01
....................aaAaaaguuuguuuggguuu......................................................................   13   1   s01
....................aauaCaguuuguuuggguuuu.....................................................................   1    1   s01
....................aaAaaaguuuguuuggguuuu.....................................................................   25   1   s01
....................aUuaaaguuuguuuggguuuu.....................................................................   1    1   s01
....................aaAaaaguuuguuuggguuuuu....................................................................   5    1   s01
.....................aAaaaguuuguuuggguuu......................................................................   2    1   s01
.....................................................................Ugaaacuuuuuggccaaccca....................   1    1   s01

....................aauaaaguuuguuuggguuu......................................................................   1    0   s04
....................aaAaaaguuuguuuggguuu......................................................................   5    1   s04
....................aauaaaguuuguuuggguuuu.....................................................................   1    0   s04
....................aaAaaaguuuguuuggguuuu.....................................................................   15   1   s04
....................aauaaaguuuguuuggguuuuu....................................................................   1    0   s04
....................aaAaaaguuuguuuggguuuuu....................................................................   2    1   s04

....................aaAaaaguuuguuuggguu.......................................................................   2    1   s13
....................aaAaaaguuuguuuggguuu......................................................................   4    1   s13
....................aauaCaguuuguuuggguuuu.....................................................................   1    1   s13
....................aaAaaaguuuguuuggguuuu.....................................................................   21   1   s13
....................aaAaaaguuuguuuggguuuuu....................................................................   5    1   s13
.....................aAaaaguuuguuuggguuu......................................................................   1    1   s13
.....................auaaaguuCguuuggguuuu.....................................................................   1    1   s13

....................aaAaaaguuuguuuggguu.......................................................................   1    1   s15
....................aaAaaaguuuguuuggguuu......................................................................   3    1   s15
....................aaAaaaguuuguuuggguuuu.....................................................................   24   1   s15
....................aaAaaaguuuguuuggguuuuu....................................................................   2    1   s15

....................aaAaaaguuuguuuggguu.......................................................................   3    1   s12
....................aaAaaaguuuguuuggguuu......................................................................   22   1   s12
....................aauaaaguuuguuuggguuuu.....................................................................   1    0   s12
....................aaAaaaguuuguuuggguuuu.....................................................................   73   1   s12
....................aaAaaaguuuguuuggguuuuu....................................................................   14   1   s12
.....................aAaaaguuuguuuggguuu......................................................................   1    1   s12
```
