# Supplementary material for: The Identification of Circulating MiRNA in Bovine Serum and Their Potential as Novel Biomarkers of Early Mycobacterium avium subsp paratuberculosis Infection
Source: PLoS One. 2015 Jul 28;10(7):e0134310. doi: 10.1371/journal.pone.0134310 (PMC4517789; doi:10.1371/journal.pone.0134310)
Supplement: S1 File — (ZIP) [file pone.0134310.s008.zip › novel_pdfs/10_1480.pdf]

Provisional ID	:	10_1480
Score total	:	38.7
Score for star read(s)	:	3.9
Score for read counts	:	31.7
Score for mfe	:	2.1
Score for randfold	:	1.6
Score for cons. seed	:	-0.6
Total read count	:	74
Mature read count	:	54
Loop read count	:	0
Star read count	:	20

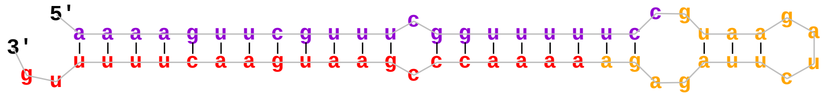

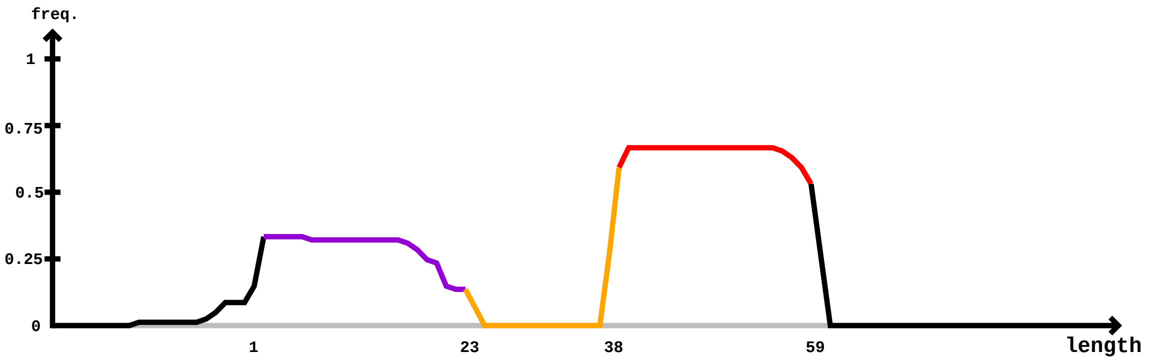

Star Mature

```
5'- accauauauuggguuggccaaaaaguucguuucgguuuuuccguaagaucuuagagaaaaacccgaaugaacuuuuuggugagcccaguaauucaccuguguaaagugu   -3'   obs
    accauauauuggguuggccaaaaaguucguuucgguuuuuccguaagaucuuagagaaaaacccgaaugaacuuuuuggugagcccaguaauucaccuguguaaagugu         exp
    ......(((((((((.((((((((((((((((.((((((((..(((....)))..)))))))).)))))))))))))))))).))))))))))...(((.........))).   reads   mm   sample
    ................gccaaaaaguucguuuGgguu........................................................................   1       1    s19
    ....................aaaaguucguuucgguuuuu.....................................................................   1       0    s19
    ....................aaaaguucguuucgguuuuucc...................................................................   1       0    s19
    ....................aaaaCuucguuucgguuuuucc...................................................................   1       1    s19
    ........................................................aaaaacccgaaCgaacuuuu.................................   1       1    s19
    ........................................................aaaaacccgaaugaacuuuuG................................   1       1    s19
    ........................................................aaaaCcccgaaugaacuuuuug...............................   1       1    s19
    ........................................................aaaaacccgaaCgaacuuuuug...............................   3       1    s19
    ........................................................aaaaacccgaaugaacuuuuug...............................   1       0    s19
    .........................................................aaaacccgaaugaacuuuu.................................   1       0    s19
    .........................................................aaaacccgaaugaacuuuuug...............................   4       0    s19
    .........................................................aaaacccgaaCgaacuuuuug...............................   2       1    s19

    ........................................................aaaaacccgaaugaacuuuuugA..............................   1       1    s09
    ........................................................aaaGcccgaaugaacuuuuu.................................   1       1    s09

    ...............ggccaaaaaguucguuuGgg..........................................................................   1       1    s22
    ........................................................aaaaacccgaaCgaacuuuuu................................   1       1    s22
    ..........................................................aaacccgaaCgaacuuuuug...............................   1       1    s22
    ..........................................................aaacccgaaugaacuuuuug...............................   2       0    s22
    ..........................................................aaacUcgaaugaacuuuuug...............................   1       1    s22
    ..........................................................aaacccgaaCgaacuuuuugg..............................   2       1    s22

    ..............uggccaaaaaguucguuuGgguu........................................................................   1       1    s06
    ................gccaaaaaguucguuuGggu.........................................................................   1       1    s06
    .........................................................aaaacccgaaugaacuuuuug...............................   1       0    s06

    ........................................................aaaaacccgaaugaacuuuuug...............................   2       0    s05
    .........................................................aaaacccgaaugaacuuuuugA..............................   3       1    s05

    ...................aaaaaguucguuucgguuuuu.....................................................................   1       0    s02
    ....................aaaaguucguuucgguuuuu.....................................................................   1       0    s02
    ....................aaaaguucguuucgguuuuucc...................................................................   2       0    s02
```

```
                    Star                                 Mature
accauauauuggguuggccaaaaaguucguuucgguuuuuccguaagaucuuagagaaaaacccgaaugaacuuuuuggugagcccaguaauucaccuguguaaagugu

...................aaaaaguucguuucgguuuuu.....................................................................    1    0    s13
....................aaaaguucguuucgguuuuucc...................................................................    3    0    s13

.........................................................aaaacccgaaugaacuuuuug...............................    1    0    s04
.........................................................aaaacccgaaGgaacuuuuug...............................    1    1    s04

........................................................aaaaacccgaaugaacuuuuG................................    1    1    s15

........................................................aaaaacccgaaugaacuCu..................................    1    1    s01

.........................................................aaaacccgaaugaacuuu..................................    1    0    s12

........................................................aaaaacccgaaCgaacuuuu.................................    1    1    s03
........................................................aaaaacccgaaCgaacuuuuugg..............................    1    1    s03
.........................................................aaaacccgaaugaacuuuuug...............................    1    0    s03

.......aGuggguuggccaaaaag....................................................................................    1    1    s08
.........................................................aaaacccgaaugaacuuuuuU...............................    1    1    s08

........................................................aaaaCcccgaaugaacuu...................................    1    1    s10
........................................................aaaaacccgaaugaacuuuuGg...............................    1    1    s10
........................................................aaaaCcccgaaugaacuuuuug...............................    1    1    s10
.........................................................aaaacccgaaugaacuuuuug...............................    1    0    s10

...................aaaaaguucguuucgguuuu......................................................................    1    0    s18
....................aCaaguucguuucgguuuu......................................................................    1    1    s18
....................aaaaguucguuucgguuuuucc...................................................................    2    0    s18
.........................................................aaaacccgaaugaacuuuuugA..............................    1    1    s18

...............ggccaaaaaguucguuuGggu.........................................................................    1    1    s11
...................aaaaaguucguuucgguuu.......................................................................    1    0    s11
....................aaaaguucguuucgguuuu......................................................................    1    0    s11
....................aaaaguucguuucgguuuuucc...................................................................    2    0    s11
........................................................aaaaacccgaaugaacuuuuG................................    1    1    s11
.........................................................aaaacccgaaugaacuuuuug...............................    2    0    s11
.........................................................aaaacccgaaugaacuuuuugA..............................    1    1    s11

................gccaaaaaguucguuuGgguu........................................................................    1    1    s24
........................................................aaaaacccgaaugaacuuuuug...............................    1    0    s24
........................................................aaaaacccgaaugaacuuuuugA..............................    2    1    s24
.........................................................aaaacccgaaugaacuuuuug...............................    2    0    s24
.........................................................aaaacccgaaugaacuuuuugA..............................    1    1    s24

.........................................................aaaacccgaaugaacuuuuugA..............................    1    1    s21

...................aaaaaguucguuucgguuuu......................................................................    1    0    s23
```
