# Supplementary material for: The Identification of Circulating MiRNA in Bovine Serum and Their Potential as Novel Biomarkers of Early Mycobacterium avium subsp paratuberculosis Infection
Source: PLoS One. 2015 Jul 28;10(7):e0134310. doi: 10.1371/journal.pone.0134310 (PMC4517789; doi:10.1371/journal.pone.0134310)
Supplement: S1 File — (ZIP) [file pone.0134310.s008.zip › novel_pdfs/4_20156.pdf]

Provisional ID	:	4_20156
Score total	:	102.5
Score for star read(s)	:	3.9
Score for read counts	:	95.5
Score for mfe	:	2.1
Score for randfold	:	1.6
Score for cons. seed	:	-0.6
Total read count	:	199
Mature read count	:	198
Loop read count	:	0
Star read count	:	1

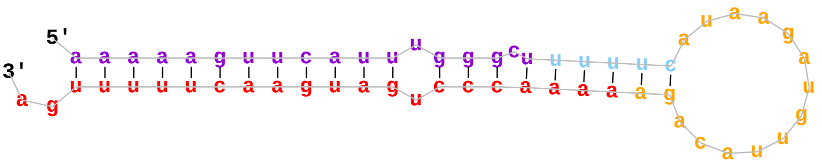

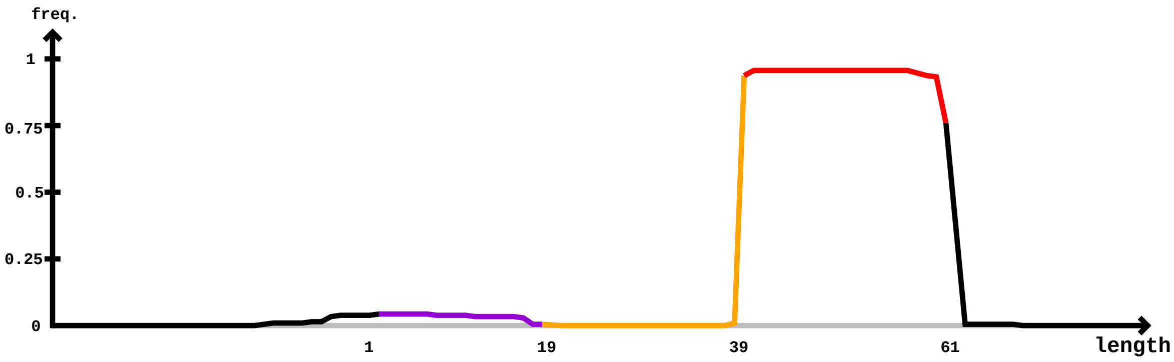


```
                                             Star                                 Mature
5'- ggcucaggaauuugccuuguauuggguuggccaaaaaguucauuugggcuuuuucauaagauguuacagaaaaacccugaugaacuuuuugaucaacccaauaaaaggccua   -3'    obs
    ggcucaggaauuugccuuguauuggguuggccaaaaaguucauuugggcuuuuucauaagauguuacagaaaaacccugaugaacuuuuugaucaacccaauaaaaggccua          exp
    .............(((((.(((((((((((.(((((((((((((.(((.((((((.............))))))))).))))))))))))).))))))))))).)))))...    reads    mm    sample
    ......................................................................aaaacccugaugaacuuuuug.....................    1        0     s18
    ......................................................................aaaacccugaugaacuuuuuga....................    10       0     s18
    ......................................................................aaUacccugaugaacuuuuuga....................    1        1     s18
    ......................................................................aaaaAccugaugaacuuuuuga....................    2        1     s18

    .....................................................................aaaaacccugaugaacuuuuug.....................    1        0     s10
    ......................................................................aaaacccugaugaacuuuuug.....................    6        0     s10
    ......................................................................aaUacccugaugaacuuuuuga....................    1        1     s10
    ......................................................................aaaacccugaugaacuuuuuga....................    15       0     s10
    ......................................................................aaaaAccugaugaacuuuuuga....................    3        1     s10
    .......................................................................aaacccAgaugaacuuuuug.....................    1        1     s10
    .......................................................................aaacccugaugaacuuuuuga....................    1        0     s10

    ....................aGuggguuggccaaaaag..........................................................................    1        1     s08
    ......................................................................aaaacccugaugaacuuuuug.....................    1        0     s08
    ......................................................................aaaaGccugaugaacuuuuuga....................    1        1     s08

    ......................................................................aaaacccugaugaacuuuuug.....................    1        0     s03
    ......................................................................aaUacccugaugaacuuuuuga....................    1        1     s03
    ......................................................................aaaacccugaugaacuuuuuga....................    6        0     s03
    ......................................................................aaaacccugaugaacuuuuugaucaaccc.............    1        0     s03

    ......................................................................aaaacccugaugaacuuuuug.....................    4        0     s20
    ......................................................................aaaacccugaugaacuuuuuga....................    8        0     s20
    ......................................................................aaGacccugaugaacuuuuuga....................    1        1     s20
    ......................................................................aaaaAccugaugaacuuuuuga....................    1        1     s20

    .....................uuggguuggcUaaaaaguuca......................................................................    1        1     s24
    .........................guuCgccaaaaaguucauuugg.................................................................    1        1     s24
    ......................................................................aaaacccugaugaacuuuuu......................    1        0     s24
    ......................................................................aaaacccugaugaacuuuuug.....................    1        0     s24
    ......................................................................aaaacccugaugaacuuuuuga....................    7        0     s24
    ......................................................................aaaaAccugaugaacuuuuuga....................    1        1     s24
```

```
                                Star                                Mature
ggcucaggaauuugccuuguauuggguuggccaaaaaguucauuugggcuuuuucauaagauguuacagaaaaacccugaugaacuuuuugaucaacccaauaaaaggccua
......................................................................aUaacccugaugaacuuuuuga....................    1    1    s24

......................................................................aaaacccugaugaacuuuuuga....................    2    0    s23

......................................................................aaaacccugaugaacuuuu.......................    1    0    s11
......................................................................aaaacccugaugaacuuuuug.....................    3    0    s11
......................................................................aaaacccugaugaacuuuuuga....................    5    0    s11
......................................................................aaaaAccugaugaacuuuuuga....................    1    1    s11
......................................................................aaaaUccugaugaacuuuuuga....................    1    1    s11

......................................................................aaaacccugaugaacuuu........................    1    0    s09
......................................................................aaaacccugaugaacuuuuug.....................    2    0    s09
......................................................................aaaacccugaugaacuuuuuga....................    3    0    s09
.......................................................................aaacccAgaugaacuuuuuga....................    1    1    s09

......................................................................aaaacccugaugaacuuuu.......................    1    0    s19
......................................................................aaaacccugaugaacuuuuug.....................    1    0    s19
......................................................................aaaaUccugaugaacuuuuuga....................    1    1    s19
......................................................................aaaacccugaugaacuuuuuga....................    6    0    s19
......................................................................aaaaAccugaugaacuuuuuga....................    1    1    s19
.......................................................................aaacccAgaugaacuuuuug.....................    1    1    s19

......................................................................aaaacccugaugaacuuuuuga....................    2    0    s14

......................................................................aaaacccugaugaacuuuuuga....................    6    0    s07
......................................................................aaaaAccugaugaacuuuuuga....................    1    1    s07

...........................uggccaaaaaguucauCuggg................................................................    1    1    s12
......................................................................aaaacccugaugaacuuuuug.....................    1    0    s12
......................................................................aaUacccugaugaacuuuuuga....................    1    1    s12
......................................................................aaaacccugaugaacuuuuuga....................    6    0    s12
......................................................................aaaaUccugaugaacuuuuuga....................    1    1    s12

......................................................................aaaacccugaugaacuuuuug.....................    1    0    s01
......................................................................aaaacccugaugaacuuuuuga....................    6    0    s01
......................................................................aaaacccugaugaacCuuuuga....................    1    1    s01
......................................................................aaaaAccugaugaacuuuuuga....................    2    1    s01

......................................................................aaaacccugaugaacuuuuuga....................    1    0    s04

......................................................................aaaacccugaugaacuuuuuga....................    1    0    s13

......................................................................aaaacccugaugaacuuuuug.....................    2    0    s15
......................................................................aaaaAccugaugaacuuuuuga....................    1    1    s15
......................................................................aaaacccugaugaacuuuuuga....................    8    0    s15

......................................................................aaaacccugaugaacuuu........................    1    0    s17
......................................................................aaaacccugaugaacuCuuug.....................    1    1    s17
......................................................................aaaacccugaugaacuuuuuga....................    3    0    s17

......................................................................aaaaAccugaugaacuuuuuga....................    4    1    s02
......................................................................aaaaUccugaugaacuuuuuga....................    1    1    s02
......................................................................aaaacccugaugaacuuuuuga....................    1    0    s02

......................................................................aaaacccugaugaacuuuuuga....................    1    0    s06

............................ggccaaaaaguucGuuggg.................................................................    1    1    s22
......................................................................aaUacccugaugaacuuuuuga....................    1    1    s22
......................................................................aaaacccugaugaacuuuuuga....................    4    0    s22
......................................................................aaaaAccugaugaacuuuuuga....................    1    1    s22

................................aaaaaguucauuugggcu..............................................................    1    0    s16
......................................................................aaaaAccugaugaacuuuuuga....................    1    1    s16
......................................................................aaaacccugaugaacuuuuuga....................    1    0    s16

...........................uggccaaaaaguucauCuggg................................................................    3    1    s05
.....................................................................aaaaacccugaugaacuuuuug.....................    1    0    s05
......................................................................aaaacccugaugaacuuuuug.....................    6    0    s05
......................................................................aaUacccugaugaacuuuuug.....................    1    1    s05
......................................................................aaaacccugaugaacuuuuuga....................    20   0    s05
```

Star **Mature**

```
ggcucaggaauuugccuuguauuggguuggccaaaaaguucauuugggcuuuuucauaagauguuacagaaaaacccugaugaacuuuuugaucaacccaauaaaaggccua
.................................................................aaaaAccugaugaacuuuuuga....................    1    1    s05
```
