# Supplementary material for: The Identification of Circulating MiRNA in Bovine Serum and Their Potential as Novel Biomarkers of Early Mycobacterium avium subsp paratuberculosis Infection
Source: PLoS One. 2015 Jul 28;10(7):e0134310. doi: 10.1371/journal.pone.0134310 (PMC4517789; doi:10.1371/journal.pone.0134310)
Supplement: S1 File — (ZIP) [file pone.0134310.s008.zip › novel_pdfs/3_18437.pdf]

Provisional ID	:	3_18437
Score total	:	136.1
Score for star read(s)	:	3.9
Score for read counts	:	128.6
Score for mfe	:	2.6
Score for randfold	:	1.6
Score for cons. seed	:	-0.6
Total read count	:	264
Mature read count	:	250
Loop read count	:	1
Star read count	:	13

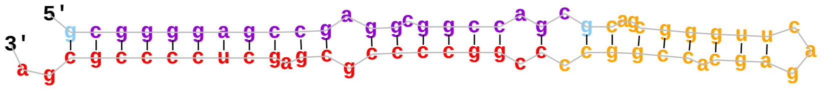

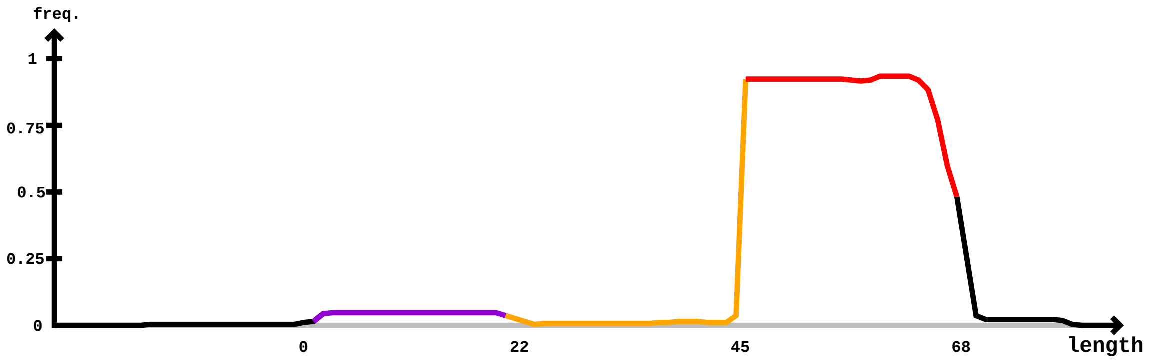


```
                              Star                                         Mature
5'- cugggcggagacaggcggcggccgcgcggggagccgaggcggccagcgcagcggguucagagcaccggccccggccccgcgagcuccccgcgagcugccgccacagacc   -3'   obs
    cugggcggagacaggcggcggccgcgcggggagccgaggcggccagcgcagcggguucagagcaccggccccggccccgcgagcuccccgcgagcugccgccacagacc         exp
    (((........)))((((((((..((((((((((((.((.((((.(.((..((((((...))).))))).).)))))).)).)))))))))).))))))))........   reads    mm    sample
    ..........................cggUgagccgaggcggccagc..............................................................   1        1     s01
    ......................................................................ccggccccgcgagcuccccg...................   1        0     s01
    ......................................................................ccggccccgcgagcuccccgc..................   2        0     s01
    ......................................................................ccggccccgcgagcuccccgcg.................   1        0     s01
    ......................................................................ccgUccccgcgagcuccccgcga................   1        1     s01
    ......................................................................ccggAcccgcgagcuccccgcga................   1        1     s01
    ......................................................................ccggccccgcgagcuccccgcga................   4        0     s01

    ..........................cggggagccgaggcggccag...............................................................   1        0     s04
    .....................................................................cccggccccgcgagcuccc.....................   1        0     s04
    .....................................................................cccggccccgcgagcuccccgcU.................   1        1     s04
    ......................................................................ccggccccgcgagcuccccg...................   1        0     s04
    ......................................................................cUggccccgcgagcuccccgc..................   1        1     s04
    ......................................................................ccggAcccgcgagcuccccgc..................   2        1     s04
    ......................................................................ccggccccgcgagcuccccgcC.................   1        1     s04
    ......................................................................ccggccccgcgagcuccccgcU.................   1        1     s04
    ......................................................................ccggccccgcgagcuccccgcga................   3        0     s04

    ......................................................................ccggccccgcgagcuccc.....................   1        0     s15
    ......................................................................ccggccccgcgagcucccc....................   1        0     s15
    ......................................................................ccggccccgcgagcuccccg...................   2        0     s15
    ......................................................................ccUgccccgcgagcuccccgc..................   1        1     s15
    ......................................................................ccggccccgcgagcuccccgc..................   1        0     s15
    ......................................................................ccggccccgcgagcuccccgcg.................   3        0     s15
    ......................................................................ccggAcccgcgagcuccccgcga................   1        1     s15
    ......................................................................ccggccccgcgagcuccccgcga................   4        0     s15
    ......................................................................ccggccccgcgagcuccccgcgaU...............   1        1     s15
    ......................................................................ccggccccgcgagcuccccgcgaC...............   1        1     s15
    ......................................................................ccggccccgcgagcuccccgcgagA..............   1        1     s15

    ..........................cggggagccgaggcggccCgc..............................................................   1        1     s13
    ......................................................................ccggAcccgcgagcuccccg...................   1        1     s13
    ......................................................................ccggccccgcgagcuccccg...................   1        0     s13
```

```
                          Star                                          Mature
cugggcggagacaggcggcggccgcgcggggagccgaggcggccagcgcagcggguucagagcaccggccccggccccgcgagcuccccgcgagcugccgccacagacc
......................................................................ccggccccgcgagcuccccgcC.................   1   1   s13
......................................................................ccggccccgcgagcuccccgcga................   2   0   s13
......................................................................ccggccccgcgagcuccccgcgag...............   1   0   s13

..........................cggggagccgCggcggccag...............................................................   1   1   s02
......................................................................ccggccccgcgagcuccccgc..................   1   0   s02
......................................................................ccggccccgcgagcuccccgcgag...............   2   0   s02

......................................................................ccggccccgcgagcuccccg...................   2   0   s17
......................................................................ccggUcccgcgagcuccccgcga................   1   1   s17
......................................................................ccggccccgcgagcuccccgcga................   5   0   s17
......................................................................ccggccccgcgagcuccGcgcga................   1   1   s17
......................................................................ccggccccgcgagcuccccgcgaA...............   1   1   s17

......................................................................ccggccccgcgagcuccccgc..................   3   0   s06
......................................................................ccggccccgcgagcuccccgU..................   1   1   s06
......................................................................ccggccccgcgagcuccccgcU.................   2   1   s06
......................................................................ccggccccgcgagcuccccgcg.................   2   0   s06
......................................................................ccggccccgcgagcuccccgcC.................   1   1   s06
......................................................................ccggccccgcgagcuccccgcga................   6   0   s06
......................................................................ccggccccgcgagcuccccgcgag...............   1   0   s06

.....................................................................cccggccccgcgagcuccccgcga................   1   0   s22
.....................................................................cccgCccccgcgagcuccccgcga................   1   1   s22
......................................................................ccggccccgcgagcucccc....................   2   0   s22
......................................................................ccgUccccgcgagcuccccg...................   1   1   s22
......................................................................ccggccccgcgagcuccccg...................   2   0   s22
......................................................................ccggccccgcgagcuccccgc..................   4   0   s22
......................................................................ccUgccccgcgagcuccccgc..................   1   1   s22
......................................................................ccggccccgcgagcuccccgcU.................   1   1   s22
......................................................................ccggccccgcgagcuccccgcg.................   5   0   s22
......................................................................ccggccccgcgagcuccccgcga................   3   0   s22
......................................................................ccggccccgcgagcuccccgcCa................   1   1   s22
......................................................................ccgUccccgcgagcuccccgcga................   1   1   s22
......................................................................ccggGcccgcgagcuccccgcga................   1   1   s22
......................................................................ccggccccgcgagcuccccgcgaA...............   1   1   s22
......................................................................ccggccccgcgagGuccccgcgag...............   1   1   s22
......................................................................ccggccccgcgagcuccccgcgagA..............   1   1   s22
....................................................................................uccccgcgagcugccgccac.....   2   0   s22
....................................................................................uccccgcgagcugccgccaca....   1   0   s22

......................................................................ccggccccgcgagcuccccgc..................   1   0   s05
......................................................................ccggccccgcgagcuccccgcgag...............   2   0   s05

..........................cggggagccgaggcggcca................................................................   1   0   s16
......................................................................ccggccccgcgagcuccccg...................   3   0   s16
......................................................................ccggccccgcgagcuccccgU..................   1   1   s16
......................................................................ccgUccccgcgagcuccccgc..................   1   1   s16
......................................................................ccggccccgcgagcuccccgcU.................   1   1   s16
......................................................................ccggccccgcgagcuccccgcga................   3   0   s16
......................................................................ccggccccgcgagcuccccgcgaA...............   1   1   s16
......................................................................ccggccccgcgagcuccccgcgag...............   2   0   s16

......................................................................ccggccccgcgagcuccccg...................   1   0   s12
......................................................................ccggAcccgcgagcuccccg...................   1   1   s12
......................................................................ccggccccgcgagcuccccgU..................   1   1   s12
......................................................................ccggccccgcgagcuccccgc..................   2   0   s12
......................................................................ccggccccgcgagcuccccgcga................   2   0   s12
......................................................................ccggccccgcgagcuccccgcgag...............   1   0   s12

........................cgcggggagccgaggUggccag...............................................................   1   1   s14
.........................gcggggagccgaggcggccag...............................................................   1   0   s14
......................................................................ccggccccgcgagcucccc....................   1   0   s14
......................................................................ccggccccgcgagcuccccg...................   1   0   s14
......................................................................ccggccccgcgagcuccccgc..................   1   0   s14
......................................................................ccggccccgcgagcuccccgcg.................   1   0   s14
......................................................................ccggccccgcgagcuccccgcU.................   1   1   s14
......................................................................ccggccccgcgagcuccccgcga................   4   0   s14
......................................................................ccgUccccgcgagcuccccgcga................   1   1   s14
......................................................................ccggccccgcgagcuccccgcgag...............   1   0   s14
```

```
                        Star                                          Mature
cugggcggagacaggcggcggccgcgcggggagccgaggcggccagcgcagcggguucagagcaccggccccggccccgcgagcuccccgcgagcugccgccacagacc
......................................................................ccggccccgcgagcuccccgcgaA...............  1  1  s14

........................cgcggggagccgaggcggcca................................................................  1  0  s07
......................................................................ccUgccccgcgagcuccc.....................  1  1  s07
......................................................................ccggAcccgcgagcuccccgc..................  1  1  s07
......................................................................ccggccccgcgagcuccccgcga................  1  0  s07
......................................................................ccggccccgcgagcuccccgcgag...............  2  0  s07
......................................................................ccggccccgcgagcuccccgcgagA..............  1  1  s07

........agaGaggcggcggccgcg...................................................................................  1  1  s09
..........................cggggagccgCggcggcca................................................................  1  1  s09
......................................................................ccggccccgcgagcuUcccg...................  1  1  s09
......................................................................ccggccccgcgagcuccccg...................  3  0  s09
......................................................................ccggccccgcgagcuccccgc..................  3  0  s09
......................................................................ccgUccccgcgagcuccccgc..................  1  1  s09
......................................................................ccggccccgcgagcuccccgcga................  2  0  s09
......................................................................ccgUccccgcgagcuccccgcga................  1  1  s09
......................................................................ccggccccgcgagcuccccgcgaA...............  1  1  s09

......................................................................ccggccccgcgagcuccccgcg.................  1  0  s19
......................................................................ccggUcccgcgagcuccccgcga................  1  1  s19

..........................cggggagccgaggcggccagc..............................................................  1  0  s20
......................................................................ccggccccgcgagcuccccg...................  1  0  s20
......................................................................ccggccccgcgagcuccccgc..................  2  0  s20
......................................................................ccggccccgcgagcuccccgcga................  1  0  s20
......................................................................ccggccccgcgagcuccccgcgag...............  1  0  s20

......................................................................ccggccccgcgagcuccccgcga................  1  0  s24
......................................................................ccgUccccgcgagcuccccgcga................  2  1  s24

..........................cggggagccgaggcggccagc..............................................................  1  0  s21
...........................ggggagccgaggcggccag...............................................................  1  0  s21
......................................................................ccggccccgcgagcucccc....................  1  0  s21
......................................................................ccggccccgcgagcuccccg...................  3  0  s21
......................................................................ccggccccgcgagcuccccgc..................  2  0  s21
......................................................................ccggAcccgcgagcuccccgcga................  1  1  s21
......................................................................ccggccccgcgagcuccccgcga................  4  0  s21
......................................................................ccggccccgcgagcuccccgcgag...............  1  0  s21

.................................................agcggguucagagcaccggccccggccccgcga...........................  1  0  s23
......................................................................ccggccccgcgagcuccc.....................  1  0  s23
......................................................................ccggccccgcgagcucccc....................  1  0  s23
......................................................................ccggccccgcgagcuccccgc..................  3  0  s23
......................................................................ccgUccccgcgagcuccccgc..................  1  1  s23
......................................................................ccggccccgcgagcuccccgcg.................  1  0  s23
......................................................................ccggccccgcgagcuccccgcC.................  2  1  s23
......................................................................ccggccccgcgagcuccccgcga................  6  0  s23
......................................................................ccggccccgcgagcuccccgcgag...............  1  0  s23

................................................cagcUgguucagagcacc...........................................  1  1  s11
.....................................................................cccggccccgcgagcuccccgcgaA...............  1  1  s11
......................................................................ccggccccgcgagcucccc....................  1  0  s11
......................................................................ccggccccgcgagcuccccg...................  1  0  s11
......................................................................ccggccccgcgagcuccccgc..................  2  0  s11
......................................................................ccggccccgcgagcuccccgcga................  8  0  s11

..........................cggggagccgaggcggccag...............................................................  1  0  s03
...............................................................accggccccggccccgcgagc.........................  1  0  s03
......................................................................ccggAcccgcgagcuccccg...................  1  1  s03
......................................................................ccggccccgcgagcuccccgc..................  1  0  s03
......................................................................ccggccccgcgagcuccccgcg.................  1  0  s03
......................................................................ccggccccgcgagcuccccgcga................  2  0  s03

.....................................................................cccgAccccgcgagcuccccgcga................  1  1  s18
......................................................................ccggccccgcgagcuccccg...................  2  0  s18
......................................................................ccggccccgcgagcuccccgc..................  2  0  s18
......................................................................ccggccccgcgagcuccccgcg.................  1  0  s18
......................................................................ccggccccgcgagcuccccgcga................  1  0  s18
......................................................................ccggccccgcgagcuccccgcgag...............  1  0  s18
```

Star **Mature**

cugggcggagacaggcggcggccgcgcggggagccgaggcggccagcgcagcggguucagagcaccggccccggccccgcgagcuccccgcgagcugccgccacagacc			
....uccccgcgagcugccgcca......	1	0	s18
....gcUccggccccggccccgcg............................	1	1	s10
....cccggccccgcgagcuccccgc..................	1	0	s10
....ccggccccgcgagcucccc....................	3	0	s10
....ccggAcccgcgagcuccccg...................	2	1	s10
....ccggccccgcgagcuccccgc..................	1	0	s10
....ccggAcccgcgagcuccccgc..................	2	1	s10
....ccggccccgcgagcuccccgcC.................	1	1	s10
....ccggccccgcgagcuccccgcg.................	1	0	s10
....ccggccccgcgagcuccccgcU.................	1	1	s10
....ccggAcccgcgagcuccccgcga................	2	1	s10
....ccUgccccgcgagcuccccgcga................	1	1	s10
....ccggccccgcgagcuccccgcga................	9	0	s10
....ccggccccgcgagcuccccgcgaA...............	1	1	s10
....ccggccccgcgagcuccccgcgagA..............	1	1	s10
....cuccccgcgagcugccgccac.....	1	0	s10
....uccccgcgagcugccgccac.....	1	0	s10
....ccggAcccgcgagcuccccgc..................	1	1	s08
....ccggccccgcgagcuccccgcC.................	1	1	s08
....ccggccccgcgagcuccccgcga................	4	0	s08
....ccggccccgcgagcuccccgcgaA...............	1	1	s08
