# Supplementary material for: The Identification of Circulating MiRNA in Bovine Serum and Their Potential as Novel Biomarkers of Early Mycobacterium avium subsp paratuberculosis Infection
Source: PLoS One. 2015 Jul 28;10(7):e0134310. doi: 10.1371/journal.pone.0134310 (PMC4517789; doi:10.1371/journal.pone.0134310)
Supplement: S1 File — (ZIP) [file pone.0134310.s008.zip › novel_pdfs/20_11724.pdf]

Provisional ID	: 20_11724
Score total	: 133.9
Score for star read(s)	: 3.9
Score for read counts	: 123.5
Score for mfe	: 1.9
Score for randfold	: 1.6
Score for cons. seed	: 3
Total read count	: 254
Mature read count	: 223
Loop read count	: 0
Star read count	: 31

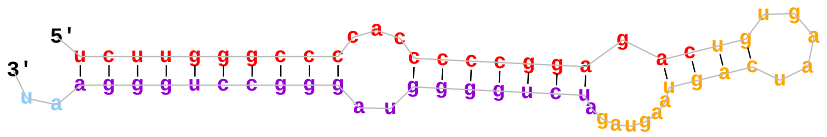

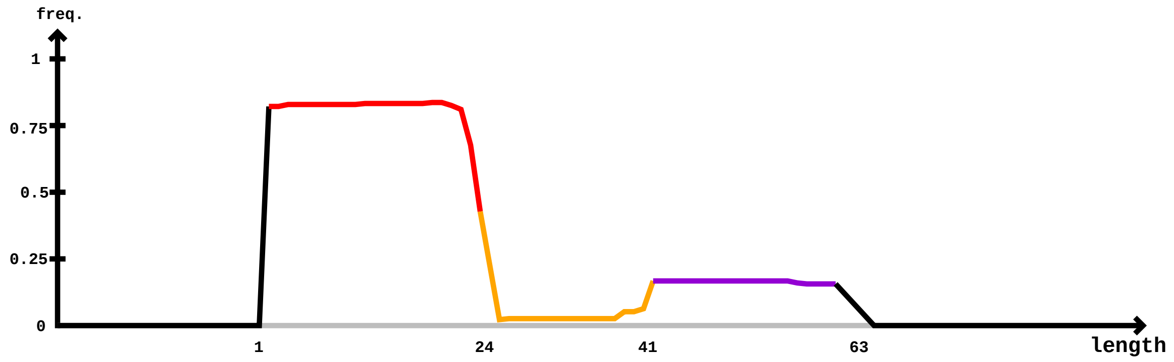


```
                        Mature                           Star
5'- agauuauuaaaaauacagaaucuugggccccacccccggagacugugaaucaguaaguagaucugggguagggccugggaauuuguguuuuuaaacaaauaacuucaggug  -3'   obs
    agauuauuaaaaauacagaaucuugggccccacccccggagacugugaaucaguaaguagaucugggguagggccugggaauuuguguuuuuaaacaaauaacuucaggug         exp
    ......((((((((((((.((((((((((...(((((((.((((.....)))).......)))))))..)))))))))).))))))))))))...................  reads   mm   sample
    ....................ucuugggccccacccccgga.......................................................................  1       0    s22
    ....................ucuugggccccacccccggaga.....................................................................  1       0    s22
    ....................ucuugggccccacccccggagac....................................................................  3       0    s22
    ....................ucuuCggccccacccccggagac....................................................................  1       1    s22
    ....................ucCugggccccacccccggagac....................................................................  1       1    s22

    ....................ucAugggccccacccccggag......................................................................  1       1    s06
    ....................ucuugggccccacccccggag......................................................................  1       0    s06
    ....................ucGugggccccacccccggag......................................................................  1       1    s06
    ....................ucCugggccccacccccggaga.....................................................................  1       1    s06
    ....................ucuugggccccacccccggaga.....................................................................  2       0    s06
    ....................ucuugggccccacccccggagac....................................................................  4       0    s06

    ....................ucuugggccccacccccggag......................................................................  3       0    s05
    ....................ucuugggccccacccccggagG.....................................................................  1       1    s05
    ....................ucuugggccccacccccggaga.....................................................................  2       0    s05
    ....................ucuugggccUcacccccggaga.....................................................................  1       1    s05
    ....................ucuugggccccacccccggagac....................................................................  10      0    s05
    ............................................................aucugggguagggccuggga...............................  1       0    s05
    ............................................................aucugggguagggccugggU...............................  1       1    s05

    ....................ucuugggccccacccccggag......................................................................  1       0    s16
    ....................ucGugggccccacccccggaga.....................................................................  1       1    s16
    ....................ucuugggccccacccccggaga.....................................................................  1       0    s16
    ....................ucuugggccccacccccggagac....................................................................  4       0    s16
    ...........................................................gaucugggguagggccCggga...............................  1       1    s16
    ...........................................................gaucugggguagggccugggaU..............................  1       1    s16
    ............................................................aucugggguGgggccugggaa..............................  1       1    s16

    ....................ucuugggccccacccccggaga.....................................................................  2       0    s02
    ....................ucuugggccccacccccggagac....................................................................  3       0    s02
    ............................................................aucugggguagggccuggga...............................  1       0    s02
    ............................................................aucugggguagggccugggaa..............................  1       0    s02
```

```
                    Mature                                  Star
agauuauuaaaaauacagaaucuugggccccacccccggagacugugaaucaguaaguagaucugggguagggccugggaauuuguguuuuuaaacaaauaacuucaggug

....................ucuugggccccacccccggag......................................................................   3   0   s17
....................ucuuCggccccacccccggag......................................................................   1   1   s17
....................ucGugggccccacccccggag......................................................................   1   1   s17
....................ucGugggccccacccccggaga.....................................................................   1   1   s17
....................ucuugggccccacccccggagU.....................................................................   2   1   s17
....................ucuugggccccacccccggaga.....................................................................   5   0   s17
....................ucuugggccccaccccAggagac....................................................................   1   1   s17
....................ucuGggccccacccccggagac.....................................................................   1   1   s17
.....................................ggagacugugaaucaguaaguagaucugggguGgggccugggaa..............................   1   1   s17
............................................................aucugggguagggccuggga...............................   1   0   s17
............................................................aucugggguagggccugggaa..............................   2   0   s17

....................ucuugggccccacccccggag......................................................................   1   0   s13
....................ucAugggccccacccccggag......................................................................   1   1   s13
....................ucuugggccccacccccggaga.....................................................................   2   0   s13
....................ucuugggccccacccccggagac....................................................................   1   0   s13
....................ucuugggccccacccccggagacC...................................................................   1   1   s13
............................................................aucugggguagggccuggga...............................   1   0   s13

....................ucuugggccccacccccggagac....................................................................   3   0   s15
....................ucuuCggccccacccccggagac....................................................................   1   1   s15
............................................gugaaucaguaaguagaucugggguGgggccugggaa..............................   1   1   s15
............................................gugaaucaguaaguagaucugggguagggccugggaaC.............................   1   1   s15
.........................................................uagaucugggguagggcc....................................   2   0   s15
.........................................................uagaucugggguagggccugggaa..............................   1   0   s15
............................................................aucugggguagggccugggaU..............................   1   1   s15

....................ucGugggccccacccccgg........................................................................   1   1   s04
....................ucuGggccccacccccggaga......................................................................   1   1   s04
....................ucGugggccccacccccggaga.....................................................................   1   1   s04
....................ucuugggccccacccccggaga.....................................................................   2   0   s04
....................ucAugggccccacccccggagac....................................................................   1   1   s04
....................ucuugggccccacccccggagac....................................................................   1   0   s04
.........................................................uagaucugggguagggccugggaa..............................   1   0   s04

....................ucCugggccccacccccgga.......................................................................   1   1   s01
....................ucuugggccccacccccggaga.....................................................................   1   0   s01
....................ucuugggccccacccccggagac....................................................................   3   0   s01
......................uugggccccacccccggagac....................................................................   1   0   s01
..........................................cugugaaucaguaaguagaucugggguagggccugggaa..............................   1   0   s01
............................................................aucugggguagggccuggga...............................   1   0   s01

....................ucuugggccccacccccggag......................................................................   2   0   s12
....................ucuuCggccccacccccggag......................................................................   1   1   s12
....................ucCugggccccacccccggag......................................................................   1   1   s12
....................ucuugggccccacccccggaga.....................................................................   5   0   s12
....................ucGugggccccacccccggaga.....................................................................   1   1   s12
....................ucuugggccccacccccggagac....................................................................   7   0   s12
....................ucuugggcccUaccccggagac.....................................................................   1   1   s12
....................ucCugggccccacccccggagac....................................................................   1   1   s12
...........................................................gaucugggguagggccugggaa..............................   1   0   s12
............................................................aucugggguagggccugggaa..............................   1   0   s12

....................ucuugggccccacccccggag......................................................................   1   0   s07
....................ucuugggccccacccccggaC......................................................................   1   1   s07
....................ucuugggccccacccccggaga.....................................................................   3   0   s07
....................ucuugggccccacccccggagac....................................................................   6   0   s07
............................................................aucugggguagggccugggaU..............................   1   1   s07

....................ucuugggccccacccccggag......................................................................   2   0   s14
....................ucCugggccccacccccggaga.....................................................................   1   1   s14
....................ucuugggccccacccccggaga.....................................................................   1   0   s14
....................ucCugggccccacccccggagac....................................................................   1   1   s14
....................ucGugggccccacccccggagac....................................................................   1   1   s14
..............................cacccccggagacugugaauca...........................................................   1   0   s14
............................................................aGcugggguagggccuggga...............................   1   1   s14

....................ucGugggccccacccccggag......................................................................   1   1   s19
....................ucuugggccccacccccggag......................................................................   3   0   s19
```

Mature Star			
agauuauuaaaaauacagaaucuugggccccacccccggagacugugaaucaguaaguagaucugggguagggccugggaauuuguguuuuuaaacaaauaacuucaggug			
....................ucCugggccccacccccggaga...	1	1	s19
....................ucuugggccccacccccggaga...	6	0	s19
....................ucGugggccccacccccggagac..	1	1	s19
....................ucuugggccccacccccggagac..	8	0	s19
....................ucuugggccccacccccggagacG...	1	1	s19
....aucugggguagggccCggga...............................	1	1	s19
....aucugggguagggccugggaa..............................	1	0	s19
....aucugggguGgggccugggaa..............................	1	1	s19
....................ucCugggccccacccccgg..	1	1	s09
....................ucuugggccccacccccgga...	1	0	s09
....................ucuugggccccacccccggag..	2	0	s09
....................ucAugggccccacccccggaga...	1	1	s09
....................ucCugggccccacccccggaga...	1	1	s09
....................ucuugggccccacccccggagac..	1	0	s09
....guaaguagaucugggguGgggccugggaa..............................	1	1	s09
....................ucuugggccccacccccggaga...	2	0	s11
....................ucuugggAcccacccccggagac..	1	1	s11
....................ucGugggccccacccccggagac..	1	1	s11
....................ucuugggccccacccccggagac..	3	0	s11
....uagaucugggguagggccC...................................	1	1	s11
....aucugggguagggccuggCa...............................	1	1	s11
....aucugggguagggccCgggaa..............................	1	1	s11
....................ucuugggccccacccccggag..	1	0	s23
....................ucuugggccccacccccggaga...	3	0	s23
....................ucuugggccccacGcccggagac..	1	1	s23
....................ucuugggccccacccccggagac..	3	0	s23
....uagaucugggguGgggccugggaa..............................	1	1	s23
....aucugggguagggccuggga...............................	1	0	s23
....................ucuGggccccacccccggag...	1	1	s24
....................ucuugggccccacccccggaga...	2	0	s24
....................ucAugggccccacccccggagac..	2	1	s24
....................ucuugggccccacccccggagac..	3	0	s24
....aucugggguaUggccugggaau.............................	1	1	s24
....................ucGugggccccacccccggag..	1	1	s21
....................ucuugggccccacccccggaga...	1	0	s21
....................ucuugggccccacccccggagac..	3	0	s21
....aucugggguagggccuggga...............................	1	0	s21
....................ucuugggccccacccccggag..	2	0	s20
....................ucuugggccccacccccggaga...	1	0	s20
....................ucuugggccccacccccggagac..	1	0	s20
....................ucuugggccccacccccgg..	1	0	s03
....................ucuugggccccacccccggaga...	1	0	s03
....................ucCugggccccacccccggagac..	1	1	s03
....................ucuugggccccacccccggagac..	2	0	s03
....................ucuuCggccccacccccggagac..	1	1	s03
....aucugggguagggccugggaaC.............................	2	1	s03
....................ucuugggccccacccccgga...	1	0	s08
....................ucAugggccccacccccggaga...	1	1	s08
....................ucuuCggccccacccccggagac..	1	1	s08
....................ucCugggccccacccccggagac..	1	1	s08
....................ucuugggccccacccccggagac..	3	0	s08
....................ucGugggccccacccccggagac..	2	1	s08
....gugaaucaguaaguagaucugggguGgggccugggaau.............................	1	1	s08
....aucugggguagggccugggaa..............................	1	0	s08
....aucugggguGgggccugggaa..............................	1	1	s08
....................ucuugggccccacccccggag..	3	0	s10
....................ucuugggccccacccccggaga...	5	0	s10
....................ucCugggccccacccccggaga...	1	1	s10
....................ucAugggccccacccccggaga...	1	1	s10
....................ucGugggccccacccccggagac..	2	1	s10
....................ucCugggccccacccccggagac..	1	1	s10

Mature **Star**

```
agauuauuaaaaauacagaaucuugggccccacccccggagacugugaaucaguaaguagaucugggguagggccugggaauuuguguuuuuaaacaaauaacuucaggug
....................ucuugggccccacccccggagac....................................................................	7	0	s10
.............................................ugaaucaguaaguagaucugggguagggccugggaa..............................	1	0	s10
.........................................................uagaucugggguagggccuggga...............................	1	0	s10
............................................................aucugggguagggccuggga...............................	2	0	s10

....................ucuugggccccacccccggaga.....................................................................	3	0	s18
....................ucuugggccccacccccggagac....................................................................	4	0	s18
......................uugggccccacccccggagac....................................................................	1	0	s18
```
