# Supplementary material for: The Identification of Circulating MiRNA in Bovine Serum and Their Potential as Novel Biomarkers of Early Mycobacterium avium subsp paratuberculosis Infection
Source: PLoS One. 2015 Jul 28;10(7):e0134310. doi: 10.1371/journal.pone.0134310 (PMC4517789; doi:10.1371/journal.pone.0134310)
Supplement: S1 File — (ZIP) [file pone.0134310.s008.zip › novel_pdfs/24_14535.pdf]

Provisional ID	:	24_14535
Score total	:	198.4
Score for star read(s)	:	3.9
Score for read counts	:	191.8
Score for mfe	:	1.7
Score for randfold	:	1.6
Score for cons. seed	:	-0.6
Total read count	:	388
Mature read count	:	228
Loop read count	:	0
Star read count	:	160

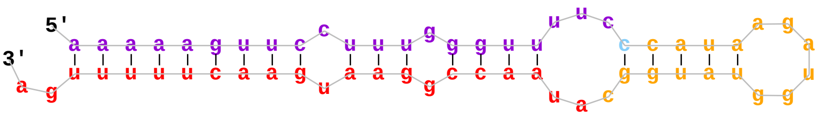

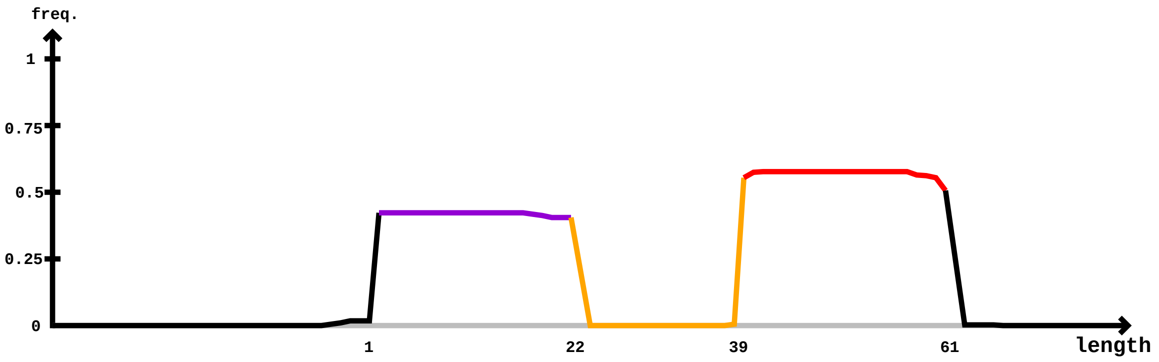


```
                                                    Star                                    Mature
5'- acuauauucuaaggggaaauauuagguuggccaaaaaguuccuuuggguuuucccauaagaugguauggcauaaccggaaugaacuuuuugaccaacucaauaucacucu  -3'   obs
    acuauauucuaaggggaaauauuagguuggccaaaaaguuccuuuggguuuucccauaagaugguauggcauaaccggaaugaacuuuuugaccaacucaauaucacucu         exp
    ............((((..(((((.((((((.((((((((((.(((.((((...(((((......)))))...)))).))).))))))))))).)))))).)))))..))))   reads   mm   sample
    .............................gccaaaaaguucGuuuggguu............................................................   1       1    s19
    ................................aaaaaguucGuuuggguuuuc.........................................................   11      1    s19
    .....................................................................Aauaaccggaaugaacuuuuu....................   1       1    s19
    ......................................................................auaaAcggaaugaacuuuuu....................   1       1    s19
    ......................................................................auUaccggaaugaacuuuuug...................   1       1    s19
    ......................................................................auaaccggaaugaacuuuuugG..................   1       1    s19
    ......................................................................aAaaccggaaugaacuuuuuga..................   36      1    s19
    .......................................................................Aaaccggaaugaacuuuuuga..................   1       1    s19

    ................................aaaaaguucGuuuggguuuuc.........................................................   5       1    s09
    ......................................................................aAaaccggaaugaacuuuuuga..................   5       1    s09
    .......................................................................Aaaccggaaugaacuuuuuga..................   2       1    s09

    ................................aaaaaguucGuuuggguuuuc.........................................................   3       1    s07
    ......................................................................aCaaccggaaugaacuuuuug...................   1       1    s07
    ......................................................................auaGccggaaugaacuuuuug...................   1       1    s07
    ......................................................................aAaaccggaaugaacuuuuuga..................   2       1    s07

    ................................aaaaaguucGuuuggguuuuc.........................................................   2       1    s14
    ......................................................................aCaaccggaaugaacuuuuuga..................   1       1    s14
    ......................................................................aAaaccggaaugaacuuuuuga..................   5       1    s14

    ................................aaaaaguucGuuuggguuuuc.........................................................   10      1    s12
    ......................................................................aGaaccggaaugaacuuu......................   2       1    s12
    ......................................................................auaaccggaaugaacuuuuug...................   1       0    s12
    ......................................................................aGaaccggaaugaacuuuuug...................   1       1    s12
    ......................................................................aAaaccggaaugaacuuuuuga..................   13      1    s12

    ................................aaaaaguucGuuuggguuuuc.........................................................   3       1    s17
    ......................................................................aCaaccggaaugaacuuuuug...................   2       1    s17
    ......................................................................aAaaccggaaugaacuuuuuga..................   8       1    s17

    ................................aaaaaguucGuuuggguuuuc.........................................................   8       1    s02
```

```
                                Star                                    Mature
acuauauucuaaggggaaauauuagguuggccaaaaaguuccuuuggguuuucccauaagaugguauggcauaaccggaaugaacuuuuugaccaacucaauaucacucu
......................................................................auUaccggaaugaacuuuuug...................   1   1   s02
......................................................................aAaaccggaaugaacuuuuuga..................   5   1   s02

................................aaaaaguucGuuuggguuuuc.........................................................   15  1   s05
.....................................................................Aauaaccggaaugaacuuuuug...................   1   1   s05
......................................................................auaaUcggaaugaacuuu......................   1   1   s05
......................................................................auaaAcggaaugaacuuuuug...................   1   1   s05
......................................................................aAaaccggaaugaacuuuuuga..................   17  1   s05

............................ggccaaaaaguucGuuuggg..............................................................   1   1   s22
................................aaaaaguucGuuuggguuuuc.........................................................   6   1   s22
......................................................................aAaaccggaaugaacuuuuuga..................   2   1   s22

...........................uggccaaaaaguucGuuuggguu............................................................   1   1   s06
.............................gccaaaaaguucGuuugggu.............................................................   1   1   s06
................................aaaaaguucGuuuggguuuuc.........................................................   5   1   s06
......................................................................auUaccggaaugaacuuuuug...................   1   1   s06
......................................................................auaaccggaaugaacuuuuugG..................   1   1   s06
......................................................................aAaaccggaaugaacuuuuuga..................   3   1   s06

...........................uggccaaaaaguuccuCuggg..............................................................   1   1   s16
................................aaaaaguucGuuuggguuuuc.........................................................   6   1   s16
......................................................................aAaaccggaaugaacuuuuuga..................   4   1   s16

................................aaaaaguucGuuuggguuuuc.........................................................   2   1   s01
......................................................................auaaAcggaaugaacuuu......................   1   1   s01
......................................................................auaaccggaaugaacuuuuug...................   1   0   s01
......................................................................aAaaccggaaugaacuuuuuga..................   6   1   s01
......................................................................auaaccggaaugaacuuuuuga..................   1   0   s01

................................aaaaaguucGuuuggguuuuc.........................................................   6   1   s13
......................................................................aAaaccggaaugaacuuuuuga..................   3   1   s13

................................aaaaaguucGuuuggguuuuc.........................................................   7   1   s15
......................................................................aGaaccggaaugaacuuuuug...................   2   1   s15
......................................................................aAaaccggaaugaacuuuuuga..................   2   1   s15

................................aaaaaguucGuuuggguuuuc.........................................................   4   1   s04
......................................................................aAaaccggaaugaacuuuuuga..................   4   1   s04

................................aaaaaguucGuuuggguuuuc.........................................................   19  1   s10
......................................................................auUaccggaaugaacuuuuug...................   1   1   s10
......................................................................aAaaccggaaugaacuuuuuga..................   5   1   s10
.......................................................................Aaaccggaaugaacuuuuuga..................   2   1   s10

................................aaaaaguucGuuuggguuuuc.........................................................   11  1   s08
......................................................................aGaaccggaaugaacuuuuug...................   1   1   s08
......................................................................aAaaccggaaugaacuuuuuga..................   7   1   s08
.......................................................................Aaaccggaaugaacuuuuuga..................   1   1   s08

......................................................................aAaaccggaaugaacuuuuuga..................   5   1   s18
........................................................................aaccUgaaugaacuuuuugaccaac.............   1   1   s18

................................aaaaaguucGuuuggguuuuc.........................................................   8   1   s03
......................................................................aAaaccggaaugaacuuuuuga..................   8   1   s03

............................ggccaaaaaguucGuuugggu.............................................................   1   1   s11
................................aaaaaguucGuuuggguuuuc.........................................................   3   1   s11
......................................................................aCaaccggaaugaacuuu......................   1   1   s11
......................................................................auaaccUgaaugaacuuuuu....................   1   1   s11
......................................................................aAaaccggaaugaacuuuuuga..................   15  1   s11

................................aaaaaguucGuuuggguuuuc.........................................................   6   1   s20
......................................................................aAaaccggaaugaacuuuuuga..................   4   1   s20

................................aaaaaguucGuuuggguuuuc.........................................................   6   1   s23
......................................................................aAaaccggaaugaacuuuuuga..................   4   1   s23

................................aaaaaguucGuuuggguuuuc.........................................................   6   1   s21
......................................................................aGaaccggaaugaacuuuuug...................   1   1   s21
```

```
                                Star                                 Mature
acuauauucuaaggggaaauauuagguuggccaaaaaguuccuuuggguuuucccauaagaugguauggcauaaccggaaugaacuuuuugaccaacucaauaucacucu
......................................................................aAaaccggaaugaacuuuuuga..................   3    1    s21
.......................................................................Aaaccggaaugaacuuuuuga..................   1    1    s21

.............................gccaaaaaguucGuuuggguu............................................................   1    1    s24
................................aaaaaguucGuuuggguuuuc.........................................................   8    1    s24
......................................................................aCaaccggaaugaacuuuu.....................   1    1    s24
......................................................................auaaccggaaugaacuuuuug...................   1    0    s24
......................................................................aCaaccggaaugaacuuuuug...................   1    1    s24
......................................................................aAaaccggaaugaacuuuuuga..................   21   1    s24
.......................................................................Aaaccggaaugaacuuuuuga..................   1    1    s24
```
