# Supplementary material for: The Identification of Circulating MiRNA in Bovine Serum and Their Potential as Novel Biomarkers of Early Mycobacterium avium subsp paratuberculosis Infection
Source: PLoS One. 2015 Jul 28;10(7):e0134310. doi: 10.1371/journal.pone.0134310 (PMC4517789; doi:10.1371/journal.pone.0134310)
Supplement: S1 File — (ZIP) [file pone.0134310.s008.zip › novel_pdfs/6_21896.pdf]

Provisional ID	:	6_21896
Score total	:	120.6
Score for star read(s)	:	3.9
Score for read counts	:	114.3
Score for mfe	:	1.4
Score for randfold	:	1.6
Score for cons. seed	:	-0.6
Total read count	:	236
Mature read count	:	235
Loop read count	:	0
Star read count	:	1

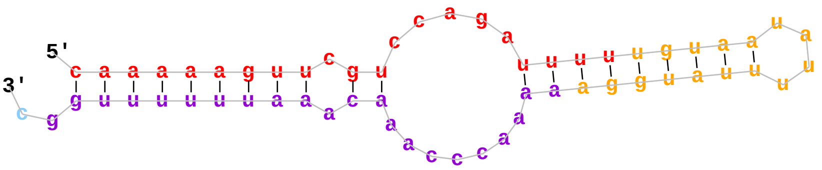

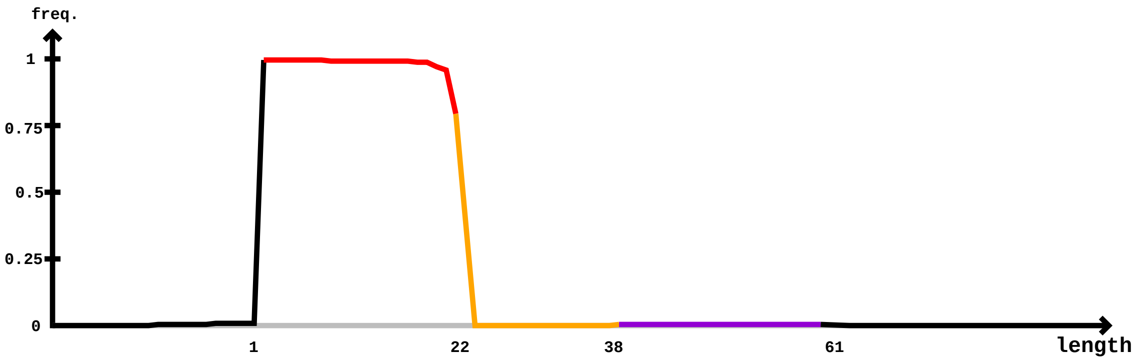


```
                         Mature                                  Star
5'- aaacaagguauuggguuggccaaaaaguucguccagauuuuuguaauauuuuauggaaaaacccaaacaaauuuuuuggccaacccaauauaaaggcacaaaugaguu   -3'   obs
    aaacaagguauuggguuggccaaaaaguucguccagauuuuuguaauauuuuauggaaaaacccaaacaaauuuuuuggccaacccaauauaaaggcacaaaugaguu         exp
    .......((((((((((((((((((((((.((.....(((((((((....))))))))).......)).)))))))))))))))))))))).................    reads   mm   sample
    ....................caaaaaguucguccagauuuu...................................................................    2       0    s20

    ....................caaaaaguucguccagauuuu...................................................................    8       0    s23
    .........................................................aaaacccaaacaaaCuuuuugg.............................    1       1    s23

    ....................caaaaaguucguccagauuuu...................................................................    5       0    s21

    ...............uuggccaaaaaguucguUcag........................................................................    1       1    s24
    ....................caaaaaguucguccagauu.....................................................................    1       0    s24
    ....................caUaaaguucguccagauuuu...................................................................    1       1    s24
    ....................caaaaaguucguccagauuuu...................................................................    8       0    s24
    ....................caaaaaguucguccagauuuuu..................................................................    1       0    s24

    ....................caaaaaguucguccagauuu....................................................................    1       0    s11
    ....................caaaCaguucguccagauuu....................................................................    1       1    s11
    ....................caaaaaguucguccagauuuu...................................................................    4       0    s11
    ....................caUaaaguucguccagauuuu...................................................................    1       1    s11

    ....................caaaaaguucguccagauuu....................................................................    1       0    s18
    ....................caaaaaguucguccagauuuu...................................................................    7       0    s18
    ....................caaaaaguucguccagauuuuu..................................................................    1       0    s18

    ....................caaaaaguucguccagau......................................................................    2       0    s10
    ....................caaaaaguucguccagauuu....................................................................    1       0    s10
    ....................caaaCaguucguccagauuuu...................................................................    1       1    s10
    ....................caaaaaguucguccagauuuu...................................................................    12      0    s10

    .........aGuggguuggccaaaaag.................................................................................    1       1    s08
    ....................caaaaaguucguccagauuu....................................................................    1       0    s08
    ....................caaaaaguucguccagauuuu...................................................................    5       0    s08
    ....................caaaCaguucguccagauuuu...................................................................    1       1    s08
    ....................caaaaaguucguccagauuuuu..................................................................    1       0    s08

    ....................caUaaaguucguccagauuuu...................................................................    4       1    s03
```

```
                    Mature                               Star
aaacaagguauuggguuggccaaaaaguucguccagauuuuuguaauauuuuauggaaaaacccaaacaaauuuuuuggccaacccaauauaaaggcacaaaugaguu
....................caaaaaguucguccagauuuu...................................................................   13   0   s03
....................caaaaaguucguccagauuuuu..................................................................   1    0   s03

....................caaaaaguucguccagau......................................................................   1    0   s12
....................caaaaaguGcguccagauuu....................................................................   1    1   s12
....................caaaaaguucguccagauuu....................................................................   7    0   s12
....................caaaaaguucguccagauuuu...................................................................   17   0   s12
....................caaaCaguucguccagauuuu...................................................................   1    1   s12
....................caaaGaguucguccagauuuu...................................................................   1    1   s12

....................caaaaaguucguccagauuu....................................................................   4    0   s01
....................caaaaaguucguccagauuuu...................................................................   6    0   s01
....................caaaCaguucguccagauuuu...................................................................   1    1   s01

....................caaaaaguucguccagauuu....................................................................   1    0   s15
....................caaaaaguucguccagauuuu...................................................................   1    0   s15
....................caaaaaguucguccagauuuuu..................................................................   1    0   s15

....................caaaaaguucguccagauuuu...................................................................   1    0   s13

....................caaaaaguucguccaUauuuu...................................................................   1    1   s04
....................caaaaaguucguccagauuuu...................................................................   4    0   s04
....................caaaaaguucguccCgauuuu...................................................................   1    1   s04

....................caUaaaguucguccagauuu....................................................................   1    1   s17
....................caaaaaguucguccagauuu....................................................................   3    0   s17
....................caUaaaguucguccagauuuu...................................................................   2    1   s17
....................caaaaaguucguccagauuuu...................................................................   7    0   s17

....................caaaaaguucguccagauuuu...................................................................   10   0   s02

....................caaaaaguucguccagauu.....................................................................   1    0   s22
....................caaaaaguucguccagauuu....................................................................   3    0   s22
....................caaaaaguucguccagauuuu...................................................................   5    0   s22
....................caaaCaguucguccagauuuu...................................................................   1    1   s22

....................caaaaaguucguccagauuu....................................................................   1    0   s06
....................caaaaaguucguccagauuuu...................................................................   1    0   s06
....................caaaCaguucguccagauuuu...................................................................   1    1   s06
....................caaaaaguucgAccagauuuu...................................................................   1    1   s06

....................caaaaaguucguccagauuu....................................................................   1    0   s16
....................caaaaaguucguccagauuuu...................................................................   2    0   s16
....................caUaaaguucguccagauuuu...................................................................   1    1   s16
....................caaaaaguucguccagauuuuu..................................................................   1    0   s16

....................caaaaaguucguccagau......................................................................   1    0   s05
....................caaaaaguucguccagauu.....................................................................   1    0   s05
....................caaaaaguucguccagauuuu...................................................................   14   0   s05

....................caaaaaguucguccagauuu....................................................................   1    0   s09
....................caaaaaguucguccagauuuu...................................................................   3    0   s09

....................caaaaaguucguccagauuu....................................................................   5    0   s19
....................caUaaaguucguccagauuu....................................................................   1    1   s19
....................caaaaaguucguccagauuuu...................................................................   17   0   s19
....................caaGaaguucguccagauuuu...................................................................   1    1   s19
....................caaaCaguucguccagauuuu...................................................................   2    1   s19
....................caUaaaguucguccagauuuu...................................................................   1    1   s19

....................caaaaaguucguccagauuu....................................................................   2    0   s14
....................caaaCaguucguccagauuu....................................................................   1    1   s14
....................caaaaaguucguccCgauuuu...................................................................   1    1   s14
....................caaaaaguucguccagauuuu...................................................................   4    0   s14

....................caaaaaguucguccagauuu....................................................................   2    0   s07
....................caaaaaguucguccagauuuu...................................................................   2    0   s07
....................caaaCaguucguccagauuuu...................................................................   1    1   s07
```
