# Supplementary material for: The Identification of Circulating MiRNA in Bovine Serum and Their Potential as Novel Biomarkers of Early Mycobacterium avium subsp paratuberculosis Infection
Source: PLoS One. 2015 Jul 28;10(7):e0134310. doi: 10.1371/journal.pone.0134310 (PMC4517789; doi:10.1371/journal.pone.0134310)
Supplement: S1 File — (ZIP) [file pone.0134310.s008.zip › novel_pdfs/13_4213.pdf]

Provisional ID	:	13_4213
Score total	:	160
Score for star read(s)	:	3.9
Score for read counts	:	152.6
Score for mfe	:	2.6
Score for randfold	:	1.6
Score for cons. seed	:	-0.6
Total read count	:	311
Mature read count	:	309
Loop read count	:	0
Star read count	:	2

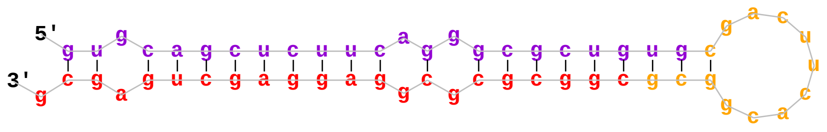

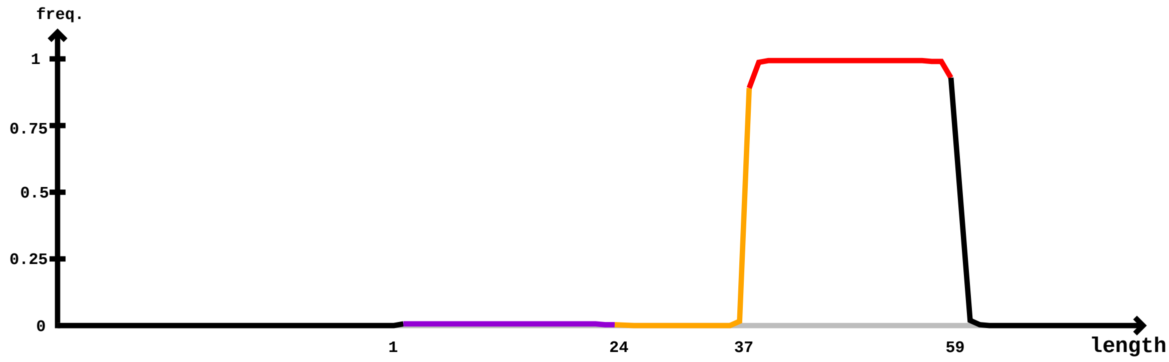

Star Mature

```
5'- caguaccgcggagccuugcagccccccggcgcccgugcagcucuucagggcgcugugcgacuucacggcgcggcgcgcggaggagcugagcgucagccgcggggacaggcu   -3'    obs
    caguaccgcggagccuugcagccccccggcgcccgugcagcucuucagggcgcugugcgacuucacggcgcggcgcgcggaggagcugagcgucagccgcggggacaggcu          exp
    ...........(((((.....((((.((((...(((.(((((((((.(.(((((((((.........))))))))).).))))))))).)))...)))).)))).)))))   reads   mm   sample
    ......................................................................cggcgcgcggaggagcugagcg...................   7       0    s11
    ......................................................................cggcgcgcggaggagcugagcgu..................   2       0    s11
    ......................................................................cggcgcgcggaggagcuUagcgu..................   1       1    s11
    .......................................................................ggcgcgcggaggagcugagcg...................   1       0    s11
    .......................................................................ggcUcgcggaggagcugagcgu..................   1       1    s11

    ......................................................................cggcgcgcggaggagcugagcg...................   2       0    s23
    ......................................................................cggcgcgcggaggagcugagcgu..................   1       0    s23
    .......................................................................ggcgcgcggaggagcugagcg...................   1       0    s23

    ......................................................................cggcgcgcggaggagcugagc....................   2       0    s21
    ......................................................................cggcgcgcggaggagcugagcg...................   3       0    s21
    ......................................................................cggcgcgcggaggagcugagcgu..................   2       0    s21
    .......................................................................ggcgcgcggaggagcugagcg...................   1       0    s21

    .....................................................................gcggAgcgcggaggagcugagcgu..................   1       1    s24
    ......................................................................cUgcgcgcggaggagcugagc....................   1       1    s24
    ......................................................................cggcgcgcggaggagcugagc....................   1       0    s24
    ......................................................................cggcgcgcggaggagcugagcg...................   7       0    s24
    ......................................................................cggcgcgcggaggagcugagcgu..................   1       0    s24

    .....................................................................gcggcgcgcggaggagcugagcg...................   2       0    s20
    .....................................................................gcUgcgcgcggaggagcugagcg...................   1       1    s20
    ......................................................................cggcgcgcggaggagcugagcg...................   4       0    s20

    ......................................................................cggcgcgcggaggagcugagc....................   1       0    s03
    ......................................................................cggcgcgcggaggagcugagcg...................   2       0    s03
    ......................................................................cggcgcgcggCggagcugagcg...................   1       1    s03
    ......................................................................cggcgcgcggaggagcugagcgu..................   1       0    s03
    .......................................................................ggUgcgcggaggagcugagcg...................   1       1    s03
    .......................................................................ggcgcgcggaggagcugagcg...................   1       0    s03

    ......................................................................cggcgcgcggaggagcugagc....................   1       0    s08
```

```
                                 Star                              Mature
caguaccgcggagccuugcagcccccggcgcccgugcagcucuucagggcgcugugcgacuucacggcgcggcgcgcggaggagcugagcgucagccgcggggacaggcu
.....................................................................cggcgcgcggaggagcugagcg...................   5    0    s08
......................................................................ggcgcgcggaggagcugagcg...................   1    0    s08

.....................................................................cggcgcgcggCggagcugagcg...................   2    1    s10
.....................................................................cggcgcgcggaggagcugagcg...................   21   0    s10
.....................................................................cgUcgcgcggaggagcugagcg...................   2    1    s10
.....................................................................cggcAcgcggaggagcugagcg...................   1    1    s10
.....................................................................cggcCcgcggaggagcugagcg...................   2    1    s10
.....................................................................cggcgcgcggaggagcugagcgu..................   6    0    s10
.....................................................................cggcgcgcggaggagcugagcgG..................   1    1    s10
......................................................................ggcgcgcAgaggagcugagcg...................   1    1    s10
......................................................................ggcgcgcggaggagcugagcg...................   3    0    s10
......................................................................ggcgAgcggaggagcugagcg...................   1    1    s10
......................................................................ggcgcgcggaggagcugagcguA.................   1    1    s10

.....................................................................cggcgcgcggaggagcugagc....................   1    0    s18
.....................................................................cggcgcgcggaggagcugagcg...................   6    0    s18
.....................................................................cggGgcgcggaggagcugagcg...................   1    1    s18
.....................................................................cggcgcgcggCggagcugagcg...................   1    1    s18
.....................................................................cggcgcgcggaggagcugagcgu..................   2    0    s18
.....................................................................cAgcgcgcggaggagcugagcgu..................   1    1    s18

.....................................................................cggcgcgcggaggagcugCgcg...................   1    1    s16
.....................................................................cggcgcgcAgaggagcugagcg...................   1    1    s16
.....................................................................cggcgcgcggaggagcugagcg...................   4    0    s16
.....................................................................cggcCcgcggaggagcugagcg...................   1    1    s16
.....................................................................cggcgAgcggaggagcugagcg...................   1    1    s16
.....................................................................cggcgcgcggaggagcugagcgu..................   1    0    s16
......................................................................ggcgcgcggaggagcugagcg...................   1    0    s16

.....................................................................cggcgcgcggaggagcugagc....................   1    0    s06
.....................................................................cgUcgcgcggaggagcugagcg...................   1    1    s06
.....................................................................cggcgcgcggaggagcugagcg...................   5    0    s06
.....................................................................cggcgcgcggaggagcugagcgG..................   1    1    s06

.....................................................................cggcgcgcggaggagcugagc....................   2    0    s22
.....................................................................cggcgcgcggCggagcugagcg...................   1    1    s22
.....................................................................cggcgcgcggaggagcugagcg...................   11   0    s22
.....................................................................cggcgcgcggaggagcugagcgu..................   3    0    s22
.....................................................................cUgcgcgcggaggagcugagcgu..................   1    1    s22
.....................................................................cggcgcgcggaggagcugagcguA.................   1    1    s22
......................................................................gUcgcgcggaggagcugagcgu..................   1    1    s22

.................................gugcagcucuucagggcgcug........................................................   1    0    s05
.....................................................................cggcgcgcggaggagcugagcg...................   36   0    s05
.....................................................................cUgcgcgcggaggagcugagcg...................   1    1    s05
.....................................................................cggcgcgcggaggagcugCgcg...................   1    1    s05
.....................................................................cgUcgcgcggaggagcugagcg...................   1    1    s05
.....................................................................cggcgcgcggCggagcugagcg...................   1    1    s05
.....................................................................cggcCcgcggaggagcugagcg...................   1    1    s05
.....................................................................cggcgcgcggCggagcugagcgu..................   1    1    s05
.....................................................................cggcgcgcggaggagcugagcgu..................   4    0    s05
.....................................................................cggcgcgcggaggagcugagcguA.................   1    1    s05
......................................................................ggcgcgcggaggagcugagcg...................   5    0    s05
.......................................................................gcgcgcggaggagcugagcguc.................   1    0    s05

.................................gugcagcucuucagggcgcugug......................................................   1    0    s17
.....................................................................cggcgcgcggaggagcugagc....................   1    0    s17
.....................................................................cgUcgcgcggaggagcugagcg...................   1    1    s17
.....................................................................cggcgcgcggaggagcugagcg...................   6    0    s17
.....................................................................cggcgcgcggaggagcugagcgu..................   1    0    s17
......................................................................ggcgcgcggaggagcugagcg...................   2    0    s17

.....................................................................cggcgcgcggaggagcugagc....................   1    0    s02
.....................................................................cggcgcgcggCggagcugagcg...................   1    1    s02
.....................................................................cggcgcgcggaggagcugagcg...................   4    0    s02
.....................................................................cggcgcgcggaggagcugagcgu..................   3    0    s02
.....................................................................cggcgcgcggaggagcugagcguAa................   1    1    s02
......................................................................ggcgcgcggaggagcugagcgu..................   2    0    s02

.....................................................................cggcgcgcggaggagcugagcg...................   3    0    s04
```

```
                                 Star                              Mature
caguaccgcggagccuugcagcccccggcgcccgugcagcucuucagggcgcugugcgacuucacggcgcggcgcgcggaggagcugagcgucagccgcggggacaggcu

....................................................................gcggcgcgcggaggagcugagcg...................	1	0	s13
.....................................................................cggcgcgcggaggagcugagc....................	1	0	s13
.....................................................................cgUcgcgcggaggagcugagc....................	1	1	s13
.....................................................................cggcCcgcggaggagcugagcg...................	1	1	s13
.....................................................................cggcgcgcggaggagcugagcg...................	5	0	s13
.....................................................................cggcgcgcggaggagcugCgcgu..................	1	1	s13
.....................................................................cggcgcgcggaggagcugagcgu..................	3	0	s13
......................................................................ggcUcgcggaggagcugagcg...................	1	1	s13

.....................................................................cggcgcgcggaggagcugagc....................	1	0	s15
.....................................................................cggcgcgcggaggagcugCgcg...................	1	1	s15
.....................................................................cggcgcgcggaggagcugagcg...................	9	0	s15
......................................................................ggcgAgcggaggagcugagcg...................	1	1	s15

.....................................................................cggcgcgcggCggagcugagcg...................	1	1	s01
.....................................................................cggcgcgcggaggagcugagcg...................	5	0	s01
.....................................................................cggcgcgcggaggagcugagcgu..................	1	0	s01
......................................................................ggcgAgcggaggagcugagcg...................	1	1	s01
......................................................................ggcgcgcggaggagcugagcgu..................	1	0	s01

.....................................................................cggcgcgcggaggagcugagc....................	1	0	s12
.....................................................................cggcgcgcggaggagcugagcg...................	7	0	s12
.....................................................................cUgcgcgcggaggagcugagcg...................	1	1	s12
.....................................................................cggcgcgcggCggagcugagcg...................	1	1	s12
.....................................................................cggcgcgcggaggagcugagcgu..................	2	0	s12
......................................................................ggcgcgcggaggagcugagcg...................	1	0	s12
.......................................................................gcgcgcggaggagcugagcguc.................	1	0	s12

.....................................................................cggcgcgcggaggagcugagcg...................	1	0	s07

.....................................................................Uggcgcgcggaggagcugagc....................	1	1	s14
.....................................................................cggcgcgcggaggagcugagcg...................	8	0	s14
.....................................................................cggcgcgcggaggagcugagcgu..................	1	0	s14

.....................................................................cggcCcgcggaggagcuga......................	1	1	s19
.....................................................................cggcgcgcggaggagcugagc....................	1	0	s19
.....................................................................cggcgcgcggaggagcugagcg...................	10	0	s19
.....................................................................cggcgcgcggaggagcugagcgu..................	1	0	s19

.....................................................................cggcgcgcggaggagcugagc....................	1	0	s09
.....................................................................cggcgcgcggaggagcugagcg...................	6	0	s09
.....................................................................cggcgcgcggCggagcugagcg...................	1	1	s09
.....................................................................cggcgcgcggaggagcugagcgu..................	1	0	s09
......................................................................ggcgcgcggaggagcugagcgu..................	1	0	s09
```
