# Supplementary material for: The Identification of Circulating MiRNA in Bovine Serum and Their Potential as Novel Biomarkers of Early Mycobacterium avium subsp paratuberculosis Infection
Source: PLoS One. 2015 Jul 28;10(7):e0134310. doi: 10.1371/journal.pone.0134310 (PMC4517789; doi:10.1371/journal.pone.0134310)
Supplement: S1 File — (ZIP) [file pone.0134310.s008.zip › novel_pdfs/28_17041.pdf]

Provisional ID : 28_17041
Score total : 5.3
Score for star read(s) : -1.3
Score for read counts : 0
Score for mfe : 2
Score for randfold : 1.6
Score for cons. seed : 3
Total read count : 256
Mature read count : 256
Loop read count : 0
Star read count : 0

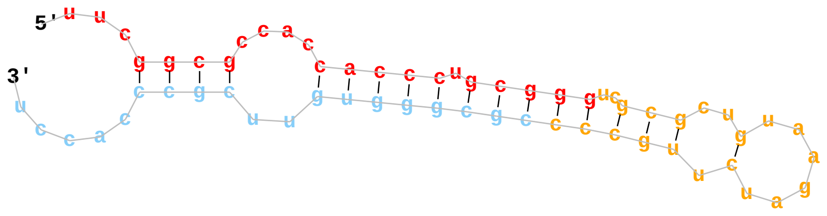

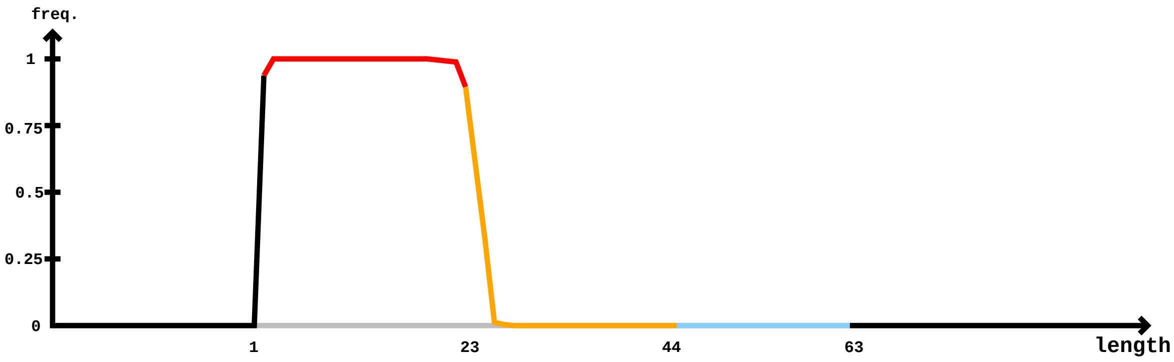

Mature Star

```
5'- ugacggcuggaguugacaguuucggcgccaccacccugcggguucgcgcuguaagaucuugccccgcggguguucgcccaccugucaucuccucugcgccucagcccucugcc  -3'  exp
    (((.(((.((((.((((((....((((....(((((.(((((..(((..(......).)))))))))))))..))))...)))))).)))).....))))))..........       reads  mm  sample
    ....................uucggcgccaccacccugcgg.......................................................................       1      0   s03
    ....................uucggcgccaccacccugcggg......................................................................       5      0   s03
    ....................uAcggcgccaccacccugcggg......................................................................       1      1   s03

    ....................uucggcgccaccacccugc.........................................................................       1      0   s10
    ....................uucggcgccaccacccugcgg.......................................................................       1      0   s10
    ....................uucggcgccaccacccugcggA......................................................................       1      1   s10
    ....................uGcggcgccaccacccugcggg......................................................................       1      1   s10
    ....................uucggcgccaccacccugcggg......................................................................       10     0   s10
    ....................uucUgcgccaccacccugcggg......................................................................       1      1   s10
    ....................uucggcgccaccacccugcgggu.....................................................................       2      0   s10
    ....................uucggcgccaccacccugcggguc....................................................................       9      0   s10
    ....................uucgCcgccaccacccugcggguc....................................................................       1      1   s10
    ....................uucUgcgccaccacccugcggguc....................................................................       1      1   s10
    .....................ucgUcgccaccacccugcggg......................................................................       1      1   s10
    .....................ucggcgccaccacccugcgggA.....................................................................       1      1   s10

    ....................uucggcgccaccacccugcggg......................................................................       6      0   s08
    ....................uucggcgccaccacccugcgggG.....................................................................       1      1   s08
    ....................uucggcgccaccacccugcgggA.....................................................................       1      1   s08
    ....................uucggcgccaccacccugcggguA....................................................................       1      1   s08
    ....................uucggcgccaccacccugcggguc....................................................................       1      0   s08
    .....................ucggcgccaccacccugcgggu.....................................................................       1      0   s08

    ....................uucggcgccaccacccugcggg......................................................................       3      0   s18
    ....................uGcggcgccaccacccugcggg......................................................................       1      1   s18
    ....................uucCgcgccaccacccugcggg......................................................................       1      1   s18
    ....................uCcggcgccaccacccugcggg......................................................................       1      1   s18
    ....................uucggcgccaccacccugcgggA.....................................................................       1      1   s18
    ....................uucggcgccaccacccugcggguc....................................................................       2      0   s18

    ....................uucggcgccaccacccug..........................................................................       1      0   s11
    ....................uucggcgccaccacccugcgg.......................................................................       2      0   s11
    ....................uucggcgccaccacccugcggg......................................................................       2      0   s11
```

```
                    Mature                                        Star
ugacggcuggaguugacaguuucggcgccaccacccugcgggucgcgcuguaagaucuugccccgcggguguucgcccaccugucaucuccucugcgccucagcccucugcc
....................uucggcgccaccacccugcggguc....................................................................    4    0    s11

....................uucggcgccaccacccugcggg......................................................................    3    0    s20

....................uucgAcgccaccacccugcggg......................................................................    1    1    s24
....................uucggcgccaccacccugcggg......................................................................    5    0    s24
....................uucggcgccaccacccugcgggu.....................................................................    1    0    s24
....................uucggcgccaccacccugcggguc....................................................................    3    0    s24
.....................ucggcgccaccacccugcggg......................................................................    2    0    s24

....................uucggcgccaccacccugcgg.......................................................................    1    0    s21
....................uucCgcgccaccacccugcggg......................................................................    1    1    s21
....................uucggcgccaccacccugcggg......................................................................    2    0    s21
....................uucggcgccaccacccugcggguc....................................................................    3    0    s21
....................uucggcgccaccacccugcgggucgU..................................................................    1    1    s21

....................uucggcgccaccacccugcgg.......................................................................    1    0    s23
....................uCcggcgccaccacccugcggg......................................................................    1    1    s23
....................uucggcgccaccacccugcggU......................................................................    1    1    s23
....................uucggcgccaccacccugcgggu.....................................................................    1    0    s23
....................uucggcgccaccacccugcggguc....................................................................    3    0    s23
.....................ucggcgccaccacccugcggg......................................................................    1    0    s23
.....................ucgUcgccaccacccugcggg......................................................................    1    1    s23

....................uucggcgccaccacccugcgg.......................................................................    1    0    s07
....................uucggcgccaccacccugcggg......................................................................    2    0    s07
....................uucggcgccaccacccugcggguc....................................................................    3    0    s07
.....................ucggcgccaccacccugcggg......................................................................    1    0    s07

....................uucggcgccaccacccugcggg......................................................................    1    0    s14
....................uucggcgccaccacccugcggguc....................................................................    1    0    s14
.....................ucggcgccaccacccugcggg......................................................................    1    0    s14
.....................ucggAgccaccacccugcggg......................................................................    1    1    s14

....................uucggcgccaccacccugcgg.......................................................................    3    0    s19
....................uucggcgccaccacccugcggg......................................................................    1    0    s19
....................uucUgcgccaccacccugcggg......................................................................    1    1    s19
....................uucggcgccaccacccugcgggu.....................................................................    1    0    s19
....................uGcggcgccaccacccugcgggu.....................................................................    1    1    s19
....................uucggcgccaccacccugcggguc....................................................................    1    0    s19

....................uucggcgccaccacccugcgg.......................................................................    1    0    s09
....................uucUgcgccaccacccugcggg......................................................................    1    1    s09
....................uucggcgccaccacccugcggg......................................................................    7    0    s09
....................uucggcgccaccacccugcggguc....................................................................    2    0    s09
.....................ucggcgccaccacccugcggg......................................................................    1    0    s09

....................uGcggcgccaccacccugcgg.......................................................................    1    1    s17
....................uucggcgccaccacccugcgg.......................................................................    1    0    s17
....................uucggcgccaccacccugUggg......................................................................    1    1    s17
....................uGcggcgccaccacccugcggg......................................................................    1    1    s17
....................uucggcgccaccacccugcggg......................................................................    4    0    s17
....................uucUgcgccaccacccugcggg......................................................................    1    1    s17
....................uucggcgccaccacccugcggguA....................................................................    1    1    s17
....................uGcggcgccaccacccugcggguc....................................................................    2    1    s17
....................uucggcgccaccacccugcggguc....................................................................    5    0    s17
....................uucggcgccaccacccugcggguU....................................................................    1    1    s17

....................uucggcgccaccacccugcggg......................................................................    1    0    s02
....................uucgCcgccaccacccugcggg......................................................................    1    1    s02
....................uuUggcgccaccacccugcggg......................................................................    1    1    s02
....................uucggcgccaccacccugcggguc....................................................................    5    0    s02

....................uucUgcgccaccacccugcggg......................................................................    1    1    s06
....................uucggcgccaccacccugcggg......................................................................    4    0    s06
....................uucggcgccaccacccugcggguc....................................................................    1    0    s06
....................uucggcgccaccacccugcgggucU...................................................................    1    1    s06
.....................ucggcgccaccacccugcggg......................................................................    1    0    s06

....................uucggcgccaccacccugcgA.......................................................................    1    1    s16
```

```
                    Mature                                      Star
ugacggcuggaguugacaguuucggcgccaccacccugcgggucgcgcuguaagaucuugccccgcggguguucgcccaccugucaucuccucugcgccucagcccucugcc
....................uucggcgccaccacccugcggg......................................................................  2  0  s16
....................uucUgcgccaccacccugcggg......................................................................  1  1  s16
....................uucgCcgccaccacccugcggguc....................................................................  1  1  s16
....................uucggcgccaccacccugcggguc....................................................................  1  0  s16

....................uucggcgccaccacccugcgg.......................................................................  1  0  s05
....................uucggcgccaccacccugcggg......................................................................  9  0  s05
....................uGcggcgccaccacccugcggg......................................................................  2  1  s05
....................uucUgcgccaccacccugcggg......................................................................  1  1  s05
....................uucgCcgccaccacccugcggg......................................................................  1  1  s05
....................uucggcgccaccacccugcgggG.....................................................................  1  1  s05
....................uucggcgccaccacccugcgggu.....................................................................  1  0  s05
....................uucggcgccaccacccugcggguA....................................................................  2  1  s05
....................uucggcgccaccacccugcggguc....................................................................  3  0  s05

....................uucggcgccaccacccugcgg.......................................................................  2  0  s22
....................uucggcgcAaccacccugcggg......................................................................  1  1  s22
....................uucggcgccaccacccugcggg......................................................................  3  0  s22
....................uucggcgccaccacccugcggguc....................................................................  3  0  s22

....................uucggcgccaccacccugcgg.......................................................................  1  0  s01
....................uucggcgccaccacccugcggg......................................................................  2  0  s01
....................uucggcgccaccacccugcgggA.....................................................................  1  1  s01
....................uucggcgccaccacccugcgggu.....................................................................  1  0  s01
....................uuUggcgccaccacccugcggguc....................................................................  1  1  s01
....................uucggcgccaccacccugcggguc....................................................................  3  0  s01
.....................ucggcgccaccacccugcggg......................................................................  1  0  s01

....................uucggcgccaccacccugcggg......................................................................  4  0  s13
....................uucggcgccaccacccugcggguc....................................................................  4  0  s13

....................uucggcgccaccacccugcgg.......................................................................  2  0  s04
....................uucggcgccaccacccugcggg......................................................................  3  0  s04
....................uGcggcgccaccacccugcggg......................................................................  1  1  s04
....................uucggcgccaccacccugcgggu.....................................................................  2  0  s04
....................uucggcgccaccacccugcggguc....................................................................  3  0  s04
....................uucggcgccaccacccugcggguA....................................................................  1  1  s04
....................uuUggcgccaccacccugcggguc....................................................................  1  1  s04
.....................ucggcgccaccacccugcgg.......................................................................  1  0  s04
.....................ucgCcgccaccacccugcggg......................................................................  1  1  s04

....................uucggcgccaccacccugcA........................................................................  1  1  s15
....................uucggcgccaccacccugcgg.......................................................................  1  0  s15
....................uucUgcgccaccacccugcggg......................................................................  1  1  s15
....................uucggcgccaccacccugcggg......................................................................  2  0  s15
....................uucggcgccaccacccugcgggA.....................................................................  1  1  s15
....................uucggcgccaccacccugcggguc....................................................................  3  0  s15
....................uCcggcgccaccacccugcggguc....................................................................  1  1  s15
....................uuUggcgccaccacccugcggguc....................................................................  1  1  s15

....................uucggcgccaccacccugcgg.......................................................................  2  0  s12
....................uucUgcgccaccacccugcggg......................................................................  1  1  s12
....................uucggcgccaccacccugcggg......................................................................  5  0  s12
....................uucggcgccaccacccugcggguc....................................................................  3  0  s12
....................uucggcgccaccacccugcgggucU...................................................................  1  1  s12
.....................ucggAgccaccacccugcggg......................................................................  1  1  s12
```
