# Supplementary material for: The Identification of Circulating MiRNA in Bovine Serum and Their Potential as Novel Biomarkers of Early Mycobacterium avium subsp paratuberculosis Infection
Source: PLoS One. 2015 Jul 28;10(7):e0134310. doi: 10.1371/journal.pone.0134310 (PMC4517789; doi:10.1371/journal.pone.0134310)
Supplement: S1 File — (ZIP) [file pone.0134310.s008.zip › novel_pdfs/22_13473.pdf]

Provisional ID	:	22_13473
Score total	:	133.1
Score for star read(s)	:	3.9
Score for read counts	:	126.1
Score for mfe	:	2.1
Score for randfold	:	1.6
Score for cons. seed	:	-0.6
Total read count	:	259
Mature read count	:	257
Loop read count	:	0
Star read count	:	2

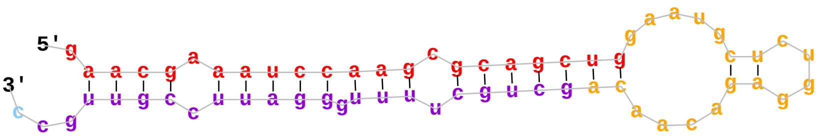

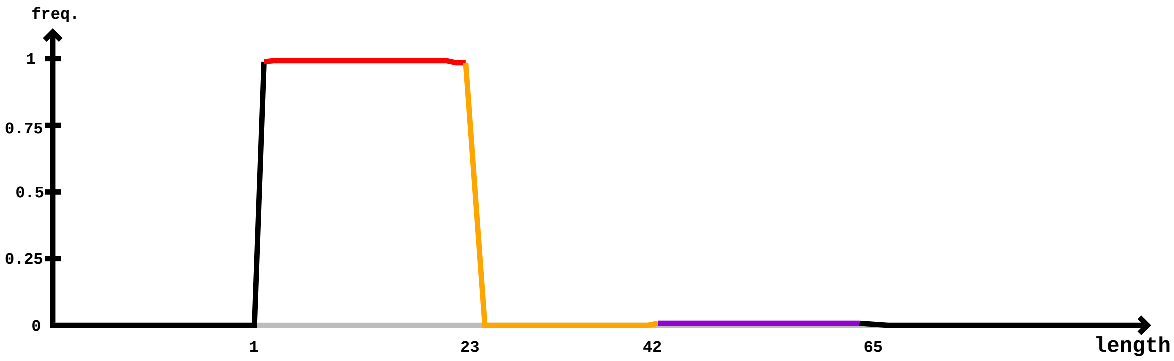

Mature Star

```
5'- ugcucaggcaggagagcagggaacgaaauccaagcgcagcuggaaugcucuggagacaacagcugcuuuugggauuccguugcccgcuguccagccauggcggggcggggag   -3'   obs
    ugcucaggcaggagagcagggaacgaaauccaagcgcagcuggaaugcucuggagacaacagcugcuuuugggauuccguugcccgcuguccagccauggcggggcggggag         exp
    ((((..(((.(((.(((.((.((((.((((((((.(((((((.....((....))....))))))).))).))))).)))).)).))).))).)))..))))..........   reads   mm   sample
    ....................gaacgaaauccaagcgcagcug......................................................................   8       0    s09

    ....................gaacgaaauccaagcgcagcug......................................................................   31      0    s19
    ....................gaacgaaaucUaagcgcagcug......................................................................   1       1    s19
    ....................gUacgaaauccaagcgcagcug......................................................................   1       1    s19
    ....................gaacgaaauccaGgcgcagcug......................................................................   1       1    s19
    ....................gaacgaaCuccaagcgcagcug......................................................................   2       1    s19

    ....................gaacgaaauccaagcgcagcug......................................................................   9       0    s14

    ....................gaacgaaauccaagcgcagcug......................................................................   1       0    s07

    ....................gaacgaaauccaagcgcagcug......................................................................   18      0    s12
    ....................gaacgaaCuccaagcgcagcug......................................................................   1       1    s12
    ....................gaaGgaaauccaagcgcagcug......................................................................   1       1    s12
    ....................gCacgaaauccaagcgcagcug......................................................................   1       1    s12
    ....................gaacgaaauccaGgcgcagcug......................................................................   1       1    s12

    ....................gaacgaaauccaagcgcagcug......................................................................   4       0    s15

    ....................gaacgaaauccaagcgcagcug......................................................................   3       0    s13

    ....................gaacgaaauccaagcgcagcug......................................................................   2       0    s04

    ....................gaacgaaauccaagcgcagcug......................................................................   9       0    s01
    ....................gaacgaaauccaGgcgcagcug......................................................................   1       1    s01
    .............................................................gcugcuuuugggauuccguugc.............................   1       0    s01
    .............................................................gcGgcuuuugggauuccguugc.............................   1       1    s01

    ....................gaacgaaauccaagcgcagcug......................................................................   4       0    s06

    ....................gaacgaaauccaagcgcagcug......................................................................   6       0    s16

    ....................gaacCaaauccaagcgcagcug......................................................................   1       1    s05
```

```
                    Mature                                        Star
ugcucaggcaggagagcagggaacgaaauccaagcgcagcuggaaugcucuggagacaacagcugcuuuugggauuccguugcccgcuguccagccauggcggggcggggag
....................gaacgaaauccaGgcgcagcug......................................................................    3     1     s05
....................gaacgaaauccaagcgcagcug......................................................................    30    0     s05
....................gaacgaaauUcaagcgcagcug......................................................................    1     1     s05

....................gaacgaaauccaagcgcagcug......................................................................    2     0     s22
....................gaacgaaCuccaagcgcagcug......................................................................    1     1     s22

....................gaacgaaauccaagcgcCgcug......................................................................    1     1     s02
....................gaaUgaaauccaagcgcagcug......................................................................    1     1     s02
....................gaacgaaauccaagcgcagcug......................................................................    19    0     s02
....................gaacgaaCuccaagcgcagcug......................................................................    1     1     s02
.....................aacgaaauccaagcgcagcug......................................................................    1     0     s02

....................gaacgaaauccaagcgcagcug......................................................................    9     0     s17
....................gaacgaaauccaGgcgcagcug......................................................................    1     1     s17

....................gaacgaaauccaagcgcagc........................................................................    1     0     s18
....................gaacgaaCuccaagcgcagcug......................................................................    1     1     s18
....................gaacgaaauccaagcgcagcug......................................................................    5     0     s18

....................gaacgaaauccaagcgcagcug......................................................................    6     0     s08
....................gaacgaaauccaagcgcagcugA.....................................................................    1     1     s08

....................gaacgaaauccaGgcgcagcug......................................................................    1     1     s10
....................gaacgaaauccaagcgcagcGg......................................................................    1     1     s10
....................gaacgaaauccaagcgcagcug......................................................................    15    0     s10

....................gUacgaaauccaagcgcagcug......................................................................    1     1     s03
....................gaacgaaauccaGgcgcagcug......................................................................    2     1     s03
....................gaacgaaauccaagcgcagcug......................................................................    8     0     s03

....................gaacgaaauccaagcgcagcug......................................................................    20    0     s24
....................gaacCaaauccaagcgcagcug......................................................................    1     1     s24
....................gaacgaaauccaGgcgcagcug......................................................................    1     1     s24

....................gaacgaaauccaagcgcagcug......................................................................    3     0     s23
....................gUacgaaauccaagcgcagcug......................................................................    1     1     s23

....................gaacgaaauccaagcgcagcug......................................................................    2     0     s21

....................gaacgaaauccaagcgcagcug......................................................................    2     0     s20

....................gaacgaaauccaagcgcagc........................................................................    1     0     s11
....................gaacgaaauccaagcgcagcug......................................................................    7     0     s11
....................gaacgaaauccaagcgcagcugA.....................................................................    1     1     s11
```
