# Supplementary material for: The Identification of Circulating MiRNA in Bovine Serum and Their Potential as Novel Biomarkers of Early Mycobacterium avium subsp paratuberculosis Infection
Source: PLoS One. 2015 Jul 28;10(7):e0134310. doi: 10.1371/journal.pone.0134310 (PMC4517789; doi:10.1371/journal.pone.0134310)
Supplement: S1 File — (ZIP) [file pone.0134310.s008.zip › novel_pdfs/9_24602.pdf]

Provisional ID	:	9_24602
Score total	:	5.5
Score for star read(s)	:	-1.3
Score for read counts	:	0
Score for mfe	:	2.2
Score for randfold	:	1.6
Score for cons. seed	:	3
Total read count	:	636
Mature read count	:	439
Loop read count	:	176
Star read count	:	21

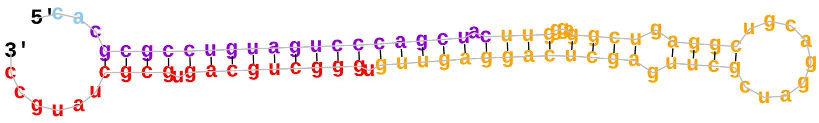

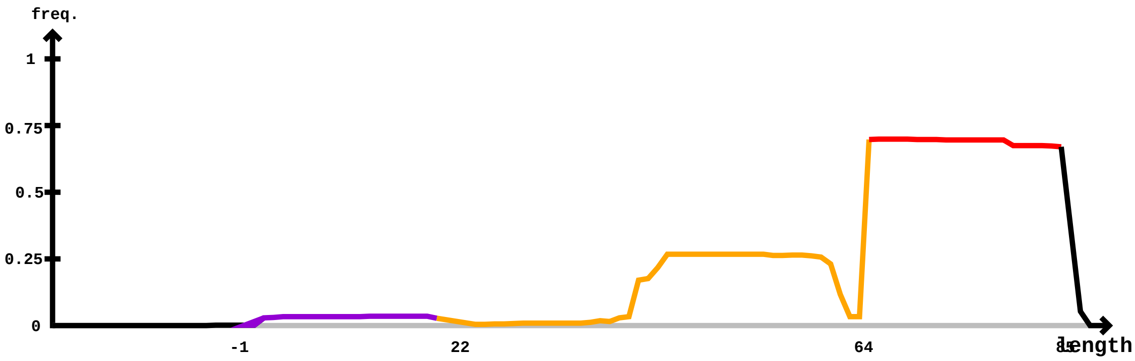


```
                     Star                                         Mature
5'- uccagccgggcgcaguggcacgcgccuguagucccagcuacuugggaggcugaggcugcaggaucgcuugagcucaggaguugugggcugcagugcgcuaugccgauc  -3'  obs
    uccagccgggcgcaguggcacgcgccuguagucccagcuacuugggaggcugaggcugcaggaucgcuugagcucaggaguugugggcugcagugcgcuaugccgauc       exp
    .((.....)).....(((((.((((((((((((((((((.((((...((((.((((.........)))).))))))))))))))).))))))))).)))).)))))...   reads   mm   sample
    ..............................................aggcugaggcugUaggaucgcuugagcucag...............................   1   1   s23
    ...........................................................aggaCcgcuugagcucaggag............................   1   1   s23
    ...........................................................aggaucgcuugagcucaggag............................   1   0   s23
    ...........................................................aggaucUcuugagcucaggagu...........................   1   1   s23
    ...........................................................aggaucgcuugagcucaggagu...........................   1   0   s23
    .............................................................gaucgcuugagcucaggag............................   1   0   s23
    ..............................................................aucgcuugagcucaggag............................   1   0   s23
    ..............................................................aucgcuugagcucaggagu...........................   1   0   s23
    ...................................................................................ugggcugcagugcgcuaugcc....   4   0   s23
    ...................................................................................ugUgcugcagugcgcuaugcc....   1   1   s23

    ...............uggcUcgcgccuguagucccagc......................................................................   1   1   s24
    ......................cgccuguagucccagcuac...................................................................   1   0   s24
    .......................................................cugcaggaucgcuugagcucaggaguuCugg......................   1   1   s24
    .........................................................gUaggaucgcuugagcucagga.............................   2   1   s24
    ...........................................................aggaucgcuugagcucagg..............................   1   0   s24
    ...........................................................aggCucgcuugagcucagga.............................   1   1   s24
    ...........................................................aggaucgcuugagcucaggag............................   2   0   s24
    ...........................................................aggaucgcuugagcucaggagu...........................   2   0   s24
    .............................................................gaucgcuugagcucagga.............................   1   0   s24
    ..............................................................aucgcuugagcucaggag............................   1   0   s24
    ..............................................................aucgcuugagcucaggagu...........................   1   0   s24
    ...................................................................................ugggcuUcagugcgcuaugcc....   1   1   s24
    ...................................................................................ugggcugcagugcgcuaugcc....   15  0   s24
    ...................................................................................ugUgcugcagugcgcuaugcc....   1   1   s24
    ...................................................................................uggCcugcagugcgcuaugcc....   1   1   s24
    ...................................................................................ugggcugcagugcAcuaugcc....   1   1   s24
    ...................................................................................ugggAugcagugcgcuaugccga..   1   1   s24
    ...................................................................................ugggcugcagugcgcuaugccga..   2   0   s24

    ....................cgcgccuguagucccagcuac...................................................................   1   0   s21
    ............................................................ggaucgcuugagcucaggag............................   1   0   s21
    ..............................................................................aguuAugggcugcagugcgc..........   1   1   s21
```

```
                    Star                                                             Mature
uccagccgggcgcaguggcacgcgccuguagucccagcuacuugggaggcugaggcugcaggaucgcuugagcucaggaguugugggcugcagugcgcuaugccgauc
...................................................................................ugggcugcagugcgcuaugcc....    4     0     s21

..........................................ugggaggcugaggcugGaggaucgcuugagc...................................    1     1     s20
......................................................gcugcaggaucgcuugagcucaggag............................    1     0     s20
...........................................................aggaucgcuugagcucagg..............................    1     0     s20
...........................................................aggaucgcuugagcucaggag............................    1     0     s20
.............................................................gaAcgcuugagcucaggag............................    1     1     s20
.............................................................gaucCcuugagcucaggag............................    1     1     s20
..............................................................aucgcuugagcucaggagu...........................    1     0     s20
................................................................................uuUugggcugcagugcgcuaugcc....    1     1     s20
...................................................................................ugUgcugcagugcgcuaugcc....    1     1     s20
...................................................................................ugggAugcagugcgcuaugcc....    2     1     s20
...................................................................................ugggcugcagugcgcuaugcc....    7     0     s20
...................................................................................ugggcugcagugcgcuaugccga..    2     0     s20

...........................................................aggaucgcuugagcucagg..............................    1     0     s11
...........................................................aggaucgcuugagcucaggag............................    3     0     s11
.............................................................gaucgcuugagcucaggag............................    1     0     s11
.............................................................gaGcgcuugagcucaggag............................    1     1     s11
.............................................................gaGcgcuugagcucaggagu...........................    1     1     s11
..............................................................auUgcuugagcucaggagu...........................    1     1     s11
..............................................................................aguugugggcugcagugcgc..........    1     0     s11
...................................................................................ugUgcugcagugcgcuaugcc....    1     1     s11
...................................................................................ugggcugcagugcgcuaugcc....    8     0     s11
...................................................................................ugggAugcagugcgcuaugcc....    1     1     s11

...........................................................aggaucgcuugagcucagg..............................    1     0     s18
...........................................................aggaucgcuugagcucaggag............................    2     0     s18
............................................................ggaucgcuugagcucaggag............................    1     0     s18
..............................................................................aguuAugggcugcagugcgc..........    1     1     s18
..............................................................................aguuAugggcugcagugcgcuaugccg...    1     1     s18
...................................................................................ugggcugcagugcgcuaugcc....    24    0     s18
...................................................................................uggCcugcagugcgcuaugcc....    1     1     s18
...................................................................................ugggAugcagugcgcuaugcc....    2     1     s18
...................................................................................ugggcugcagugcgcuaugccg...    1     0     s18
...................................................................................ugggcAgcagugcgcuaugccga..    1     1     s18
...................................................................................ugggcugcagugcgcuaugccga..    4     0     s18
...................................................................................ugggAugcagugcgcuaugccga..    1     1     s18

...............................................ggcugaggcugUaggaucgcuugagcucagg..............................    1     1     s08
...........................................................aggaucgcuugagcucagg..............................    2     0     s08
...........................................................aggaucgcuugagcucagga.............................    1     0     s08
...........................................................aggaucgcuugagcucaggag............................    11    0     s08
...........................................................aggGucgcuugagcucaggag............................    1     1     s08
...........................................................aggaucgcuugagcucaggagu...........................    2     0     s08
.............................................................gaucgcuugagcucaggag............................    2     0     s08
.............................................................gaucgcuugagcucaggagu...........................    3     0     s08
..............................................................aucgcuugagcucaggag............................    1     0     s08
...................................................................................ugggcugcagugcgcuaugcc....    6     0     s08
...................................................................................uggCcugcagugcgcuaugcc....    1     1     s08
...................................................................................ugggcugcagugcgcuaugccga..    1     0     s08
....................................................................................gggcugcagugcgcuaugccga..    1     0     s08

....................cgUgccuguagucccagcuac...................................................................    1     1     s10
....................cgcgAcuguagucccagcuac...................................................................    2     1     s10
...............................ucccagcuacuugggaggcugaggc....................................................    1     0     s10
...........................................................aCgaucgcuugagcucagga.............................    1     1     s10
...........................................................aggaucgcuugagcucaggagu...........................    1     0     s10
..............................................................aucgcuugagcucaggagu...........................    3     0     s10
..............................................................................aguuUugggcugcagugcgc..........    1     1     s10
..............................................................................aguuUugggcugcagugcgcuaugccga..    1     1     s10
...................................................................................ugUgcugcagugcgcuaugcc....    2     1     s10
...................................................................................ugggUugcagugcgcuaugcc....    2     1     s10
...................................................................................ugggAugcagugcgcuaugcc....    2     1     s10
...................................................................................uggCcugcagugcgcuaugcc....    1     1     s10
...................................................................................ugggcugcagugcgcuaugcc....    26    0     s10
...................................................................................uggUcugcagugcgcuaugcc....    1     1     s10
...................................................................................ugggcugcagugcgcuaugccga..    2     0     s10

.......................................................cugUaggaucgcuugagc...................................    1     1     s03
```

Star

Mature

```
uccagccgggcgcaguggcacgcgccuguagucccagcuacuugggaggcugaggcugcaggaucgcuugagcucaggaguugugggcugcagugcgcuaugccgauc
.........................................................gUaggaucgcuugagcucaggag............................   1   1   s03
.........................................................gAaggaucgcuugagcucaggagu...........................   1   1   s03
..........................................................Uaggaucgcuugagcucagga.............................   1   1   s03
...........................................................aggaucgcuugagcucagg..............................   1   0   s03
..............................................................aucgcuugagcucaggagu...........................   2   0   s03
..............................................................................aguugugggcugcagugcgc..........   1   0   s03
...................................................................................ugggcugcagugcgcuaug......   1   0   s03
...................................................................................ugggcugcagugcgcuaugc.....   2   0   s03
...................................................................................uggUcugcagugcgcuaugcc....   2   1   s03
...................................................................................ugggAugcagugcgcuaugcc....   4   1   s03
...................................................................................ugUgcugcagugcgcuaugcc....   2   1   s03
...................................................................................ugggcugcagugcgcuaugcc....  41   0   s03
...................................................................................uggCcugcagugcgcuaugcc....   1   1   s03
...................................................................................ugggcugcagugcgcuaugccg...   3   0   s03
...................................................................................ugggAugcagugcgcuaugccga..   1   1   s03
...................................................................................ugggcugcagugcgcuaugccga..   1   0   s03

....................cgcUccuguagucccagcuac...................................................................   1   1   s12
.........................................................gUaggaucgcuugagcucaggag............................   1   1   s12
...........................................................aggaucgcuugagcucag...............................   1   0   s12
...........................................................aggaucgcuugagcucagg..............................   1   0   s12
...........................................................aggaucgcuugagcucaggag............................   2   0   s12
...........................................................aUgaucgcuugagcucaggagu...........................   1   1   s12
...........................................................aggaucgcuugagcucaggagu...........................   3   0   s12
.............................................................gaucgcuugagcucaggag............................   1   0   s12
..............................................................aucgcuugagcucaggagu...........................   1   0   s12
...........................................................................aggaguuAugggcugcagugcgc..........   1   1   s12
..............................................................................aguuAugggcugcagugcgc..........   2   1   s12
...................................................................................ugggAugcagugcgcuaugcc....   4   1   s12
...................................................................................ugggGugcagugcgcuaugcc....   1   1   s12
...................................................................................uggUcugcagugcgcuaugcc....   2   1   s12
...................................................................................ugUgcugcagugcgcuaugcc....   1   1   s12
...................................................................................ugggcugcagugcgcuaugcc....  42   0   s12
...................................................................................ugggcugcagugcgcuaugccg...   1   0   s12
...................................................................................ugggcugcagugcgcuaugccga..   2   0   s12

.............................................................gaucgcuugagcucaggagu...........................   1   0   s15
..............................................................aucgcuugagcucaggag............................   1   0   s15
..............................................................aucgcuugagcucaggagu...........................   1   0   s15
...................................................................................ugUgcugcagugcgcuaugcc....   1   1   s15
...................................................................................ugggUugcagugcgcuaugcc....   1   1   s15
...................................................................................ugggcugcagugcgcuaugcc....   7   0   s15
...................................................................................ugggAugcagugcgcuaugcc....   1   1   s15

....................cgcgccuguagucccagcuac...................................................................   1   0   s13
...........................................................aggaucgcuugagcucagg..............................   1   0   s13
...........................................................aggaucgcuugagcucagga.............................   1   0   s13
...........................................................aggaucgcuugagcucagUa.............................   1   1   s13
...........................................................aCgaucgcuugagcucagga.............................   1   1   s13
...........................................................aggaucgcuugagcucaggag............................   1   0   s13
..............................................................aucgcuugagcucaggagu...........................   2   0   s13
...................................................................................ugggcugcagugcgcuaugcc....   1   0   s13
...................................................................................ugggcugcagugcgcuaugccga..   2   0   s13

....................cgcgccuguagucccagcuac...................................................................   1   0   s04
............................................ggaggcugaggcugAaggaucgcuugagc...................................   1   1   s04
...........................................................aggaucgcuugagcucagg..............................   1   0   s04
...........................................................aggaucgcuugagcucaggagu...........................   1   0   s04
.............................................................gaAcgcuugagcucaggagu...........................   1   1   s04
..............................................................aGcgcuugagcucaggagu...........................   1   1   s04
..............................................................................aguuAugggcugcagugcgc..........   1   1   s04
...................................................................................uUggcugcagugcgcuaugcc....   1   1   s04
...................................................................................ugggcugcagugcgcuaugcc....  11   0   s04

....................cgcgccuguagucccagc......................................................................   1   0   s01
....................cgcgccuguagucccagcuac...................................................................   1   0   s01
.........................................................gUaggaucgcuugagcucagg..............................   1   1   s01
...........................................................aggaucgcuugagcucaggag............................   3   0   s01
.............................................................Caucgcuugagcucaggag............................   1   1   s01
..............................................................aucgcuugagcucaggag............................   2   0   s01
```

```
                    Star                                                          Mature
uccagccgggcgcaguggcacgcgccuguagucccagcuacuugggaggcugaggcugcaggaucgcuugagcucaggaguugugggcugcagugcgcuaugccgauc
..............................................................aucgcuugagcucaggagu...........................   1    0    s01
...................................................................................ugUgcugcagugcgcuaugcc....   1    1    s01
...................................................................................uggUcugcagugcgcuaugcc....   1    1    s01
...................................................................................ugggcugcagugcgcuaugcc....   26   0    s01
...................................................................................ugggAugcagugcgcuaugcc....   1    1    s01
...................................................................................uggUcugcagugcgcuaugccg...   1    1    s01
...................................................................................ugggcugcagugcgcuaugccga..   1    0    s01

......................................................gcGgcaggaucgcuugagcucaggagu...........................   1    1    s16
...........................................................aggaCcgcuugagcucagg..............................   1    1    s16
...........................................................aggaucgcuugagcucagga.............................   1    0    s16
...........................................................aggaucgcuugagcucaggag............................   1    0    s16
.............................................................gaucgcuugagcucaggag............................   2    0    s16
.............................................................gaucgcuugagcucaggagu...........................   1    0    s16
...................................................................................ugggcugcagugcgcuaugcc....   3    0    s16
...................................................................................ugggcugcagugcgcuaugccga..   1    0    s16

..........................................................Uaggaucgcuugagcucaggag............................   2    1    s22
...........................................................aggaucgcuugagcucaggag............................   1    0    s22
...........................................................aggaucgcuugagcucaggagu...........................   1    0    s22
...........................................................agUaucgcuugagcucaggagu...........................   1    1    s22
..............................................................................aguuAugggcugcagugcgc..........   2    1    s22
...................................................................................uggUcugcagugcgcuaugcc....   1    1    s22
...................................................................................ugggUugcagugcgcuaugcc....   1    1    s22
...................................................................................ugggcugcagugcgcuaugcc....   6    0    s22
...................................................................................ugUgcugcagugcgcuaugcc....   1    1    s22

....................cgcgccuguagucccagcuac...................................................................   1    0    s06
......................cgccuguagucccagcuac...................................................................   1    0    s06
.........................................................gcaggaucgcuugagcucaggaguuCugggcugc.................   1    1    s06
...........................................................aggaucgcuuAagcucagga.............................   1    1    s06
...........................................................aggaucgcuugagcucaggag............................   1    0    s06
............................................................ggaucgcuugagcucaggag............................   1    0    s06
.............................................................gaAcgcuugagcucaggagu...........................   1    1    s06
..............................................................aucgcuugagcucaggagu...........................   1    0    s06
...................................................................................ugggcugcagugcgcuaugcc....   5    0    s06
...................................................................................uAggcugcagugcgcuaugcc....   1    1    s06
...................................................................................ugUgcugcagugcgcuaugcc....   1    1    s06
...................................................................................ugggcugcagugcgcuCugcc....   1    1    s06
...................................................................................uggUcugcagugcgcuaugccga..   1    1    s06

......................................uacuAgggaggcugaggc....................................................   1    1    s05
...........................................................aggaucgcuugagcucagg..............................   1    0    s05
...........................................................aggaucgcuugagcucagga.............................   1    0    s05
...........................................................aggaAcgcuugagcucaggag............................   1    1    s05
...........................................................aggaucgcuugagcucaggagu...........................   1    0    s05
.............................................................gaucgcuugagcucaggag............................   1    0    s05
..............................................................aucgcuugagcucaggag............................   1    0    s05
..............................................................................aguuAugggcugcagugcgc..........   1    1    s05
...................................................................................ugggAugcagugcgcuaugcc....   2    1    s05
...................................................................................ugggcugcagugcgcuaugcc....   19   0    s05
...................................................................................uggUcugcagugcgcuaugcc....   1    1    s05
...................................................................................ugggUugcagugcgcuaugcc....   1    1    s05
...................................................................................ugggcugcagugcgcuaugccga..   1    0    s05
...................................................................................uggUcugcagugcgcuaugccga..   1    1    s05

....................cgcgccuguagucccagc......................................................................   1    0    s02
.....................gcgccuguagucccagcua....................................................................   1    0    s02
.......................................................cugUaggaucgcuugagcucagg..............................   1    1    s02
...........................................................aggaucgcuugagcucagg..............................   1    0    s02
...........................................................aggaucgcuugagcucaggag............................   1    0    s02
..............................................................aucgAuugagcucaggagu...........................   1    1    s02
...............................................................................guugugggcugcagugcgc..........   1    0    s02
...................................................................................ugggcugcagugcgcuaugcc....   3    0    s02

....................cgcgccuguagucccagc......................................................................   1    0    s17
.......................................................cugUaggaucgcuugagcucaggagu...........................   1    1    s17
...........................................................aggaucgcuugagcucaggag............................   1    0    s17
...........................................................aggaucgcuugagcucaggagu...........................   1    0    s17
.............................................................gaucgcuugagcucaggag............................   1    0    s17
```

Star **Mature**

uccagccgggcgcaguggcacgcgccuguagucccagcuacuugggaggcugaggcugcaggaucgcuugagcucaggaguugugggcugcagugcgcuaugccgauc			
....aucgcuugagcucaggag............................	1	0	s17
....aucgcuugagcucaggagu...........................	1	0	s17
....ugggcugcagugcgcuaugcc....	4	0	s17
....ugggcugcagugcgcuaugccg...	1	0	s17
....ugggcugcagugcgcuaugccga..	1	0	s17
....uggUcugcagugcgcuaugccga..	1	1	s17
....gcaggaucgcuugagcucaggaguuCugggc....................	1	1	s09
....aggaucgcuugagcucaggag............................	1	0	s09
....aggaucgcuugagcucaggagu...........................	1	0	s09
....ggaucgcuugagcucaggagu...........................	1	0	s09
....gaucgcuugagcuUaggag............................	1	1	s09
....gaucCcuugagcucaggagu...........................	1	1	s09
....aCcgcuugagcucaggag............................	1	1	s09
....ugggUugcagugcgcuaugcc....	1	1	s09
....ugggcugcagugcgcuaugcc....	21	0	s09
....................cgcgccuguagucccagcua..	1	0	s19
....................cgcgccuguagucccCgcuac...	1	1	s19
....aggaucgcuugagcucagg..............................	1	0	s19
....aggaucgcuugagcucaggag............................	1	0	s19
....gaucgcuugagcucaggag............................	1	0	s19
....aucgcuugagcucaggagu...........................	1	0	s19
....guugugggcugcagugcgcuaugccg...	1	0	s19
....ugggcugcagugcgcuaugcc....	19	0	s19
....uggUcugcagugcgcuaugcc....	2	1	s19
....ugUgcugcagugcgcuaugcc....	1	1	s19
....ugggAugcagugcgcuaugcc....	2	1	s19
....uggCcugcagugcgcuaugcc....	1	1	s19
....ugggcugcagugcgcuaugccga..	2	0	s19
....gcugcagugcgcuaugccga..	1	0	s19
....................cgcgccuguagucccagc..	1	0	s14
....................cgcgccuguagucccagcuac...	1	0	s14
....gUaggaucgcuugagcucaggagu...........................	1	1	s14
....aggaucgcuugagcucagga.............................	1	0	s14
....aCgaucgcuugagcucaggag............................	1	1	s14
....aggauAgcuugagcucaggagu...........................	1	1	s14
....aucgcuugagcucaggagu...........................	1	0	s14
....aguuAugggcugcagugcgc..........	1	1	s14
....aguuAugggcugcagugcgcuaugccg...	1	1	s14
....ugggUugcagugcgcuaugcc....	2	1	s14
....ugggAugcagugcgcuaugcc....	3	1	s14
....ugggcugcagugcgcuaugcc....	19	0	s14
....ugggGugcagugcgcuaugcc....	1	1	s14
....uggUcugcagugcgcuaugcc....	1	1	s14
....................cgcgccuguagucccagc..	1	0	s07
....................cgcgUcuguagucccagcu...	1	1	s07
....aggaucgcuugagcucagg..............................	1	0	s07
....aggaucgcuugagcucagga.............................	4	0	s07
....aggGucgcuugagcucagga.............................	1	1	s07
....aggaucgcuugagcucaggag............................	4	0	s07
....gaucgcuugagcucaggag............................	2	0	s07
....aucgcuugagcucaggag............................	1	0	s07
....aucgcuugaCcucaggagu...........................	1	1	s07
....aucgcuugagcucaggagu...........................	2	0	s07
....ugggcugcagugcgcuaugcc....	4	0	s07
....ugggcugcagugcgcuaugccga..	2	0	s07
....uggUcugcagugcgcuaugccga..	1	1	s07
