# Supplementary material for: The Identification of Circulating MiRNA in Bovine Serum and Their Potential as Novel Biomarkers of Early Mycobacterium avium subsp paratuberculosis Infection
Source: PLoS One. 2015 Jul 28;10(7):e0134310. doi: 10.1371/journal.pone.0134310 (PMC4517789; doi:10.1371/journal.pone.0134310)
Supplement: S1 File — (ZIP) [file pone.0134310.s008.zip › novel_pdfs/13_4632.pdf]

Provisional ID	:	13_4632
Score total	:	5.7
Score for star read(s)	:	-1.3
Score for read counts	:	0
Score for mfe	:	2.4
Score for randfold	:	1.6
Score for cons. seed	:	3
Total read count	:	3135
Mature read count	:	3135
Loop read count	:	0
Star read count	:	0

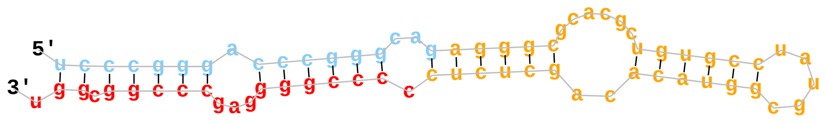

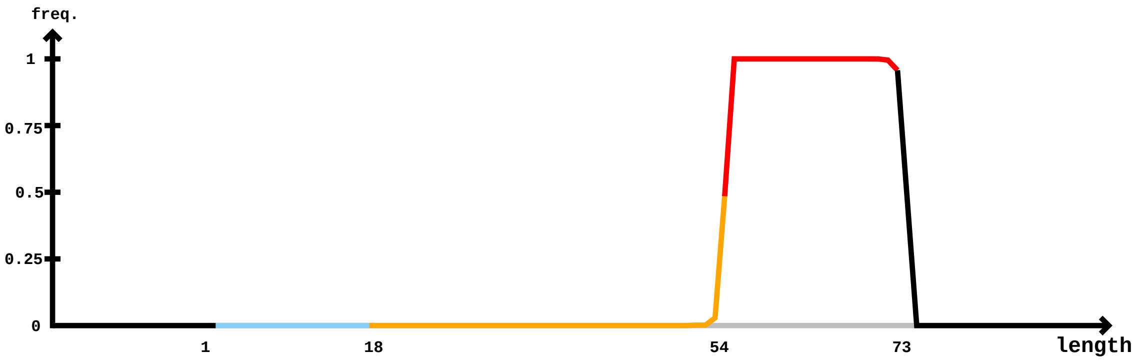

Star **Mature**

```
5'- cgugguccggggcgcucccgggacccgggcagagggcgcacgcugugccuaugcgguacacagcucucccccggggagcccggcggugcacgugagacccacuuugcg -3'   exp
    ...((((((.((((((.(((((.((((((..((((((......((((((.....))))))..)))))).))))))...))))).))))).).)).)))).........   reads   mm   sample
    ...................................................................cccccggggagcccggcg.......................   1       0    s07
    ...................................................................cccccggggagcccggcggG.....................   4       1    s07
    ....................................................................ccccggggagcccggcgg......................   6       0    s07
    ....................................................................ccccggggaAcccggcgg......................   1       1    s07
    ....................................................................ccccggggagcccggcggG.....................   83      1    s07
    ....................................................................ccccggggagcccggcggGg....................   2       1    s07
    .....................................................................cccggggagcccggcggG.....................   50      1    s07

    ...................................................................ccccAggggagcccggcgg......................   1       1    s14
    ...................................................................cccccggggagcccggcggG.....................   3       1    s14
    ....................................................................ccccggggagcccggcgg......................   3       0    s14
    ....................................................................ccccggggagcccggcggG.....................   40      1    s14
    ....................................................................ccccggggagcccggcggGg....................   1       1    s14
    .....................................................................cccggggagcccggcggC.....................   1       1    s14
    .....................................................................cccggggagcccggcggG.....................   46      1    s14

    .................................................................cGcccccggggagcccggcgg......................   1       1    s19
    ...................................................................cccccggggagcccggcggG.....................   9       1    s19
    ....................................................................ccccggggagcccggAgg......................   1       1    s19
    ....................................................................ccccggggagcccggcgg......................   4       0    s19
    ....................................................................ccccggggagcccggcggC.....................   1       1    s19
    ....................................................................ccccggggagcccggcggG.....................   39      1    s19
    ....................................................................ccccggggagcccggcggGg....................   2       1    s19
    .....................................................................cccggggagcccggcggG.....................   52      1    s19
    .....................................................................cccggggagcccggcggC.....................   1       1    s19
    .....................................................................cccggggagcccggcggGg....................   1       1    s19

    ...................................................................cccccggggagcccggcggG.....................   2       1    s09
    ....................................................................ccccggggagcccUgcgg......................   1       1    s09
    ....................................................................ccccggggagcccggcgg......................   3       0    s09
    ....................................................................ccccggggagcccggcggG.....................   31      1    s09
    ....................................................................ccccggggagcccggcggGg....................   2       1    s09
    .....................................................................cccggggagcccggcggG.....................   73      1    s09

    .................................................................cGcccccggggagcccggc........................   1       1    s05
```

```
                   Star                                                   Mature
cgugguccggggcgcucccgggacccgggcagagggcgcacgcugugccuaugcgguacacagcucuccccggggagcccggcggugcacgugagacccacuuugcg
...................................................................cccccggggagcccggcggG.....................   3     1    s05
....................................................................ccccggggagcccggcgg......................   4     0    s05
....................................................................ccccggggagcccggcggG.....................   50    1    s05
.....................................................................cccggggagcccggcggG.....................   63    1    s05

...................................................................cccccggggagcccggcg.......................   1     0    s06
....................................................................ccccggggagcccggcgg......................   4     0    s06
....................................................................ccccggggagcccggcggG.....................   56    1    s06
....................................................................ccccggggagcccggcggC.....................   1     1    s06
.....................................................................cccggggagcccggcggG.....................   41    1    s06

...................................................................cccccggggagcccggcg.......................   2     0    s22
...................................................................cccccggggagcccggcggG.....................   5     1    s22
....................................................................Ucccggggagcccggcgg......................   1     1    s22
....................................................................ccccggggaCcccggcgg......................   1     1    s22
....................................................................ccccggggagcccggcgg......................   6     0    s22
....................................................................ccccggggagcccggcggG.....................   63    1    s22
.....................................................................cccggggagcccggcggG.....................   83    1    s22

...................................................................cccccggggagcccggcg.......................   1     0    s16
...................................................................ccccAggggagcccggcgg......................   1     1    s16
...................................................................cccccggggagcccggcggG.....................   1     1    s16
....................................................................ccccggggagcccggcgg......................   3     0    s16
....................................................................cGccggggagcccggcgg......................   1     1    s16
....................................................................Gcccggggagcccggcgg......................   1     1    s16
....................................................................ccccggggagcccggcggG.....................   29    1    s16
.....................................................................cccggggagcccggcggG.....................   63    1    s16
.....................................................................cccggggagcccggcggC.....................   1     1    s16

....................................................................ccccggggagcccggcggG.....................   16    1    s02
....................................................................ccccggggagcccggcggGg....................   1     1    s02
.....................................................................cccggggagcccggcggG.....................   29    1    s02

...................................................................cccccggggagcccggcggG.....................   1     1    s17
....................................................................ccccggggagcccggcgg......................   4     0    s17
....................................................................ccccggggagcccggcggC.....................   1     1    s17
....................................................................ccccggggagcccggcggG.....................   48    1    s17
....................................................................ccccggggagcccggcggGg....................   1     1    s17
.....................................................................cccggggagcccggcggG.....................   67    1    s17

....................................................................ccccggggagcccggcgg......................   2     0    s15
....................................................................ccccggggagcccggcggG.....................   57    1    s15
....................................................................ccccggggagcccggcggGg....................   1     1    s15
.....................................................................cccggggagcccggcggG.....................   81    1    s15

..................................................................Gcccccggggagcccggcgg......................   1     1    s13
...................................................................cccccggggagcccggcggG.....................   7     1    s13
....................................................................cccAggggagcccggcgg......................   1     1    s13
....................................................................ccccggggagcccggcgg......................   19    0    s13
....................................................................ccccggggagcccggcggC.....................   1     1    s13
....................................................................ccccggggagcccggcggG.....................   136   1    s13
....................................................................ccccggggagcccggcggGg....................   6     1    s13
.....................................................................cccggggagcccggcggG.....................   95    1    s13

...................................................................cccccggggagcccggcg.......................   1     0    s04
...................................................................cccccggggagcccggcggG.....................   7     1    s04
....................................................................ccccggggagcccggcggG.....................   45    1    s04
.....................................................................cccggggagcccggcggG.....................   88    1    s04

...................................................................cccccggggagcccggcg.......................   1     0    s01
...................................................................cccccggggagcccggcggG.....................   1     1    s01
....................................................................ccccggggagcccggcgg......................   3     0    s01
....................................................................ccccggggagcccggcggG.....................   31    1    s01
....................................................................ccccggggagcccggcggC.....................   1     1    s01
.....................................................................cccggggagcccggcggG.....................   53    1    s01

...................................................................cccccggggagcccggcggG.....................   4     1    s12
....................................................................ccccggggagcccggcgg......................   3     0    s12
....................................................................ccccggggagcccggcggG.....................   89    1    s12
....................................................................ccccggggagcccggcggGg....................   2     1    s12
```

Star Mature

cgugguccggggcgcucccgggacccgggcagagggcgcacgcugugccuaugcgguacacagcucucccccggggagcccggcggugcacgugagacccacuuugcg			
....cccggggagcccggcggG.....................	91	1	s12
....cGcccccggggagcccggcgg......................	1	1	s03
....ccccGgggagcccggcg........................	1	1	s03
....cccccggggagcccggcg.......................	1	0	s03
....cccccggggagcccggcgg......................	1	0	s03
....cccccggggagcccggcggG.....................	3	1	s03
....ccccggggagcccggcgg......................	6	0	s03
....ccUcggggagcccggcgg......................	2	1	s03
....ccccggggagcccggcggG.....................	40	1	s03
....ccccggggagcccggcggGg....................	1	1	s03
....cccggggagcccggcggG.....................	56	1	s03
....cccggggagcccggcggC.....................	1	1	s03
....cGcccccggggagcccggcgg......................	1	1	s08
....cccccggggagcccggcggG.....................	5	1	s08
....ccccggggagcccggcgg......................	6	0	s08
....ccccggggagcccggcggC.....................	1	1	s08
....ccccggggagcccggcggG.....................	75	1	s08
....ccccggggagcccggcggGg....................	2	1	s08
....cccggggagcccggcggG.....................	84	1	s08
....cccccggggagcccggcggG.....................	1	1	s10
....ccccggggagcccggcgg......................	5	0	s10
....ccccggggagcccggcggG.....................	61	1	s10
....ccccggggagcccggcggGg....................	1	1	s10
....cccggggagcccggcggG.....................	87	1	s10
....cccccggggagcccggcg.......................	3	0	s18
....cccccggggagcccggcggG.....................	2	1	s18
....ccccggggagcccggcgg......................	6	0	s18
....ccccggggagcccggcggG.....................	63	1	s18
....cccggggagcccggcggG.....................	85	1	s18
....cccccggggagcccggcggG.....................	1	1	s11
....ccccggggagcccggcgg......................	1	0	s11
....ccccggggagcccggcggG.....................	25	1	s11
....ccccggggagcccggcggGg....................	1	1	s11
....cccggggagcccggcggC.....................	1	1	s11
....cccggggagcccggcggG.....................	60	1	s11
....cccccggggagcccggcggG.....................	1	1	s23
....ccccggggagcccggcgg......................	1	0	s23
....ccccggggagcccggcggG.....................	48	1	s23
....cccggggagcccggcggG.....................	73	1	s23
....cccccggggagcccggcg.......................	1	0	s21
....cccccggggagcccggcgg......................	1	0	s21
....cccccggggagcccggcggG.....................	3	1	s21
....ccccggggagcccggcgg......................	5	0	s21
....ccccggggagcccggcggG.....................	54	1	s21
....ccccggggagcccggcggC.....................	2	1	s21
....ccccggggagcccggcggGg....................	2	1	s21
....cccggggagcccggcggG.....................	70	1	s21
....cccccggggagcccggcggG.....................	5	1	s24
....ccccggggagcccggcgg......................	7	0	s24
....ccccggggagcccggcggG.....................	59	1	s24
....cccggggagcccggcggG.....................	69	1	s24
....ccccggggagcccggcgg......................	1	0	s20
....ccccggggagcccggcggG.....................	49	1	s20
....cccggggagcccggcggG.....................	52	1	s20
