# Supplementary material for: The Identification of Circulating MiRNA in Bovine Serum and Their Potential as Novel Biomarkers of Early Mycobacterium avium subsp paratuberculosis Infection
Source: PLoS One. 2015 Jul 28;10(7):e0134310. doi: 10.1371/journal.pone.0134310 (PMC4517789; doi:10.1371/journal.pone.0134310)
Supplement: S1 File — (ZIP) [file pone.0134310.s008.zip › novel_pdfs/26_16197.pdf]

Provisional ID	:	26_16197
Score total	:	887.3
Score for star read(s)	:	3.9
Score for read counts	:	880.1
Score for mfe	:	2.3
Score for randfold	:	1.6
Score for cons. seed	:	-0.6
Total read count	:	1738
Mature read count	:	1736
Loop read count	:	0
Star read count	:	2

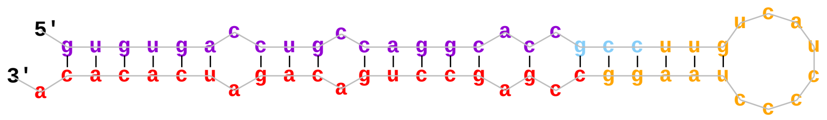

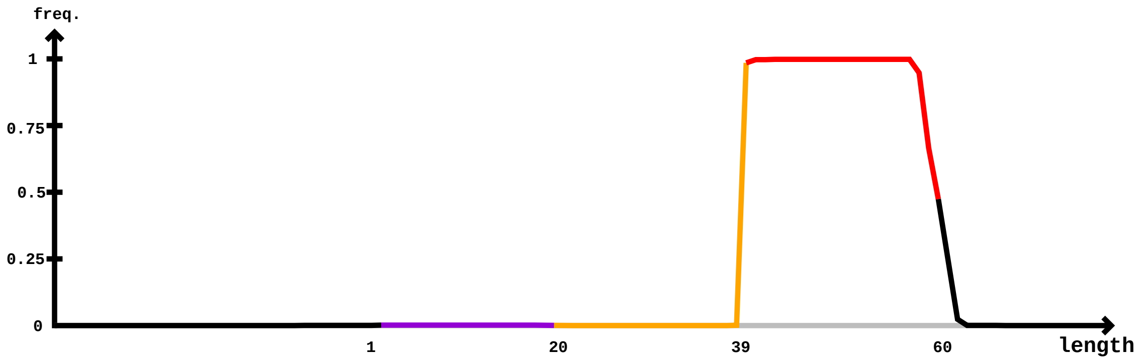


```
                     Star                               Mature
5'- ccaggucugcugugagcagggcaguggccccugugugaccugccaggcaccgccuugucauccccuaaggccgagccugacagaucacacacgggacuugaccuggag   -3'   obs
    ccaggucugcugugagcagggcaguggccccugugugaccugccaggcaccgccuugucauccccuaaggccgagccugacagaucacacacgggacuugaccuggag         exp
    ((((((((((((.......)))))))(((.(((((((.(((.(((((.(.((((((........)))))).).))))).))).)))))))).))).....))))))..   reads   mm   sample
    ......................................................................ccgagccugacagaucac....................   3   0   s19
    ......................................................................ccgaCccugacagaucac....................   1   1   s19
    ......................................................................ccgaUccugacagaucaca...................   1   1   s19
    ......................................................................ccgGgccugacagaucaca...................   1   1   s19
    ......................................................................ccgagccugacagaucaca...................   46   0   s19
    ......................................................................ccUagccugacagaucaca...................   1   1   s19
    ......................................................................ccgaAccugacagaucacac..................   1   1   s19
    ......................................................................ccgagccugacagaucacac..................   30   0   s19
    ......................................................................ccgagccugGcagaucacac..................   1   1   s19
    ......................................................................ccgaCccugacagaucacac..................   1   1   s19
    ......................................................................ccgagccugacagaucacCc..................   1   1   s19
    ......................................................................ccgagccugacagaucacaca.................   36   0   s19
    ......................................................................ccgaCccugacagaucacaca.................   2   1   s19
    ......................................................................ccgagccugacagaucacCca.................   2   1   s19
    ......................................................................ccgagccugacagaucacacac................   1   0   s19
    ......................................................................ccgagccugacagaucacacacA...............   1   1   s19
    .......................................................................cgagccugacagaucacaca.................   1   0   s19

    .....................................................................Cccgagccugacagaucacac..................   1   1   s09
    ......................................................................ccgagccugacagaucac....................   5   0   s09
    ......................................................................ccgagccugacagaucaca...................   15   0   s09
    ......................................................................ccgagccugacagaucacCc..................   1   1   s09
    ......................................................................ccgagccugacagaucacac..................   7   0   s09
    ......................................................................ccUagccugacagaucacaca.................   1   1   s09
    ......................................................................ccgagccugacagaucacaca.................   13   0   s09
    ......................................................................ccgagccugacagaucacacac................   1   0   s09
    ......................................................................ccgagccugacagaucacacaA................   1   1   s09
    .......................................................................cgagccugacagaucacaca.................   1   0   s09

    .....................................................................gccgagccugacagaucacaca.................   1   0   s07
    ......................................................................ccgagccugacagaucac....................   1   0   s07
    ......................................................................ccgagccugacagaucaca...................   9   0   s07
    ......................................................................ccgagccugacagaucacac..................   11   0   s07
```

```
                                Star                                  Mature
ccaggucugcugugagcagggcaguggccccugugugaccugccaggcaccgccuugucauccccuaaggccgagccugacagaucacacacgggacuugaccuggag
......................................................................ccUagccugacagaucacaca.................   1    1    s07
......................................................................ccgGgccugacagaucacaca.................   1    1    s07
......................................................................ccgagccugacagaucacaca.................   9    0    s07
......................................................................ccgaAccugacagaucacaca.................   1    1    s07
......................................................................ccgagccugacagaucacacac................   3    0    s07

......................................................................ccgaAccugacagaucac....................   1    1    s14
......................................................................ccgagccugacagaucac....................   5    0    s14
......................................................................ccgagccugacagaucaca...................   11   0    s14
......................................................................ccgagccugacagaucacac..................   8    0    s14
......................................................................ccgagccugacagaucacaca.................   8    0    s14
......................................................................ccgagccugacagaucacCca.................   1    1    s14
......................................................................ccgagccugacagaucacacac................   1    0    s14
......................................................................ccgGgccugacagaucacacac................   1    1    s14
......................................................................ccgagccugacagaucacacacg...............   1    0    s14
.......................................................................cgagccugacagaucacac..................   1    0    s14

......................................................................ccgGgccugacagaucac....................   1    1    s12
......................................................................ccgagccugacagaucac....................   7    0    s12
......................................................................ccgaCccugacagaucaca...................   3    1    s12
......................................................................ccgagccugacagaucaca...................   56   0    s12
......................................................................ccgagccugacagaucacaA..................   1    1    s12
......................................................................ccgaAccugacagaucacac..................   1    1    s12
......................................................................ccgagccugacagaucacac..................   19   0    s12
......................................................................ccgagccugacagaucacCc..................   1    1    s12
......................................................................ccgagccugacagauUacaca.................   1    1    s12
......................................................................ccgaCccugacagaucacaca.................   3    1    s12
......................................................................cGgagccugacagaucacaca.................   1    1    s12
......................................................................ccgCgccugacagaucacaca.................   1    1    s12
......................................................................ccgagccugacagaucacaca.................   61   0    s12
......................................................................ccUagccugacagaucacaca.................   1    1    s12
......................................................................ccgagccugacagaucacCca.................   2    1    s12
......................................................................ccgagccugacagaucacacaA................   9    1    s12
......................................................................ccgagccugacagaucacacac................   3    0    s12
......................................................................ccgagccugacagaucacacacA...............   3    1    s12

......................................................................ccgagccugacagaucac....................   4    0    s17
......................................................................ccgagccugacagaucaca...................   22   0    s17
......................................................................ccgagccugacagaucacaA..................   3    1    s17
......................................................................ccgagccugacagaucacac..................   19   0    s17
......................................................................ccgagccugacagaucacCca.................   1    1    s17
......................................................................ccUagccugacagaucacaca.................   1    1    s17
......................................................................ccgGgccugacagaucacaca.................   1    1    s17
......................................................................ccgagccugacagaucacaca.................   26   0    s17
......................................................................ccgagccugacagaucacacaA................   1    1    s17
......................................................................ccgagccugacagaucacacac................   8    0    s17
......................................................................ccgagccugacagaucacacacA...............   1    1    s17
.......................................................................cgagccugacagaucacaca.................   1    0    s17
.......................................................................cgagccugacagaucacacacC...............   1    1    s17

................................guAugaccugccaggcac..........................................................   1    1    s02
......................................................................ccgagccugacagaucacC...................   1    1    s02
......................................................................ccgagccugacaUaucaca...................   1    1    s02
......................................................................ccgagccugacagaucaca...................   10   0    s02
......................................................................ccgagccugacagaucacac..................   4    0    s02
......................................................................ccgagccugacagaucacaca.................   9    0    s02
......................................................................ccgagccugacagaucacacaA................   1    1    s02
......................................................................ccgagccugacagaucacacac................   3    0    s02
.......................................................................cgagccugacagaucacaca.................   1    0    s02
.......................................................................cgaUccugacagaucacaca.................   1    1    s02

......................................................................ccUagccugacagaucac....................   1    1    s22
......................................................................ccgaCccugacagaucaca...................   2    1    s22
......................................................................ccgagccugacagaucacC...................   1    1    s22
......................................................................ccgagccugacagaucaca...................   11   0    s22
......................................................................ccgCgccugacagaucaca...................   1    1    s22
......................................................................ccUagccugacagaucaca...................   1    1    s22
......................................................................cUgagccugacagaucacac..................   1    1    s22
......................................................................ccgaAccugacagaucacac..................   1    1    s22
......................................................................ccgagccugacagaucacac..................   9    0    s22
```

```
                                Star                                Mature
ccaggucugcugugagcagggcaguggccccugugugaccugccaggcaccgccuugucauccccuaaggccgagccugacagaucacacacgggacuugaccuggag
......................................................................ccUagccugacagaucacac..................    1    1    s22
......................................................................ccgagccugacagaucacCca.................    3    1    s22
......................................................................ccgagccugacagaucacaca.................    14   0    s22
......................................................................ccgaAccugacagaucacaca.................    1    1    s22
......................................................................ccgaCccugacagaucacaca.................    1    1    s22
......................................................................ccgagccugacagaucacacaA................    3    1    s22
......................................................................ccgagccugacagaucacacaAg...............    1    1    s22

......................................................................ccgagccugacagaucac....................    1    0    s05
......................................................................ccgagccugacagaucaca...................    9    0    s05
......................................................................Gcgagccugacagaucacac..................    1    1    s05
......................................................................ccgagccugacagaucacac..................    3    0    s05
......................................................................ccgagccugacagaucacaca.................    15   0    s05
......................................................................ccgagccugacagaucacacaA................    1    1    s05

......................................................................ccgagccugacagaucac....................    2    0    s16
......................................................................ccgagccugacagaucaca...................    9    0    s16
......................................................................ccgagccuUacagaucacac..................    1    1    s16
......................................................................ccgagccugacagaucacac..................    6    0    s16
......................................................................ccgagccugacagaucacaca.................    11   0    s16
......................................................................ccgagccugacagaucacacac................    5    0    s16
......................................................................ccgagccugacagaucacacacA...............    1    1    s16
......................................................................ccgagccugacagaucacacacC...............    1    1    s16
.......................................................................cgagccugacCgaucacacac................    1    1    s16
.......................................................................cgagccugacagaucacacac................    3    0    s16
.......................................................................cgagAcugacagaucacacac................    1    1    s16

................................gugugaccugccaggcacc.........................................................    1    0    s06
......................................................................ccgagccugacagaucac....................    2    0    s06
......................................................................ccgagccugacagaucaca...................    5    0    s06
......................................................................ccgagccugacagaucacC...................    1    1    s06
......................................................................ccgagccugacagaucacac..................    4    0    s06
......................................................................ccgagccugacagaucacaca.................    12   0    s06
......................................................................ccgagccugacagaucacacac................    1    0    s06

......................................................................ccgagccugacagaucac....................    8    0    s01
......................................................................ccgagccugacagaucaca...................    14   0    s01
......................................................................ccgGgccugacagaucaca...................    1    1    s01
......................................................................ccgagccugacagaucacac..................    13   0    s01
......................................................................ccgCgccugacagaucacac..................    1    1    s01
......................................................................ccgaCccugacagaucacac..................    1    1    s01
......................................................................ccgagccugacagaucacCc..................    2    1    s01
......................................................................cUgagccugacagaucacaca.................    1    1    s01
......................................................................ccgaCccugacagaucacaca.................    1    1    s01
......................................................................ccgagccugacagaucacCca.................    1    1    s01
......................................................................ccgagccugacagaucacaca.................    24   0    s01
......................................................................ccUagccugacagaucacaca.................    1    1    s01
......................................................................ccgagccugacagaucacacac................    3    0    s01
......................................................................ccgagccugacagaucacacaA................    3    1    s01
......................................................................ccgagccugacagaucacacacA...............    3    1    s01
......................................................................ccgagccugacagaucacacacC...............    1    1    s01

......................................................................ccgagccugacagaucac....................    5    0    s04
......................................................................ccgGgccugacagaucac....................    1    1    s04
......................................................................ccgCgccugacagaucac....................    1    1    s04
......................................................................ccgagccugacagaucaca...................    26   0    s04
......................................................................ccgagccugacagaucacC...................    1    1    s04
......................................................................ccgagccugacaUaucaca...................    1    1    s04
......................................................................ccUagccugacagaucaca...................    1    1    s04
......................................................................ccgaAccugacagaucacac..................    1    1    s04
......................................................................cGgagccugacagaucacac..................    1    1    s04
......................................................................ccgaCccugacagaucacac..................    1    1    s04
......................................................................ccgagccugacagaucacac..................    20   0    s04
......................................................................ccgagccugacagaucacaca.................    34   0    s04
......................................................................ccgagccugacagaucacaAa.................    1    1    s04
......................................................................ccgaCccugacagaucacaca.................    1    1    s04
......................................................................ccgagccugacagaucacacac................    4    0    s04
......................................................................ccgagccugacagaucacacaA................    1    1    s04
......................................................................ccgagccugacagaucCcacac................    1    1    s04
......................................................................ccgagccugacagaucacacacA...............    3    1    s04
```

Star **Mature**

ccaggucugcugugagcagggcaguggccccugugugaccugccaggcaccgccuugucauccccuaaggccgagccugacagaucacacacgggacuugaccuggag			
....ccgagccugacagaucacacacC...............	2	1	s04
....ccgagccugacagaucac....................	2	0	s15
....ccgagccugacagaucacC...................	1	1	s15
....ccgagccugacagaucaca...................	8	0	s15
....ccgagccugacagaucacac..................	11	0	s15
....ccgagccugacagaucacaca.................	13	0	s15
....ccgagccugacagaucacCca.................	1	1	s15
....ccgaCccugacagaucacaca.................	1	1	s15
....ccgagccugacagaucacacaA................	3	1	s15
....ccgagccugacagaucacacac................	2	0	s15
....cgagccugacagaucacacac................	1	0	s15
....ccgagccugacagaucac....................	3	0	s13
....ccgagccugacagaucaca...................	3	0	s13
....cUgagccugacagaucaca...................	1	1	s13
....ccgagccugacagaucacac..................	2	0	s13
....agccugacagaucacacacgggac...........	1	0	s13
....ccgagccugacagaucac....................	1	0	s10
....ccgagccugacagaucaca...................	7	0	s10
....ccgGgccugacagaucacac..................	1	1	s10
....ccgagccugacagaucacac..................	14	0	s10
....ccgagccugacagaucacCc..................	2	1	s10
....ccgagccugacagaucacaca.................	14	0	s10
....cUgagccugacagaucacaca.................	1	1	s10
....ccgagccugacagaucacacac................	1	0	s10
....ccgagccugacagaucacacacA...............	1	1	s10
........................uggccccugugugaccugccaggAa...	1	1	s08
....ccgagccugacagaucac....................	3	0	s08
....cUgagccugacagaucac....................	1	1	s08
....ccgagccugacagaucacC...................	1	1	s08
....ccgGgccugacagaucaca...................	1	1	s08
....ccgagccugacagaucaca...................	36	0	s08
....ccgagccugacagaucacac..................	32	0	s08
....ccgagccugacagaucacaA..................	1	1	s08
....ccgGgccugacagaucacac..................	1	1	s08
....ccgagcAugacagaucacaca.................	1	1	s08
....ccgagccugacagaucCcaca.................	1	1	s08
....ccgaCccugacagaucacaca.................	3	1	s08
....ccgagccuAacagaucacaca.................	2	1	s08
....ccUagccugacagaucacaca.................	1	1	s08
....ccgagccugacagaucacaca.................	53	0	s08
....ccgagccugacagaucacCca.................	3	1	s08
....ccgagccugacagaucacacac................	11	0	s08
....cGgagccugacagaucacacac................	1	1	s08
....ccgagccugacagaucacacacA...............	1	1	s08
....cgagccugacagaucacacacA...............	1	1	s08
....cgagccugacagaucacacacC...............	1	1	s08
....ccgagccugacagaucac....................	5	0	s18
....ccgagccugacagaucaca...................	10	0	s18
....ccgagccugacagaucacC...................	1	1	s18
....ccgagccugacagaucacCc..................	1	1	s18
....ccgagccugacagaucacac..................	5	0	s18
....ccgagccugaUagaucacaca.................	1	1	s18
....ccgagccugacagaucacaca.................	17	0	s18
....ccgaCccugacagaucacaca.................	1	1	s18
....ccgagccugacagaucacacaA................	2	1	s18
....ccgagccugacagaucacacac................	2	0	s18
....ccgaCccugacagaucacacac................	2	1	s18
....ccgagccugacagaucacacacA...............	1	1	s18
....ccgagccugacagaucacacacC...............	1	1	s18
....ccUagccugacagaucac....................	1	1	s03
....ccgagccugacagaucac....................	5	0	s03
....ccgagccugacagaucaca...................	18	0	s03
....ccgagccugacagaucacC...................	3	1	s03
....ccgagccugacagaucacac..................	10	0	s03

```
                                  Star                                  Mature
ccaggucugcugugagcagggcaguggccccugugugaccugccaggcaccgccuugucaucccuaaggccgagccugacagaucacacacgggacuugaccuggag
.....................................................................ccgaCccugacagaucacac..................   1    1    s03
.....................................................................ccgaCccugacagaucacaca.................   1    1    s03
.....................................................................ccUagccugacagaucacaca.................   1    1    s03
.....................................................................ccgagccugacagaucacCca.................   1    1    s03
.....................................................................ccgagccugacagaucacaca.................   31   0    s03
.....................................................................ccgaAccugacagaucacaca.................   1    1    s03
.....................................................................ccgagccugacagaucacacac................   11   0    s03
.....................................................................ccgaCccugacagaucacacac................   1    1    s03
.....................................................................ccgagccugacagaucacacaAg...............   1    1    s03
.....................................................................ccgagccugacagaucacacacA...............   5    1    s03
.....................................................................ccgagccugacagaucacacacC...............   1    1    s03

.....................................................................ccgagccugacagaucac....................   7    0    s11
.....................................................................ccgagccugacagaucaca...................   31   0    s11
.....................................................................ccgGgccugacagaucaca...................   1    1    s11
.....................................................................ccgagccugacagaucacCc..................   1    1    s11
.....................................................................ccgagccugacagaucacaA..................   2    1    s11
.....................................................................ccgagccugacagaucacac..................   11   0    s11
.....................................................................ccgagccugacagaucacaca.................   41   0    s11
.....................................................................ccgagccugacagaucacCca.................   3    1    s11
.....................................................................ccgagccugacagaucacacG.................   1    1    s11
.....................................................................ccgagccugacagaucacacaA................   1    1    s11
.....................................................................ccgagccugacagaucacacac................   1    0    s11
.....................................................................ccgagccugacagaucacacacA...............   1    1    s11
......................................................................cgagccugacagaucacaca.................   1    0    s11

.....................................................................ccgagccugacagaucac....................   1    0    s20
.....................................................................ccgagccugacagaucaca...................   14   0    s20
.....................................................................ccgagccugacagaucacC...................   1    1    s20
.....................................................................ccgagccugacagaucacac..................   4    0    s20
.....................................................................ccgagccugacagaucacaca.................   28   0    s20
.....................................................................ccgagccugacagaucacCca.................   2    1    s20
.....................................................................ccgagccugacaCaucacaca.................   1    1    s20
.....................................................................ccgagccugacagaucacacac................   1    0    s20
.....................................................................ccgagccugacagaucacacacA...............   1    1    s20
........................................................................agccugacagaucacacac................   1    0    s20

.....................................................................ccgagccugacagaucac....................   4    0    s24
.....................................................................cUgagccugacagaucaca...................   1    1    s24
.....................................................................ccgagccugacagaucaca...................   41   0    s24
.....................................................................ccgagccugacagaucacac..................   24   0    s24
.....................................................................ccgagccugacagaucacaca.................   61   0    s24
.....................................................................ccUagccugacagaucacaca.................   1    1    s24
.....................................................................ccgagccugacagaucacCca.................   4    1    s24
.....................................................................ccgagccugacagaucacacac................   5    0    s24
.....................................................................ccgagccugacagaucacacacA...............   3    1    s24
......................................................................cgagccugacagaucaca...................   1    0    s24
......................................................................cgagccugacagaucCcacac................   1    1    s24
......................................................................cgagccugacagaucacacacC...............   1    1    s24

.....................................................................ccgagccugacagaucac....................   3    0    s21
.....................................................................ccgGgccugacagaucaca...................   1    1    s21
.....................................................................ccgagccugacagaucacC...................   1    1    s21
.....................................................................ccgagccugacagaucaca...................   15   0    s21
.....................................................................ccgagccugacagaucacac..................   11   0    s21
.....................................................................ccgaCccugacagaucacac..................   1    1    s21
.....................................................................ccgagccugacagauGacaca.................   1    1    s21
.....................................................................ccUagccugacagaucacaca.................   1    1    s21
.....................................................................ccgGgccugacagaucacaca.................   1    1    s21
.....................................................................ccgagccugacagaucacCca.................   2    1    s21
.....................................................................ccgagccugacagaucacaca.................   25   0    s21
.....................................................................ccgagccugacagaucacacacC...............   1    1    s21
.....................................................................ccgagccugacagaucacacacA...............   1    1    s21
......................................................................cgagccugacagaucacacac................   2    0    s21

.....................................................................ccgagccugacagaucac....................   3    0    s23
.....................................................................cUgagccugacagaucaca...................   2    1    s23
.....................................................................ccUagccugacagaucaca...................   1    1    s23
.....................................................................ccgaAccugacagaucaca...................   1    1    s23
.....................................................................ccgagccugacagaucaca...................   30   0    s23
```

```
                                Star                                  Mature
ccaggucugcugugagcagggcaguggccccugugugaccugccaggcaccgccuugucauccccuaaggccgagccugacagaucacacacgggacuugaccuggag
......................................................................ccgaCccugacagaucacac..................   1    1    s23
......................................................................ccgagccugacagaucCcac..................   1    1    s23
......................................................................ccgagccugacagaucacCc..................   3    1    s23
......................................................................ccgGgccugacagaucacac..................   1    1    s23
......................................................................ccgagccugacagaucacac..................   13   0    s23
......................................................................ccgaUccugacagaucacaca.................   1    1    s23
......................................................................ccgaCccugacagaucacaca.................   1    1    s23
......................................................................ccgagccugacagaucacaca.................   34   0    s23
......................................................................ccgagccugacagaucacacC.................   1    1    s23
......................................................................ccgagccugacagaucacCca.................   1    1    s23
......................................................................ccgagccugacagaucacacac................   6    0    s23
```
