# Supplementary material for: The Identification of Circulating MiRNA in Bovine Serum and Their Potential as Novel Biomarkers of Early Mycobacterium avium subsp paratuberculosis Infection
Source: PLoS One. 2015 Jul 28;10(7):e0134310. doi: 10.1371/journal.pone.0134310 (PMC4517789; doi:10.1371/journal.pone.0134310)
Supplement: S1 File — (ZIP) [file pone.0134310.s008.zip › novel_pdfs/3_18517.pdf]

Provisional ID	:	3_18517
Score total	:	1374.1
Score for star read(s)	:	3.9
Score for read counts	:	1367
Score for mfe	:	2.3
Score for randfold	:	1.6
Score for cons. seed	:	-0.6
Total read count	:	2693
Mature read count	:	2681
Loop read count	:	0
Star read count	:	12

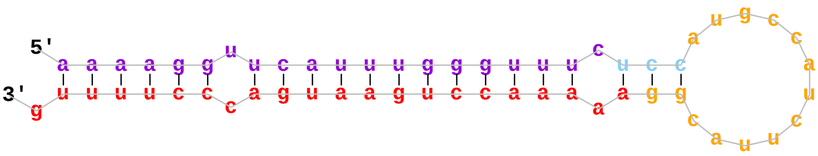

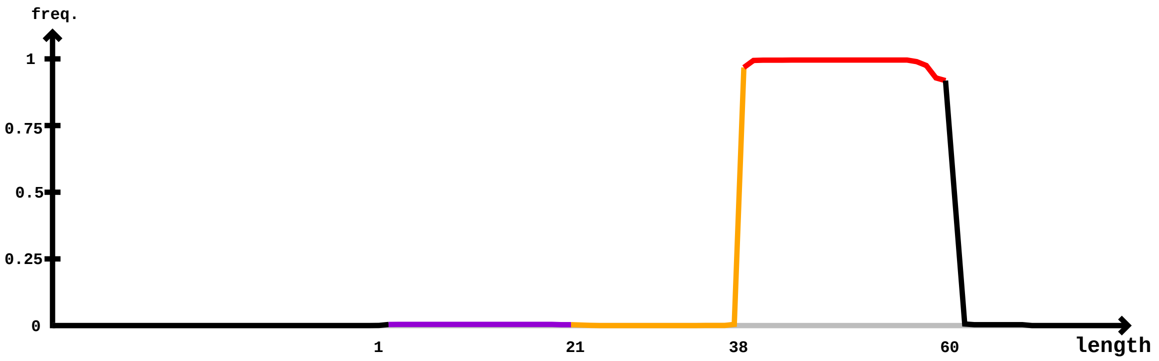

Star **Mature**

```
5'- uuggauuacuuauugcuuuguauuggguuggccaaaagguucauuuggguuucuccaugccaucuuacggaaaaaccugaaugacccuuuuggccagcccaauaauauag   -3'    obs
    uuggauuacuuauugcuuuguauuggguuggccaaaagguucauuuggguuucuccaugccaucuuacggaaaaaccugaaugacccuuuuggccagcccaauaauauag          exp
    ....................(((((((((((((((((((.((((((((((((.(((............))).)))))))))))).)))))))))))))))))))......   reads     mm     sample
    ......................................................................aaaaaccugaaugacccu......................   1         0      s11
    ......................................................................aaaaaccugaaugacccuu.....................   4         0      s11
    ......................................................................aaaaCccugaaugacccuu.....................   1         1      s11
    ......................................................................aaaaCccugaaugacccuuu....................   1         1      s11
    ......................................................................aaaaaccugaaugacccuuu....................   11        0      s11
    ......................................................................aCaaaccugaaugacccuuu....................   1         1      s11
    ......................................................................aaaaaccugaaugacccuuuu...................   1         0      s11
    ......................................................................aaaaaccugaCugacccuuuug..................   3         1      s11
    ......................................................................aaUaaccugaaugacccuuuug..................   3         1      s11
    ......................................................................aaaaaccugaaugacccuuuug..................   47        0      s11
    ......................................................................aUaaaccugaaugacccuuuug..................   1         1      s11
    ......................................................................aaaGaccugaaugacccuuuug..................   1         1      s11
    ......................................................................aaaaaccugaaugacccCuuug..................   1         1      s11
    ......................................................................aaaaCccugaaugacccuuuug..................   5         1      s11
    ......................................................................aaaaaccugaaugacccuuuugg.................   6         0      s11
    ......................................................................aaaaaccugaaugacccuuuugA.................   29        1      s11
    ......................................................................aaaaaccugaaugacccuuuuggccagccc..........   1         0      s11
    .......................................................................aaaaccugaaugacccuuuugg.................   1         0      s11
    .......................................................................aaaaccugaaugacccuuuugA.................   1         1      s11
    ...........................................................................ccugaaugacccuuuugA.................   1         1      s11

    .....................................................................Caaaaaccugaaugacccuuuug..................   1         1      s23
    ......................................................................aaaaaccugaaugacccu......................   1         0      s23
    ......................................................................aaaaaccugaaugacccuu.....................   1         0      s23
    ......................................................................aaaaaccugaaugacccuuu....................   3         0      s23
    ......................................................................aaGaaccugaaugacccuuuug..................   1         1      s23
    ......................................................................aaaaCccugaaugacccuuuug..................   2         1      s23
    ......................................................................aaaaaccugaaugacccuuuug..................   54        0      s23
    ......................................................................aaUaaccugaaugacccuuuug..................   1         1      s23
    ......................................................................aaaaaccugaCugacccuuuug..................   1         1      s23
    ......................................................................aaaaaccugaaugacccCuuug..................   1         1      s23
    ......................................................................aaaaaccugaaugacccuuuugg.................   2         0      s23
    ......................................................................aaaaaccugaaugacccuuuugA.................   29        1      s23
```

Star   Mature

```
uuggauuacuuauugcuuuguauuggguuggccaaaagguucauuuggguuucuccaugccaucuuacggaaaaaccugaaugacccuuuuggccagcccaauaauauag
.......................................................................aaaaccugaaugacccuuuug..................   1   0   s23
.......................................................................aaaaAcugaaugacccuuuugg.................   1   1   s23

......................................................................aaUaaccugaaugacccu......................   1   1   s24
......................................................................aaaaaccugaaugacccuu.....................   2   0   s24
......................................................................aaaaaccugaaugacccuuu....................   1   0   s24
......................................................................aUaaaccugaaugacccuuuug..................   1   1   s24
......................................................................aaaaaccugaaugacccuuuug..................   81  0   s24
......................................................................aaaaaccugaauAacccuuuug..................   1   1   s24
......................................................................aaaaaccugaaugUcccuuuug..................   1   1   s24
......................................................................aaaaUccugaaugacccuuuug..................   2   1   s24
......................................................................aaUaaccugaaugacccuuuug..................   4   1   s24
......................................................................aaaaCccugaaugacccuuuug..................   6   1   s24
......................................................................aaaaaccugaCugacccuuuug..................   5   1   s24
......................................................................aaaaaccugaaugaAccuuuug..................   1   1   s24
......................................................................aaaaaccugaaugacccuuuugg.................   1   0   s24
......................................................................aaaaaccugaaugacccuuuugA.................   65  1   s24
......................................................................aaaaCccugaaugacccuuuugg.................   1   1   s24
......................................................................aaaaaccugaCugacccuuuugg.................   1   1   s24
.......................................................................aaaaccugaaugacccuuuugg.................   5   0   s24

................................caaaagguucauuCggguu...........................................................   1   1   s21
.................................aaaagguucauuuggguuuc.........................................................   1   0   s21
.....................................................................Caaaaaccugaaugacccuuuug..................   1   1   s21
.....................................................................gaaaaaccugaaugacccuuuuggccagccc..........   1   0   s21
......................................................................aaaaaccugaaugacccuu.....................   2   0   s21
......................................................................aaaaaccugaaugacccuuu....................   5   0   s21
......................................................................aaaaaccugaaugacccuuuu...................   1   0   s21
......................................................................aaaaUccugaaugacccuuuug..................   1   1   s21
......................................................................aaaaCccugaaugacccuuuug..................   1   1   s21
......................................................................aaUaaccugaaugacccuuuug..................   2   1   s21
......................................................................aaaaaccugaaugacccuuuug..................   22  0   s21
......................................................................aaaaaccugaaugacccGuuug..................   1   1   s21
......................................................................aaaaaccugaaugacccuuuugA.................   8   1   s21
......................................................................aaaaaccugaaugacccuuuugg.................   1   0   s21
.......................................................................aaaaccugaaugacccuuuug..................   1   0   s21
.......................................................................aaaaccugaaugacUcuuuugg.................   1   1   s21

.................................aaaagguucauuuggguuucuc.......................................................   1   0   s20
.....................................................................gaaaaaccugaaugacccuuuuggccagccc..........   1   0   s20
......................................................................aaaaaccugaaugacccu......................   1   0   s20
......................................................................aaaaaccugaaugacccuu.....................   2   0   s20
......................................................................aaaaaccugaaugacccuuu....................   6   0   s20
......................................................................aaaaaccugaCugacccuuu....................   2   1   s20
......................................................................aaaaaccugaaugacccuuuu...................   1   0   s20
......................................................................aaaaUccugaaugacccuuuug..................   2   1   s20
......................................................................aGaaaccugaaugacccuuuug..................   1   1   s20
......................................................................aaaaaccugaCugacccuuuug..................   1   1   s20
......................................................................aUaaaccugaaugacccuuuug..................   1   1   s20
......................................................................aaaaaccugaaugacccuuuug..................   41  0   s20
......................................................................aaaaaccugaaugacccuuuuA..................   1   1   s20
......................................................................aaaaaccugaaugacccuuuugg.................   2   0   s20
......................................................................aaaaaccugaaugacccuuuuUg.................   1   1   s20
......................................................................aaaaaccugaaugacccuuuugA.................   26  1   s20
.......................................................................aaaaccugaaugacccuuuug..................   2   0   s20
.......................................................................aaaaccugaaugacccuuuugA.................   1   1   s20
.......................................................................aaaaccugaaugacccuuuugg.................   1   0   s20

......................................................................aaaaaccugaaugacccu......................   1   0   s03
......................................................................aaaaaccugaaugacccuuu....................   3   0   s03
......................................................................aaaaaccugaCugacccuuuug..................   4   1   s03
......................................................................aaaaaccugaaugaAccuuuug..................   1   1   s03
......................................................................aaUaaccugaaugacccuuuug..................   1   1   s03
......................................................................aGaaaccugaaugacccuuuug..................   1   1   s03
......................................................................aaaaaccugaaugacccuuuug..................   36  0   s03
......................................................................aaaaCccugaaugacccuuuug..................   2   1   s03
......................................................................aaaaaccugaaugacccuuuugA.................   24  1   s03

.................................aaaagguucauuuggguu...........................................................   1   0   s08
.................................aaaagguucCuuuggguuuc.........................................................   1   1   s08
```

```
                                 Star                                 Mature
uuggauuacuuauugcuuuguauuggguuggccaaaagguucauuuggguuucuccaugccaucuuacggaaaaaccugaaugacccuuuuggccagcccaauaauauag
.................................aaaagguucauuuggguuucuc.......................................................  1    0    s08
......................................................................aaaaaccugaaugacccuu.....................  1    0    s08
......................................................................aGaaaccugaaugacccuuu....................  1    1    s08
......................................................................aaaaCccugaaugacccuuu....................  2    1    s08
......................................................................aaaaaccugaaugacccuuu....................  5    0    s08
......................................................................aaaaaccugaCugacccuuu....................  2    1    s08
......................................................................aaaaaccugaaugacccuuuu...................  1    0    s08
......................................................................aaaaaccugaCugacccuuuug..................  4    1    s08
......................................................................aaaCaccugaaugacccuuuug..................  1    1    s08
......................................................................aaaaaccugaaugUcccuuuug..................  1    1    s08
......................................................................aaUaaccugaaugacccuuuug..................  1    1    s08
......................................................................aaaaCccugaaugacccuuuug..................  2    1    s08
......................................................................aaaaaccugaaugacccuuuug..................  74   0    s08
......................................................................aaaaaccugaaugacccuuuuA..................  1    1    s08
......................................................................aaaaaccugaaugaAccuuuug..................  1    1    s08
......................................................................aaaaaccuAaaugacccuuuug..................  1    1    s08
......................................................................aCaaaccugaaugacccuuuug..................  1    1    s08
......................................................................aaaaaAcugaaugacccuuuug..................  1    1    s08
......................................................................aaaaaccugaaugacccuuuuAg.................  1    1    s08
......................................................................aaUaaccugaaugacccuuuugg.................  1    1    s08
......................................................................aaaaaccugaaugacccuuuugA.................  45   1    s08
......................................................................aaaaaccugaaugacccuuuugg.................  7    0    s08

.....................................................................Caaaaaccugaaugacccuuuug..................  1    1    s10
......................................................................aaaaaccugaaugacccu......................  1    0    s10
......................................................................aaaaaccugaaugCcccuu.....................  1    1    s10
......................................................................aaaaaccugaaugacccuu.....................  3    0    s10
......................................................................aaUaaccugaaugacccuuu....................  2    1    s10
......................................................................aaaaaccugaCugacccuuu....................  1    1    s10
......................................................................aaaaaccugaaugacccuuu....................  13   0    s10
......................................................................aaaaaccugaaugacccuuuu...................  3    0    s10
......................................................................aaaaUccugaaugacccuuuug..................  1    1    s10
......................................................................aaaaaccugaaugaAccuuuug..................  1    1    s10
......................................................................aaaaaccugaaugacccuuuug..................  130  0    s10
......................................................................aaaaaccugaaugacccuuuuU..................  1    1    s10
......................................................................aaaaaccugaaugacccCuuug..................  1    1    s10
......................................................................aGaaaccugaaugacccuuuug..................  1    1    s10
......................................................................aaaaaccugaCugacccuuuug..................  6    1    s10
......................................................................aaUaaccugaaugacccuuuug..................  5    1    s10
......................................................................aaaaCccugaaugacccuuuug..................  6    1    s10
......................................................................aaaaaccugaaugacccuuuugg.................  5    0    s10
......................................................................aaaaaccugaaugacccuuuugA.................  76   1    s10
......................................................................aaaaaccugaaugacccuuuuggccagccc..........  1    0    s10
.......................................................................aaaaccugaaugacccuuuug..................  3    0    s10
.......................................................................aaaaccugaaugacccuuuugg.................  3    0    s10
.......................................................................aaaaAcugaaugacccuuuugg.................  2    1    s10

..................................aaagguucauuuggguuuc.........................................................  1    0    s18
......................................................................aaaaaccugaaugacccu......................  3    0    s18
......................................................................aaaaaccugaaugacccuu.....................  1    0    s18
......................................................................aaUaaccugaaugacccuuu....................  1    1    s18
......................................................................aGaaaccugaaugacccuuu....................  1    1    s18
......................................................................aaaaaccugaaugacccuuu....................  4    0    s18
......................................................................aaaaaccugaaugacccuuuu...................  1    0    s18
......................................................................aaaaaccugaaugacccuuuug..................  27   0    s18
......................................................................aaUaaccugaaugacccuuuug..................  1    1    s18
......................................................................aaaaCccugaaugacccuuuug..................  1    1    s18
......................................................................aaaaaccugaaugacccuuuugg.................  5    0    s18
......................................................................aaaaaccugaaugacccuuuugA.................  24   1    s18
.......................................................................aaaaccugaaugacccuuuug..................  1    0    s18
.......................................................................aaaaccugaaugacccuuuugg.................  1    0    s18

......................................................................aaaaaccugaaugacccuu.....................  2    0    s05
......................................................................aaaaaccugaaugacccuuu....................  2    0    s05
......................................................................aaaaaccugaaugacccuuuu...................  1    0    s05
......................................................................aaaaaccugaaugaAccuuuug..................  3    1    s05
......................................................................aaaaCccugaaugacccuuuug..................  7    1    s05
......................................................................aaaaUccugaaugacccuuuug..................  1    1    s05
......................................................................aaUaaccugaaugacccuuuug..................  3    1    s05
......................................................................aaaaaccugaCugacccuuuug..................  4    1    s05
```

```
                                Star                                Mature
uuggauuacuuauugcuuuguauuggguuggccaaaagguucauuuggguuucuccaugccaucuuacggaaaaaccugaaugacccuuuuggccagcccaauaauauag
......................................................................aaaaGccugaaugacccuuuug..................    1     1    s05
......................................................................aaaaaccugaaugacccuuuug..................    114   0    s05
......................................................................aaaaaccugaaugacccCuuug..................    1     1    s05
......................................................................aaaaaccugaaugacccuuuugg.................    5     0    s05
......................................................................aaaaaccugaaugacccuuuugC.................    1     1    s05
......................................................................aaaaaccugaaugaAccuuuugg.................    1     1    s05
......................................................................Caaaaccugaaugacccuuuugg.................    1     1    s05
......................................................................aaaaaccugaaugacccuuuugA.................    59    1    s05
......................................................................aaaaaccugaaugacccuuuuggA................    1     1    s05
.......................................................................aaaaAcugaaugacccuuuugg.................    1     1    s05
.......................................................................aaaaccugaaugacccuuuugg.................    4     0    s05
.......................................................................aaaaccugaaugacccuuuuggA................    1     1    s05
........................................................................aaaccugaaugacccuuuug..................    1     0    s05

......................................................................aaaaaccugaaugacccu......................    1     0    s16
......................................................................aaaaaccugaaugacccuu.....................    1     0    s16
......................................................................aaaaaccugaaugacccuuu....................    1     0    s16
......................................................................aaaaCccugaaugacccuuuug..................    1     1    s16
......................................................................aaUaaccugaaugacccuuuug..................    1     1    s16
......................................................................aaaaaccugaaugacccuuuug..................    11    0    s16
......................................................................aaaaaccugaaugacccuuuugA.................    9     1    s16

.................................aaaagguucauuuggguu...........................................................    1     0    s22
......................................................................aaaaCccugaaugacccuu.....................    1     1    s22
......................................................................aaaaaccugaaugacccuu.....................    1     0    s22
......................................................................aaaaaccugaaugacccuuu....................    5     0    s22
......................................................................aaaaaccugaaugacccuuuu...................    5     0    s22
......................................................................aaUaaccugaaugacccuuuu...................    1     1    s22
......................................................................aaaaCccugaaugacccuuuug..................    1     1    s22
......................................................................aUaaaccugaaugacccuuuug..................    1     1    s22
......................................................................Naaaaccugaaugacccuuuug..................    1     1    s22
......................................................................aaUaaccugaaugacccuuuug..................    1     1    s22
......................................................................aaaaaccugaaugacccuuuug..................    73    0    s22
......................................................................aaaaaccugaCugacccuuuug..................    2     1    s22
......................................................................aaaaaccugaaugacccuuuugg.................    2     0    s22
......................................................................aaaaaccugaaugacccuuuugA.................    39    1    s22
......................................................................aaaaaccugaCugacccuuuugg.................    1     1    s22
.......................................................................aaaaAcugaaugacccuuuug..................    1     1    s22
.......................................................................aaUaccugaaugacccuuuugg.................    1     1    s22
.......................................................................aaaaAcugaaugacccuuuugg.................    1     1    s22
.......................................................................aaaaccugaaugacccuuuugg.................    3     0    s22

.................................aaaagguucauuuggguuuc.........................................................    1     0    s06
......................................................................aaaaaccugaaugacccuu.....................    1     0    s06
......................................................................aaaaaccugaaugacccuuu....................    2     0    s06
......................................................................aaaaCccugaaugacccuuuug..................    2     1    s06
......................................................................aaaaaccugaCugacccuuuug..................    1     1    s06
......................................................................aaaaaccugaaugacccuuuug..................    41    0    s06
......................................................................aaaaaccugaaugacccuuuugA.................    33    1    s06
......................................................................aaaaaccugaaugacccuuuugg.................    1     0    s06
......................................................................aaaaaccugaaugacccuuuuggA................    1     1    s06

.................................aaUagguucauuuggguuuc.........................................................    1     1    s17
.................................aaaagguucauuuggguuuc.........................................................    1     0    s17
......................................................................aaaaaccugaaugacccuu.....................    1     0    s17
......................................................................aaaaaccugaCugacccuu.....................    1     1    s17
......................................................................aaUaaccugaaugacccuuu....................    2     1    s17
......................................................................aaaaCccugaaugacccuuu....................    1     1    s17
......................................................................aaaaaccugaaugacccuuu....................    6     0    s17
......................................................................aaaaaccugaaugacccuuuu...................    4     0    s17
......................................................................aaaaaccugaaugacccuuuug..................    67    0    s17
......................................................................aaUaaccugaaugacccuuuug..................    5     1    s17
......................................................................aaaaaccugaCugacccuuuug..................    1     1    s17
......................................................................aaaaCccugaaugacccuuuug..................    4     1    s17
......................................................................aaaaaccugaaugacccuuuugg.................    1     0    s17
......................................................................aaaaaccugaaugacccuuuugA.................    24    1    s17
.......................................................................aaaaccugaaugacccuuuugg.................    3     0    s17

......................................................................aaaaaccugaaugacccuuu....................    1     0    s02
......................................................................aaaaaccugaCugacccuuuug..................    1     1    s02
```

Star Mature

```
uuggauuacuuauugcuuuguauuggguuggccaaaagguucauuuggguuucuccaugccaucuuacggaaaaaccugaaugacccuuuuggccagcccaauaauauag
......................................................................aUaaaccugaaugacccuuuug..................   1    1    s02
......................................................................aaaaCccugaaugacccuuuug..................   3    1    s02
......................................................................aaUaaccugaaugacccuuuug..................   1    1    s02
......................................................................aaaaaccugaaugacccuuuug..................   37   0    s02
......................................................................aaaaaccugaaugacccuuuugA.................   26   1    s02
......................................................................aaaaaccugaaugacccuuuugg.................   1    0    s02
.......................................................................aaaaccugaaugacccuuuugg.................   2    0    s02
.......................................................................aaaaccugaaugacccuuuuggA................   1    1    s02
.......................................................................aaaaccugaaugacccuuuuggc................   1    0    s02
........................................................................aaaccugaaugacccuuuuggc................   1    0    s02

......................................................................aaaaaccugaaugacccuuuug..................   7    0    s13
......................................................................aGaaaccugaaugacccuuuug..................   1    1    s13
......................................................................aaaaaccugaaugacccuuuugg.................   1    0    s13
......................................................................aaaaaccugaaugacccuuuugA.................   12   1    s13
.......................................................................aaaaAcugaaugacccuuuugg.................   1    1    s13

......................................................................aaaaaccugaaugacccu......................   1    0    s15
......................................................................aaaaCccugaaugacccuuu....................   1    1    s15
......................................................................aaaaaccugaaugacccuuu....................   1    0    s15
......................................................................aaaaaccugaCugacccuuuug..................   2    1    s15
......................................................................aCaaaccugaaugacccuuuug..................   1    1    s15
......................................................................aaaaCccugaaugacccuuuug..................   2    1    s15
......................................................................aaaaaccugaaugacccuuuug..................   41   0    s15
......................................................................aaaaaccugaaugacccuuuugg.................   2    0    s15
......................................................................aaaaCccugaaugacccuuuugg.................   1    1    s15
......................................................................aaaaaccugaaugacccuuuugA.................   26   1    s15
.......................................................................aaaaccugaaugacccuuuug..................   1    0    s15
.......................................................................aaaaccugaaugaAccuuuugg.................   2    1    s15

.................................aaaagguucauuuggguuuc.........................................................   1    0    s04
.....................................................................Caaaaaccugaaugacccuuuug..................   1    1    s04
.....................................................................gaaaaaccugCaugacccuuuuggccagccc..........   1    1    s04
......................................................................aaaaaccugaaugacccuuu....................   3    0    s04
......................................................................aaaaCccugaaugacccuuu....................   1    1    s04
......................................................................aaaaaccugaaugacccuuuu...................   2    0    s04
......................................................................aaaaCccugaaugacccuuuug..................   1    1    s04
......................................................................aaaaaccugaCugacccuuuug..................   1    1    s04
......................................................................aaUaaccugaaugacccuuuug..................   1    1    s04
......................................................................aaaaaccugaaugacccuuuug..................   27   0    s04
......................................................................aaaaaccugaaugacccuuuugA.................   18   1    s04
......................................................................aaaaaccugaaugacccuuuugg.................   2    0    s04
.......................................................................aaaaccugaaugaAccuuuug..................   1    1    s04
.......................................................................aaaaccugaaugacccuuuug..................   1    0    s04

.....................................................................Caaaaaccugaaugacccuuuu...................   1    1    s01
......................................................................aaaaaccugaaugacccuu.....................   2    0    s01
......................................................................aaaaaccugaCugacccuuu....................   1    1    s01
......................................................................aaaaaccugaaugacccuuu....................   1    0    s01
......................................................................aaaaaccugaCugacccuuuug..................   2    1    s01
......................................................................aaaGaccugaaugacccuuuug..................   1    1    s01
......................................................................aaaaaccugaaugacccuuuug..................   25   0    s01
......................................................................aaUaaccugaaugacccuuuug..................   3    1    s01
......................................................................aaaaaccugaaugacccuuuuA..................   1    1    s01
......................................................................aaaaaccugaaugacccuuuugg.................   5    0    s01
......................................................................aaaaaccugaaugacccuuuugA.................   11   1    s01
.......................................................................aaaaccugaaugacccuuuug..................   1    0    s01

..................................................................Gcggaaaaaccugaaugacccuuuug..................   1    1    s12
.....................................................................Aaaaaaccugaaugacccuuuug..................   1    1    s12
......................................................................aaaaaccugaaugacGcu......................   1    1    s12
......................................................................aaaaaccugaaugacccu......................   1    0    s12
......................................................................aaaaaccugaCugacccuu.....................   1    1    s12
......................................................................aaaaaccugaaugacccuu.....................   5    0    s12
......................................................................aaaaaccugaaugacccuuu....................   14   0    s12
......................................................................aaaUaccugaaugacccuuu....................   1    1    s12
......................................................................aaaaCccugaaugacccuuuu...................   1    1    s12
......................................................................aaaaaccugaaugacccuuuu...................   1    0    s12
......................................................................aaaaaccugaaugacGcuuuug..................   1    1    s12
......................................................................aaUaaccugaaugacccuuuug..................   4    1    s12
```

Star **Mature**

```
uuggauuacuuauugcuuuguauuggguuggccaaaagguucauuuggguuucuccaugccaucuuacggaaaaaccugaaugacccuuuuggccagcccaauaaauauag
......................................................................aaaaaccugaCugacccuuuug...................   4     1    s12
......................................................................aaaCaccugaaugacccuuuug...................   1     1    s12
......................................................................aaaaaAcugaaugacccuuuug...................   1     1    s12
......................................................................aaaaCccugaaugacccuuuug...................   5     1    s12
......................................................................aaaGaccugaaugacccuuuug...................   3     1    s12
......................................................................aaaaGccugaaugacccuuuug...................   1     1    s12
......................................................................aUaaaccugaaugacccuuuug...................   2     1    s12
......................................................................aaaaaccugaaugacccuuuug...................   121   0    s12
......................................................................aaaaUccugaaugacccuuuug...................   1     1    s12
......................................................................aaaaaccugaaugaAccuuuugg..................   1     1    s12
......................................................................aaaaaccugaaugacccuuuugg..................   4     0    s12
......................................................................aaaaaccugaaugacccuuuugA..................   57    1    s12
.......................................................................aaaaAcugaaugacccuu......................   1     1    s12
.......................................................................aaaaccugaaugacccuu......................   1     0    s12
.......................................................................aaaaccugaaugacccuuuug...................   1     0    s12
.......................................................................aaaaccugaaugacccuuuugg..................   5     0    s12

......................................................................aaaaaccugaaugacccuu.....................   1     0    s07
......................................................................aaaaaccugaaugacccuuu....................   4     0    s07
......................................................................aCaaaccugaaugacccuuuug...................   1     1    s07
......................................................................aaaaaccGgaaugacccuuuug...................   1     1    s07
......................................................................aaaaCccugaaugacccuuuug...................   1     1    s07
......................................................................aaaaaccugaaugacccuuuug...................   65    0    s07
......................................................................aUaaaccugaaugacccuuuug...................   2     1    s07
......................................................................aaUaaccugaaugacccuuuug...................   4     1    s07
......................................................................aaaaaccugaaugacccuuuugA..................   28    1    s07
......................................................................aaaaaccugaaugacccuuuugg..................   1     0    s07
.......................................................................aaaaAcugaaugacccuuuugg..................   1     1    s07
.......................................................................aaaaccugaaugaAccuuuugg..................   1     1    s07
........................................................................aaaccugaaugacccuuuuggccagccc...........   1     0    s07

......................................................................aaaaaccugaaugacccuuu....................   1     0    s14
......................................................................Uaaaaccugaaugacccuuuug...................   1     1    s14
......................................................................aaaaaccugaaugacccuuuug...................   21    0    s14
......................................................................aaaaCccugaaugacccuuuug...................   2     1    s14
......................................................................aaaaaccugaCugacccuuuug...................   1     1    s14
......................................................................aaaaaccugaaugacccuuuugA..................   9     1    s14
......................................................................aaaaaccugaaugacccuuuugg..................   2     0    s14
......................................................................aaaaaccugaaugacccuuuuggccagccc...........   1     0    s14
.......................................................................aaaaAcugaaugacccuuuug...................   1     1    s14
.......................................................................aaaaccugaaugacccuuuugg..................   1     0    s14

.....................................................................Caaaaaccugaaugacccuuuug...................   1     1    s19
......................................................................aaaaaccugaaugacccu......................   3     0    s19
......................................................................aaaaaccugaaugacccuu.....................   3     0    s19
......................................................................aaaaCccugaaugacccuu.....................   1     1    s19
......................................................................aaaaaccugaaugacccuuu....................   10    0    s19
......................................................................aaaaaccugaCugacccuuuug...................   7     1    s19
......................................................................aaaaaccugaaugaAccuuuug...................   1     1    s19
......................................................................aaaCaccugaaugacccuuuug...................   2     1    s19
......................................................................aGaaaccugaaugacccuuuug...................   1     1    s19
......................................................................aaaaaccugaaugacccuuuug...................   134   0    s19
......................................................................aaUaaccugaaugacccuuuug...................   4     1    s19
......................................................................aaaGaccugaaugacccuuuug...................   1     1    s19
......................................................................aUaaaccugaaugacccuuuug...................   2     1    s19
......................................................................aaaaCccugaaugacccuuuug...................   5     1    s19
......................................................................aaaaUccugaaugacccuuuug...................   1     1    s19
......................................................................aaaaaccugaaugacccuuuugA..................   83    1    s19
......................................................................aaUaaccugaaugacccuuuugg..................   1     1    s19
......................................................................aaaaCccugaaugacccuuuugg..................   1     1    s19
......................................................................aaaaaccugaaugacccuCuugg..................   1     1    s19
......................................................................aaaaaccugaaugacccuuuugg..................   5     0    s19
.......................................................................aaaaAcugaaugacccuuuu....................   1     1    s19
.......................................................................aaaaAcugaaugacccuuuugg..................   2     1    s19
.......................................................................aaUaccugaaugacccuuuugg..................   1     1    s19
.......................................................................aaaaccugaaugacccuuuugA..................   1     1    s19
.......................................................................aaaaccugaaugacccuuuugg..................   3     0    s19

.....................................................................Caaaaaccugaaugacccuuu.....................   1     1    s09
......................................................................aaaaaccugaaugacccuuu....................   1     0    s09
```

Star Mature

```
uuggauuacuuauugcuuuguauuggguuggccaaaagguucauuuggguuucuccaugccaucuuacggaaaaaccugaaugacccuuuuggccagcccaauaauauag
.......................................................................aaaaaccugaaugacccuuuu..................   1    0    s09
.......................................................................aaUaaccugaaugacccuuuug.................   1    1    s09
.......................................................................aaaaCccugaaugacccuuuug.................   1    1    s09
.......................................................................aaaaaccugaaugacccuuuug.................   21   0    s09
.......................................................................aaaaaccugaCugacccuuuug.................   1    1    s09
.......................................................................aaaaaccugaaugacccuuuugA................   15   1    s09
.......................................................................aaaaCccugaaugacccuuuugg................   1    1    s09
.......................................................................aaaaaccugaaugacccuuuuggA...............   1    1    s09
.......................................................................aaaaaccugaaugacccuuuuggccagccc..........   2    0    s09
```
