# Supplementary material for: The Identification of Circulating MiRNA in Bovine Serum and Their Potential as Novel Biomarkers of Early Mycobacterium avium subsp paratuberculosis Infection
Source: PLoS One. 2015 Jul 28;10(7):e0134310. doi: 10.1371/journal.pone.0134310 (PMC4517789; doi:10.1371/journal.pone.0134310)
Supplement: S1 File — (ZIP) [file pone.0134310.s008.zip › novel_pdfs/4_19879.pdf]

Provisional ID	:	4_19879
Score total	:	5.1
Score for star read(s)	:	-1.3
Score for read counts	:	0
Score for mfe	:	1.8
Score for randfold	:	1.6
Score for cons. seed	:	3
Total read count	:	682
Mature read count	:	682
Loop read count	:	0
Star read count	:	0

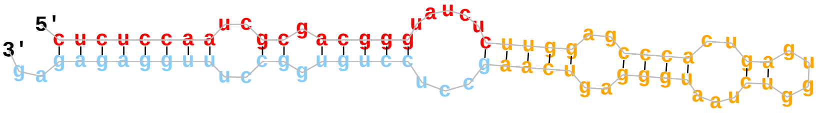

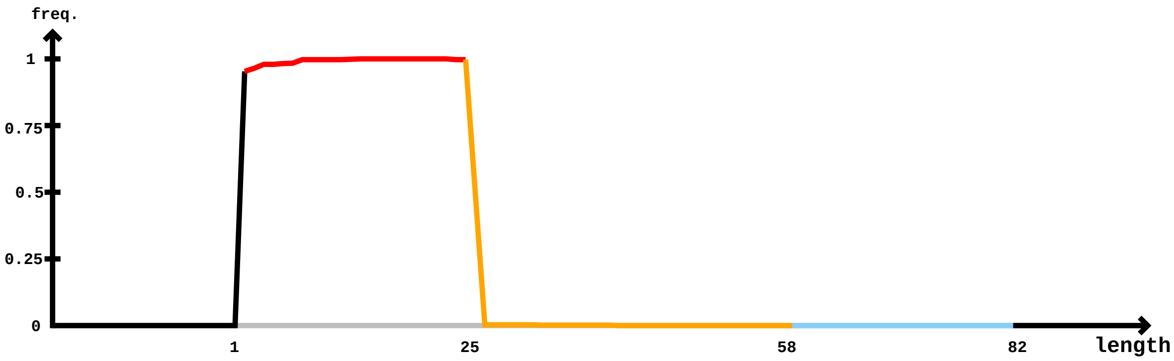

Mature Star

```
5'- gcaaagaacuccgcuauccucuccaaucgcgacggguaucucuuggagcccacugaguggucuaaugggagucaagccuccuguggccuuuggagagaggacacgggauuac   -3'   exp
    .........((((...((((((((((..((.(((((.....(((((..((((..((....))...))))..)))))...))))).))..)))))))).))...)))).....   reads   mm   sample
    ..................cucucGaaucgcgacggguaucuc......................................................................   38      1    s10
    ....................cuccaaucgcgacggguaucuc......................................................................   1       0    s10
    .......................caauAgcgacggguaucuc......................................................................   1       1    s10
    ........................aaucgcgacggguaucuc......................................................................   1       0    s10

    ..................cucucGaaucgcgacggguaucuc......................................................................   30      1    s08
    ...................ucuccaaucgcgacggguaucuc......................................................................   1       0    s08
    ........................aaucgcgacggguaucuc......................................................................   1       0    s08

    ..................cucucGaaucgcgacggguaucuc......................................................................   15      1    s18
    ....................cGccaaucgcgacggguaucuc......................................................................   1       1    s18

    ..................cucuccaaucgcUacggguaucuc......................................................................   1       1    s03
    ..................cucucGaaucgcgacggguaucuc......................................................................   30      1    s03

    ..................cucucGaaucgcgacggguaucuc......................................................................   25      1    s11
    ....................cuccaaucgcgacggguaucuc......................................................................   1       0    s11

    ..................cucucGaaucgcgacggguauc........................................................................   1       1    s20
    ..................cucucGaaucgcgacggguaucuc......................................................................   23      1    s20

    ..................cucucGaaucgcgacggguaucuc......................................................................   34      1    s23
    ...................ucuccaaucgcgacggguaucuc......................................................................   1       0    s23
    ....................cuccaaucgcgacggguaucuc......................................................................   3       0    s23
    ....................cuUcaaucgcgacggguaucuc......................................................................   1       1    s23
    ........................aaucgcgacggguaucuc......................................................................   1       0    s23
    ..............................gacggguaucucuuggagc...............................................................   1       0    s23

    ..................cucucGaaucgcgacggguaucuc......................................................................   8       1    s24
    ........................aaAcgcgacggguaucuc......................................................................   1       1    s24

    ..................cucucGaaucgcgacggguaucuc......................................................................   33      1    s21
    ...................ucuccaaucgcgacggguaucuc......................................................................   1       0    s21

    ..................cucucGaaucgcgacggguaucuc......................................................................   14      1    s19
```

```
                  Mature                                                  Star
gcaaagaacuccgcuauccucuccaaucgcgacggguaucucuuggagcccacugaguggucuaaugggagucaagccuccuguggccuuuggagagaggacacgggauuac
...................ucuccaaucgcgacggguaucuc......................................................................   1    0    s19

..................cucucGaaucgcgacggguaucuc......................................................................   38   1    s09
...................ucuccaaucgcgacggguaucuc......................................................................   1    0    s09

..................cucucGaaucgcgacggguaucuc......................................................................   18   1    s07

..................cucucGaaucgcgacggguauc........................................................................   1    1    s14
..................cucucGaaucgcgacggguaucuc......................................................................   48   1    s14
...................ucAccaaucgcgacggguaucuc......................................................................   1    1    s14
....................cuccCaucgcgacggguaucuc......................................................................   1    1    s14
....................cuccaaucgcgacggguaucuc......................................................................   1    0    s14

..................cucucGaaucgcgacggguaucuc......................................................................   26   1    s12
....................cuccaaucgcgacggguaucuc......................................................................   1    0    s12

..................cucucGaaucgcgacggguaucuc......................................................................   9    1    s02
...................ucuccaaucgcgacggguaucuc......................................................................   1    0    s02

..................cucucGaaucgcgacggguaucuc......................................................................   26   1    s17

..................cucucGaaucgcgacggguaucuc......................................................................   23   1    s16

..................cucucGaaucgcgacggguaucuc......................................................................   14   1    s05
........................aaucgcgacggguaucuc......................................................................   1    0    s05

..................cucucGaaucgcgacggguaucuc......................................................................   12   1    s22

..................cucucGaaucgcgacggguaucuc......................................................................   19   1    s06
........................aaucgcgacggguaucuc......................................................................   2    0    s06

..................cucucGaaucgcgacggguaucuc......................................................................   46   1    s01
......................ccaaucgcgacUgguaucuc......................................................................   1    1    s01
......................ccaaucgcgacggguaucuc......................................................................   1    0    s01
.............................cgacggguaucucuuggagcccacugGg.......................................................   1    1    s01

..................cucucGaaucgcgacggguaucuc......................................................................   72   1    s15
...................ucuccaaucgcgacggguaucuc......................................................................   1    0    s15

..................cucucGaaucgcgacggguaucuc......................................................................   38   1    s04
........................aaucgcgacggguaucuc......................................................................   1    0    s04
........................aaucgcgacgggGaucuc......................................................................   1    1    s04

..................cucucGaaucgcgacggguaucuc......................................................................   10   1    s13
```
