# Supplementary material for: The Identification of Circulating MiRNA in Bovine Serum and Their Potential as Novel Biomarkers of Early Mycobacterium avium subsp paratuberculosis Infection
Source: PLoS One. 2015 Jul 28;10(7):e0134310. doi: 10.1371/journal.pone.0134310 (PMC4517789; doi:10.1371/journal.pone.0134310)
Supplement: S1 File — (ZIP) [file pone.0134310.s008.zip › novel_pdfs/28_17046.pdf]

Provisional ID	:	28_17046
Score total	:	5.3
Score for star read(s)	:	-1.3
Score for read counts	:	0
Score for mfe	:	2
Score for randfold	:	1.6
Score for cons. seed	:	3
Total read count	:	3743
Mature read count	:	3743
Loop read count	:	0
Star read count	:	0

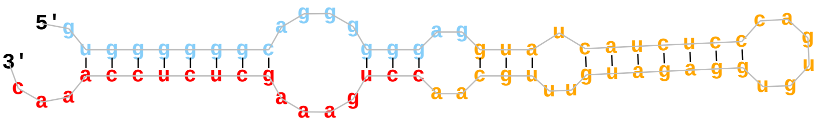

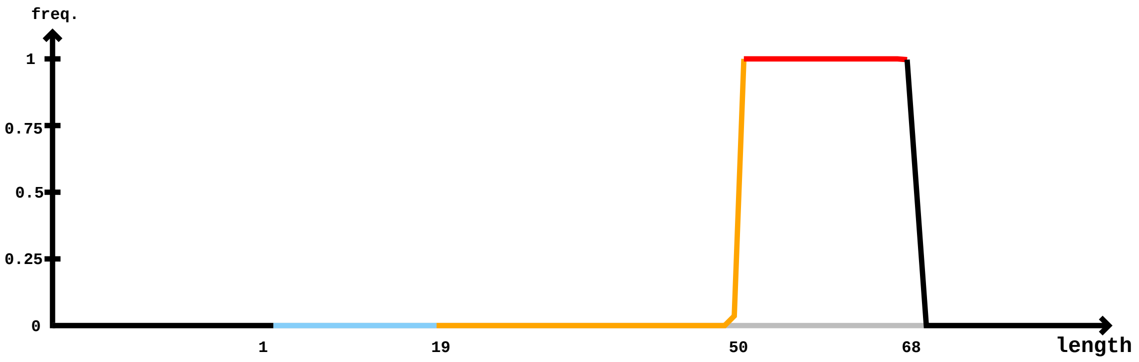


```
                     Star                                           Mature
5'- guccccauggaguugguaggguggggggcaggggggagguaucaucucccaguguggagauguuugcaaccugaaagcucuccaaaccccaugcuauugguuguau   -3'    exp
    ...((((......(((((((((((((((((....(((..(((.(((((((......)))))))..)))..)))....)))))))..))))).))))).))))......   reads   mm   sample
    .....................................................................Gccugaaagcucuccaaac...................   1       1    s02
    ......................................................................ccugUaagcucuccaaac...................   1       1    s02
    ......................................................................ccGgaaagcucuccaaac...................   85      1    s02

    .....................................................................accGgaaagcucuccaaac...................   7       1    s17
    .....................................................................Gccugaaagcucuccaaac...................   6       1    s17
    ......................................................................ccGgaaagcucuccaaac...................   134     1    s17
    ......................................................................ccugaaagcucuccaaac...................   1       0    s17

    .....................................................................accGgaaagcucuccaaac...................   1       1    s05
    ......................................................................ccGgaaagcucuccaaac...................   41      1    s05
    ......................................................................ccugaaagcucuccaaCc..................   1       1    s05

    .....................................................................Gccugaaagcucuccaaac...................   4       1    s16
    ......................................................................ccuCaaagcucuccaaac...................   1       1    s16
    ......................................................................ccugaaagcucuccaaac...................   1       0    s16
    ......................................................................ccGgaaagcucuccaaac...................   103     1    s16

    .....................................................................accGgaaagcucuccaaa....................   1       1    s22
    .....................................................................Gccugaaagcucuccaaa....................   1       1    s22
    .....................................................................accGgaaagcucuccaaac...................   1       1    s22
    .....................................................................Gccugaaagcucuccaaac...................   13      1    s22
    ......................................................................ccugaaagcucuccaaac...................   7       0    s22
    ......................................................................ccGgaaagcucuccaaac...................   601     1    s22
    ......................................................................ccGgaaagcucuccaaacc..................   1       1    s22

    .....................................................................accGgaaagcucuccaaac...................   8       1    s06
    .....................................................................Gccugaaagcucuccaaac...................   3       1    s06
    .....................................................................Uccugaaagcucuccaaac...................   1       1    s06
    ......................................................................ccugUaagcucuccaaac...................   1       1    s06
    ......................................................................ccGgaaagcucuccaaac...................   676     1    s06
    ......................................................................ccugaaagcucuccaaac...................   9       0    s06
    ......................................................................ccGgaaagcucuccaaacc..................   1       1    s06

    .....................................................................accGgaaagcucuccaaac...................   3       1    s01
```

```
                                     Star                                                   Mature
guccccauggaguugguagggguggggggcaggggggagguaucaucucccaguguggagauguuugcaaccugaaagcucuccaaaccccaugcuauuggguuguau
.....................................................................Gccugaaagcucuccaaac....................    2     1    s01
......................................................................ccGgaaagcucuccaaac....................    74    1    s01
......................................................................ccugaaagcCcuccaaac....................    1     1    s01

......................................................................ccugaaagcucuccaaac....................    1     0    s04
......................................................................ccGgaaagcucuccaaac....................    73    1    s04

.....................................................................accGgaaagcucuccaaac....................    1     1    s15
.....................................................................Gccugaaagcucuccaaac....................    2     1    s15
......................................................................ccGgaaagcucuccaaac....................    97    1    s15
......................................................................ccugaaagcucuccaCac....................    1     1    s15
......................................................................ccugaaagcucuccCaac....................    1     1    s15
......................................................................ccuCaaagcucuccaaac....................    1     1    s15

.....................................................................accGgaaagcucuccaaac....................    2     1    s13
.....................................................................Gccugaaagcucuccaaac....................    2     1    s13
......................................................................ccugaaagcucuccaaac....................    2     0    s13
......................................................................ccGgaaagcucuccaaac....................    73    1    s13

.....................................................................accugaaagcucuccaaa.....................    1     0    s12
.....................................................................accGgaaagcucuccaaac....................    4     1    s12
.....................................................................Gccugaaagcucuccaaac....................    5     1    s12
......................................................................ccGgaaagcucuccaaac....................    106   1    s12

.....................................................................Gccugaaagcucuccaaac....................    3     1    s07
......................................................................ccugaaagcucuccaaac....................    2     0    s07
......................................................................ccGgaaagcucuccaaac....................    106   1    s07
......................................................................ccugGaagcucuccaaac....................    1     1    s07

.....................................................................Gccugaaagcucuccaaa.....................    1     1    s14
.....................................................................Gccugaaagcucuccaaac....................    3     1    s14
......................................................................ccGgaaagcucuccaaac....................    107   1    s14
......................................................................ccugaaagcucuccaaac....................    1     0    s14

.....................................................................accGgaaagcucuccaaac....................    1     1    s19
......................................................................ccGgaaagcucuccaaac....................    32    1    s19

.....................................................................accGgaaagcucuccaaa.....................    1     1    s09
.....................................................................accGgaaagcucuccaaac....................    1     1    s09
.....................................................................Gccugaaagcucuccaaac....................    5     1    s09
......................................................................ccGgaaagcucuccaaac....................    78    1    s09
......................................................................ccugaaagcucuccaaac....................    1     0    s09

.....................................................................accGgaaagcucuccaaac....................    1     1    s11
.....................................................................Gccugaaagcucuccaaac....................    2     1    s11
......................................................................ccugaaagcucuccaaac....................    4     0    s11
......................................................................ccGgaaagcucuccaaac....................    62    1    s11

.....................................................................Gccugaaagcucuccaaac....................    4     1    s20
.....................................................................accugaaagcucuccaaac....................    1     0    s20
.....................................................................accGgaaagcucuccaaac....................    3     1    s20
......................................................................ccGgaaagcucuccaaac....................    175   1    s20
......................................................................ccugaaagcucuccaaac....................    5     0    s20

.....................................................................accGgaaagcucuccaaa.....................    1     1    s23
.....................................................................accGgaaagcucuccaaac....................    9     1    s23
.....................................................................Gccugaaagcucuccaaac....................    2     1    s23
......................................................................ccugaaagcucuccaaac....................    1     0    s23
......................................................................ccGgaaagcucuccaaac....................    179   1    s23

.....................................................................accGgaaagcucuccaaa.....................    1     1    s21
.....................................................................Gccugaaagcucuccaaac....................    3     1    s21
.....................................................................accGgaaagcucuccaaac....................    4     1    s21
......................................................................ccugaaagcucuccaaac....................    2     0    s21
......................................................................ccGgaaagcucuccaaac....................    100   1    s21

.....................................................................Gccugaaagcucuccaaa.....................    1     1    s24
.....................................................................accGgaaagcucuccaaac....................    2     1    s24
......................................................................ccugaaagcucuccaaac....................    1     0    s24
......................................................................ccGgaaagcucuccaaac....................    81    1    s24
```

```
                     Star                                                Mature
guccccauggaguugguagggguggggggcaggggggagguaucaucucccaguguggagauguuugcaaccugaaagcucuccaaaccccaugcuauuggguuguau

....................................................................accGgaaagcucuccaaa......................    1    1    s03
......................................................................ccugaaagcucuccaaac....................    2    0    s03
......................................................................ccGgaaagcucuccaaac....................    52   1    s03

....................................................................accGgaaagcucuccaaa......................    2    1    s10
.....................................................................Gccugaaagcucuccaaac....................    2    1    s10
....................................................................accGgaaagcucuccaaac....................    10   1    s10
......................................................................ccGgaaagcucuccaaac....................    294  1    s10
......................................................................ccugaaagcucuccaaac....................    4    0    s10

....................................................................accGgaaagcucuccaaac....................    1    1    s08
......................................................................ccugaaagcucuccaaac....................    3    0    s08
......................................................................ccGgaaagcucuccaaac....................    156  1    s08

.....................................................................Gccugaaagcucuccaaac....................    1    1    s18
......................................................................ccugGaagcucuccaaac....................    1    1    s18
......................................................................ccGgaaagcucuccaaac....................    64   1    s18
```
