# Supplementary material for: The Identification of Circulating MiRNA in Bovine Serum and Their Potential as Novel Biomarkers of Early Mycobacterium avium subsp paratuberculosis Infection
Source: PLoS One. 2015 Jul 28;10(7):e0134310. doi: 10.1371/journal.pone.0134310 (PMC4517789; doi:10.1371/journal.pone.0134310)
Supplement: S1 File — (ZIP) [file pone.0134310.s008.zip › novel_pdfs/9_25151.pdf]

Provisional ID	:	9_25151
Score total	:	4.2
Score for star read(s)	:	-1.3
Score for read counts	:	0
Score for mfe	:	0.9
Score for randfold	:	1.6
Score for cons. seed	:	3
Total read count	:	450
Mature read count	:	436
Loop read count	:	13
Star read count	:	1

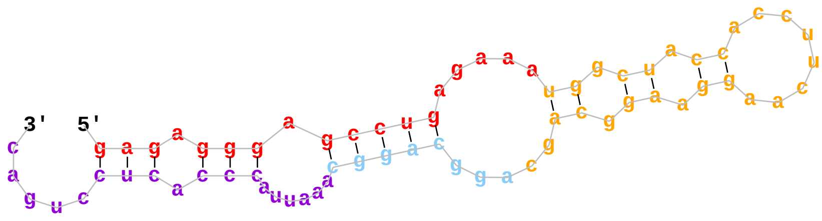

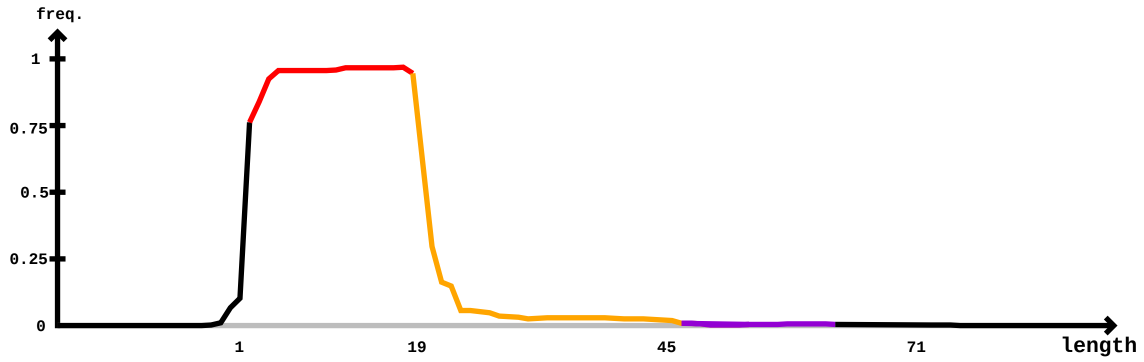

Mature Star

```
5'- agggaaacccuagagagggagagggagccugagaaauggcuaccaccuucaaggaaggcagcaggcaggcaaauuacccacuccugacccagggaggaaguaacaaaa   -3'   obs
    agggaaacccuagagagggagagggagccugagaaauggcuaccaccuucaaggaaggcagcaggcaggcaaauuacccacuccugacccagggaggaaguaacaaaa         exp
    .......((((.(.(.(((((.(((.(((((.....((.((.((........)).)).)).....)))))......))).)))))..)).))))..............   reads   mm   sample
    ..................gagagggagccugagCaa........................................................................   1       1    s07
    ..................gagagggagccugagaaa........................................................................   17      0    s07
    ..................gagaCggagccugagaaa........................................................................   1       1    s07
    ...................agagggagcAugagaaauggcua..................................................................   1       1    s07
    ....................gagggagccugagaaauggc....................................................................   1       0    s07

    ..................Cagagggagccugagaaa........................................................................   1       1    s14
    ..................gagagggagccugagaaa........................................................................   3       0    s14
    ...................agagggagccugagaaaug......................................................................   2       0    s14

    ..................gagaCggagccugagaaa........................................................................   1       1    s19
    ..................gagagggagccugagaaC........................................................................   1       1    s19
    ..................gagagggagccugagaaa........................................................................   31      0    s19
    ..................gagagggagccugagaGa........................................................................   1       1    s19
    .....................agggagUcugagaaauggc....................................................................   1       1    s19

    .................ggagagggagccugagaa.........................................................................   1       0    s09
    .................ggagCgggagccugagaa.........................................................................   1       1    s09
    ..................gagagggagccugagaaa........................................................................   10      0    s09
    ..................gagaCggagccugagaaa........................................................................   1       1    s09
    ..................gagagggagccugagCaa........................................................................   1       1    s09
    ..........................................ccaccuCcaaggaaggcagca.............................................   1       1    s09

    ...............aCggagagggagccugagaaauggc....................................................................   1       1    s05
    ................Cggagagggagccugagaaaug......................................................................   2       1    s05
    ................Cggagagggagccugagaaauggc....................................................................   3       1    s05
    ................Cggagagggagccugagaaauggcua..................................................................   1       1    s05
    .................ggagagggagccugagaa.........................................................................   1       0    s05
    ..................gCgagggagccugagaaa........................................................................   1       1    s05
    ..................gagagggagccugagaaa........................................................................   10      0    s05
    ..................gagagggagcAugagaaaug......................................................................   1       1    s05
    ..................gagagggagccugagaaauggcua..................................................................   1       0    s05
    ...................agagggagccugagaaaug......................................................................   3       0    s05
```

```
                  Mature                                              Star
agggaaacccuagagagggagagggagccugagaaauggcuaccaccuucaaggaaggcagcaggcaggcaaauuacccacuccugacccagggaggaaguaacaaaa
...................agagggagcAugagaaaug......................................................................    1     1     s05
...................agaUggagccugagaaaug......................................................................    1     1     s05
...................agagggagccugagaaauggc....................................................................    2     0     s05
....................gagggagccugagaaCug......................................................................    1     1     s05
....................gagggCgccugagaaaug......................................................................    1     1     s05
....................gagggagccugagaaaug......................................................................    8     0     s05
....................gagggaCccugagaaaug......................................................................    1     1     s05
....................gagUgagccugagaaauggc....................................................................    1     1     s05
....................gagggagccugagaaauggc....................................................................    5     0     s05
....................gagggagccugagaaauggcuac.................................................................    1     0     s05
.....................aggUagccugagaaauggc....................................................................    1     1     s05
.....................agggagccugagaaauggc....................................................................    1     0     s05
............................cugagaaaCggcuaccacc.............................................................    1     1     s05

..................gagagggCgccugagaaa........................................................................    1     1     s22
..................gagagggagccugagaaa........................................................................    3     0     s22
..................gagagggagccugagaGa........................................................................    1     1     s22
..................gUgagggagccugagaaa........................................................................    1     1     s22
..................gagaCggagccugagaaa........................................................................    2     1     s22
...................agagggGgccugagaaaug......................................................................    1     1     s22
....................gagggGgccugagaaaug......................................................................    1     1     s22
....................gaAggagccugagaaauggc....................................................................    1     1     s22
................................................ucaaggaaggcagcaggc..........................................    1     0     s22

.................ggagagggagccugagaaa........................................................................    1     0     s16
..................gagagggagccugagaaa........................................................................    1     0     s16
......................................................................aaauuacccaAuccugac....................    1     1     s16

................Cggagagggagccugagaaaug......................................................................    1     1     s06
................Cggagagggagccugagaaaugg.....................................................................    1     1     s06
................Cggagagggagccugagaaauggc....................................................................    1     1     s06
................Cggagagggagccugagaaauggcua..................................................................    3     1     s06
.................ggagagggagccugagaa.........................................................................    2     0     s06
..................gagaCggagccugagaaa........................................................................    1     1     s06
..................gagagggagccugagaaa........................................................................    16    0     s06
..................gagagggagccugagaGa........................................................................    1     1     s06
..................gagagggagccugagCaaug......................................................................    1     1     s06
..................gagagggagccugagaaaug......................................................................    4     0     s06
...................agagggagccugagaaaug......................................................................    7     0     s06
....................gagggagccugagaaCug......................................................................    1     1     s06
....................gagggagccugagaaaug......................................................................    7     0     s06
....................gagggagccugagaaaugg.....................................................................    1     0     s06
....................gagggagccugagaaauggc....................................................................    2     0     s06
....................gagggagcUugagaaauggcua..................................................................    2     1     s06
.....................agggagccugagaaauggc....................................................................    2     0     s06
.....................agggagUcugagaaauggc....................................................................    1     1     s06
...........................ccCgagaaauggcuacca...............................................................    1     1     s06
..................................aauggcuaccacAuucaaggaa....................................................    1     1     s06
..........................................ccaccuCcaaggaaggcagca.............................................    2     1     s06
............................................acAuucaaggaaggcagc..............................................    1     1     s06

................Cggagagggagccugagaaaug......................................................................    2     1     s17
.................ggagagggagccugagaaauggc....................................................................    2     0     s17
..................gaUagggagccugagaaa........................................................................    1     1     s17
..................gagaCggagccugagaaa........................................................................    1     1     s17
..................gagagggagccugagaaa........................................................................    4     0     s17
...................agagggagccugagaaaug......................................................................    2     0     s17
...................agagggagccugagaGaug......................................................................    2     1     s17
.....................agggagccugagaaauCgc....................................................................    1     1     s17
..........................................ccaccuCcaaggaaggcagca.............................................    1     1     s17
..............................................Auucaaggaaggcagcagg...........................................    1     1     s17

..................gagagggagccugagaaa........................................................................    9     0     s02
....................gagggagccugagaGauggc....................................................................    1     1     s02
.....................agggagccugagaaauggc....................................................................    1     0     s02

..................gagagggagccugagaaa........................................................................    3     0     s13
..................gagagggagccugagCaa........................................................................    1     1     s13

...............aCggagagggagccugagaaauggcuacc................................................................    1     1     s04
```

Mature Star

```
agggaaacccuagagagggagagggagccugagaaauggcuaccaccuucaaggaaggcagcaggcaggcaaauuacccacuccugacccagggaggaaguaacaaaa
................Cggagagggagccugagaaauggc....................................................................  1   1   s04
.................ggagagggagccugagaa.........................................................................  1   0   s04
..................gagaUggagccugagaaa........................................................................  1   1   s04
..................gagagggagccugagaaa........................................................................  13  0   s04
....................gagggagccugagaaauCgc....................................................................  1   1   s04
............................Augagaaauggcuaccac..............................................................  1   1   s04

................Cggagagggagccugagaaaugg.....................................................................  1   1   s15
................Cggagagggagccugagaaauggc....................................................................  1   1   s15
................Cggagagggagccugagaaauggcuacc................................................................  1   1   s15
..................gagagggagccugagaaa........................................................................  2   0   s15
..................gagaAggagccugagaaaugg.....................................................................  2   1   s15
....................gagggagccugagaaauggc....................................................................  1   0   s15
.....................agggagccugagaaauAgc....................................................................  1   1   s15
..........................................................................uacccaAuccugacccag................  1   1   s15

..................gagagggagccugagaaa........................................................................  4   0   s01
....................gagggagccugagaaaug......................................................................  1   0   s01
............................cugagaaaCggcuaccacc.............................................................  1   1   s01

...............Cgggagagggagccugagaaa........................................................................  1   1   s12
.................ggagagggagccugagaaa........................................................................  2   0   s12
..................gagagggagccugagaaa........................................................................  24  0   s12
..................gagaCggagccugagaaa........................................................................  1   1   s12
..................gagagggCgccugagaaa........................................................................  1   1   s12
..................gaUagggagccugagaaa........................................................................  1   1   s12
..................gagagggGgccugagaaaug......................................................................  1   1   s12
...................agaAggagccugagaaaug......................................................................  1   1   s12
.....................agggCgccugagaaaugg.....................................................................  1   1   s12

..................gagagggagccugagaaa........................................................................  3   0   s03
...................agagggGgccugagaaaug......................................................................  1   1   s03

................Cggagagggagccugagaaauggc....................................................................  1   1   s08
..................gagagggagccugagCaa........................................................................  1   1   s08
..................gagaCggagccugagaaa........................................................................  1   1   s08
..................gagagggagccugagaaa........................................................................  19  0   s08
.......................................cuaccaccuCcaaggaag...................................................  1   1   s08
.................................................caaggaaggcagcaggcaCgcaaauuaccc.............................  1   1   s08

................Cggagagggagccugagaaaugg.....................................................................  2   1   s10
..................gagagggagccugagaaa........................................................................  19  0   s10
..................gagagggagccugaAaaa........................................................................  1   1   s10
..................gagagggCgccugagaaa........................................................................  1   1   s10
...................agagggagccugagaaaA.......................................................................  1   1   s10
...................agagggGgccugagaaaug......................................................................  1   1   s10
...................agagggagccugagaaaug......................................................................  1   0   s10
...................agagggagcAugagaaaug......................................................................  1   1   s10
...................agagggagccugagaaauCgcuacc................................................................  1   1   s10
....................gagUgagccugagaaaug......................................................................  1   1   s10
.....................agggagccugagaaauCgc....................................................................  1   1   s10
.....................agggagccugagaaauggcuacc................................................................  1   0   s10

................Cggagagggagccugagaaauggc....................................................................  1   1   s18
.................ggagagggagccugagaaauggc....................................................................  1   0   s18
..................gagagggagccugagaaa........................................................................  3   0   s18

...............agggagagggagccugagaaaCggcuacc................................................................  1   1   s11
................Cggagagggagccugagaaauggc....................................................................  1   1   s11
.................ggagagggagccugagaa.........................................................................  2   0   s11
..................gUgagggagccugagaaa........................................................................  1   1   s11
..................gagagggagccugagaaa........................................................................  20  0   s11
..................gagagggagccugagCaa........................................................................  1   1   s11
..................gagagggagccugagaGa........................................................................  1   1   s11
...................agagggagccugagaaauCgcuacc................................................................  1   1   s11
..........................................ccaccuCcaaggaaggca................................................  1   1   s11

................Cggagagggagccugagaaauggc....................................................................  1   1   s23
.................Cgagagggagccugagaaa........................................................................  1   1   s23
..................gagagggagccugagaaa........................................................................  17  0   s23
```

```
                  Mature                                              Star
agggaaacccuagagagggagagggagccugagaaauggcuaccaccuucaaggaaggcagcaggcaggcaaauuacccacuccugacccagggaggaaguaacaaaa
..................gagGgggagccugagaaa........................................................................   1    1    s23
..................gagaCggagccugagaaa........................................................................   1    1    s23
..................gaUagggagccugagaaa........................................................................   1    1    s23
...................agaAggagccugagaaaug......................................................................   1    1    s23
..........................................ccaccuCcaaggaaggcag...............................................   1    1    s23

................Cggagagggagccugagaaauggc....................................................................   2    1    s24
.................ggagagggagccugagaa.........................................................................   1    0    s24
..................gagagCgagccugagaaa........................................................................   1    1    s24
..................gGgagggagccugagaaa........................................................................   1    1    s24
..................gagCgggagccugagaaa........................................................................   1    1    s24
..................gagagggagccugagCaa........................................................................   1    1    s24
..................gagagggagccugagaaa........................................................................   18   0    s24
...................agagggagccugagaaauggc....................................................................   1    0    s24
...................agagggagccugagaaaAggcuac.................................................................   1    1    s24
.....................agggagccugagaaauggcuacc................................................................   1    0    s24
........................................uaccacAuucaaggaaggcagcaggc..........................................   1    1    s24

..............gCgggagagggagccugagaa.........................................................................   1    1    s21
................Cggagagggagccugagaaaug......................................................................   1    1    s21
..................gagagggagccugagaaa........................................................................   5    0    s21
..................gagagggagccugagaGa........................................................................   1    1    s21
..................gagagggagccugagCaa........................................................................   1    1    s21
..................gagagggagccugagaaaug......................................................................   1    0    s21
...................agagggagccugagaaaugUcuaccac..............................................................   1    1    s21
....................gagggagccugagaaauggc....................................................................   2    0    s21
.....................agggagccugagaaauggc....................................................................   1    0    s21
..........................................ccaccuCcaaggaaggcagca.............................................   1    1    s21

.................ggagagggagccugagaa.........................................................................   1    0    s20
..................gagCgggagccugagaaa........................................................................   1    1    s20
..................gagaCggagccugagaaa........................................................................   1    1    s20
..................gUgagggagccugagaaa........................................................................   1    1    s20
..................gagagggagccugagaaa........................................................................   9    0    s20
...................agaAggagccugagaaaug......................................................................   1    1    s20
...................agagggagccugagaaaug......................................................................   2    0    s20
.....................agggagccugagaaauCgcua..................................................................   1    1    s20
............................cugagaaaCggcuaccacc.............................................................   1    1    s20
```
